# Supplementary material for: Chemoselective dual functionalization of proteins via 1,6-addition of thiols to trifunctional N-alkylpyridinium
Source: Nat Commun. 2025 Jun 6;16:5278. doi: 10.1038/s41467-025-60237-y (PMC12144261; doi:10.1038/s41467-025-60237-y)
Supplement: Supplementary file 1 — Supplementary Information [file 41467_2025_60237_MOESM1_ESM.pdf]

Supplementary Information

Chemoselective Dual Functionalization of Proteins *via* 1,6-Addition of Thiols to Trifunctional *N*-Alkylpyridinium

Lujuan Xu,[a,b,#] Maria J. S. A. Silva,[b,#] Jaime A. S. Coelho,[c] Joscha Borho,[d] Nicole Stadler,[d] Holger Barth,[d] Seah Ling Kuan,[b*] and Tanja Weil[b*]

a. Hangzhou Institute of Medicine, Chinese Academy of Sciences, Hangzhou, Zhejiang, 310018, China
b. Max Planck Institute for Polymer Research, 55128, Mainz
c. Centro de Química Estrutural, Institute of Molecular Sciences, Faculty of Sciences, University of Lisbon, 1749-016 Lisbon, Portugal
d. Institute of Experimental and Clinical Pharmacology, Toxicology and Pharmacology of Natural Products, Ulm University Medical Center, Albert-Einstein-Allee 11, 89081 Ulm, Germany.
*Corresponding authors; #both authors contributed equally

Index

1 Materials and methods ... 4
1.1 Materials ... 4
1.2 Methods ... 4
1.2.1 Nuclear magnetic resonance spectroscopy ... 4
1.2.2 High-Performance Liquid Chromatography (HPLC) ... 4
1.2.3 Liquid Chromatography - Mass Spectrometry (LC-MS) ... 4
1.2.4 Electrospray Ionization-High Resolution Mass Spectrometry ... 5
1.2.5 Matrix-Assisted Laser Desorption/Ionization - Time of Flight (MALDI-ToF) ... 5
1.2.6 Circular Dichroism Spectroscopy (CD) ... 5
2 Synthesis of the *N*-alkylpyridinium derivatives ... 6
2.1 Synthesis of compound 1 ... 6
2.2 Synthesis of compound 2 ... 7
2.3 Synthesis of compound 3 ... 8
2.4 Synthesis of compound 4 ... 9
2.5 Synthesis of compound 5 ... 10
2.6 Synthesis of compound 6 ... 10
2.7 Synthesis of RGDC-DBCO ... 12
2.8 Synthesis of PC8 maleimide conjugate ... 13
3 CLog P (n-octanol to water) prediction of *N*-alkylpyridinium derivatives based on ChemDraw ... 13
4 Evaluation of the chemoselectivity and the reaction kinetics of the *N*-alkylpyridium derivatives ... 15
4.1 Chemoselectivity check of *N*-alkylpyridinium derivatives towards thiol and amino groups 15
4.2 Evaluation of the reactivity of *N*-alkylpyridinium derivatives towards different amino acids 16
4.3 Reactivity check at different pH ... 17
4.4 Kinetics study of compound 1 with compound 7 ... 18
5 Stability study of the *N*-alkylpyridinium derivatives ... 19
6 NMR characterization of product 9 ... 21
7 Computational Methods ... 24
7.1 Computational design of new *N*-alkylpyridinium reagent ... 25
7.2 Frontier molecular orbital (FMO) analysis ... 26
7.3 Reaction potential energy surfaces for different acceptors ... 27
7.4 Proton affinity study ... 28
8 Site-selective modification of peptides ... 30
8.1 General procedure for modification of peptides with *N*-alkylpyridinium derivatives. 30
8.1.1 PC8 conjugate 11 ... 30
8.1.2 CEIE conjugate ... 35
8.1.3 WSC02 conjugate ... 37
8.1.4 RGDC conjugate ... 42
8.1.5 Tet conjugate ... 46
8.1.6 EK1C conjugate ... 50
8.2 Stability studies PC8 conjugate (11) and PC8-maleimide conjugate ... 54
8.2.1 Stability under different pHs ... 54
8.2.2 Stability of PC8 conjugate (11) and PC8-maleimide conjugate in 1 mM glutathione (GSH) in physiological pH ... 63
8.3 Site-selective modification of PC8 peptide and cysteine masking with 4-DPS ... 68
8.4 Comparison of the modification specificity of Tet peptide with using compound 1 and maleimide ... 69
9 Site-selective modification of proteins ... 73
9.1 Site-selective modification of ubiquitin-K63C ... 74
9.2 Site-selective modification of the anti-MMR nanobody ... 85
9.3 Modification of trastuzumab ... 90
9.3.1 Trastuzumab modification with reagent 6 and TCO-sulfoCy5 ... 91
9.3.2 Trastuzumab modification with Maleimide-sulfoCy5 ... 94
9.3.3 Trastuzumab reduction and deglycosylation with PNGaseF ... 98

9.4 Site-selective modification of the Cys-C3bot1 100
9.5 Dual-functionalization of Cys-C3bot1 with Cy3, Cy5 or RGDC 106
10 *In vitro* studies 111
10.1 Confocal microscopy 111
10.2 Intoxication studies 111
10.3 Western Blot analysis for ADP-ribosylation 111
11 Supporting figures 113
12 Supplementary References 128

1 Materials and methods

1.1 Materials

Unless otherwise stated, all solvents and reagents were bought from Merck, Sigma Aldrich, TCI, Click Chemistry Tools and Lumiprobe, and used directly for the reaction. HER-2 (Trastuzumab) Recombinant Human Monoclonal Antibody (4D5-8) (Article number: LT1500-1MG, Leinco Technologies, St. Louis, Missouri, USA, Lot: 1024L615) and PNGase F Glycan Cleavage Kit (Article number: A39245, Gibco™ Lot: 299434). The High-Performance Liquid Chromatography (HPLC) was performed with HPLC grade acetonitrile (ACN) and Milli-Q water. The reactions were monitored by thin-layer chromatography (TLC) with Macherey-Nagel Alugram Sil G/UV254 plates at 254 nm. Flash column chromatography was carried out with silica gel (0.04mm – 0.063 mm, 60Å).

1.2 Methods

1.2.1 Nuclear magnetic resonance spectroscopy

The NMR spectra are recorded using Bruker Avance 300, 400, 500 and 850 MHz NMR Fourier transform spectrometers using the deuterated solvents, such as $CDCl_3$, CD_2Cl_2, CD_3OD, $(CD_3)_2SO$. All coupling constants are expressed in Hz and chemical shifts (δ) in ppm using the residual solvent peaks as reference. Multiplicities are given as: s (singlet), brs (broad singlet), d (doublet), dd (double doublet), dt (double triplet), t (triplet), td (triple doublet), tt (triple triplet), q (quartet), quint (quintuplet) and m (multiplet). Acquired data was processed with MestreNova 15.0.

1.2.2 High-Performance Liquid Chromatography (HPLC)

Preparative HPLC was performed with a Shimazu LC-20AP system with using Phenomenex Gemini 5 µm NX-C18 110 Å 150 × 30 mm at the flow rate of 25 mL/min. The gradient started with 5% ACN, which was linearly increased to 100% ACN within 20 min.

Semi-preparative and analytical HPLC were performed using Shimadzu LC-20AT system. A ZORBAX Eclipse XDB-C18 HPLC column (80 Å, 9.4 × 250 mm, 5 µm) was used at a flow rate of 4 mL/min. Atlantis T3 column (100 Å, 4.6 × 100 mm, 5 µm) and Zorbax Eclipse XDB-C18 column, (4.6 × 250 mm, 5 µm) both with a flow rate of 1 mL/min. The general HPLC method started with 5% ACN, which was increased to 100% ACN within 20 min with 0.1% TFA. For some of the compounds, neutrals eluting solvents instead of the acidic ones were also used for HPLC purification. All HPLC quantification was performed with one run using an internal standard and the acquired data was processed with LabSolutions provided by Shimadzu.

1.2.3 Liquid Chromatography - Mass Spectrometry (LC-MS)

LC-MS analysis was performed on a Shimadzu LC-MS 2020 equipped with an electrospray ionization source and a SPD-20A UV-Vis detector. Milli-Q water and ACN, both with 0.1% formic acid, were used as mobile phases. The method started with 5% ACN and 95% water, reached 100% ACN at 16 min, and then went back to 5% ACN and 95% water at 20 min.

Acquired data were processed with LabSolutions provided by Shimadzu.

1.2.4 Electrospray Ionization-High Resolution Mass Spectrometry

The ESI-HRMS spectra were recorded using a SYNAPT G2-Si mass spectrometer (Waters Corp., Manchester, UK) equipped with an ESI source. The instrument was calibrated by clusters of sodium iodide in the mass range of 100-2000 *m/z*. The measurements were carried out at capillary voltage of 2–3 kV, sampling cone 20 and 40 V and source offset 50 and 80 V, and a source temperature of 100 °C. Nitrogen was used as the desolvation gas at a total flow of 500 L h^{-1}. The samples were infused at a flow rate of 5 µL/min by a syringe pump (Legato 180 from Kd Scientific, Holliston, MA, USA) into the mass spectrometer. Data processing was done with MassLynx software V4.2 (Waters GmbH, Eschborn, Germany, 2005). The deconvolution data were obtained from the full range of the combined ion series using the MaxEnt software.

Intact Protein LC-MS analysis was conducted on the same mass spectrometer, coupled to an Acquity UPLC system using an Acquity UPLC Protein BEH C4 column (300Å, 1.7 µm, 2.1 × 50 mm). Water with 0.1% formic acid (solvent A) and acetonitrile 0.1% formic acid (solvent B), were used as the mobile phase at a flow rate of 0.2 mL. The gradient was t = 0 min, 0% B; t = 2 min, 0% B; t = 18 min, 90% B; t = 19 min, 90% B; t = 20 min, 0% B, stop, detection UV/Vis detection with Diode Array (200-800 nm). The electrospray source was operated with capillary voltage of 2.0 kV, sampling cone of 40 V and source offset 80 V. Source temperature was 150 °C, desolvation temperature was 500 °C and desolvation gas at a total flow of 800 L·h^{-1}. The samples were injected and analyzed once, unless stated otherwise.

1.2.5 Matrix-Assisted Laser Desorption/Ionization - Time of Flight (MALDI-ToF)

MALDI-ToF measurements were conducted on a rapifleX MALDI-ToF/ToF mass spectrometer from Bruker with a 10 kHz scanning smartbeam three-dimensional laser (Nd:YAG at 355 nm) and a 10 bit 5 GHz digitizer in positive ion reflector mode. Calibration was done with the Bruker BSA protein calibration standard in a mass range from 20 to 220 kDa. Protein sample solutions were mixed 1:10 with a sinapinic acid matrix (saturated solution in ACN/water 1:1 with 0.1 % TFA) and applied on the target. Then, the mixtures were left in the fume hood for cocrystallization (ca. 15 min). Samples were measured at a laser power of 100 % with random walk ionization across the sample spot. The acquired data was processed with mMass version 5.5.0.

1.2.6 Circular Dichroism Spectroscopy (CD)

Circular dichroism was measured at rt from 260 to 195 nm with a bandwidth of 1 nm at 0.1 mg/mL in PB buffer (50 mM, pH 7.4). The data pitch was set to 0.2 nm with the scanning speed of 5 nm/min. Each sample was measured three times and the signal from the buffer blank was subtracted from the sample scan. The obtained data was processed in the software Spectra Analysis and CD Multivariate SSE by JASCO.

2 Synthesis of the *N*-alkylpyridinium derivatives

2.1 Synthesis of compound 1

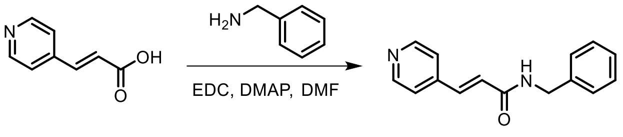

12

Commercially available compound (*E*)-3-(pyridin-4-yl)acrylic acid (400 mg, 2.68 mmol, 1 equiv) was dissolved in 40 mL DMF. After that, EDC.HCl (617 mg, 3.22 mmol, 1.2 equiv) and DMAP (39.3 mg, 0.32 mmol, 0.12 equiv) were added and the resultant mixture was stirred at room temperature (rt) for 30 min. Then, benzylamine (345 mg, 3.22 mmol, 1.2 equiv) was added and the reaction mixture was stirred overnight at rt. After that, the solvent was evaporated under reduced pressure and the crude product was purified by flash column chromatography (DCM:MeOH = 20:1) to get a colorless oil (454 mg, 71% yield).

^{1}H NMR (300 MHz, CD_2Cl_2) δ 8.57 (d, *J* = 5.8 Hz, 2H), 7.54 (d, *J* = 15.6 Hz, 1H), 7.39 – 7.23 (m, 7H), 6.64 (d, *J* = 15.6 Hz, 1H), 6.39 (brs, 1H), 4.54 (d, *J* = 5.9 Hz, 2H).

^{13}C NMR (75 MHz, CD_2Cl_2) δ 164.9, 150.8, 142.5, 138.8, 138.6, 129.1, 128.1, 127.9, 125.6, 122.1, 44.1.

LC-MS: calculated: 239.1 $[M+H]^+$, found: 239.1 $[M+H]^+$.

ESI-HRMS calculated for $C_{15}H_{15}N_2O^+$ $[M+H]^+$: 239.1179, found 239.1186.

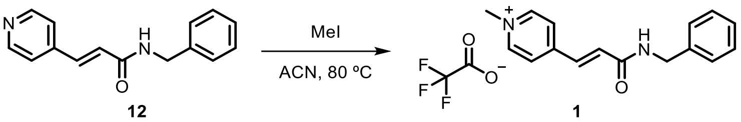

12 **1**

(*E*)-*N*-benzyl-3-(pyridin-4-yl)acrylamide **12** (98.6 mg, 0.41 mmol, 1 equiv) was dissolved in 6 mL ACN . Next, iodomethane (294 mg, 2.07 mmol, 5 equiv) was added and the reaction mixture was stirred at 80°C for overnight. After that, the solvent was evaporated under reduced pressure and the resultant residue was purified by preparative HPLC (with 0.1% TFA in the solvents) to get compound 1 (93 mg, 62% yield).

^{1}H NMR (300 MHz, CD_3OD) δ 9.30 (d, *J* = 6.6 Hz, 2H), 8.64 (d, *J* = 6.6 Hz, 2H), 8.13 (d, *J* = 15.8 Hz, 1H), 7.79 (d, *J* = 4.4 Hz, 4H), 7.76 – 7.68 (m, 1H), 7.64 (d, *J* = 15.8 Hz, 1H), 4.98 (s, 2H), 4.83 (s, 3H).

^{13}C NMR (75 MHz, CD_3OD) δ 165.9, 152.6, 146.9, 139.4, 135.2, 133.6, 129.7, 128.8, 128.5, 126.7, 48.4, 44.6.

LC-MS: calculated: 253.3 $[M+H]^+$, found: 253.1 $[M+H]^+$.

ESI-HRMS calculated for $C_{16}H_{17}N_2O^+$ $[M]^+$: 253.1338, found 253.1335.

2.2 Synthesis of compound 2

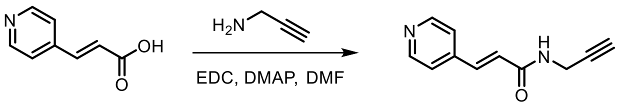

13

Commercially available compound (*E*)-3-(pyridin-4-yl)acrylic acid (200 mg, 1.34 mmol, 1 equiv) was dissolved in 20 mL DMF. After that, EDC.HCl (308 mg, 1.61 mmol, 1.2 equiv) and DMAP (20 mg, 0.16 mmol, 0.12 equiv) were added and the resultant mixture was stirred at rt for 30 min. Then, the prop-2-yn-1-amine (88.6 mg, 1.61 mmol, 1.2 equiv) was added and the reaction mixture was stirred overnight at rt. After that, the solvent was evaporated under reduced pressure and the crude product was purified by flash column chromatography (DCM:MeOH = 20:1) to get **13** as a yellow solid (172 mg, 69% yield).

^{1}H NMR (300 MHz, CD_3OD) δ 8.56 (d, *J* = 5.8 Hz, 2H), 7.57 (dd, *J* = 6.1, 2H), 7.52 (d, *J* = 16.0 Hz, 1H), 6.83 (d, *J* = 15.8 Hz, 1H), 4.10 (d, *J* = 2.5 Hz, 2H), 2.65 (t, *J* = 2.5 Hz, 1H).

^{13}C NMR (75 MHz, CD_3OD) δ 166.9, 150.7, 144.8, 138.9, 126.9, 123.5, 80.2, 72.6, 29.7.

LC-MS: calculated: 187.1 $[M+H]^+$, found: 187.1 $[M+H]^+$.

ESI-HRMS calculated for $C_{11}H_{11}N_2O^+$ $[M+H]^+$: 187.0866, found 187.0864.

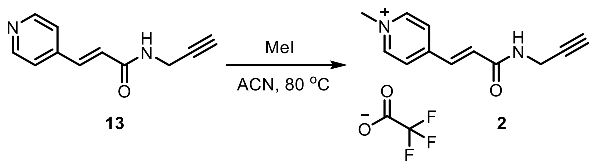

The resultant compound **13** (53.8 mg, 0.29 mmol, 1 equiv) was dissolved in 2 mL ACN first. Next, MeI (821 mg, 5.78 mmol, 20 equiv) was also added and the resultant reaction mixture was stirred overnight at 80°C. After that, the solvent was evaporated under reduced pressure and the resultant residue was purified by preparative HPLC (with 0.1% TFA in the solvents) to get compound **2** as a white solid (53 mg, 59% yield).

^{1}H NMR (300 MHz, $(CD_3)_2SO$*)* δ 8.95 (d, *J* = 6.7 Hz, 2H), 8.24 (d, *J* = 6.7 Hz, 2H), 7.63 (d, *J* = 15.8 Hz, 1H), 7.10 (d, *J* = 15.9 Hz, 1H), 4.30 (s, 3H), 4.03 (dd, *J* = 5.5, 2.5 Hz, 2H), 3.20 (t, *J* = 2.5 Hz, 1H).

^{13}C NMR (75 MHz, CD_3OD) δ 165.6, 152.5, 147.0, 135.6, 132.9, 126.8, 79.9, 72.8, 54.8, 29.9.

LC-MS: calculated: 201.1 $[M+H]^+$, found: 201.1 $[M+H]^+$.

ESI-HRMS calculated for $C_{12}H_{13}N_2O^+$ $[M]^+$: 201.1022, found 201.1026.

2.3 Synthesis of compound 3

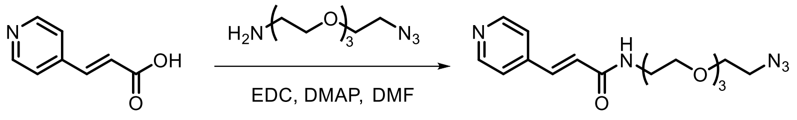

14

Commercially available compound (*E*)-3-(pyridin-4-yl)acrylic acid (150 mg, 1.00 mmol, 1 equiv) was dissolved in 15 mL DMF. After that, EDC.HCl (231 mg, 1.20 mmol, 1.20 equiv) and DMAP (14.7 mg, 0.12 mmol, 0.12 equiv) were added and the resultant mixture was stirred at rt for 30 min. Then, the 2-(2-azidoethoxy)ethan-1-amine (263 mg, 1.00 mmol, 1.20 equiv) was added and the reaction mixture was stirred overnight at rt. After that, the solvent was evaporated under reduced pressure and the crude product was purified by flash column chromatography (DCM:MeOH = 20:1) to get compound **14** as a yellow oil (309 mg, 88% yield).

^{1}H NMR (300 MHz, $CDCl_3$) δ 8.60 (d, *J* = 4.6 Hz, 2H), 7.53 (d, *J* = 15.7 Hz, 1H), 7.33 (d, *J* = 5.8 Hz, 2H), 6.61 (d, *J* = 15.7 Hz, 1H), 3.73 – 3.51 (m, 14H), 3.36 (t, *J* = 5.2 Hz, 2H).

^{13}C NMR (75 MHz, $CDCl_3$) δ 165.0, 150.5, 142.4, 138.1, 125.5, 121.8, 70.8, 70.7, 70.6, 70.4, 70.1, 69.8, 50.8, 39.7.

LC-MS: calculated: 350.2 $[M+H]^+$, found: 350.1 $[M+H]^+$.

ESI-HRMS calculated for $C_{16}H_{24}N_5O_4^+$ $[M+H]^+$: 350.1823, found 350.1836.

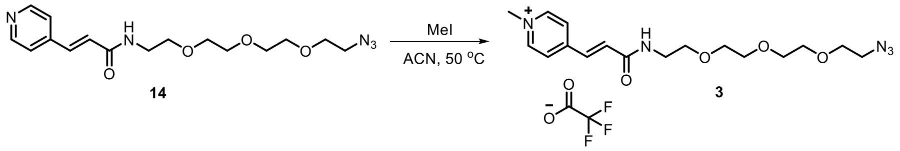

14 **3**

The resultant compound **14** (42.1 mg, 0.12 mmol, 1 equiv) was dissolved in 5 mL ACN first. Next, MeI (342 mg, 2.41 mmol, 20 equiv) was added and the resultant reaction mixture was stirred at 50°C for overnight. After that, the solvent was evaporated under reduced pressure and the resultant residue was purified by preparative HPLC (with 0.1% TFA in the solvents) to get compound **3** as a colorless oil (39 mg, 68% yield).

^{1}H NMR (300 MHz, CD_3OD) δ 8.86 (d, *J* = 6.4 Hz, 2H), 8.19 (d, *J* = 6.4 Hz, 2H), 7.65 (d, *J* = 15.8 Hz, 1H), 7.16 (d, *J* = 15.8 Hz, 1H), 4.38 (s, 3H), 3.70 – 3.60 (m, 12H), 3.53 (t, *J* = 5.3 Hz, 2H), 3.36 (t, *J* = 4.9 Hz, 2H).

^{13}C NMR (75 MHz, CD_3OD) δ 166.0, 152.7, 147.0, 135.0, 133.6, 126.7, 71.7, 71.6, 71.5, 71.3, 71.1, 70.3, 51.8, 40.9.

LC-MS: calculated: 364.4 $[M+H]^+$, found: 364.2 $[M+H]^+$.

ESI-HRMS calculated for $C_{17}H_{26}N_5O_4^+$ $[M]^+$: 364.1979, found 364.1985.

2.4 Synthesis of compound 4

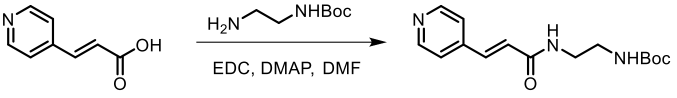

15

Commercially available compound (*E*)-3-(pyridin-4-yl)acrylic acid (500 mg, 3.35 mmol, 1.2 equiv) was dissolved in 50 mL DMF. After that, EDC.HCl (643 mg, 3.22 mmol, 3.35 equiv) and DMAP (41 mg, 0.33 mmol, 0.12 equiv) were added and the resultant mixture was stirred at rt for 30 min. Then, the *tert*-butyl (2-aminoethyl)carbamate (447 mg, 2.79 mmol, 1 equiv) was added and the reaction mixture was stirred overnight at rt. After that, the solvent was evaporated under reduced pressure and the crude product was purified by flash column chromatography (DCM:MeOH = 15:1) to get compound **15** as a white solid (762 mg, 78% yield).

^{1}H NMR (300 MHz, CD_2Cl_2) δ 8.58 (d, *J* = 4.5 Hz, 2H), 7.48 (d, *J* = 15.8 Hz, 1H), 7.36 (d, *J* = 4.6 Hz, 2H), 6.78 (brs, 1H), 6.62 (d, *J* = 15.8 Hz, 1H), 5.15 (brs, 1H), 3.45 (q, *J* = 5.1 Hz, 2H), 3.30 (q, *J* = 5.5 Hz, 2H), 1.42 (s, 9H).

^{13}C NMR (75 MHz, CD_2Cl_2) δ 165.6, 157.4, 150.6, 142.8, 138.0, 126.0, 122.1, 79.9, 41.5, 40.6, 28.5.

LC-MS calculated: 292.3 $[M+H]^+$, found: 292.2 $[M+H]^+$.

ESI-HRMS calculated for $C_{15}H_{22}N_3O_3^+$ $[M+H]^+$: 292.1656, found 292.1667.

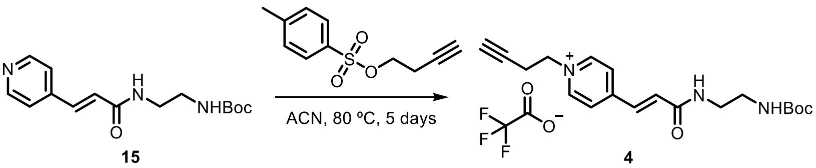

15 **4**

Compound **14** (9.7 mg, 0.033 mmol, 1 equiv) was dissolved in 5 mL ACN followed by the addition of but-3-yn-1-yl 4-methylbenzenesulfonate (15 mg, 0.067 mmol, 2 equiv). The resultant mixture was stirred at 80°C for 5 days. After that, the solvent was evaporated and the crude products were purified by HPLC in neutral conditions to get compound **4** (5.2 mg, 34% yield).

^{1}H NMR (300 MHz, CD_3OD) δ 8.98 (d, *J* = 6.7 Hz, 2H), 8.24 (d, *J* = 6.7 Hz, 2H), 7.66 (d, *J* = 15.8 Hz, 1H), 7.14 (d, *J* = 15.8 Hz, 1H), 4.75 (t, *J* = 6.3 Hz, 2H), 3.41 (t, *J* = 5.2 Hz, 2H), 3.24 (t, *J* = 6.1 Hz, 2H), 2.99 (td, *J* = 6.3, 2.6 Hz, 2H), 2.650 (t, *J* = 2.6 Hz, 1H), 1.47 (s, 9H).

^{13}C NMR (75 MHz, CD_3OD) δ 166.2, 158.6, 153.6, 146.4, 135.0, 134.0, 126.9, 80.2, 79.0, 74.9, 60.5, 54.8, 41.0, 40. 8, 28.7, 21.7.

LC-MS calculated: 344.2 $[M]^+$, found: 344.2 $[M]^+$.

ESI-HRMS calculated for $C_{19}H_{26}N_3O_3^+$ $[M+H]^+$: 344.1969, found 344.1982.

2.5 Synthesis of compound 5

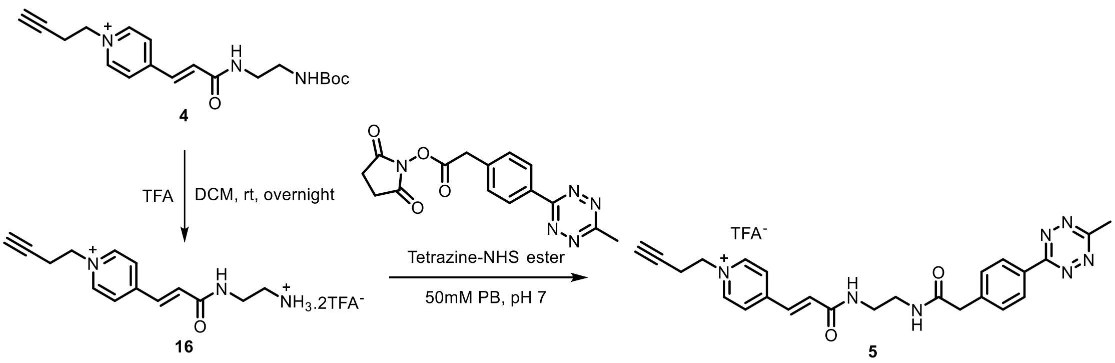

Compound **4** (100 mg, 0.29 mmol, 1 equiv) was dissolved in 4 mL DCM. TFA (995 mg, 8.71 mmol, 30 equiv) was added and the resultant mixture was stirred overnight at rt. After that, the solvent was evaporated and the obtained crude product **16** was used for the next step without further purification.

Then, from the crude of compound **16** (10 mg, 0.021 mmol, 1 equiv) was dissolved in 2 mL ACN: PB buffer (50 mM, pH7.4) and the Tetrazine-NHS ester (11 mg, 0.034 mmol, 1.6 equiv) was added. The mixture was stirred overnight at rt. After that, the resultant solution was purified via HPLC (with 0.1% TFA in the solvents) and compound **5** was obtained as a pink solid (10.9 mg, 90% yield).

^{1}H NMR (300 MHz, CD_3OD) δ 8.97 (d, *J* = 6.6 Hz, 2H), 8.37 (d, *J* = 8.3 Hz, 2H), 8.21 (d, *J* = 6.6 Hz, 2H), 7.59 (d, *J* = 15.8 Hz, 1H), 7.52 (d, *J* = 8.3 Hz, 2H), 7.05 (d, *J* = 15.8 Hz, 1H), 4.77 (t, *J* = 6.4 Hz, 2H), 3.63 (s, 2H), 3.55 – 3.41 (m, 4H), 3.04 (s, 3H), 3.01 (td, *J* = 6.4, 2.5 Hz, 2H), 2.58 (t, *J* = 2.5 Hz, 1H).

^{13}C NMR (75 MHz, CD_3OD) δ 173.9, 168.7, 166.3, 165.2, 153.4, 146.4, 141.9, 135.0, 133.9, 132.0, 131.2, 129.0, 126.9, 79.0, 74.9, 60.5, 43.8, 40.5, 40.0, 21.7, 21.1.

LC-MS: calculated: 456.2 $[M+H]^+$, found: 456.2 $[M+H]^+$.

ESI-HRMS calculated for $C_{25}H_{26}N_7O_2^+$ $[M+H]^+$: 456.2142, found 456.2141.

2.6 Synthesis of compound 6

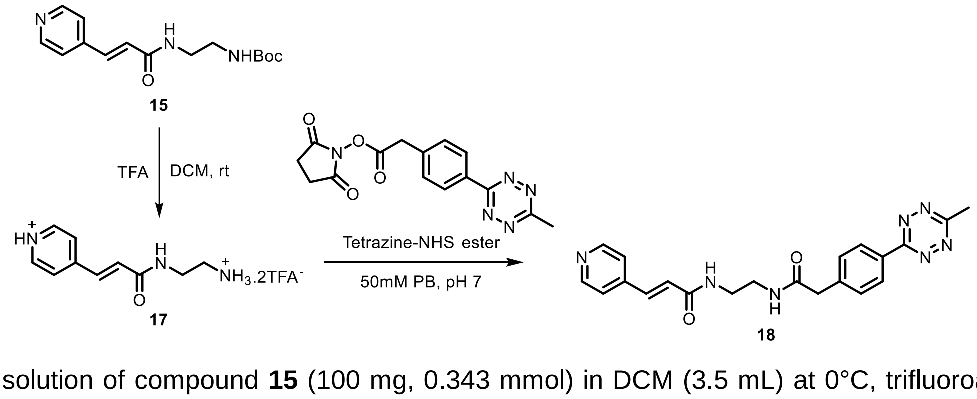

To a solution of compound **15** (100 mg, 0.343 mmol) in DCM (3.5 mL) at 0°C, trifluoroacetic acid (0.5 mL, 6.53 mmol) was added dropwise. The reaction was stirred for 2h at rt and the solvent was evaporated under vacuum. Then, the residue precipitate from MeOH and Et_2O to

afford desired product **17** as a white solid and used directly for the next step without further purification.

From the crude of compound **17** (12.3 mg, 0.029 mmol, 1.1 equiv) was dissolved in 5 mL DCM. Next, DIEA (8.3 mg, 0.064 mmol, 2.5 equiv) and tetrazine-NHS ester (8.7 mg, 0.026 mmol, 1 equiv) were added sequentially, and the resultant mixture was stirred overnight at rt. The crude product was purified by flash column chromatography (DCM:MeOH = 10:1) to obtain compound **18** as a pink solid (9.4 mg, 88% yield).

^{1}H NMR (400 MHz,) δ 8.54 (s, 2H), 8.41 (dt, *J* = 8.5, 2.0 Hz, 2H), 7.60 (d, *J* = 6.0 Hz, 2H), 7.53 (dt, *J* = 8.5, 1.9 Hz, 2H), 7.42 (d, *J* = 15.8 Hz, 1H), 6.74 (d, *J* = 15.8 Hz, 1H), 3.63 (s, 2H), 3.51 – 3.39 (m, 4H), 3.02 (s, 3H).

^{13}C NMR (101 MHz, CD_3OD) δ 173.8, 168.7, 167.4, 165.1, 149.2, 146.6, 141.9, 137.8, 132.0, 131.1, 128.9, 128.5, 124.1, 43.9, 40.2, 40.0, 21.1.

LC-MS: calculated: 404.2 $[M+H]^+$, found: 404.2 $[M+H]^+$.

ESI-HRMS calculated for $C_{21}H_{22}N_7O_2^+$ $[M+H]^+$: 404.1829, found 404.1842.

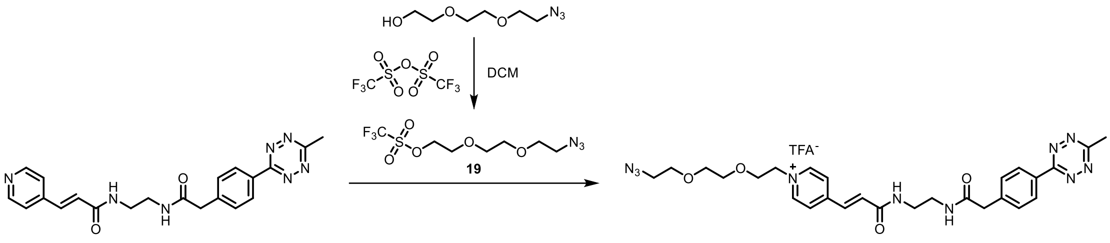

2-(2-(2-azidoethoxy)ethoxy)ethan-1-ol (30 mg, 0.17 mmol, 1 equiv) was dissolved in 4 mL anhydrous DCM, followed by the addition of 2,6-dimethyl pyridine (37 mg, 0.34 mmol, 2 equiv). After that, trifluoromethane sulfonic anhydride (72 mg, 0.25 mmol, 1.5 equiv) was added at 0 ^oC and the resultant mixture was stirred for 30 min. Without further purification, the crude compound **19** was used for the next step directly. Compound **18** (8.2 mg, 0.020 mmol, 1 equiv) was dissolved in 4 mL anhydrous DCM. Compound **19** (12.5 mg, 0.041 mmol, 2 equiv) was added and the resultant mixture was stirred overnight at rt. Thereafter, the reaction mixture was purified by HPLC (with 0.1% TFA in the solvents) and compound **6** was obtained as a pink solid (9.8 mg, 72% yield).

^{1}H NMR (500 MHz, CD_3OD) δ 8.90 (d, *J* = 6.9 Hz,2H), 8.38 (d, *J* = 8.4 Hz, 2H), 8.17 (d, *J* = 6.9 Hz, 2H), 7.58 (d, *J* = 15.7 Hz, 1H), 7.54 – 7.50 (m, 2H), 7.02 (d, *J* = 15.8 Hz, 1H), 4.82 – 4.77 (m, 2H), 4.04 – 3.98 (m, 2H), 3.68 – 3.64 (m, 5H), 3.63 (s, 2H), 33.61 – 3.57 (m, 2H), 3.52 – 3.48 (m, 2H), 3.33 (t, *J* = 4.9 Hz, 2H), 3.04 (s, 3H).

^{13}C NMR (126 MHz, CD_3OD) δ 173.9, 168.8, 166.3, 165.2, 153.1, 146.7, 141.9, 135.1, 133.7, 132.0, 131.2, 129.0, 126.7, 71.6, 71.4, 71.0, 70.1, 62.3, 51.7, 43.8, 40.5, 40.0, 21.1.

LC-MS: calculated: 561.6 $[M+H]^+$, found: 561.3 $[M+H]^+$.

ESI-HRMS calculated for $C_{27}H_{33}N_{10}O_4^+$ $[M+H]^+$: 561.2681, found 561.2706.

2.7 Synthesis of RGDC-DBCO

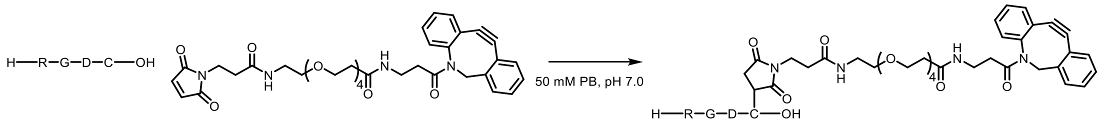

To a solution of RGDC.TFA$^-$ peptide (2.014 mg, 3.58 μmol) in 50 mM PB buffer, pH 7 (1440 μL) was added Maleimide-PEG$_4$-DBCO (57.8 μL, 4.28 μmol) and the mixture stirred for 1h at 37 °C. The desired conjugate was purified in Zorbax Eclipse XDB-C18 HPLC column (80 Å, 9.4 × 250 mm, 5 μm) at a flow rate of 4 mL/min with acetonitrile (solvent A, containing 0.1% *v/v* TFA) and Milli-Q water (solvent B, containing 0.1% *v/v* TFA). Mobile phase t = 0 min, 5% B; t = 15 min, 50%; t = 17 min, 100% B; t = 19 min, 100% B; t = 22 min, 5%, t = 26 min, 5%, stop. RGDC-thiosuccinimide-DBCO.TFA$^-$ was collected at RT 14.5 min and freeze-dried to obtain a white solid (2.043 mg, 1.651 μmol, 46 % yield).

LC-MS: calculated: 1146.5 $[M+Na]^+$, 1124.5 $[M+H]^+$, 573.7 $[M+H+Na]^{2+}$, 562.7 $[M+2H]^{2+}$, 375.5 $[M+3H]^{3+}$; found: 1146.6 $[M+Na]^+$, 1124.6 $[M+H]^+$, 574.0 $[M+H+Na]^{2+}$, 563.1 $[M+2H]^{2+}$, 375.6 $[M+3H]^{3+}$

ESI-HRMS Calculated for $C_{51}H_{70}N_{11}O_{16}S^+$ $[M+H]^+$: 1124.4717, found 1124.4720; calculated for $C_{51}H_{71}N_{11}O_{16}S^{2+}$ $[M+2H]^{2+}$ 562.7395, found 562.7421.

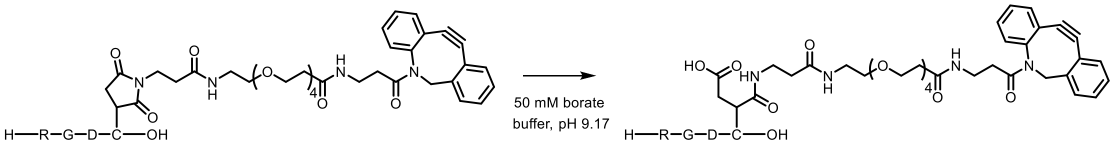

Then, a solution of RGDC-thiosuccinimide-DBCO.TFA$^-$ (1.561 mg, 1.262 μmol) in 50 mM borate buffer pH 9.17 (1.561 mL) was incubated overnight at 37 °C for the succinimide ring opening to the correspondent succinic acid thioether. The stable RGDC-DBSO conjugate was purified by semi-preparative HPLC, as previously described, to afford RGDC-DBCO.TFA$^-$ collected at RT 14.4 min and freeze-dried to obtain a white solid (1.322 mg, 1.053 μmol, 83 % yield).

LC-MS: calculated: 1164.5 $[M+Na]^+$, 1142.5 $[M+H]^+$, 571.7 $[M+2H]^{2+}$, 582.7 $[M+2H]^{2+}$, 381.5 $[M+3H]^{3+}$; found: 1164.7 $[M+Na]^+$, 1142.6 $[M+H]^+$, 572.1 $[M+2H]^{2+}$, 583.1 $[M+H+Na]^{2+}$, 381.7 $[M+3H]^{3+}$

ESI-HRMS calculated for $C_{51}H_{72}N_{11}O_{17}S^+$ $[M+H]^+$: 1142.4823, found 1142.4822; calculated for $C_{51}H_{73}N_{11}O_{17}S^{2+}$ $[M+2H]^{2+}$ 571.7448, found 571.7480.

2.8 Synthesis of PC8 maleimide conjugate

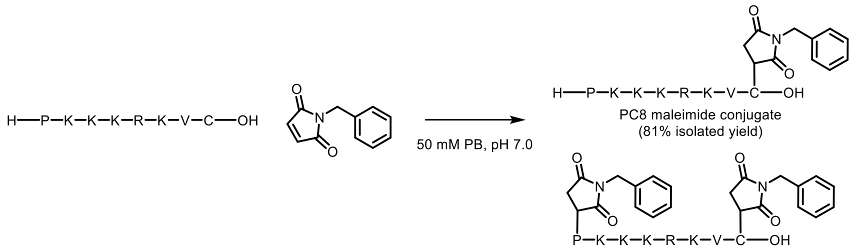

To a fresh solution of PC8 peptide (1 mg/mL in 50 mM PB pH 7.0) (500 µL, 299 nmol) was added benzylmaleimide (10 mM in ACN) (60.9 µL, 608 nmol). After 4h, the desired conjugate was purified by semi-preparative HPLC from the crude reaction mixtures with using a Zorbax Eclipse XDB-C18 HPLC column (80 Å, 9.4 × 250 mm, 5 µm) at a flow rate of 4 mL/min with acetonitrile (solvent A, containing 0.1% *v/v* TFA) and Milli-Q water (solvent B, containing 0.1% *v/v* TFA). Mobile phase t = 0 min, 5% B; t = 21 min, 100%; t = 23 min, 100% B; t = 25 min, 5% B; t = 26 min, 5%, stop. PC8 Maleimide was collected at RT 9.85 min (448 µg, 241 nmol, 81% yield).

LC-MS: calculated: 1195.7 $[M+Na]^+$, 1173.7 $[M+H]^+$; found: 1196.0 $[M+Na]^+$,1173.9 $[M+H]^+$.

ESI-HRMS Calculated for $C_{54}H_{93}N_{16}O_{11}S^+$ $[M+H]^+$: 1173.6925, found 1173.6970; calculated for $C_{54}H_{94}N_{16}O_{11}S^{2+}$ $[M+2H]^{2+}$: 587.3499, found 587.3499; calculated for $C_{54}H_{95}N_{16}O_{11}S^{3+}$ $[M+3H]^{3+}$: 391.9024, found 391.9006.

3 CLog P (n-octanol to water) prediction of *N*-alkylpyridinium derivatives based on ChemDraw

Partition coefficient (*P*) refers to the ratio of a compound in n-octanol to its concentration in water. Since *P* value usually ranges over many orders of magnitude, logarithmic form, such as log*P*, is often used in the literature to determine the hydrophobicity of a certain molecule. Here, we summarized the calculated logP values (CLog *P*) of the *N*-alkylpyridinium derivatives with using ChemDraw software to roughly estimate the hydrophilicity of these compounds compared to other dual modification reagents developed in the literature, for example, the allyl sulfone reagents developed by our group[1] and the azabicyclic vinyl sulfones developed by Bernardes group[2] and the dibromopyridazinedones by Chudasama group.[3] As shown below, the *N*-alkylpyridinium derivatives developed in this paper have lower CLog *P* values than the corresponding allyl sulfones and azabicyclic vinyl sulfones, thus indicating higher hydrophilicity. Higher hydrophilicity of the conjugation reagent is considered as a beneficial aspect to facilitate the subsequent modification reactions since less organic solvent is required to eliminate the risk of protein degradation or aggregation.

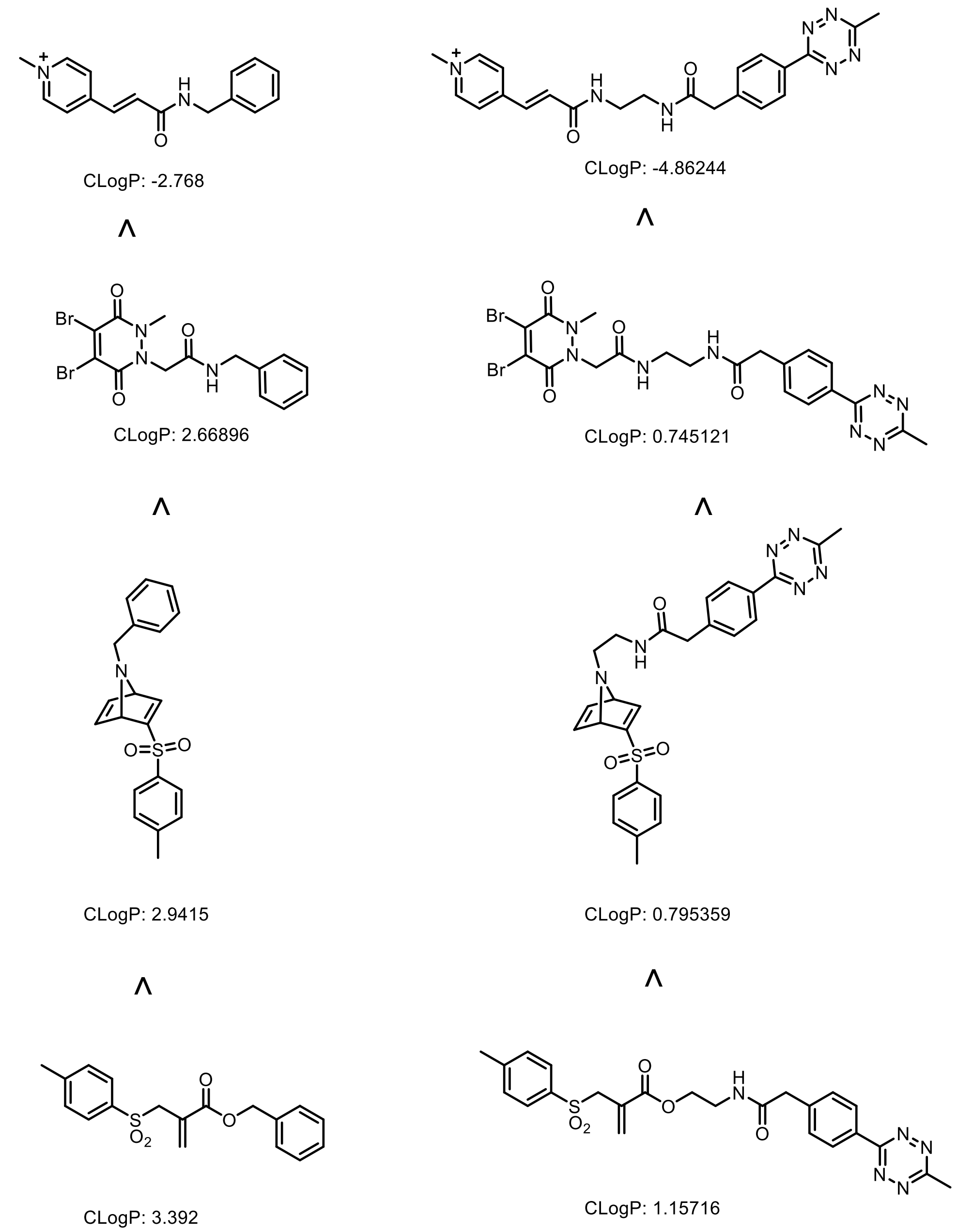

Supplementary Fig. 1: The CLog*P* values of different dual modification reagents predicted by ChemDraw 19.0.

4 Evaluation of the chemoselectivity and the reaction kinetics of the *N*-alkylpyridium derivatives

4.1 Chemoselectivity check of *N*-alkylpyridinium derivatives towards thiol and amino groups

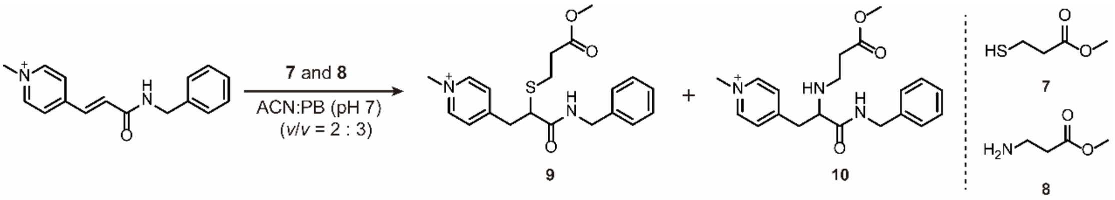

Supplementary Fig. 2: Reactions between **1** with **7** and **8**.

To investigate the reactivity of *N*-alkylpyridinium derivatives towards thiol and amino groups, **1** was chosen as the model compound to react with compounds **7** and **8**. Firstly, compound **1** (50 µg, 0.14 µmol, 1 equiv) and compound **7** (35.6 µg, 0.30 µmol, 2.1 equiv) were dissolved in 50 µL ACN:PB (50 mM, pH 7) (*v/v* = 2:3) mixture. The resultant mixture was incubated at rt for 4h. Then the mixture was injected into the HPLC to monitor the reaction. The HPLC data indicated that there is only one product formed in the mixture as shown in Fig. 3d in the main text. The LC-MS of the formed product **9** is shown below.

Similarly, compound **1** (50 µg, 0.14 µmol, 1 equiv) was also incubated with compound **7** (35.6 µg, 0.30 µmol, 2.1 equiv) and **8** (41.4 µg, 0.30 µmol, 2.1 equiv) in 50 µL ACN:PB (50 mM, pH 7) (*v/v* = 2:3) mixture. The resultant mixture was incubated at rt for 4h before injecting to the HPLC. HPLC data indicated that only product **9** was formed in the reaction without the observation of other products corresponding to the reaction between compounds **1** with **8**.

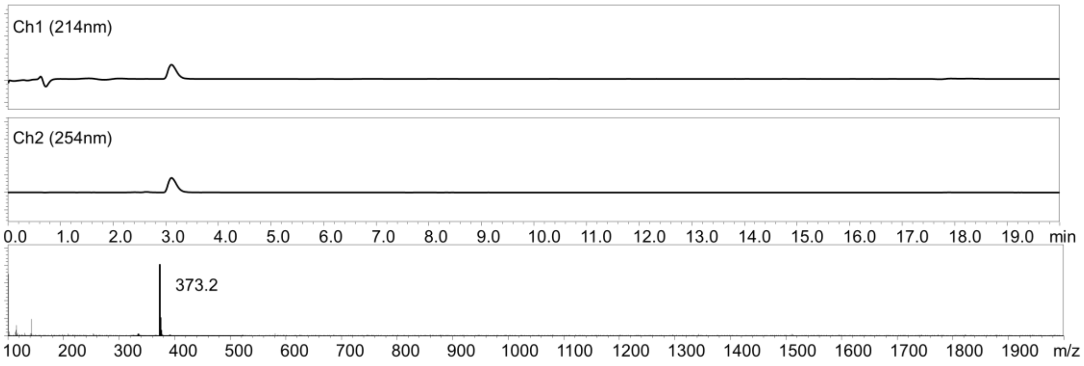

Supplementary Fig. 3: LC-MS of compound **9** (calculated: 373.5 $[M]^+$, found: 373.2 $[M]^+$).

4.2 Evaluation of the reactivity of *N*-alkylpyridinium derivatives towards different amino acids

Compound **1** (50 µg, 0.14 µmol, 1 equiv) was incubated with tyrosine (71.6 µg, 0.40 µmol, 2.8 equiv), methionine (59 µg, 0.40 µmol, 2.8 equiv), arginine (69 µg, 0.40 µmol, 2.8 equiv), histidine (61.3 µg, 0.40 µmol, 2.8 equiv), tryptophan (80.7 µg, 0.40 µmol, 2.8 equiv) and aspartic acid (53 µg, 0.40 µmol, 2.8 equiv), separately. All the reaction mixtures were incubated at rt for 4h. Thereafter, 10 µL of mixture was injected into HPLC to check if compound **1** reacted with these amino acids or not. The HPLC data shown below in Supplementary Fig. 4 indicate that compound **1** exclusively reacted with cysteine and not with any other amino acids listed below.

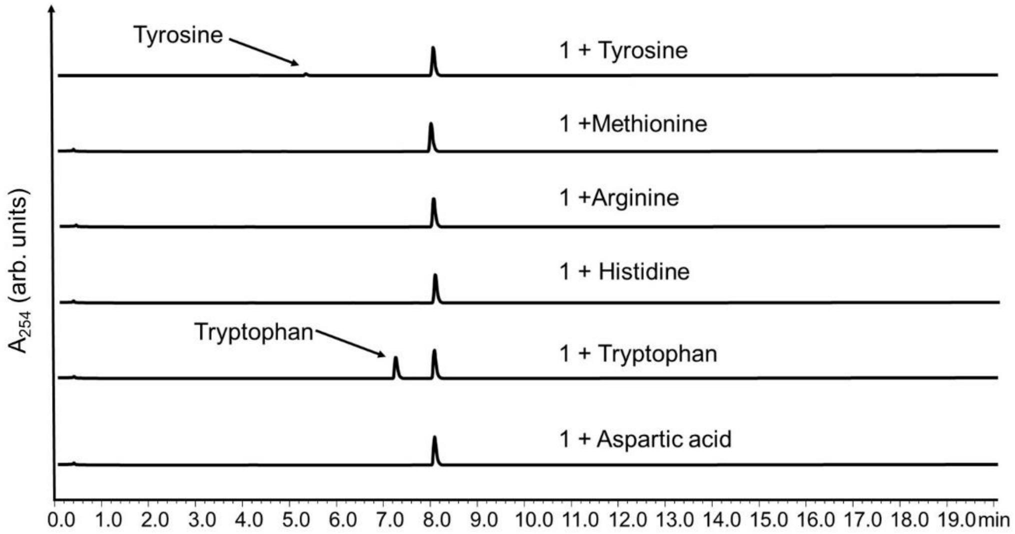

Supplementary Fig. 4: HPLC data of the reaction between compound **1** and different amino acids containing nucleophilic side chains.

4.3 Reactivity check at different pH

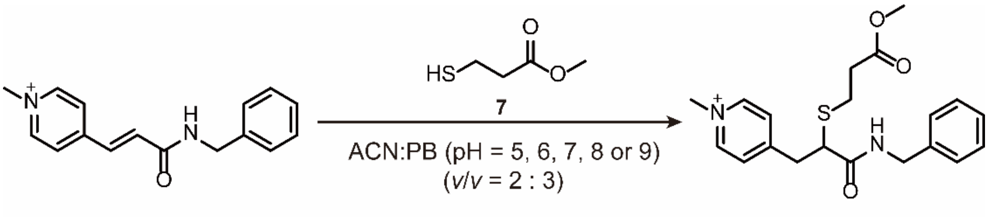

Supplementary Fig. 5: Compound 1 reacting with 7 under different pH.

In order to check the reactivity between *N*-alkylpyridinium derivatives and thiol-containing substrates at different pH, compound **1** was selected and reacted with compound **7** under five different pH (pH = 5, 6, 7, 8 and 9). Specifically, compound **1** (50 µg, 0.14 µmol, 1 equiv) was incubated with compound **7** (32.7 µg, 0.40 µmol, 2 equiv) in ACN:PB (50 mM, pH 5, 6, 7, 8 or 9) (*v/v* = 2:3). Fmoc-Phe-OH was also added as internal standard. After 4h, 10 µL of the reaction mixture was injected into HPLC to monitor the progress of the reaction. The data below indicate quantitative conversion under pH 6, 7, 8 and 9. Only at pH 5, the conversion dropped to around 80% due to the decreased amount of thiolate anion in acidic conditions.

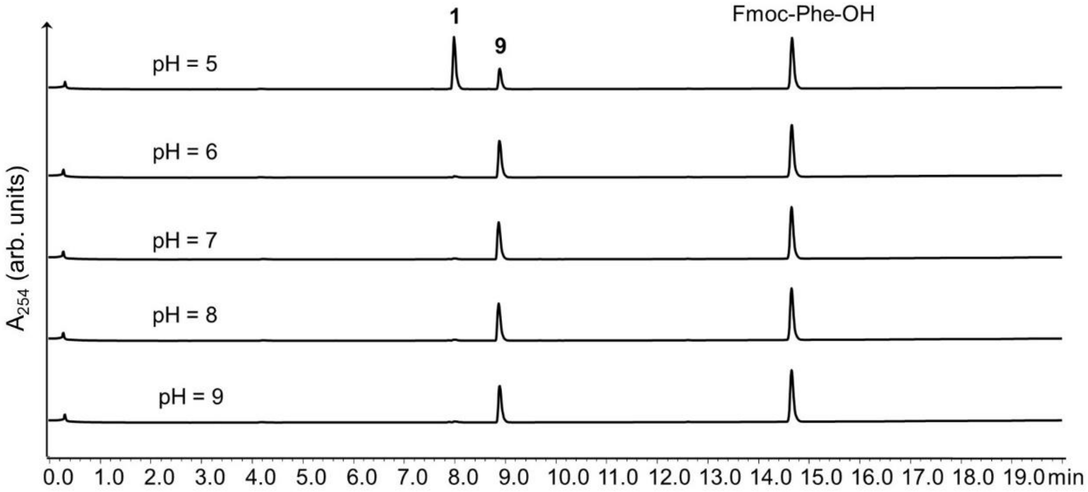

Supplementary Fig. 6: HPLC data of the reaction between compounds **1** and **7** at different pH.

4.4 Kinetics study of compound 1 with compound 7

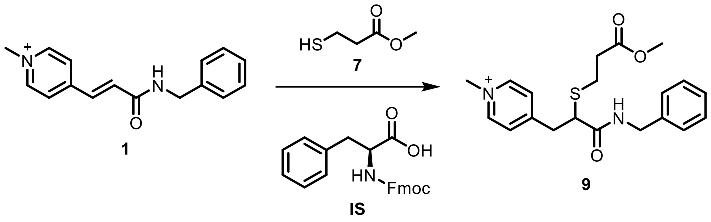

Supplementary Fig. 7: Reaction scheme between compound **1** and compound **7** with Fmoc-Phe-OH as internal standard.

For the kinetics study, compound **1** (1 mM) was mixed with compound **7** (2 mM) together followed by the addition of internal standard Fmoc-Phe-OH (0.1 mM) in ACN:PB (50 mM, pH 7). At different time intervals, 10 µL of the reaction solution was withdrawn and quenched by the 200 µL of methanol containing 10 µL of trifluoroacetic acid. After quenching the reaction, the mixture was frozen in liquid nitrogen before injecting to the HPLC. HPLC data showed that the peak belonging to compound **1** gradually decreased, while the peak belonging to compound **9** increased. The conversions were determined by the integration of the peak area of compounds **1** and **9** in comparison to the internal standard Fmoc-Phe-OH.

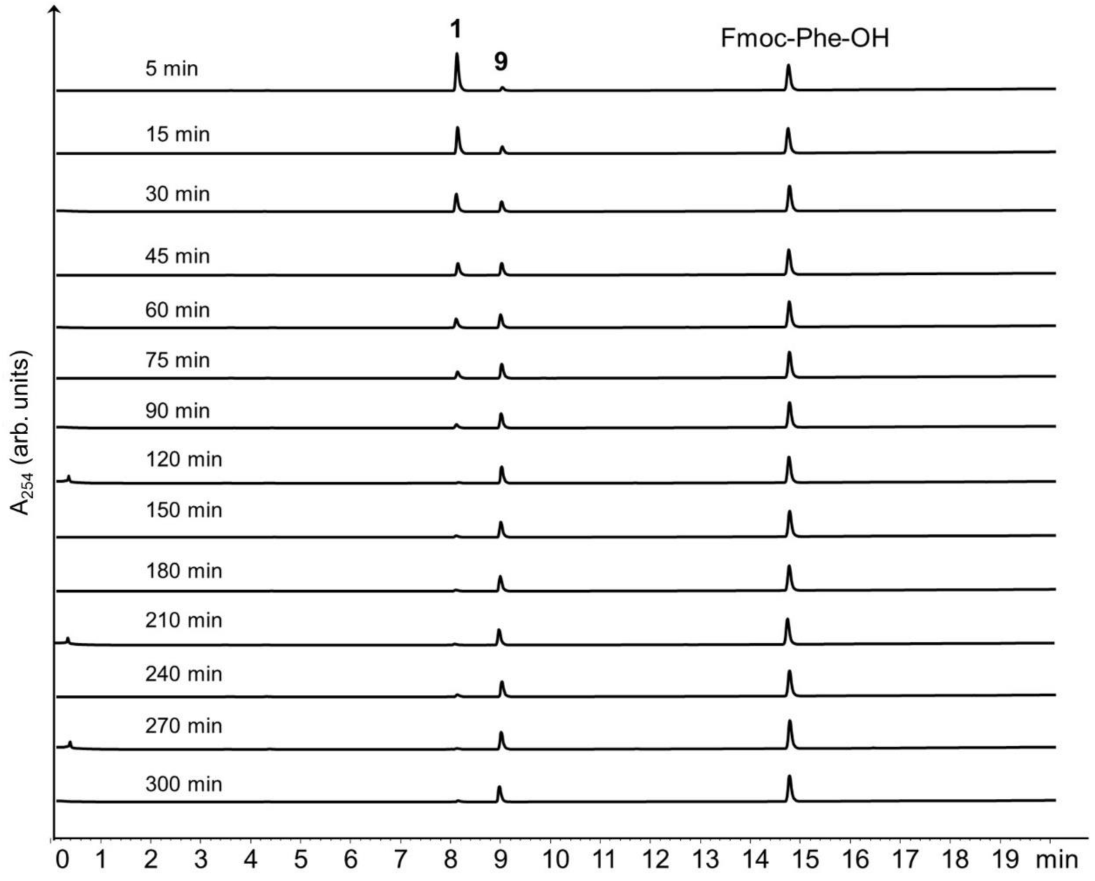

Supplementary Fig. 8: Reaction progress monitored by HPLC between compound **1** and **7**.

5 Stability study of the *N*-alkylpyridinium derivatives

Compound **1** was selected as the model compound to check its stability. Specifically, compound **1** was dissolved in 50 mM PB buffer at three different pH (pH 6, 7 or 8) with a concentration of 1 mg/mL. Fmoc-Phe-OH (0.1 mg/mL) was also added as an internal standard. The resultant solutions at three different pH were incubated for 24h. 10 μL of each solution was injected into HPLC to evaluate their stability.

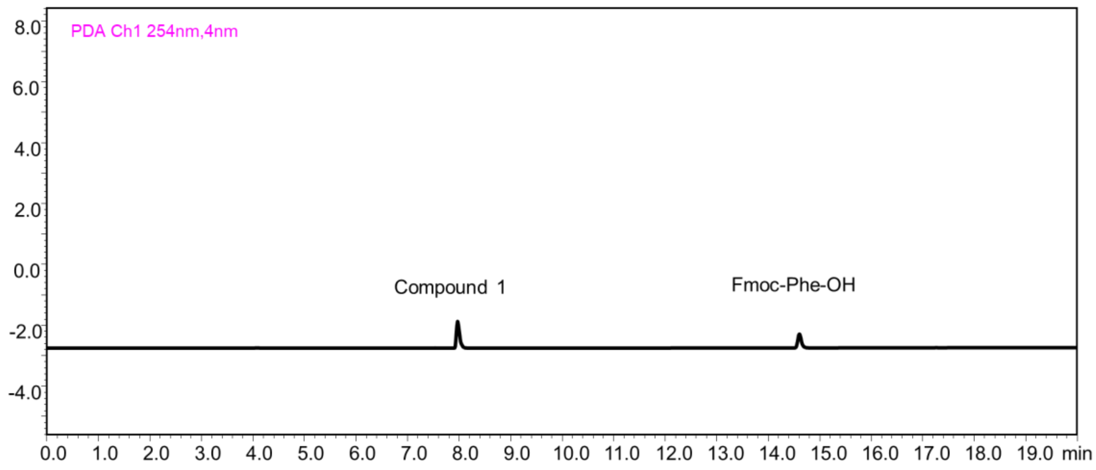

Supplementary Fig. 9: Stability of compound **1** at pH 6 for 24h.

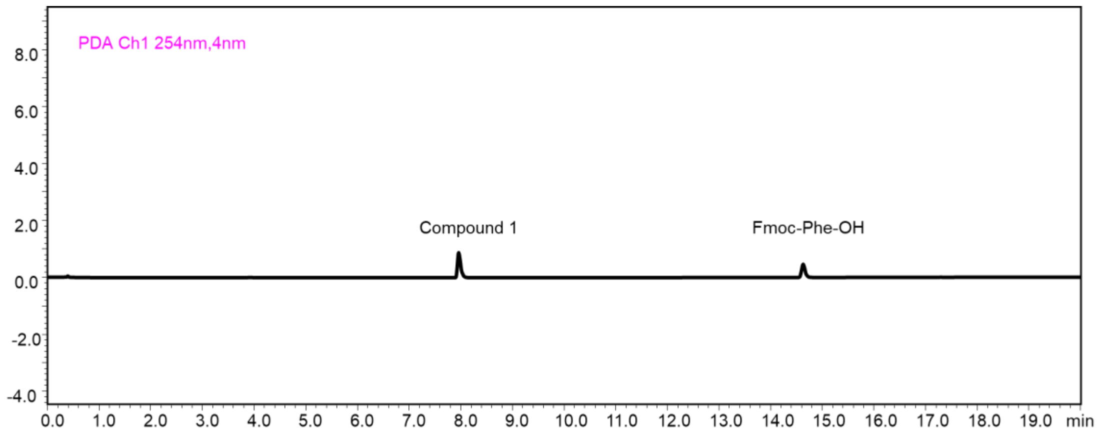

Supplementary Fig. 10: Stability of compound **1** at pH 7 for 24h.

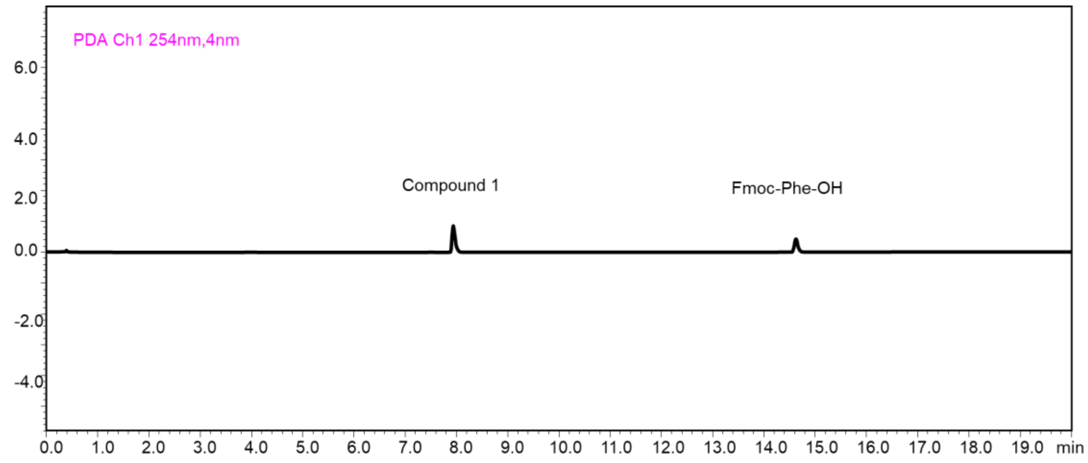

Supplementary Fig. 11 Stability of compound **1** at pH 8 for 24h.

6 NMR characterization of product 9

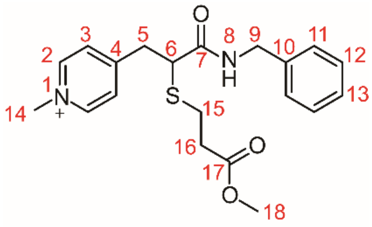

Supplementary Fig. 12: Chemical structure of product **9**.

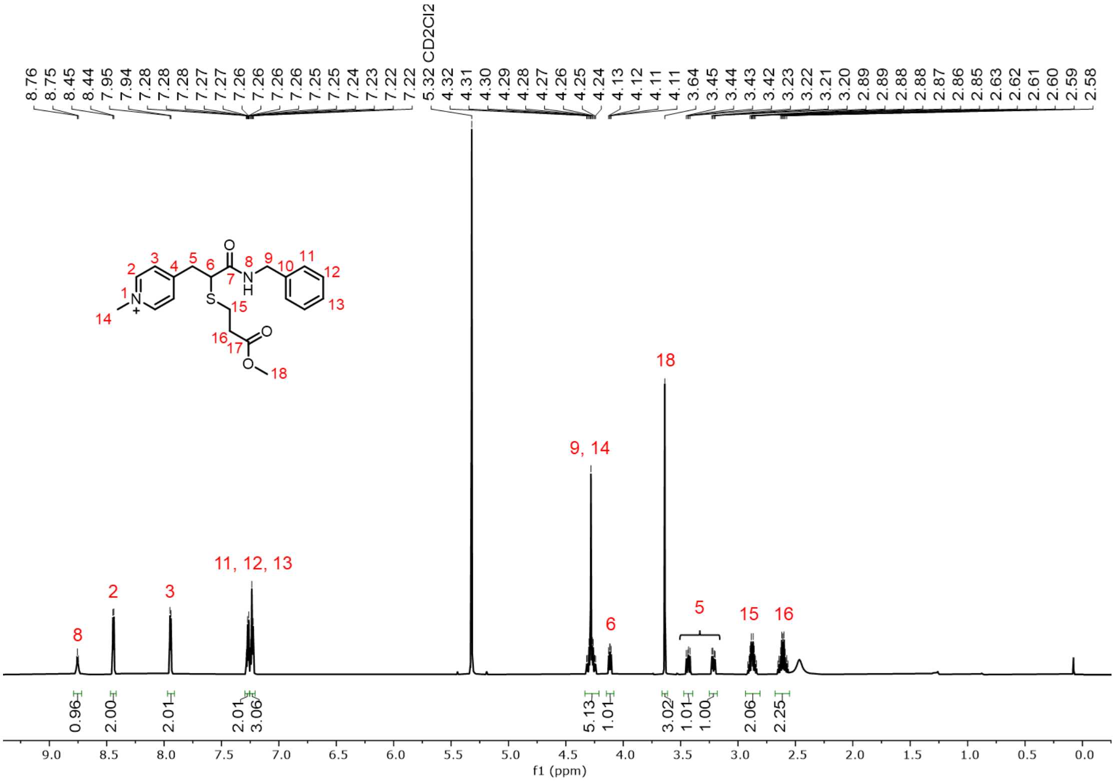

Supplementary Fig. 13: ^{1}H NMR spectrum (500 MHz, CD_2Cl_2, 298 K) of compound **9**.

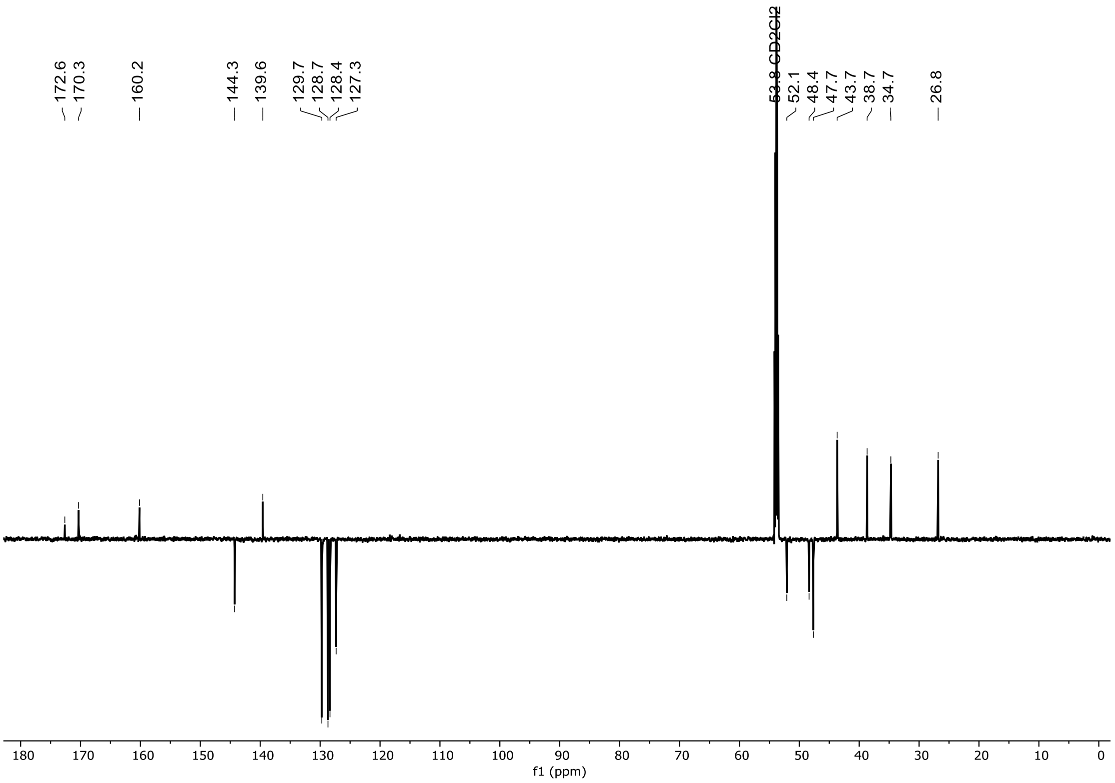

Supplementary Fig. 14: APT spectrum (213 MHz, CD_2Cl_2, 298 K) of compound **9**.

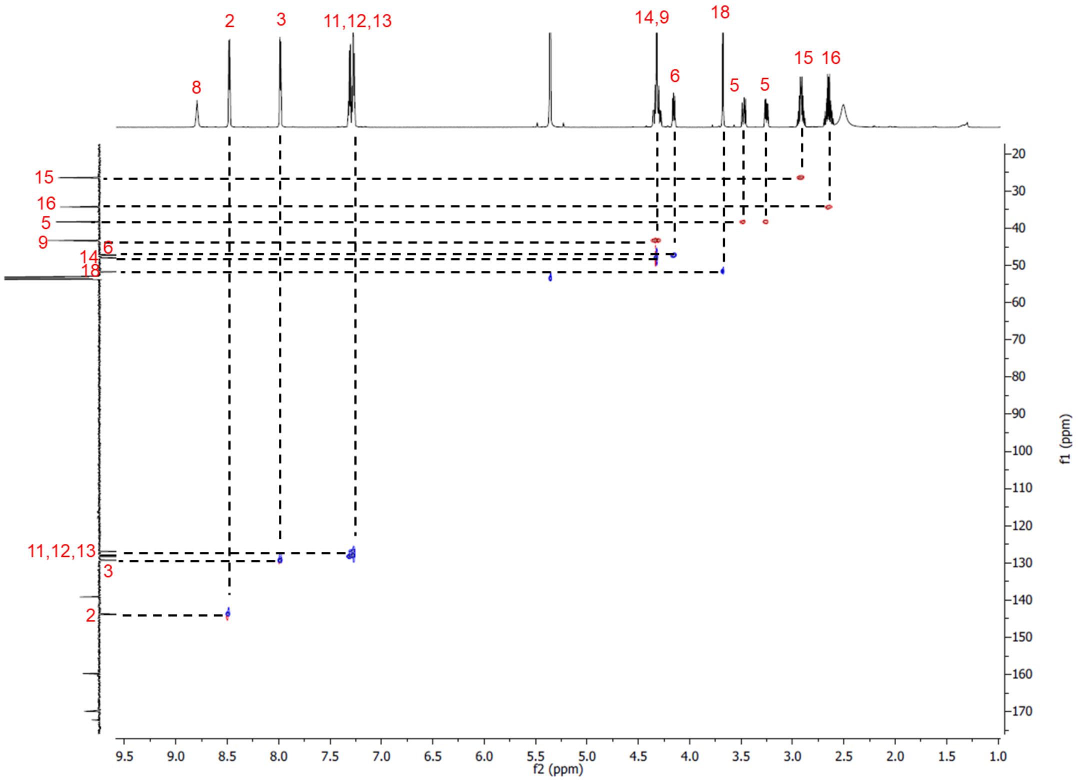

Supplementary Fig. 15: HSQC (500 MHz, CD_2Cl_2, 298 K) of compound **9**.

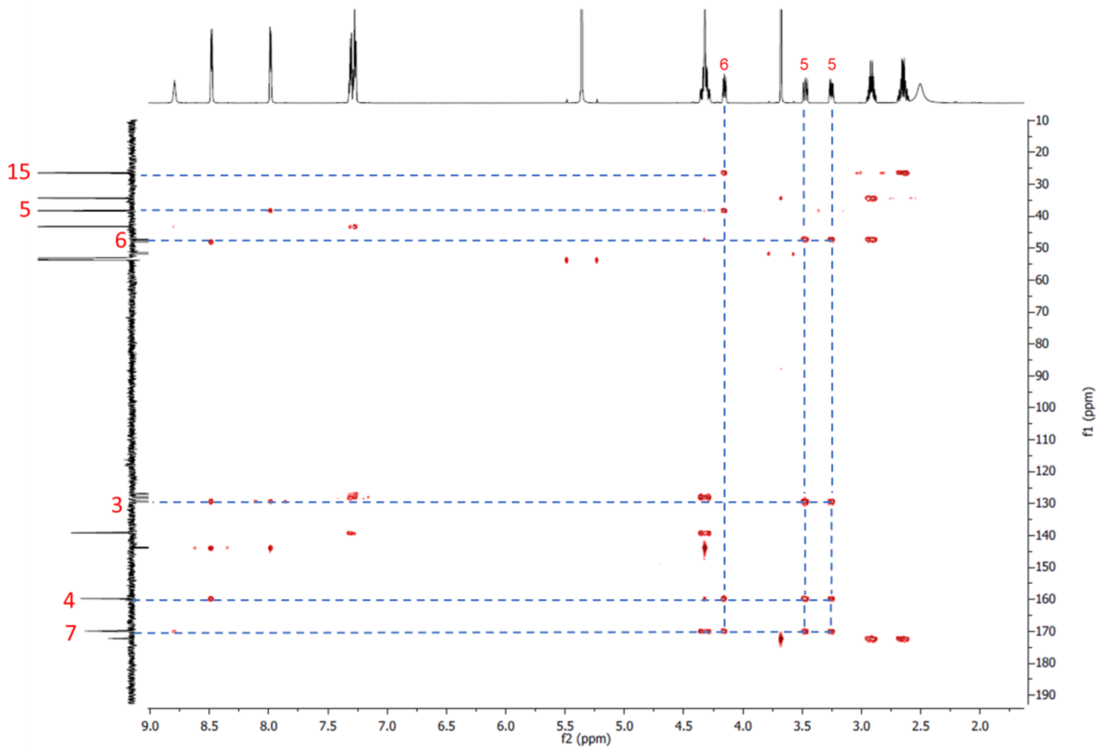

Supplementary Fig. 16: HMBC (500MHz, CD_2Cl_2, 298 K) of compound **9**.

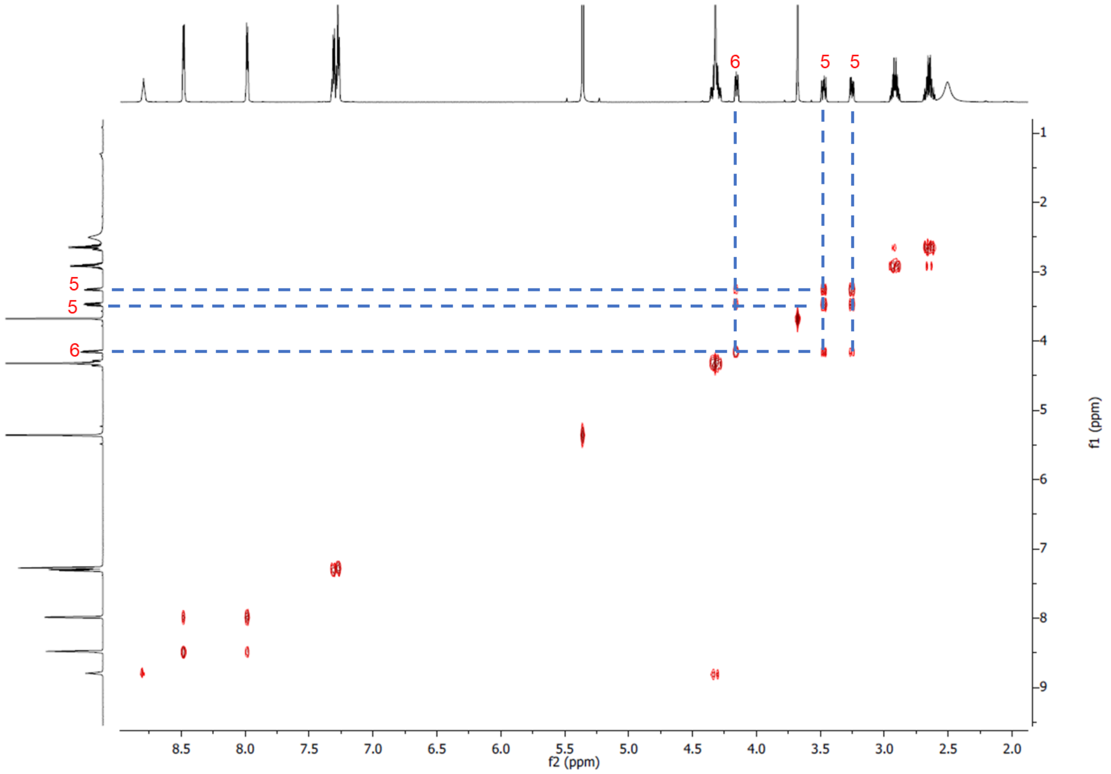

Supplementary Fig. 17: H, H COSY (500MHz, CD_2Cl_2, 298 K) of compound **9**.

7 Computational Methods

Density functional theory (DFT) calculations were performed using the Gaussian 16 software package[4] and structural representations were generated with *CYLview*.[5] For the computation of the energy profiles, all the geometry optimizations were carried out using the hybrid meta-GGA functional M06-2X developed by Truhlar and co-workers[6] and the valence double-zeta 6-31+G(d,p) basis set, with solvent effects (water) calculated by means of the Polarizable Continuum Model (PCM) as devised by Tomasi and co-workers.[7-10] All optimized geometries were characterized by vibrational frequency calculations at the same level of theory as either minima (zero imaginary frequencies) or saddle points (single imaginary frequency) on the potential energy surface. The free energy values presented along the manuscript and SI were derived from the electronic energy values obtained at the M06-2X/6-31+G(d,p) level, and corrected by using the thermal and entropic corrections based on structural and vibration frequency data calculated at the same level.

Condensed Fukui indices were calculated using total population obtained with NBO analysis at M06-2X/def2-TZVPP/PCM(water)//M06-2X/6-31+G(d,p)/PCM(water) level of theory. Proton affinity analysis was performed at M06-2X/def2-TZVPP//M06-2X/6-31+G(d,p) level of theory. HOMO and LUMO energies were calculated at M06-2X/def2-TZVPP/PCM(water)//M06-2X/6-31+G(d,p) level of theory.

7.1 Computational design of new *N*-alkylpyridinium reagent

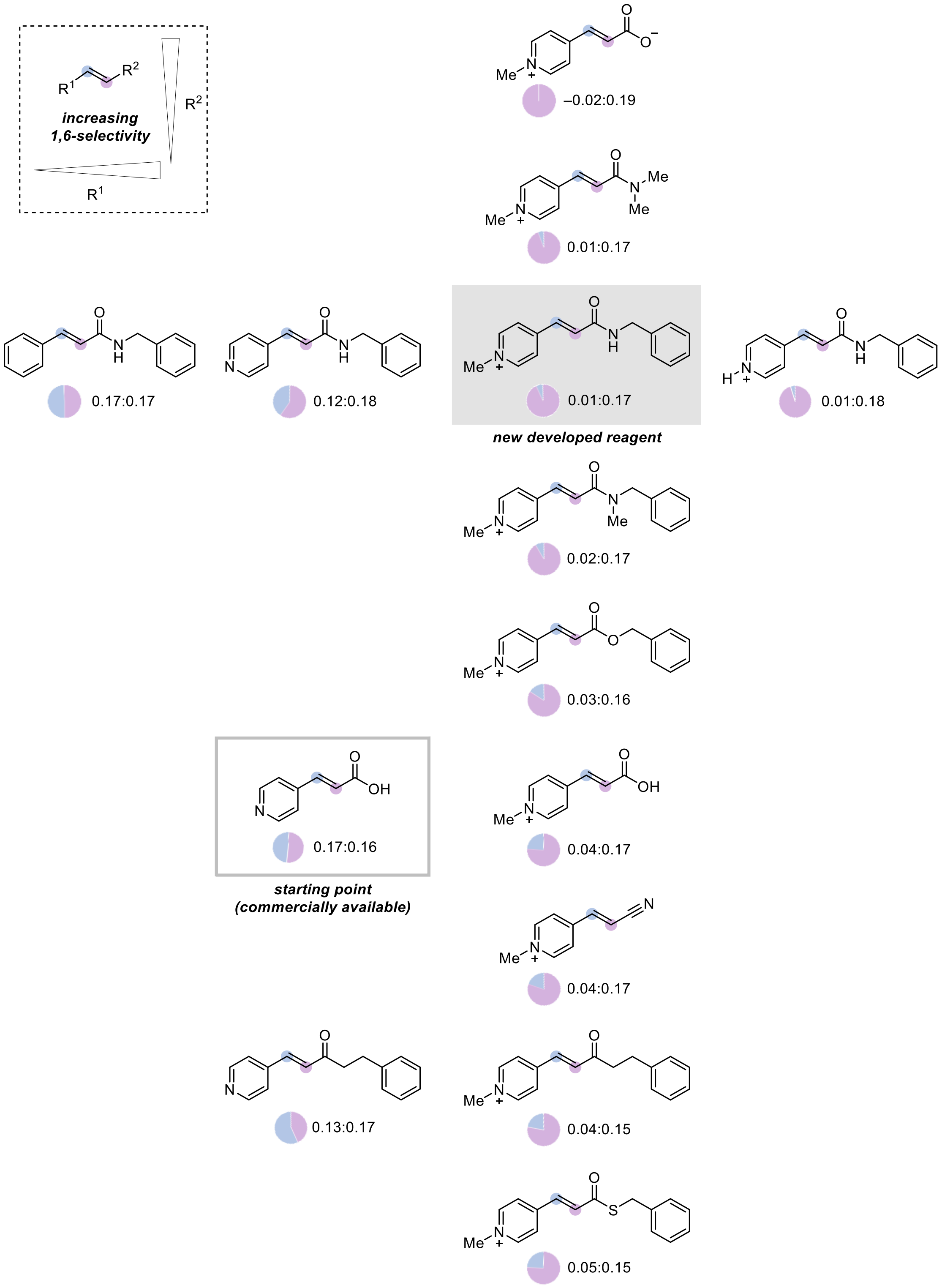

Supplementary Fig. 18: Sorted list of computed structures derived from commercially available (*E*)-3-(pyridin-4-yl)acrylic acid. Fukui indices (f+) of the carbon atoms leading to the formation of the 1,6- and 1,4-addition products, respectively, are shown next to each structure. Fukui function represents the electron density towards electrophilicity: the higher the f+, the more electrophilic the reactive site.

7.2 Frontier molecular orbital (FMO) analysis

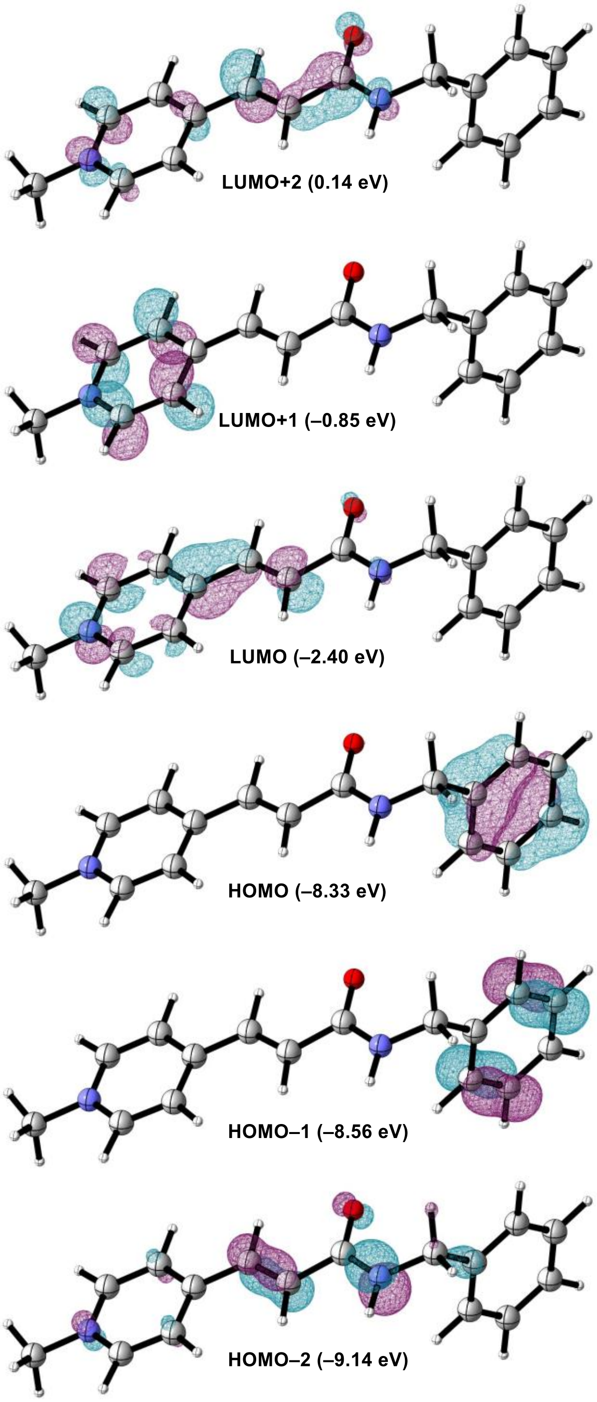

Supplementary Fig. 19: Representations of the HOMO-1, HOMO-2, HOMO, LUMO, LUMO+1 and LUMO+2 of compound **1**. Isosurfaces were generated with a contour value of 0.08 a.u.

7.3 Reaction potential energy surfaces for different acceptors

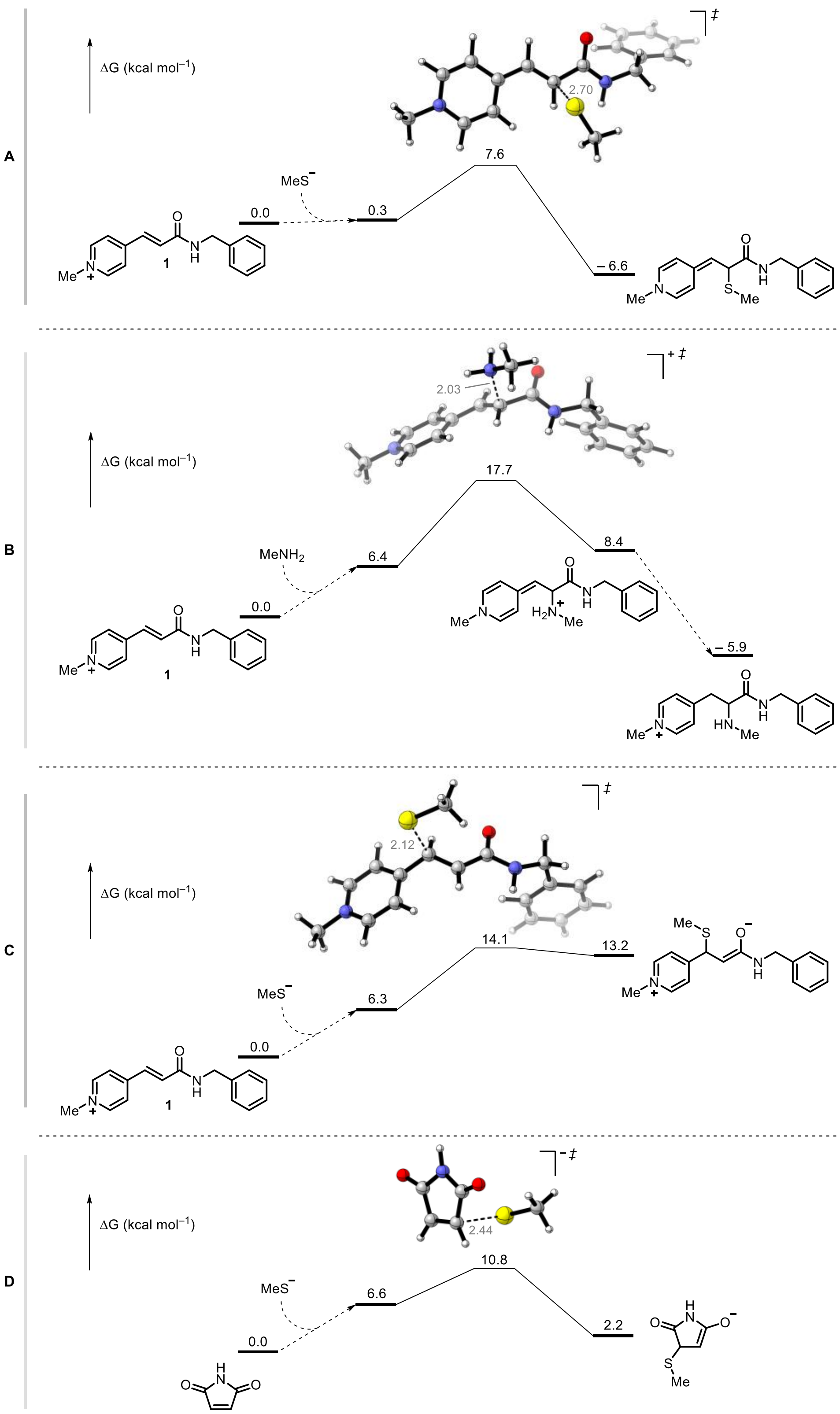

Supplementary Fig. 20: Gibbs free energy profile for **A)** 1,6-addition of methanethiolate anion to compound **1**, **B)** 1,6-addition of methylamine to compound **1**, **C)** 1,4-addition of methanethiolate anion to compound **1**, **D)** 1,4-addition of methanethiolate anion to maleimide (Mal). DFT calculations were performed at the M06-2X/6-31+G(d,p)/PCM(water) level of theory (energy values in kcal mol^{-1}). Transition state geometries depict the S-C bond distance in Å.

7.4 Proton affinity study

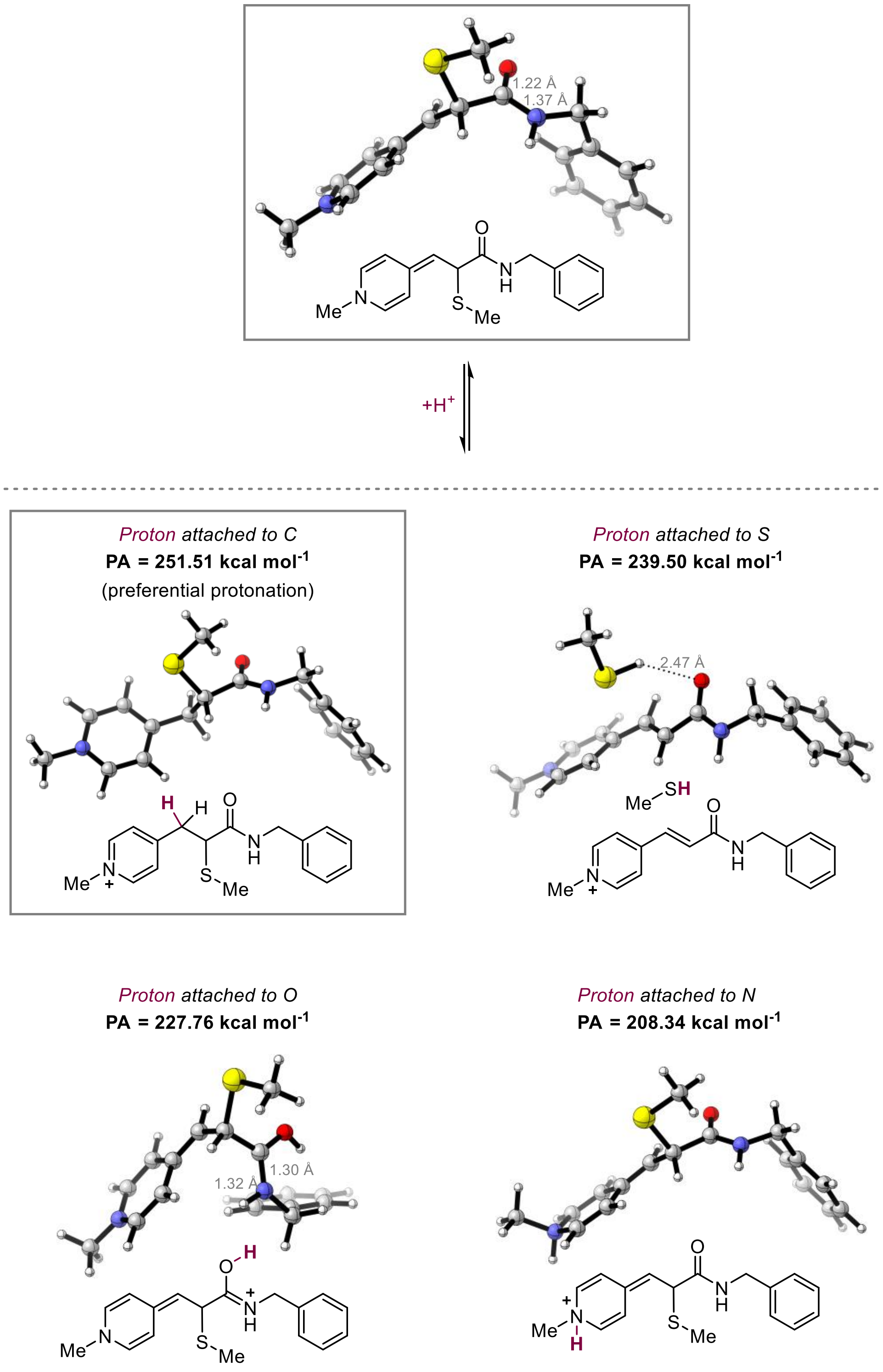

Supplementary Fig. 21: DFT computed proton affinities (PA) for intermediate product obtained from 1,6-addition of methanethiolate anion to compound **1**. Optimized structures of the products resulting from protonation at different atoms are shown.

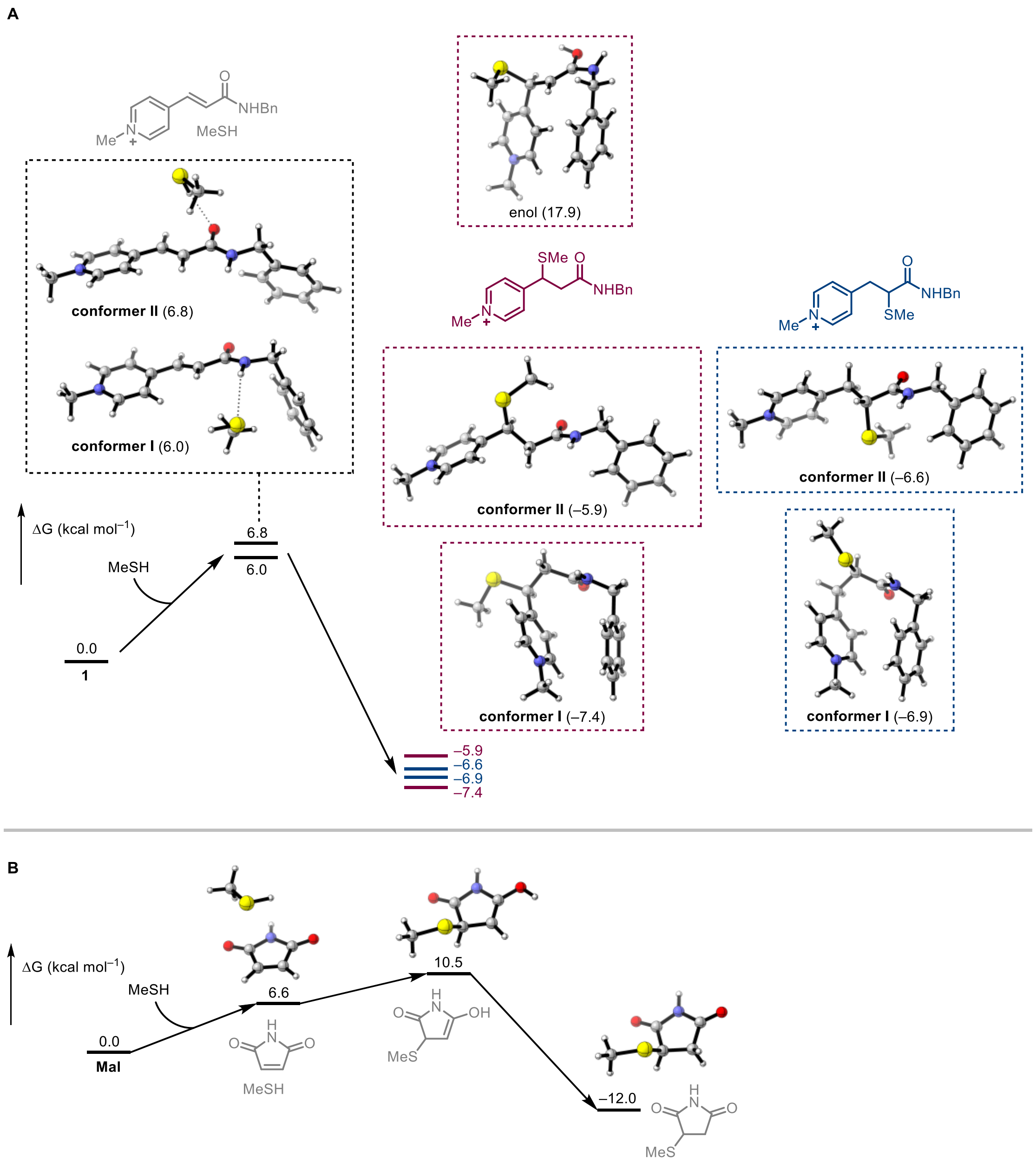

Supplementary Fig. 22: Gibbs free energies and optimized structures of reagents and products for **A)** 1,4- and 1,6-addition of methanethiol to compound **1**, **B)** 1,4-addition of methanethiol to maleimide. DFT calculations were performed at the M06-2X/6-31+G(d,p)/PCM(water) level of theory (energy values in kcal mol^{-1}).

8 Site-selective modification of peptides

8.1 General procedure for modification of peptides with *N*-alkylpyridinium derivatives

To a fresh solution of peptide (1 mg/mL) in 50 mM PB, pH 7 was added 1.2 equiv of compound **1** and mixed at rt. After 4-5 h, the conjugates were isolated by semi-preparative HPLC from the crude reaction mixtures with using a Zorbax Eclipse XDB-C18 HPLC column (80 Å, 9.4 × 250 mm, 5 μm) at a flow rate of 4 mL/min with acetonitrile (solvent A, containing 0.1% *v/v* TFA) and Milli-Q water (solvent B, containing 0.1% *v/v* TFA). **Method A:** mobile phase t = 0 min, 5% B; t = 15 min, 50%; t = 17 min, 100% B; t = 19 min, 100% B; t = 22 min, 5%, t = 26 min, 5%, stop. **Method B:** mobile phase t = 0 min, 5% B; t = 21 min, 100%; t = 23 min, 100% B; t = 25 min, 5% B; t = 26 min, 5%, stop. The modified peptide conjugates were characterized by ESI-LRMS and ESI-HRMS in positive mode as shown below.

8.1.1 PC8 conjugate 11

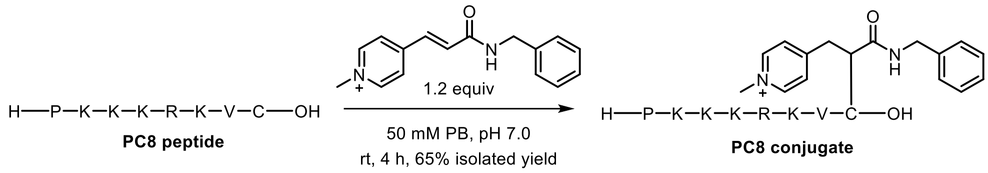

Supplementary Fig. 23: PC8 peptide modification with compound **1** (PC8 conjugate, RT 8.1 min and excess of reagent **1**, RT 10.7 min, method A).

LC-MS: calculated: 1238.8 $[M]^{+}$, 620.7 $[M+H]^{2+}$, 413.6 $[M+2H]^{3+}$, 310.4 $[M+3H]^{4+}$; found: 1239.0 $[M]^{+}$, 620.2 $[M+H]^{2+}$, 413.9 $[M+2H]^{3+}$, 310.6 $[M+3H]^{4+}$.

ESI-HRMS Calculated for $C_{59}H_{101}N_{17}O_{10}S^{2+}$ $[M+H]^{2+}$: 619.8814, found 619.8820; calculated for $C_{59}H_{102}N_{17}O_{10}S^{2+}$ $[M+2H]^{3+}$: 413.5900, found 413.5910; calculated for $C_{59}H_{103}N_{17}O_{10}S^{2+}$ $[M+3H]^{4+}$: 310.4443, found 310.4456.

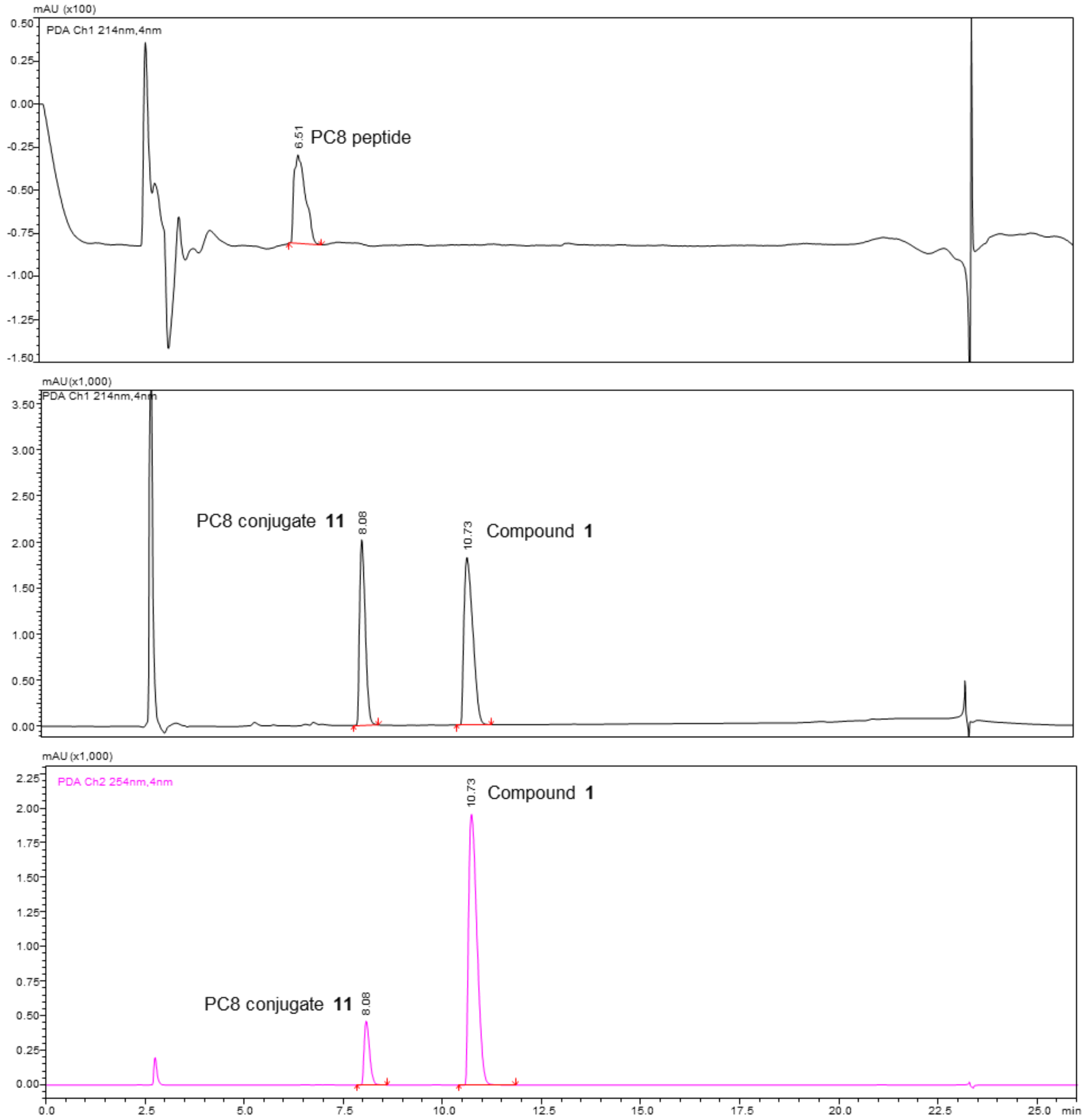

Supplementary Fig. 24: Chromatogram of initial PC8 peptide (214 nm, top) and reaction mixture for PC8 conjugate purification (214 and 254 nm detection, middle and bottom respectively).

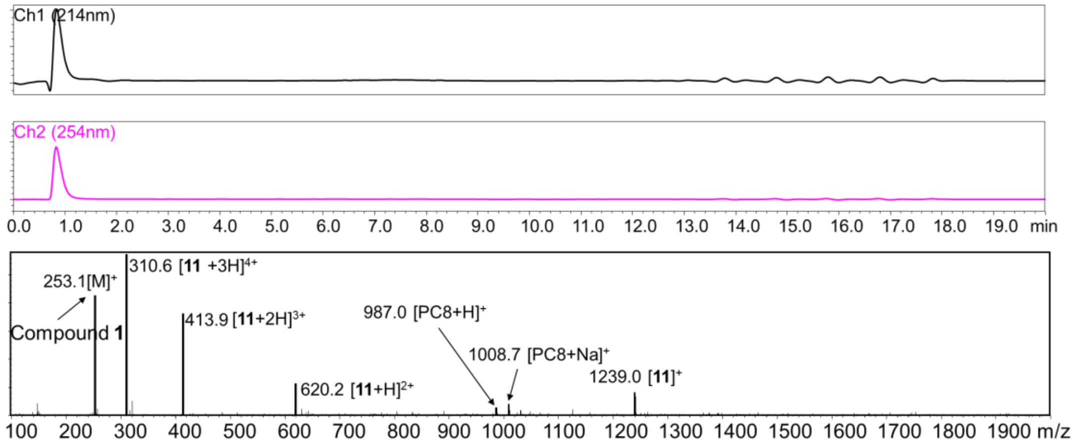

Supplementary Fig. 25: LC-MS analysis of the isolated PC8 conjugate **11** (calculated: 1239.3 $[M]^{+}$, 620.7 $[M+H]^{2+}$, 414.1 $[M+2H]^{3+}$, 310.8 $[M+3H]^{4+}$; 253.1 [Compound **1**]$^{+}$, 987.0 $[PC8+H]^{+}$, 1008.7 $[PC8+Na]^{+}$, found: 1238.9 $[M]^{+}$, 620.2 $[M+H]^{2+}$, 413.8 $[M+2H]^{3+}$, 310.6 $[M+3H]^{4+}$, 253.1 [Compound **1**]$^{+}$, 987.0 $[PC8+H]^{+}$, 1008.7 [PC8+Na]$^{+}$).

8.1.1.1 PC8 peptide HPLC conversion

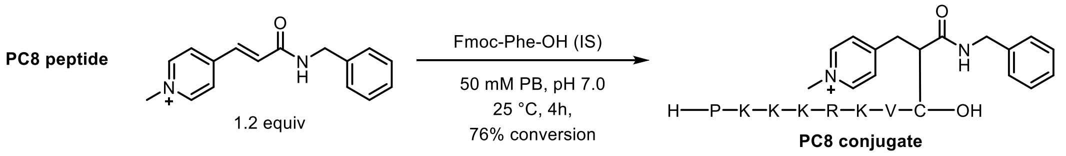

To a solution of PC8 peptide (1.0 mg/mL) (100 µg, 59.9 nmol) in 100 µL of 50 mM PB, pH 7 with Fmoc-Phe-OH as internal standard (63.2 µg/mL), was added compound **1** (50 mM in 50 mM PB, pH 7) (2.43 µL, 122 nmol). The reaction mixture was mixed at 25 °C for 4 h and transferred to an HPLC vial and 20 µL were injected into analytic HPLC (Zorbax Eclipse XDB-C18 column, 4.6 × 250 mm, 5 µm) with a flow rate of 1 mL/min with acetonitrile (solvent A, containing 0.1% *v/v* TFA) and Milli-Q water (solvent B, containing 0.1% *v/v* TFA). Mobile phase t = 0 min, 0% B; t = 2 min, 0%; t = 17 min, 100% B; t = 20 min, 100% B; t = 23 min, 0%, t = 25 min, 0%, stop. The concentration of PC8 peptide (RT 11.9 min) was normalized to the IS (RT 20.1 min) peak areas (AUC) at 214 nm. (Fig S8.4). AUC ratio was converted to concentration through a calibration curve (Fig S8.5).

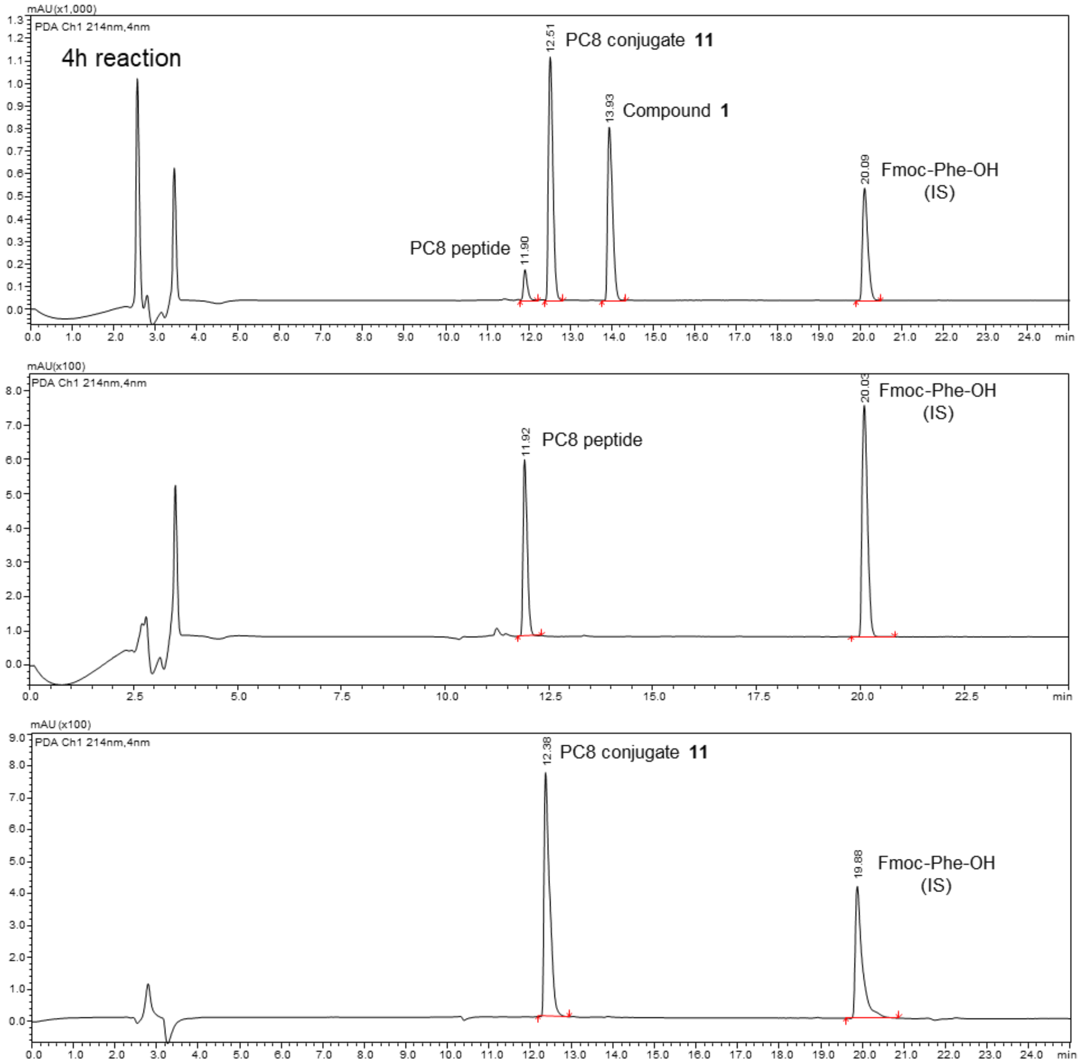

Supplementary Fig. 26: HPLC analysis of the reaction mixture of PC8 with compound **1** after 4h at 25°C (top), isolated PC8 conjugate **11** (middle) and fresh solution of PC8 peptide (bottom) with Fmoc-Phe-OH as internal standard (214 nm detection).

Supplementary Table 1: Calculations for conversion of PC8 peptide to PC8 conjugate **11** after 4h reaction in 50 mM PB pH 7.0 at 25°C.

Reaction time	PC8 area	IS area	AUC ratio	[PC8] (mg/mL)	Conversion (%)
4h	909601	4429057	0.205371	0.237	76

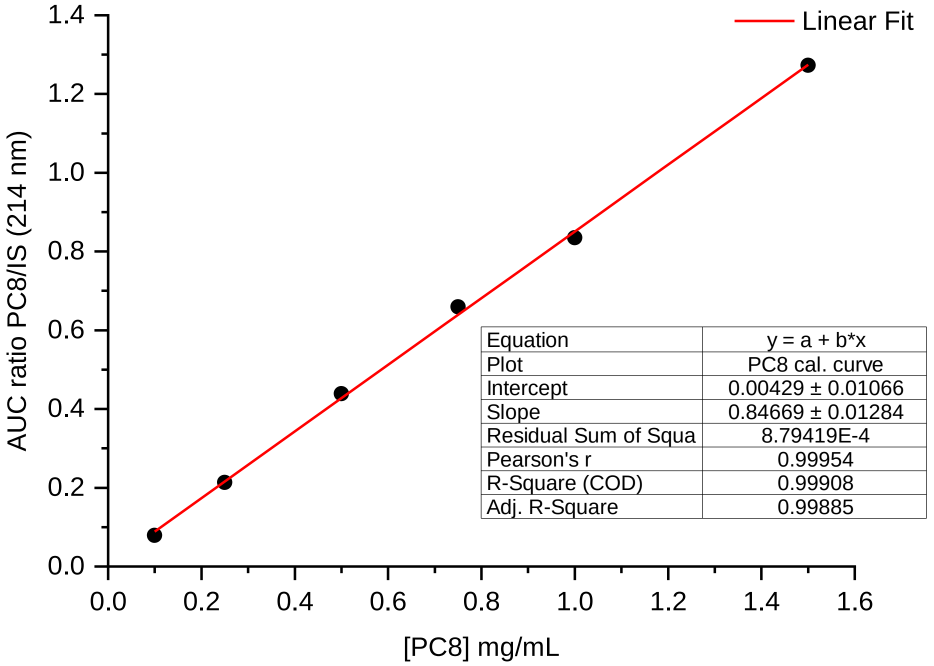

Supplementary Fig. 27: Calibration curve for PC8 concentration in 50 mM PB pH 7.0, with Fmoc-Phe-OH as internal standard (63.2 µg/mL). Source data are provided as a Source Data file.

8.1.2 CEIE conjugate

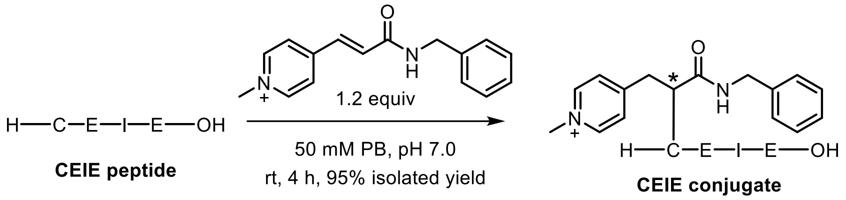

Supplementary Fig. 28: CEIE peptide modification with compound **1** (CEIE conjugate, RT 9.9 and 10.2 min and excess of reagent **1**, RT 10.8 min, method A).

LC-MS calculated: 745.3 $[M]^+$, 373.2 $[M+H]^{2+}$; found: 745.4 $[M]^+$, 373.3 $[M+H]^{2+}$).

ESI-HRMS Calculated for $C_{35}H_{49}N_6O_{10}S^+$ $[M]^+$: 745.3225, found 745.3237; calculated for $C_{35}H_{50}N_6O_{10}S^+$ $[M+H]^{2+}$: 373.1649, found 373.1661.

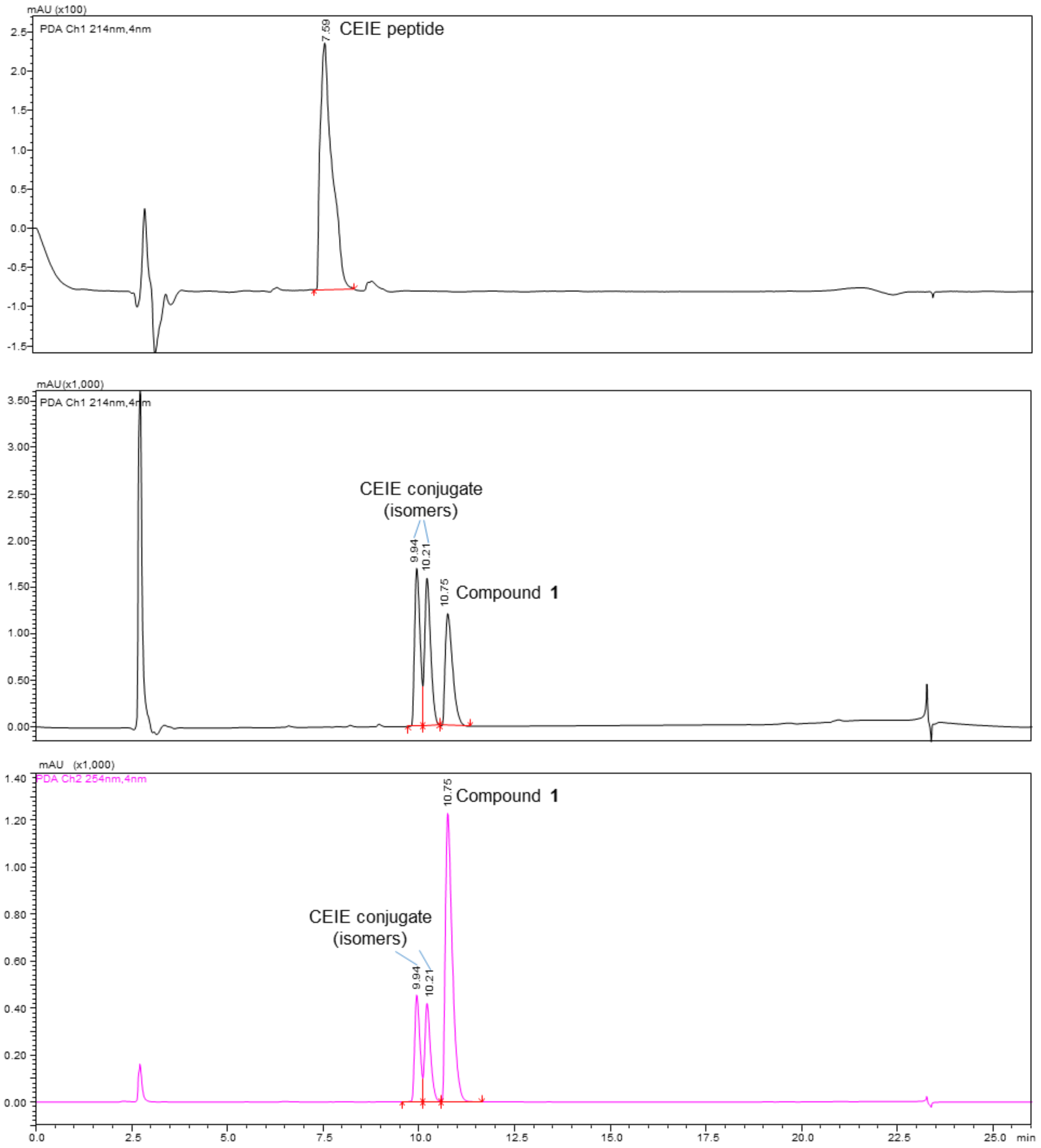

Supplementary Fig. 29: Chromatograms of initial CEIE peptide (214 nm) and reaction mixture for CEIE conjugate purification (214 and 254 nm detection).

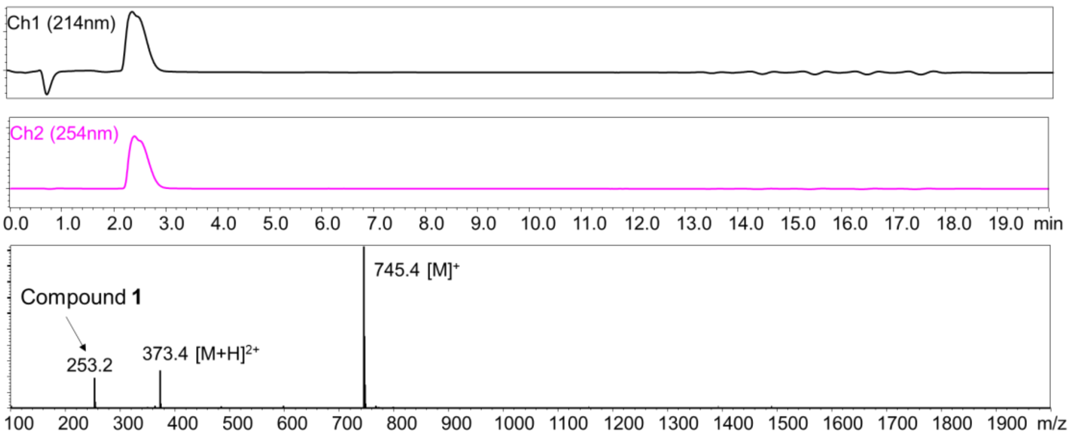

Supplementary Fig. 30: LC-MS analysis of the modified CEIE (calculated: 745.3 $[M]^+$, 373.2 $[M+H]^{2+}$; found: 745.4 $[M]^+$, 373.3 $[M+H]^{2+}$).

8.1.2.1 CEIE peptide modification and analytical HPLC analysis

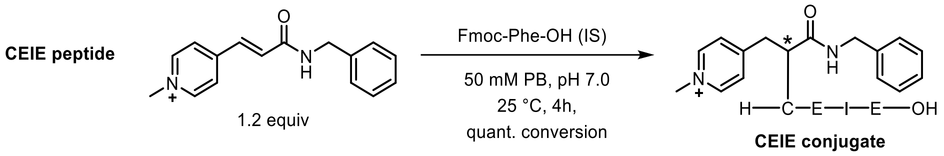

To a fresh solution of CEIE peptide (1.0 mg/mL in 50 mM PB, pH 7) (100 µL, 0.203 µmol) with Fmoc-Phe-OH as internal standard (126.6 µg/mL) was added compound **1** (50 mM in 50 mM PB, pH7) (4.87 µL, 0.244 µmol). The reaction mixture was mixed at 25 °C for 4 h and transferred to an HPLC vial and 20 µL were injected into analytic HPLC (Zorbax Eclipse XDB-C18 column, 4.6 × 250 mm, 5 µm) with a flow rate of 1 mL/min with acetonitrile (solvent A, containing 0.1% *v/v* TFA) and Milli-Q water (solvent B, containing 0.1% *v/v* TFA). Mobile phase t = 0 min, 0% B; t = 2 min, 0%; t = 17 min, 100% B; t = 20 min, 100% B; t = 23 min, 0%, t = 25 min, 0%, stop. The conversion was complete as no starting peptide could be observed in the reaction mixture chromatogram. CEIE peptide (RT 12.4 min), CEIE conjugates (RT 13.3 and 13.4 min), compound **1** (RT 13.9 min) and IS (RT 20.1 min) at 214 nm detection (Fig S8.9).

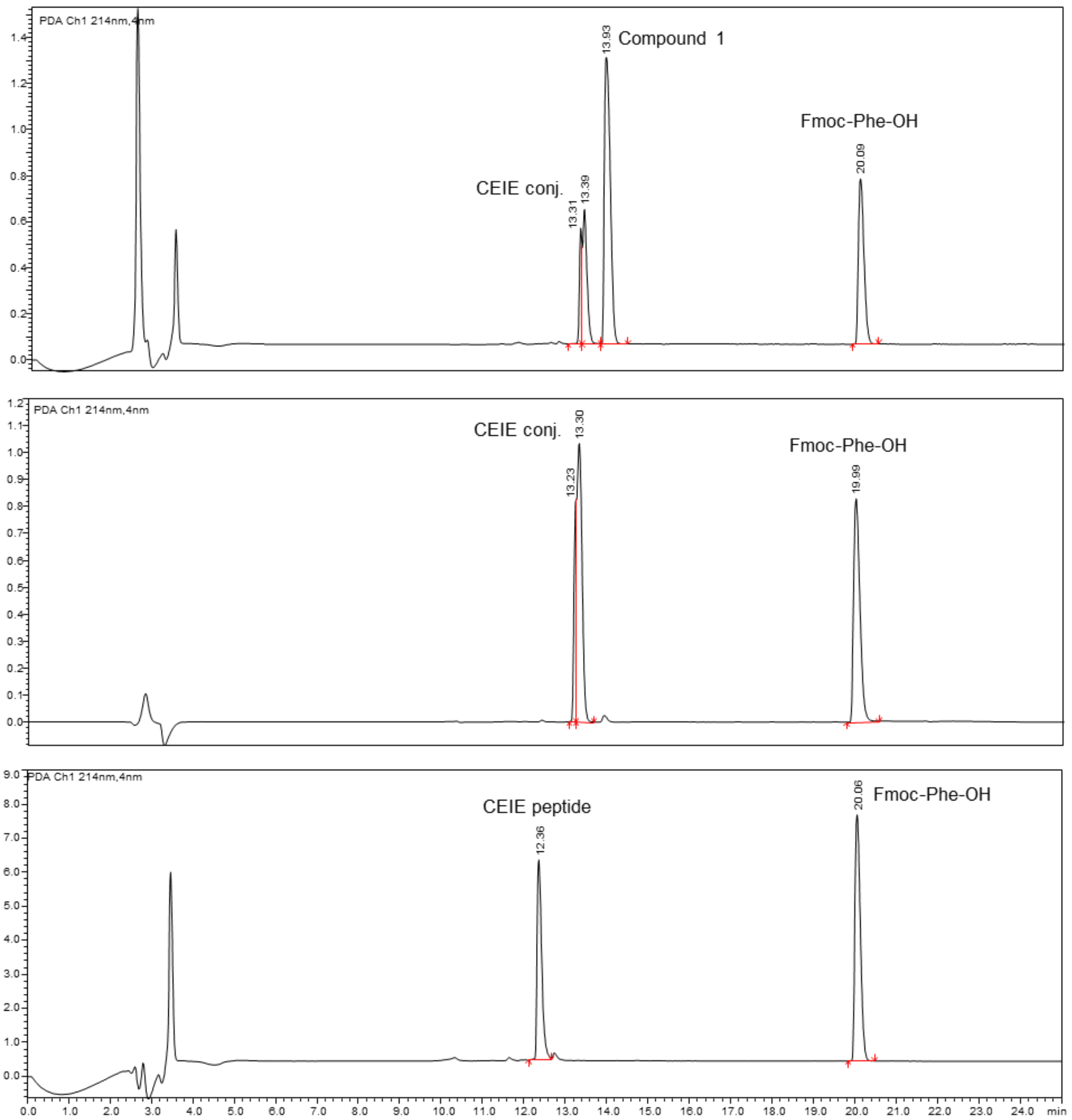

Supplementary Fig. 31: HPLC analysis of the reaction mixture of CEIE with compound **1** after 4h at 25°C (top), isolated CEIE conjugates (middle) and fresh solution of CEIE peptide (bottom) with Fmoc-Phe-OH as internal standard.

8.1.3 WSC02 conjugate

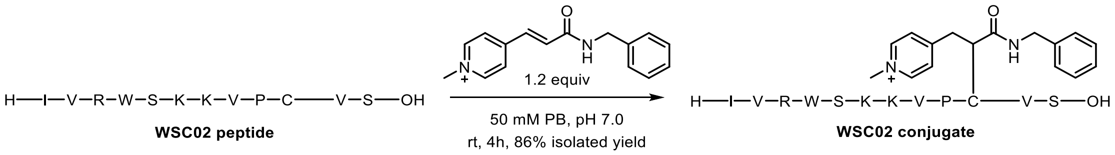

Supplementary Fig. 32: WSC02 peptide modification with compound **1** (WSC02 peptide RT 8.5 min, WSC02 conjugate, RT 9.1 min and excess of reagent **1**, RT 8.87 min, method B).

LC-MS: calculated: 1653.9 $[M]^{+}$, 827.5 $[M+H]^{2+}$, 552.0 $[M+2H]^{3+}$, 414.2 $[M+3H]^{4+}$; found: 1654.9 $[M]^{+}$, 827.9 $[M+2H]^{2+}$, 552.2 $[M+3H]^{3+}$, 414.5 $[M+4H]^{4+}$.

ESI-HRMS Calculated for $C_{80}H_{126}N_{20}O_{16}S^{+}$ $[M+H]^{2+}$: 827.4685, found 827.4698; calculated for $C_{80}H_{127}N_{20}O_{16}S^{+}$ $[M+2H]^{3+}$: 551.9814, found 551.9827; calculated for $C_{80}H_{128}N_{20}O_{16}S^{+}$

$[M+3H]^{4+}$: 414.2379, found 414.2385.

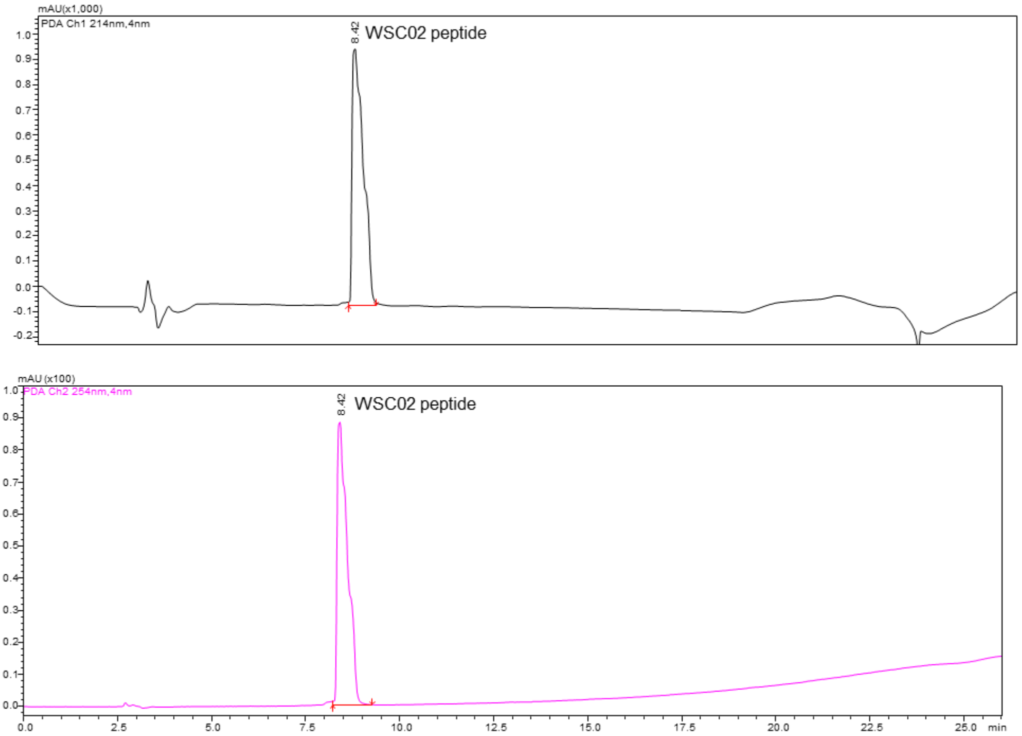

Supplementary Fig. 33: Chromatograms of initial WSC02 peptide (214 and 254 nm detection).

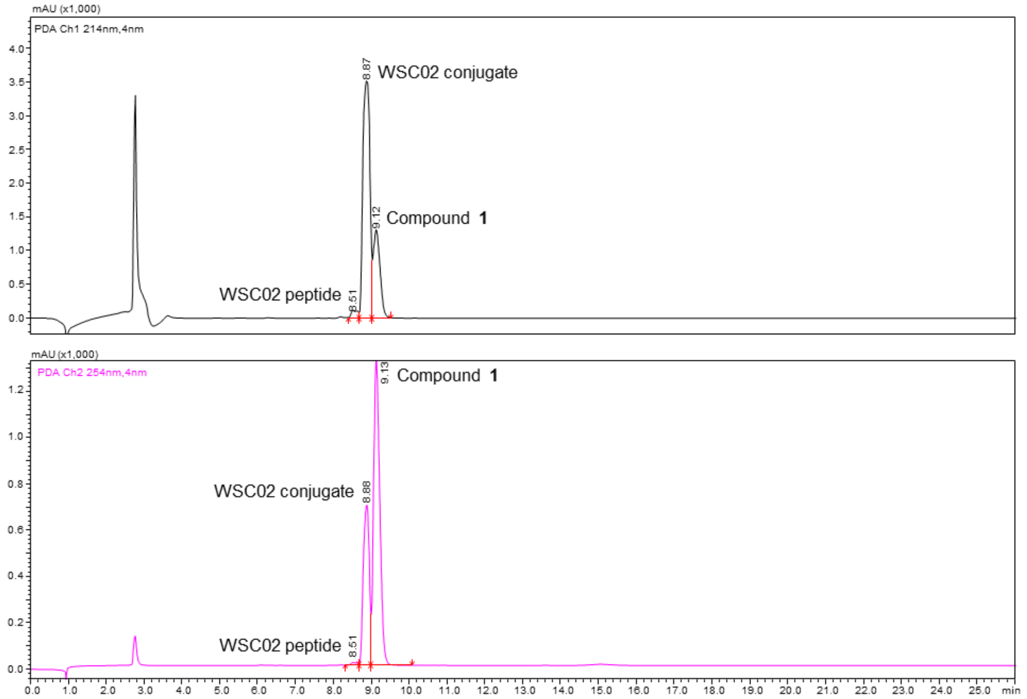

Supplementary Fig. 34: Chromatograms of reaction mixture for WSC02 conjugate purification (214 and 254 nm detection).

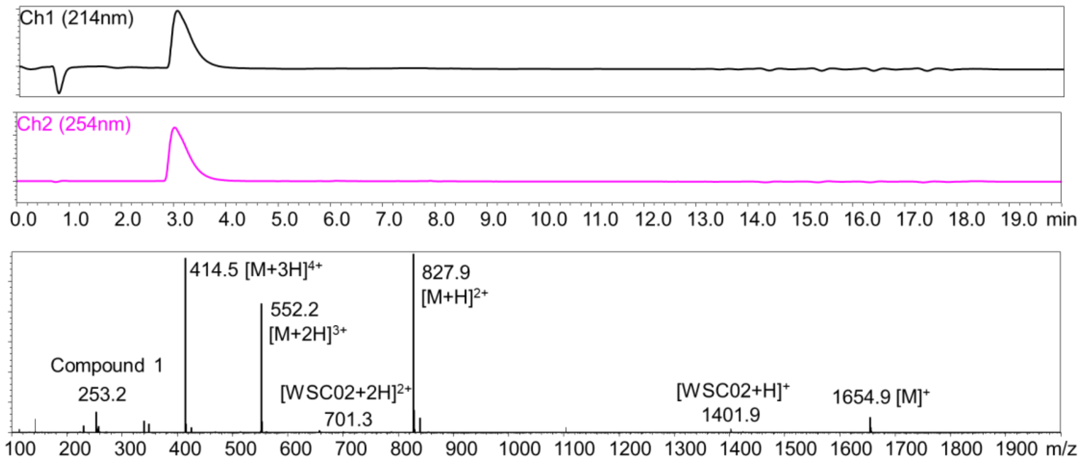

Supplementary Fig. 35: LC-MS analysis of the modified WSC02 peptide (calculated: 1653.9 $[M]^+$, 827.5 $[M+H]^{2+}$, 552.0 $[M+2H]^{3+}$, 414.2 $[M+3H]^{4+}$; found: 1654.9 $[M]^+$, 827.9 $[M+H]^{2+}$, 552.2 $[M+2H]^{3+}$, 414.5 $[M+3H]^{4+}$).

8.1.3.1 WSC02 peptide HPLC conversion

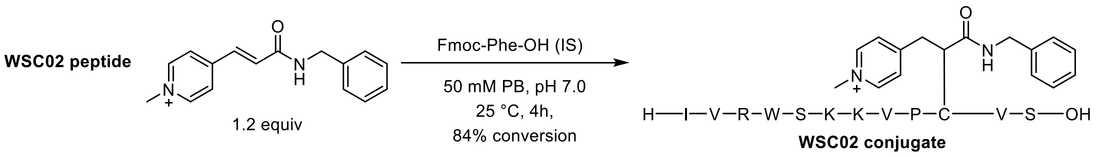

To a fresh solution of WSC02 peptide (1.0 mg/mL in 50 mM PB, pH 7.0) (138 µL, 98.5 nmol) with Fmoc-Phe-OH as internal standard (44.5 µg/mL), was added compound **1** (50 mM in 50 mM PB, pH 7.0) (2.36 µL, 118 nmol). The reaction mixture was mixed at 25 °C for 4 h and transferred to an HPLC vial and 20 µL were injected into analytic HPLC (Zorbax Eclipse XDB-

C18 column, 4.6 × 250 mm, 5 µm) with a flow rate of 1 mL/min with acetonitrile (solvent A, containing 0.1% *v/v* TFA) and Milli-Q water (solvent B, containing 0.1% *v/v* TFA). Mobile phase t = 0 min, 0% B; t = 2 min, 0%; t = 17 min, 100% B; t = 20 min, 100% B; t = 23 min, 0%, t = 25 min, 0%, stop. The concentration of WSC02 peptide (RT 13.2 min) was normalized to the IS (RT 20.1 min) peak areas (AUC) at 254 nm. (Fig S8.14). AUC ratio was converted to concentration through a calibration curve (Fig S8.15).

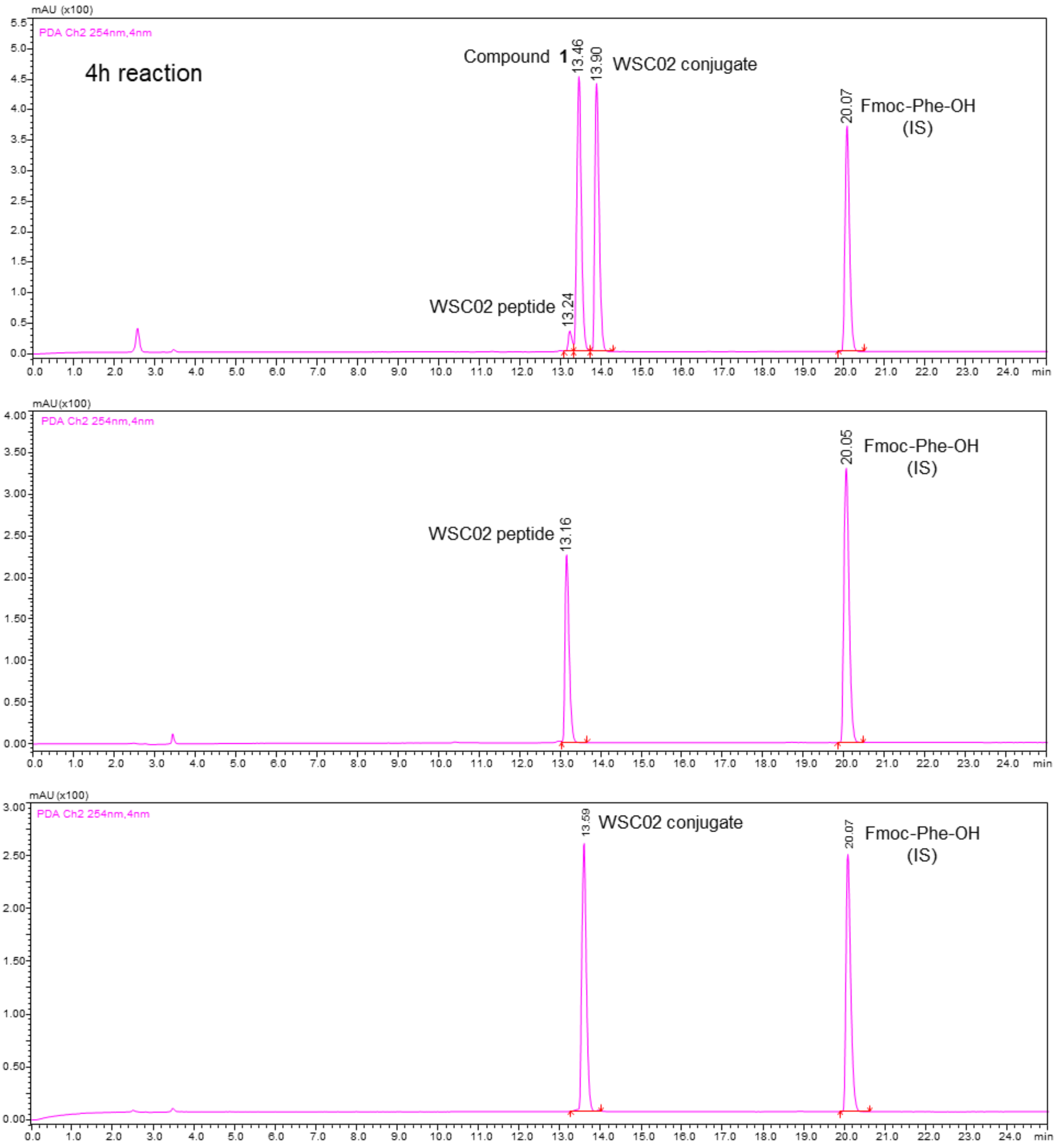

Supplementary Fig. 36: HPLC analysis of the reaction mixture of WSC02 with compound **1** after 4h at 25°C (top), isolated WSC02 conjugate (middle) and fresh solution of WSC02 peptide (bottom) with Fmoc-Phe-OH as internal standard (214 nm detection).

Supplementary Table 2: Calculations for conversion of WSC02 peptide to WSC02 conjugate after 4h reaction in 50 mM PB pH 7.0 at 25°C.

Reaction time	WSC02 area	IS area	AUC ratio	[WSC02] (mg/mL)	Conversion (%)
4h	228599	2831906	0.080723	0.156	84

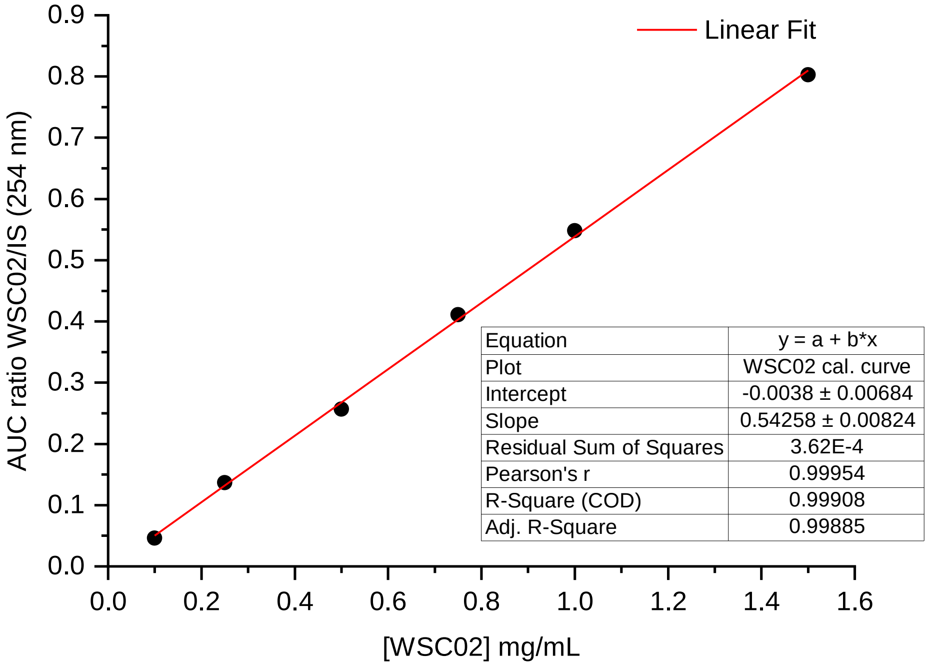

Supplementary Fig. 37: Calibration curve for WSC02 concentration in 50 mM PB pH 7.0, with Fmoc-Phe-OH as internal standard (44.5 µg/mL). Source data are provided as a Source Data file.

8.1.4 RGDC conjugate

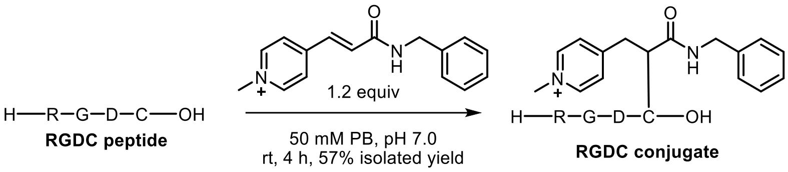

Supplementary Fig. 38: RGDC peptide modification with compound **1** (RGDC conjugate, RT 7.7 min and excess of reagent **1**, RT 10.7 min, method A).

LC-MS: calculated: 702.3 $[M]^+$, found: 702.4 $[M]^+$.

ESI-HRMS Calculated for $C_{31}H_{44}N_9O_9S^+$ $[M]^+$: 702.3028, found 702.3029; calculated for $C_{31}H_{45}N_9O_9S^{2+}$ $[M+H]^{2+}$: 351.6550, found 351.6659.

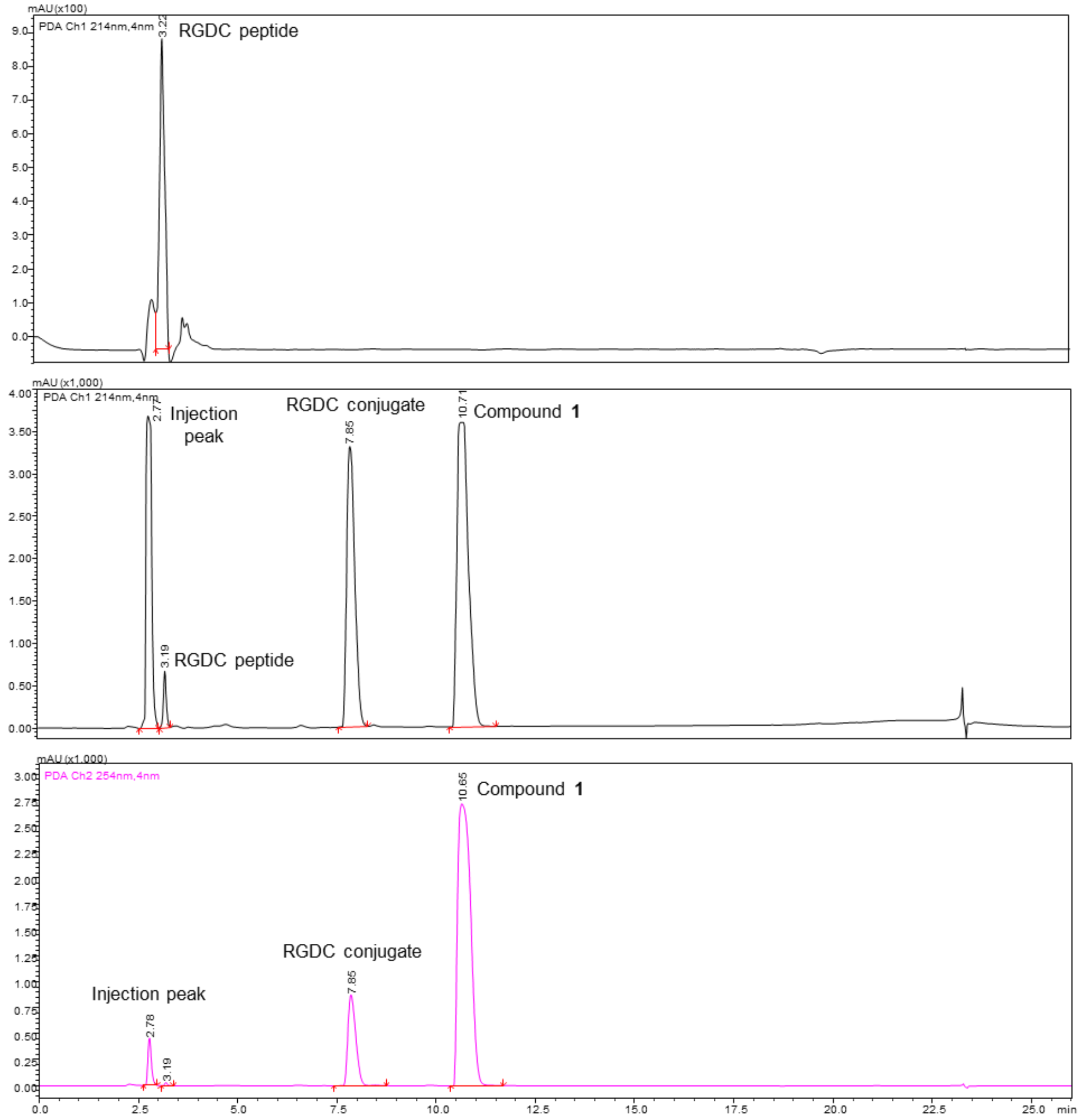

Supplementary Fig. 39: Chromatograms of initial RGDC peptide (214 nm, top) and reaction mixture for RGDC conjugate purification (214 and 254 nm detection, middle and bottom, respectively).

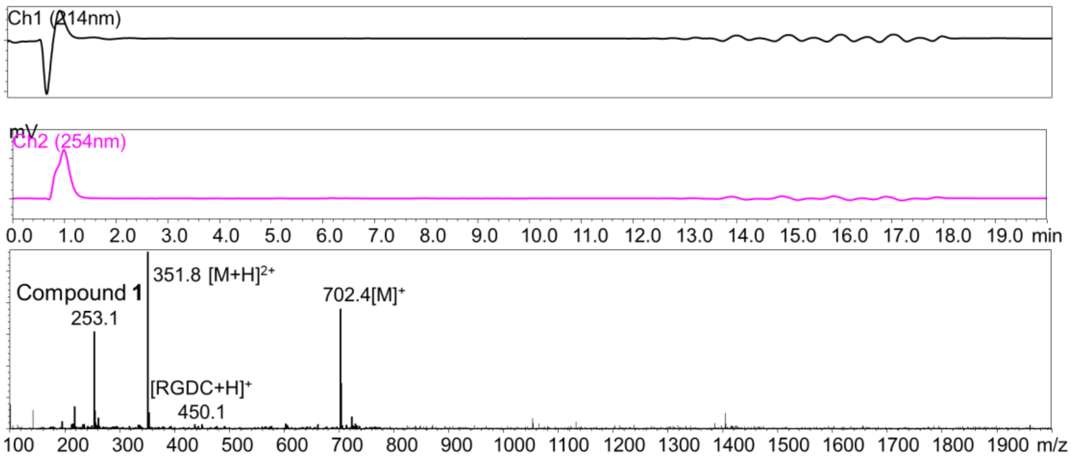

Supplementary Fig. 40: LC-MS analysis of the modified RGDC (calculated: 702.3 $[M]^+$, found: 702.4 $[M]^+$).

8.1.4.1 RGDC peptide modification and analytical HPLC analysis

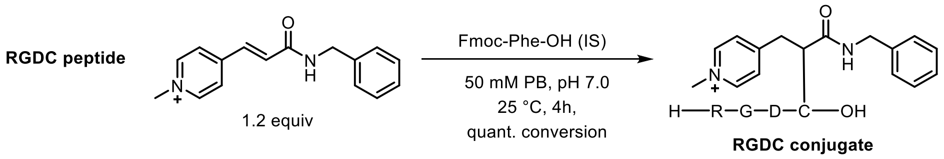

To a fresh solution of RGDC peptide (1.0 mg/mL in 50 mM PB, pH 7) (100 µL, 0.222 µmol) with Fmoc-Phe-OH as internal standard (138.8 µg/mL) was added compound **1** (50 mM in 50 mM PB, pH7) (5.30 µL, 0.267 µmol). The reaction mixture was mixed at 25 °C for 4 h and transferred to an HPLC vial and 20 µL were injected into analytic HPLC (Zorbax Eclipse XDB-C18 column, 4.6 × 250 mm, 5 µm) with a flow rate of 1 mL/min with acetonitrile (solvent A, containing 0.1% *v/v* TFA) and Milli-Q water (solvent B, containing 0.1% *v/v* TFA). Mobile phase t = 0 min, 0% B; t = 2 min, 0%; t = 17 min, 100% B; t = 20 min, 100% B; t = 23 min, 0%, t = 25 min, 0%, stop. The conversion was complete as no starting peptide could be observed in the reaction mixture chromatogram. RGDC peptide (RT 10.7 min), RGDC conjugate (RT 12.5 min), compound **1** (RT 13.9 min) and IS (RT 20.1 min) at 214 nm detection (Fig S8.19).

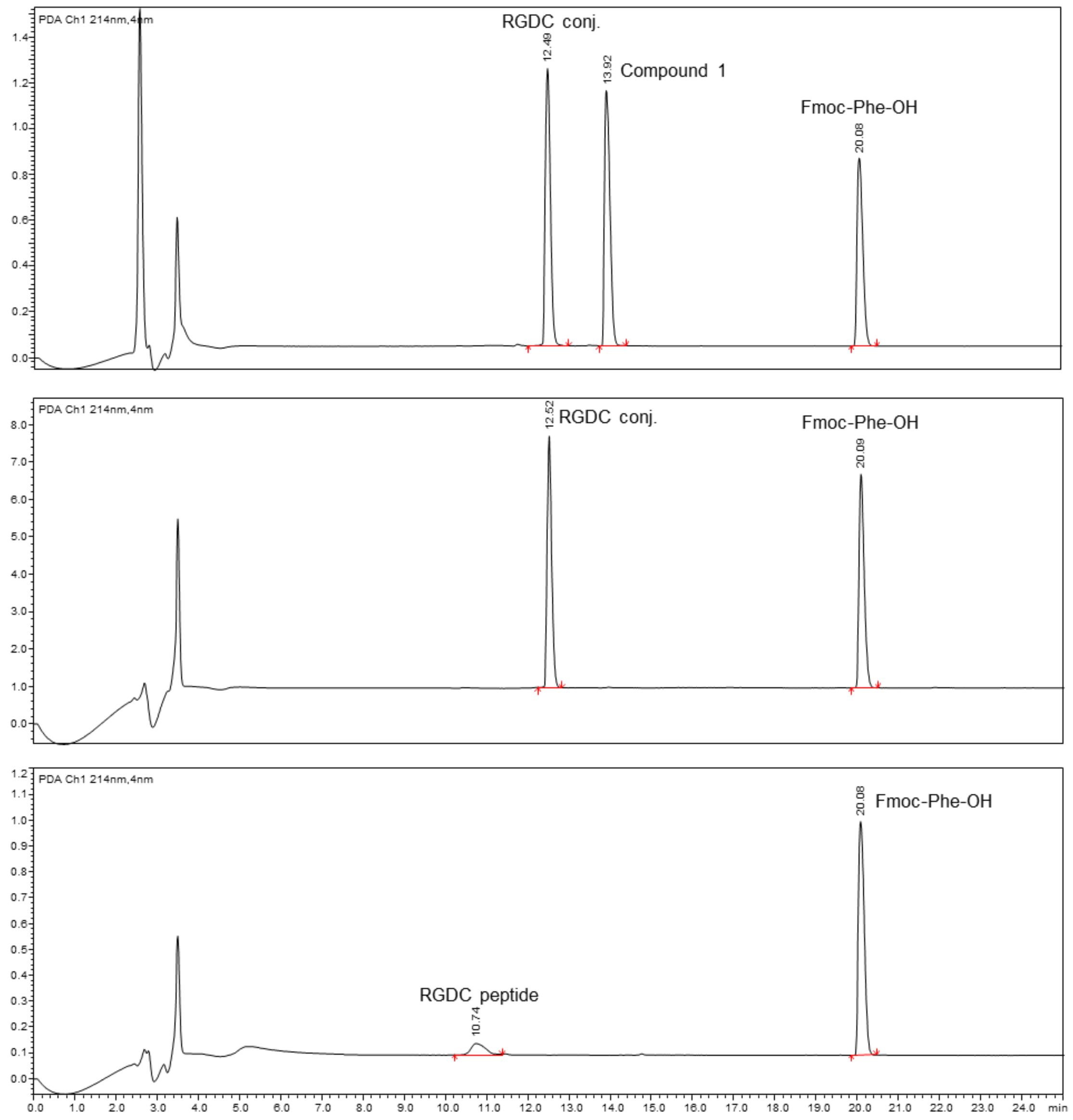

Supplementary Fig. 41: HPLC analysis of the reaction mixture of RGDC with compound **1** after 4h at 25°C (top), isolated RGDC conjugate (middle) and fresh solution of RGDC peptide (bottom) with Fmoc-Phe-OH as internal standard (214 nm detection).

8.1.5 Tet conjugate

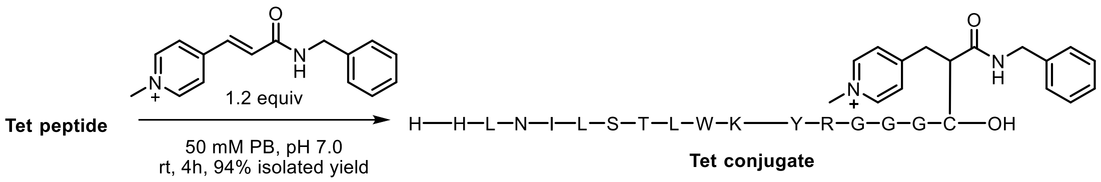

Supplementary Fig. 42: Tet peptide modification with compound **1** (Tet conjugate, RT 13.9 min and excess of reagent **1**, RT 10.7 min, method A).

LC-MS calculated: 1036.0$[M+H]^{2+}$, 691.0 $[M+2H]^{3+}$, 518.5 $[M+3H]^{4+}$, 415.0 $[M+4H]^{5+}$; found: 1036.2$[M+H]^{2+}$, 691.0 $[M+2H]^{3+}$, 518.7 $[M+3H]^{4+}$, 415.0 $[M+4H]^{5+}$).

ESI-HRMS Calculated for $C_{98}H_{147}N_{26}O_{22}S^{3+}$ $[M+2H]^{3+}$: 691.0307, found 691.0312; calculated for $C_{98}H_{148}N_{26}O_{22}S^{3+}$ $[M+3H]^{4+}$:518.5248, found 518.5258.

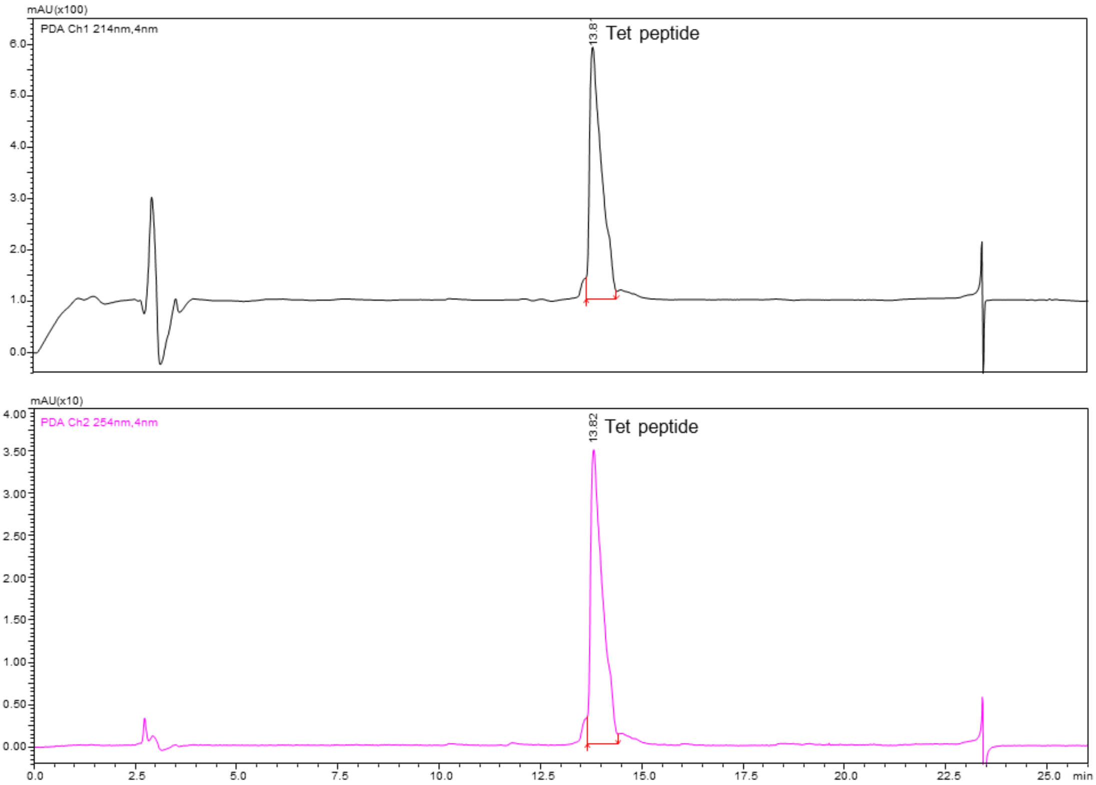

Supplementary Fig. 43: Chromatograms of initial Tet peptide (214 and 254 nm detection).

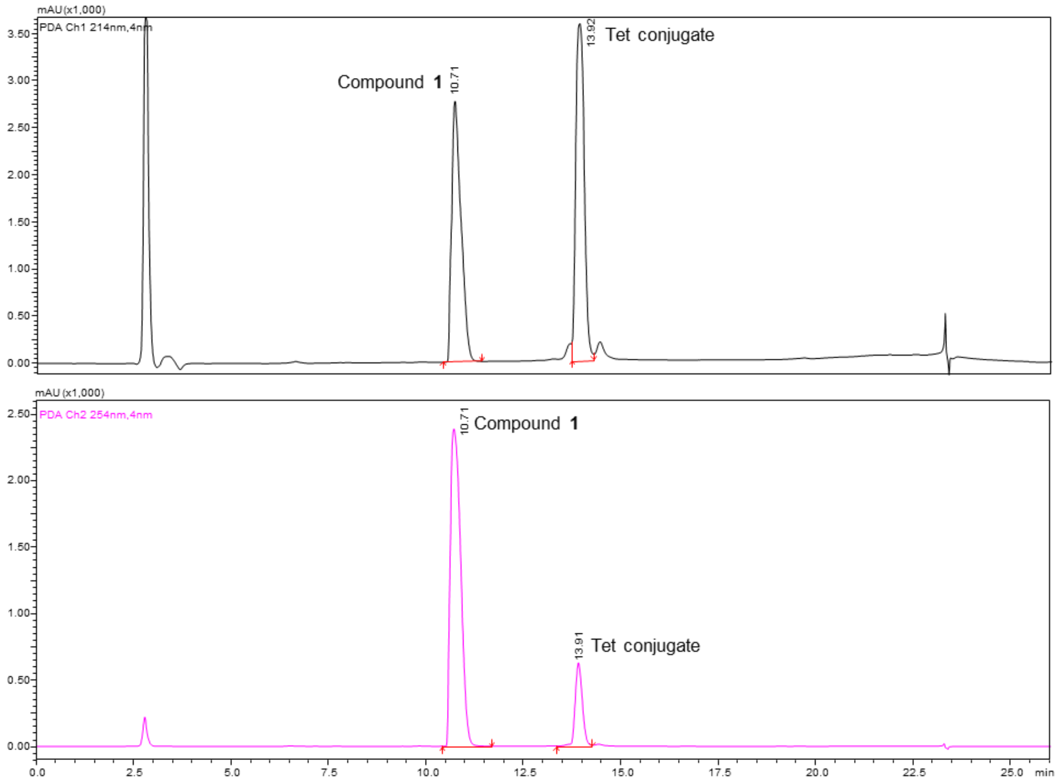

Supplementary Fig. 44: Chromatograms of reaction mixture for Tet conjugate purification (214 and 254 nm detection).

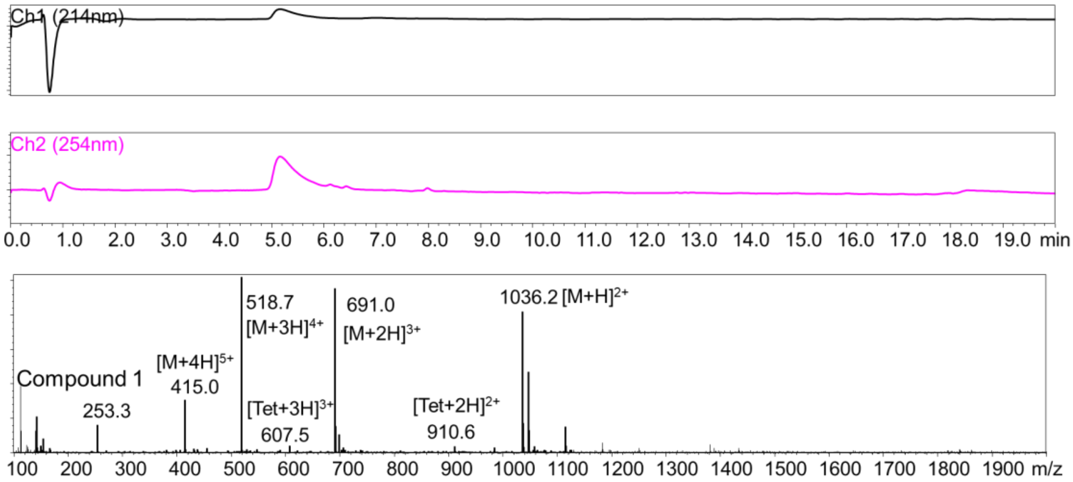

Supplementary Fig. 45: LC-MS analysis of the modified Tet peptide (calculated: 1036.0 $[M+H]^{2+}$, 691.0 $[M+2H]^{3+}$, 518.5 $[M+3H]^{4+}$, 415.0 $[M+4H]^{5+}$; found: 1036.2$[M+H]^{2+}$, 691.0 $[M+2H]^{3+}$, 518.7 $[M+3H]^{4+}$, 415.0 $[M+4H]^{5+}$).

8.1.5.1 Tet peptide modification and analytical HPLC analysis

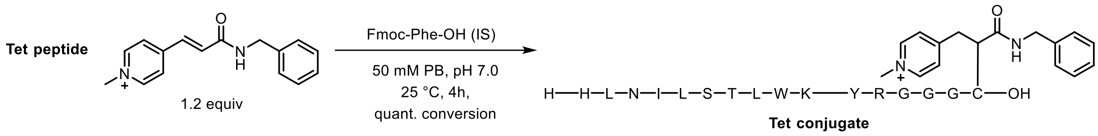

To a fresh solution of Tet peptide (1.0 mg/mL in 50 mM PB, pH 7:ACN, 3:1) (133 µL, 0.055 µmol) with Fmoc-Phe-OH as internal standard (34.3 µg/mL) was added compound **1** (50 mM in 50 mM PB, pH7) (6.60 µL, 0.066 µmol). The reaction mixture was mixed at 25 °C for 4 h and transferred to an HPLC vial and 20 µL were injected into analytic HPLC (Zorbax Eclipse XDB-C18 column, 4.6 × 250 mm, 5 µm) with a flow rate of 1 mL/min with acetonitrile (solvent A, containing 0.1% *v/v* TFA) and Milli-Q water (solvent B, containing 0.1% *v/v* TFA). Mobile phase t = 0 min, 0% B; t = 2 min, 0%; t = 17 min, 100% B; t = 20 min, 100% B; t = 23 min, 0%, t = 25 min, 0%, stop. The conversion was complete as no starting peptide could be observed in the reaction mixture chromatogram. Tet peptide (RT 10.7 min), Tet conjugate (RT 12.5 min), compound **1** (RT 13.9 min) and IS (RT 20.1 min) at 214 nm detection (Fig S8.24).

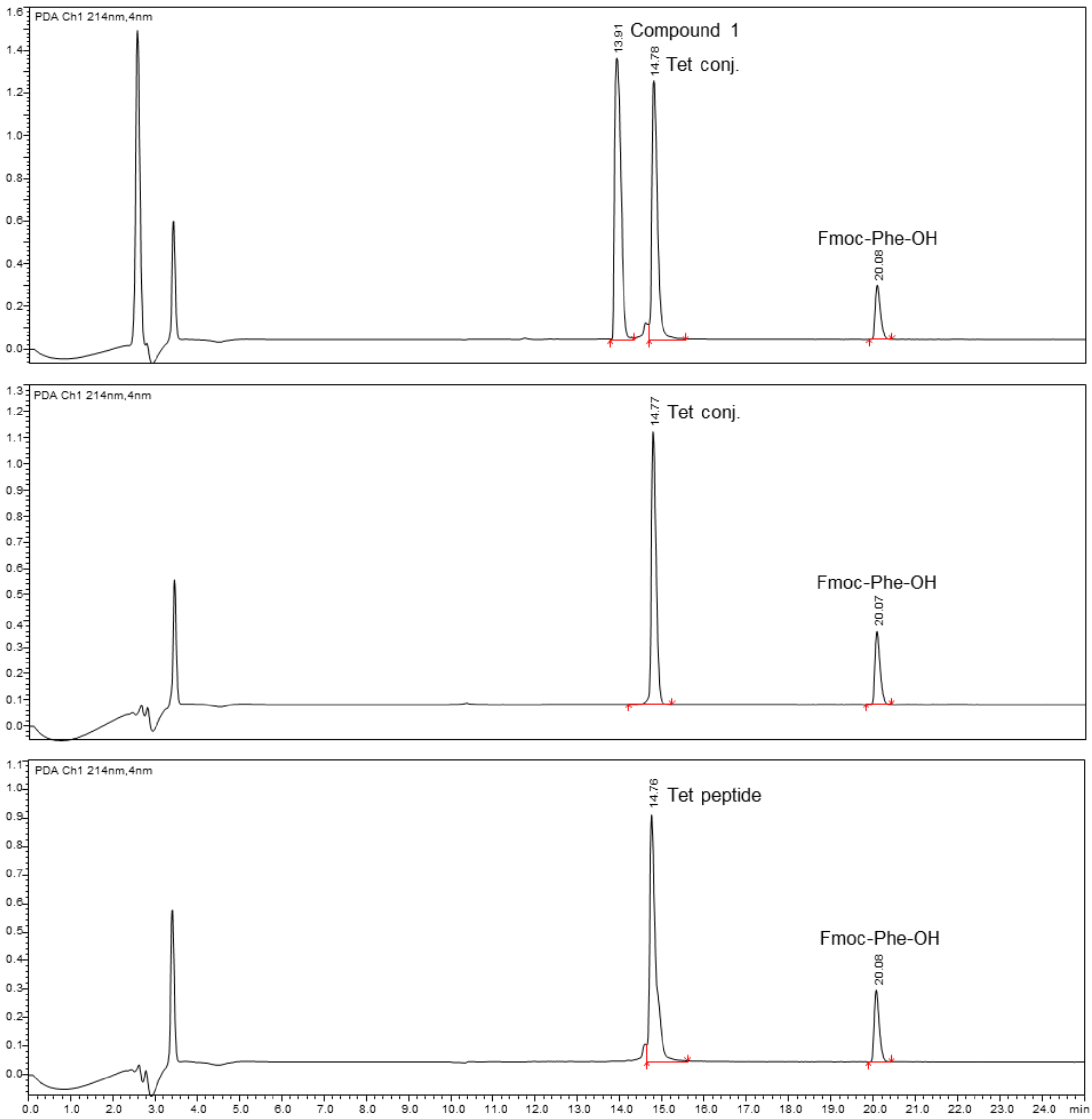

Supplementary Fig. 46: HPLC analysis of the reaction mixture of Tet with compound **1** after 4h at 25°C (top), isolated Tet conjugate (middle) and fresh solution of Tet peptide (bottom) with Fmoc-Phe-OH as internal standard (214 nm detection).

8.1.6 EK1C conjugate

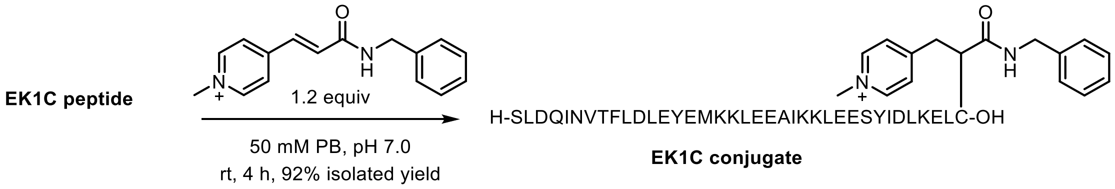

Supplementary Fig. 47: EK1C peptide modification with compound **1** (EK1C conjugate, RT 18.8 min and excess of reagent **1**, RT 10.8 min, method A).

LC-MS: calculated: 1563.1 $[M+2H]^{3+}$,1172.6 $[M+3H]^{4+}$, 938.3 $[M+4H]^{5+}$, 782.1 $[M+5H]^{6+}$, 670.5 $[M+6H]^{7+}$; found: 1563.6 $[M+2H]^{3+}$,1172.9 $[M+3H]^{4+}$, 938.5 $[M+4H]^{5+}$, 782.3 $[M+5H]^{6+}$, 670.7 $[M+6H]^{7+}$).

ESI-HRMS Calculated for $C_{215}H_{342}N_{46}O_{66}S_2^{4+}$ $[M+3H]^{4+}$: 1172.6076, found 1172.6101; calculated for $C_{215}H_{343}N_{46}O_{66}S_2^{5+}$ $[M+4H]^{5+}$: 938.2876, found 938.2902; calculated for $C_{215}H_{344}N_{46}O_{66}S_2^{6+}$ $[M+5H]^{6+}$: 782.0742, found 782.0760; calculated for $C_{215}H_{345}N_{46}O_{66}S_2^{7+}$ $[M+6H]^{7+}$: 670.4932, found 670.4954.

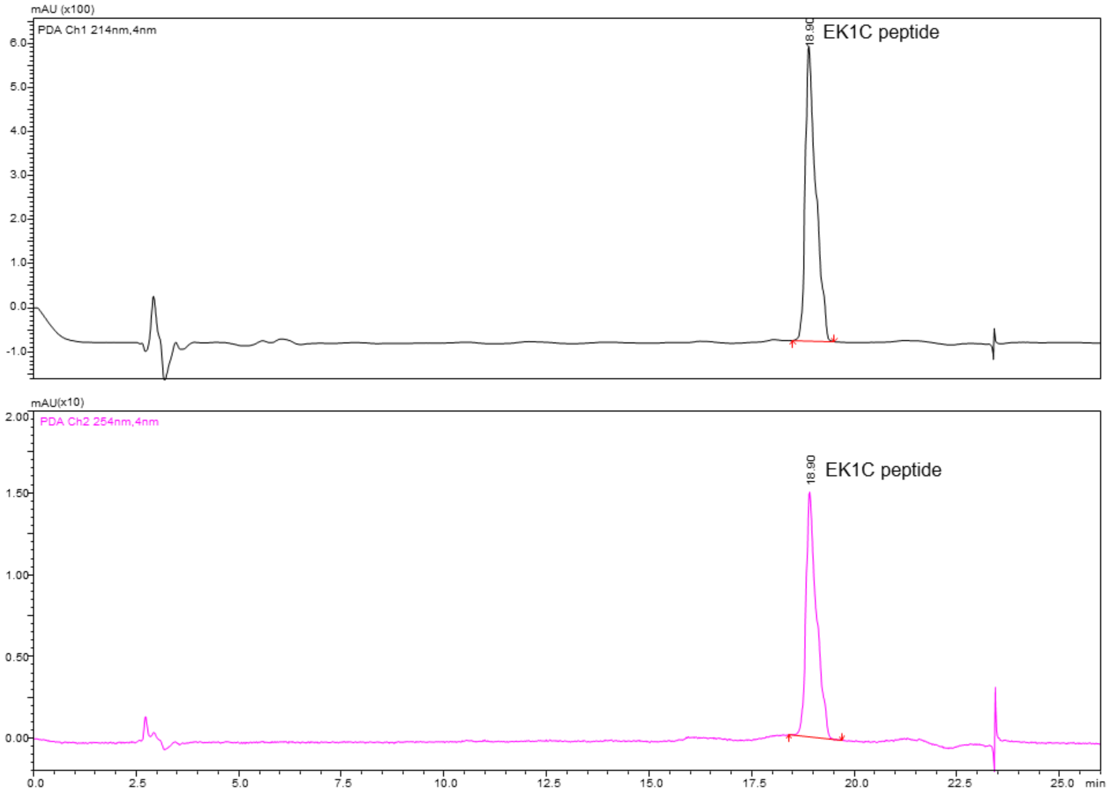

Supplementary Fig. 48: Chromatograms of initial EK1C peptide (214 and 254 nm detection).

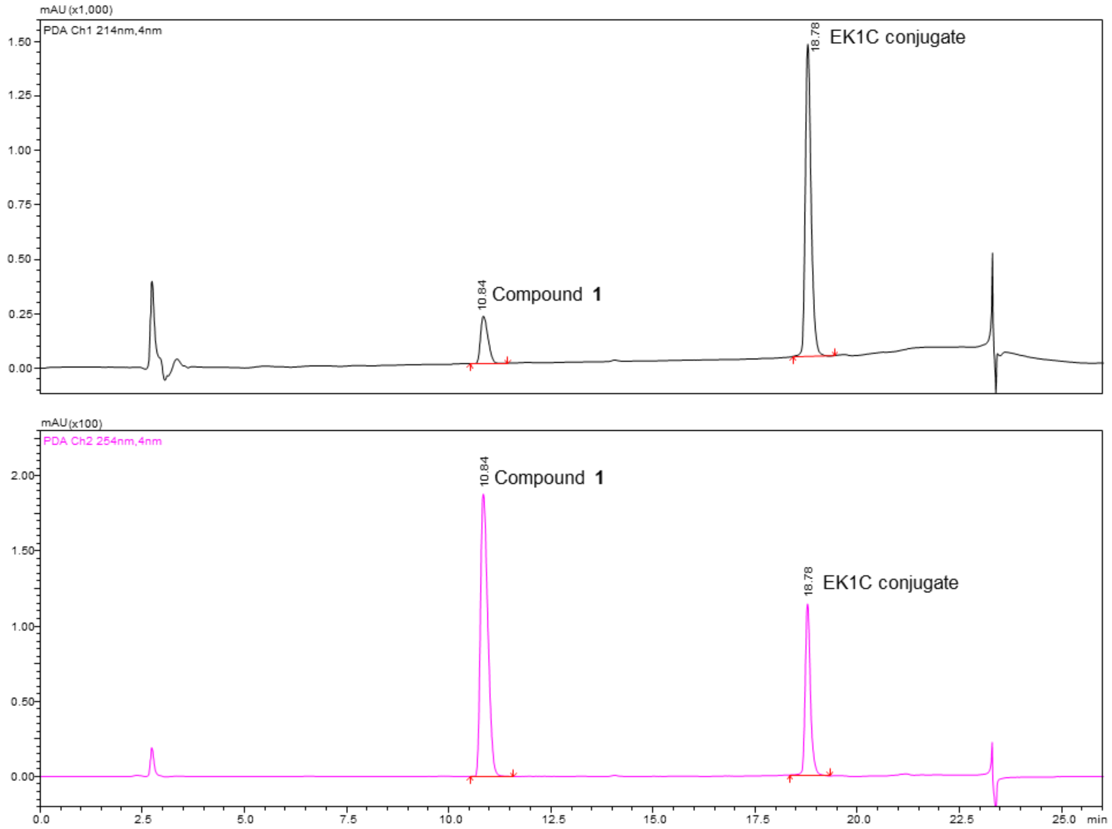

Supplementary Fig. 49: Chromatograms for EK1C conjugate purification (214 and 254 nm detection).

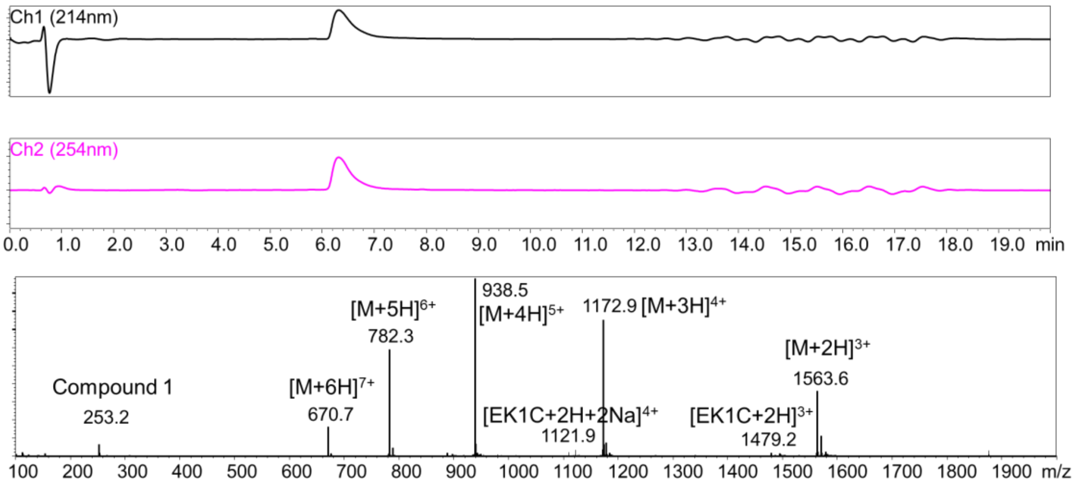

Supplementary Fig. 50: LC-MS analysis of the modified EK1C peptide (calculated:1563.1 $[M+2H]^{3+}$,1172.6 $[M+3H]^{4+}$, 938.3 $[M+4H]^{5+}$, 782.1 $[M+5H]^{6+}$, 670.5 $[M+6H]^{7+}$; found: 1563.6 $[M+2H]^{3+}$,1172.9 $[M+3H]^{4+}$, 938.5 $[M+4H]^{5+}$, 782.3 $[M+5H]^{6+}$, 670.7 $[M+6H]^{7+}$).

8.1.6.1 EK1C peptide modification and analytical HPLC analysis

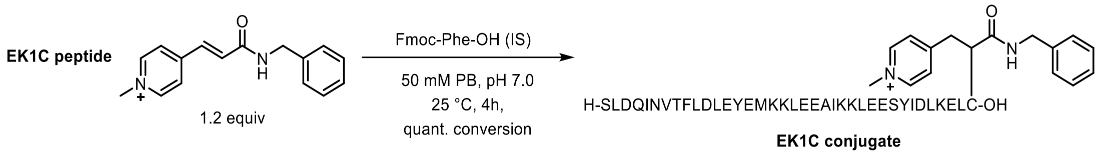

To a fresh solution of EK1C peptide (1.0 mg/mL in 50 mM PB, pH 7) (100 µL, 0.020 µmol) with Fmoc-Phe-OH as internal standard (14.1 µg/mL) was added compound **1** (50 mM in 50 mM PB, pH7) (0.54 µL, 0.027 µmol). The reaction mixture was mixed at 25 °C for 4 h and transferred to an HPLC vial and 20 µL were injected into analytic HPLC (Zorbax Eclipse XDB-C18 column, 4.6 × 250 mm, 5 µm) with a flow rate of 1 mL/min with acetonitrile (solvent A, containing 0.1% *v/v* TFA) and Milli-Q water (solvent B, containing 0.1% *v/v* TFA). Mobile phase t = 0 min, 0% B; t = 2 min, 0%; t = 17 min, 100% B; t = 20 min, 100% B; t = 23 min, 0%, t = 25 min, 0%, stop. The conversion was complete as no starting peptide could be observed in the reaction mixture chromatogram. EK1C peptide (RT 17.1 min), EK1C conjugate (RT 16.9 min), compound **1** (RT 14.0 min) and IS (RT 20.1 min) at 214 nm detection (Fig S8.29).

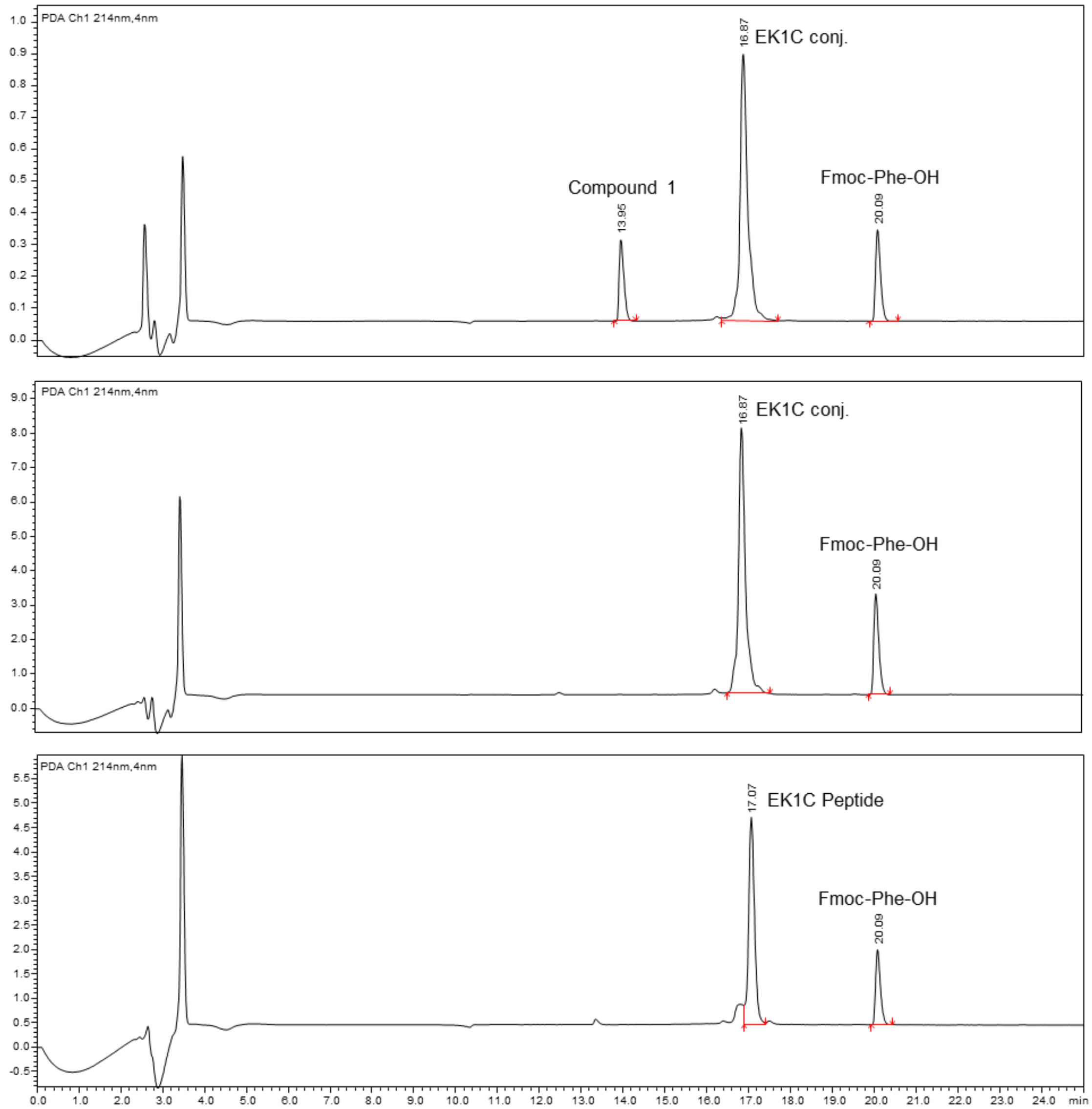

Supplementary Fig. 51: HPLC analysis of the reaction mixture of EK1C with compound **1** after 4h at 25°C (top), isolated EK1C conjugate (middle) and fresh solution of EK1C peptide (bottom) with Fmoc-Phe-OH as internal standard (214 nm detection).

8.2 Stability studies PC8 conjugate (11) and PC8-maleimide conjugate

Semi-quantitative analyses were performed using single injection per sample.

8.2.1 Stability under different pHs

PC8 conjugate (100 µg) was dissolved in 50 mM PB pH 6.0, 7.0 or 8.0 (200 µL). Fmoc-Phe-OH (0.025 mg/mL) was also added as an internal standard. The resultant solution was incubated for 45 h at 25 °C. 20 µL of each solution was injected into analytic HPLC (Atlantis T3 column (100 Å, 4.6 × 100 mm, 5 µm) with a flow rate of 1 mL/min with acetonitrile (solvent A, containing 0.1% *v/v* TFA) and Milli-Q water (solvent B, containing 0.1% *v/v* TFA). Mobile phase t = 0 min, 5% B; t = 15 min, 50%; t = 17 min, 100% B; t = 19 min, 100% B; t = 22 min, 5%, t = 26 min, 5%, stop. The stability study of PC8 conjugate **11** was determined based on the peaks: PC8 conjugate **11** (RT 9.7 min) and compound **1** (RT 10.8 min) were normalized to the IS (RT 16.0 min) peak area at 214 nm. The stability study of PC8 maleimide conjugate was determined based on the peaks: PC8 maleimide conjugate (RT 10.46 min), hydrolyzed PC8 maleimide conjugates (RT 10.3 and 10.4 min) and were normalized to the IS (RT 15.8 min) peak area at 214 nm.

a Stability of PC8 conjugate (**11**) at different pHs (pH = 6, 7 and 8)

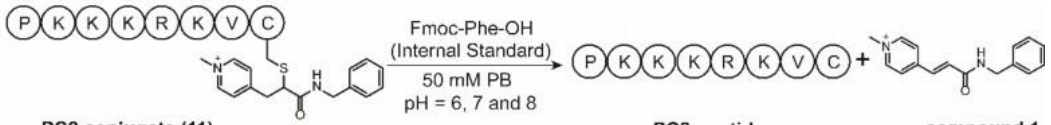

PC8 conjugate (11) **PC8 peptide** **compound 1**

b Stability of PC8 conjugate (**11**) at pH 6

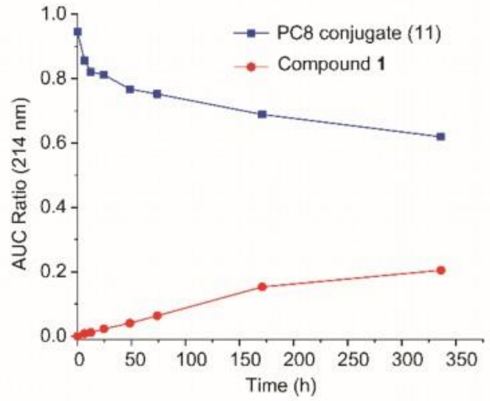

t (h)	Percentage of PC8 conjugate remained
0	100%
7	90%
13	87%
25	86%
49	81%
74	80%
171	73%
336	65%

c Stability of PC8 conjugate (**11**) at pH 7

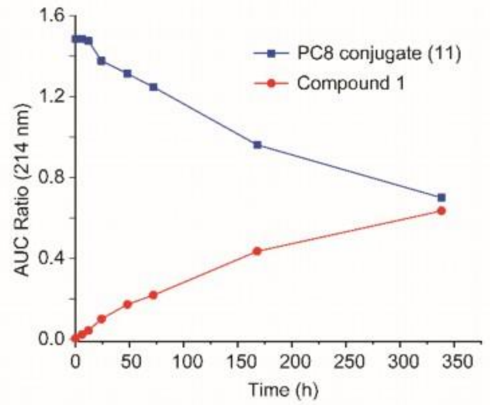

t (h)	Percentage of PC8 conjugate remained
0	100
6	100
12	99
24	93
48	88
72	84
168	65
338	47

d Stability of PC8 conjugate (**11**) at pH 8

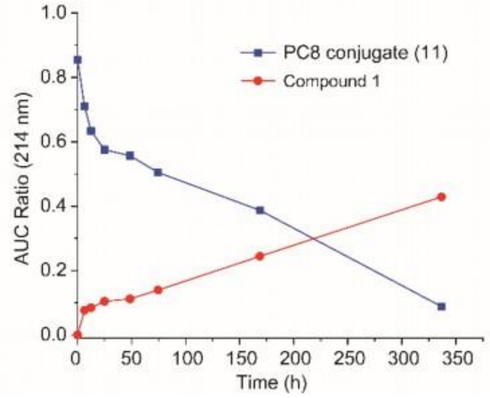

t (h)	Percentage of PC8 conjugate remained
0	100
7	83
13	74
25	67
49	65
75	59
169	45
337	10

Supplementary Fig. 52: Stability studies of PC8 conjugate **11** at three different pHs (pH = 6, 7 and 8). Source data are provided as a Source Data file.

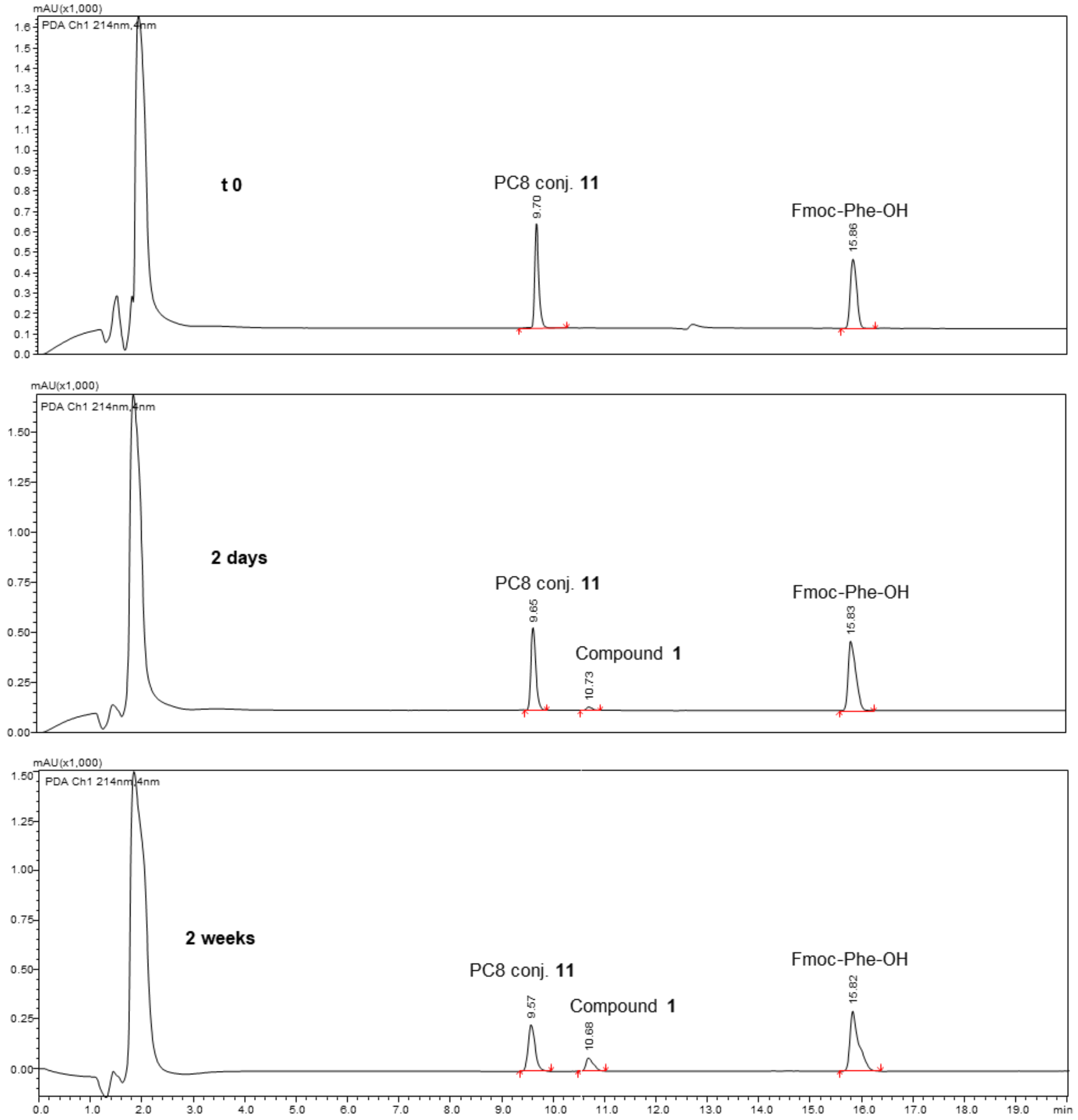

Supplementary Fig. 53: Representative chromatograms for PC8 conjugate **11** stability in 50 mM PB, pH 6.0, at t0 (top), after 2 days and 2 weeks (top to bottom) incubation at 25 °C.

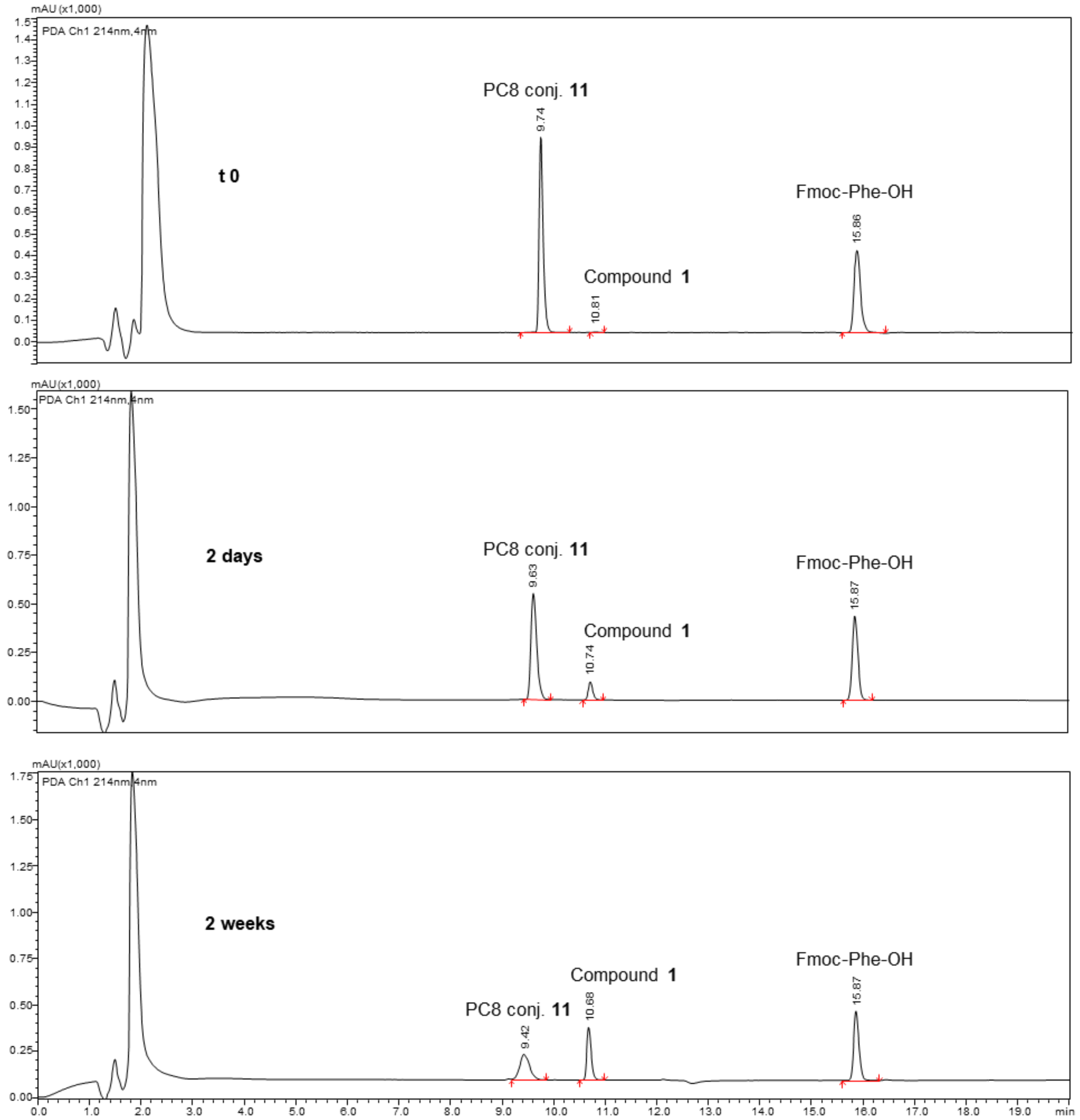

Supplementary Fig. 54: Representative chromatograms for PC8 conjugate **11** stability in 50 mM PB, pH 7.0, at t0 (top), after 2 days and 2 weeks (top to bottom) incubation at 25 °C.

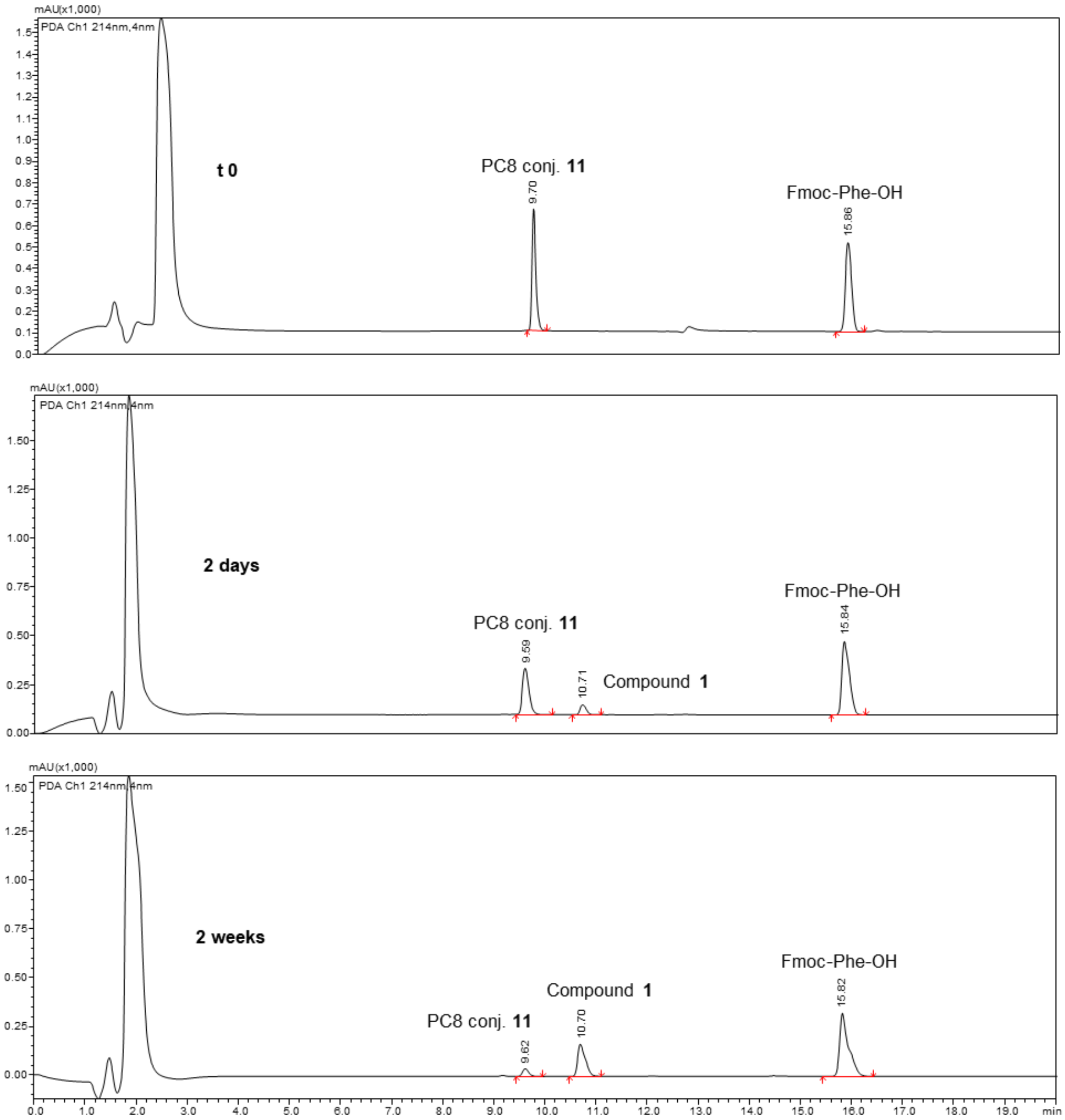

Supplementary Fig. 55: Representative chromatograms for PC8 conjugate **11** stability in 50 mM PB, pH 8.0, at t0 (top), after 2 days and 2 weeks (top to bottom) incubation at 25 °C.

a Stability of PC8-maleimide conjugate at different pHs (pH = 6, 7 and 8)

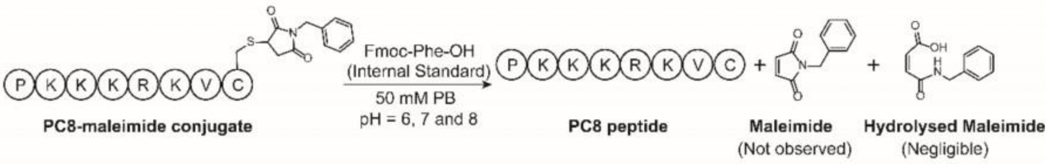

b Stability of PC8-maleimide conjugate at pH 6

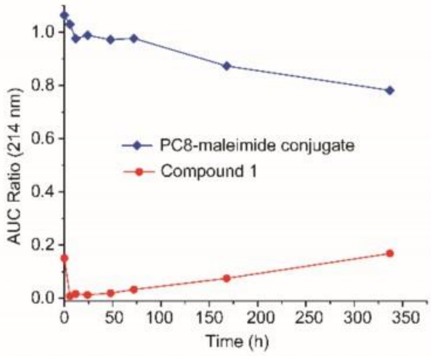

t (h)	Percentage of PC8-maleimide conjugate remained
0	100%
6	97%
12	92%
24	93%
48	91%
72	92%
168	82%
335	73%

c Stability of PC8-maleimide conjugate at pH 7

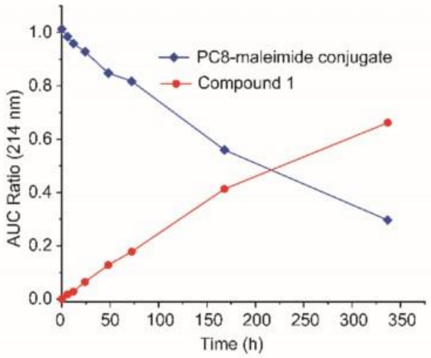

t (h)	Percentage of PC8-maleimide conjugate remained
0	100%
6	97%
12	95%
24	92%
48	84%
72	81%
168	55%
335	29%

d Stability of PC8-maleimide conjugate at pH 8

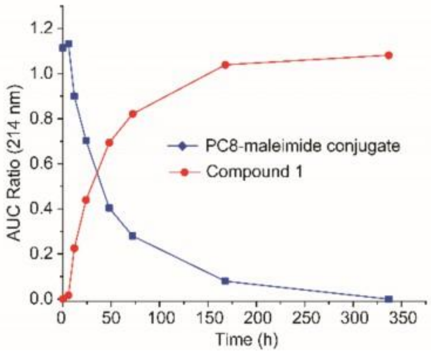

t (h)	Percentage of PC8-maleimide conjugate remained
0	100%
6	102%
12	81%
24	63%
48	36%
72	25%
168	7%
335	0%

Supplementary Fig. 56: Stability studies of PC8-maleimide conjugate at three different pHs (pH = 6, 7 and 8). Source data are provided as a Source Data file.

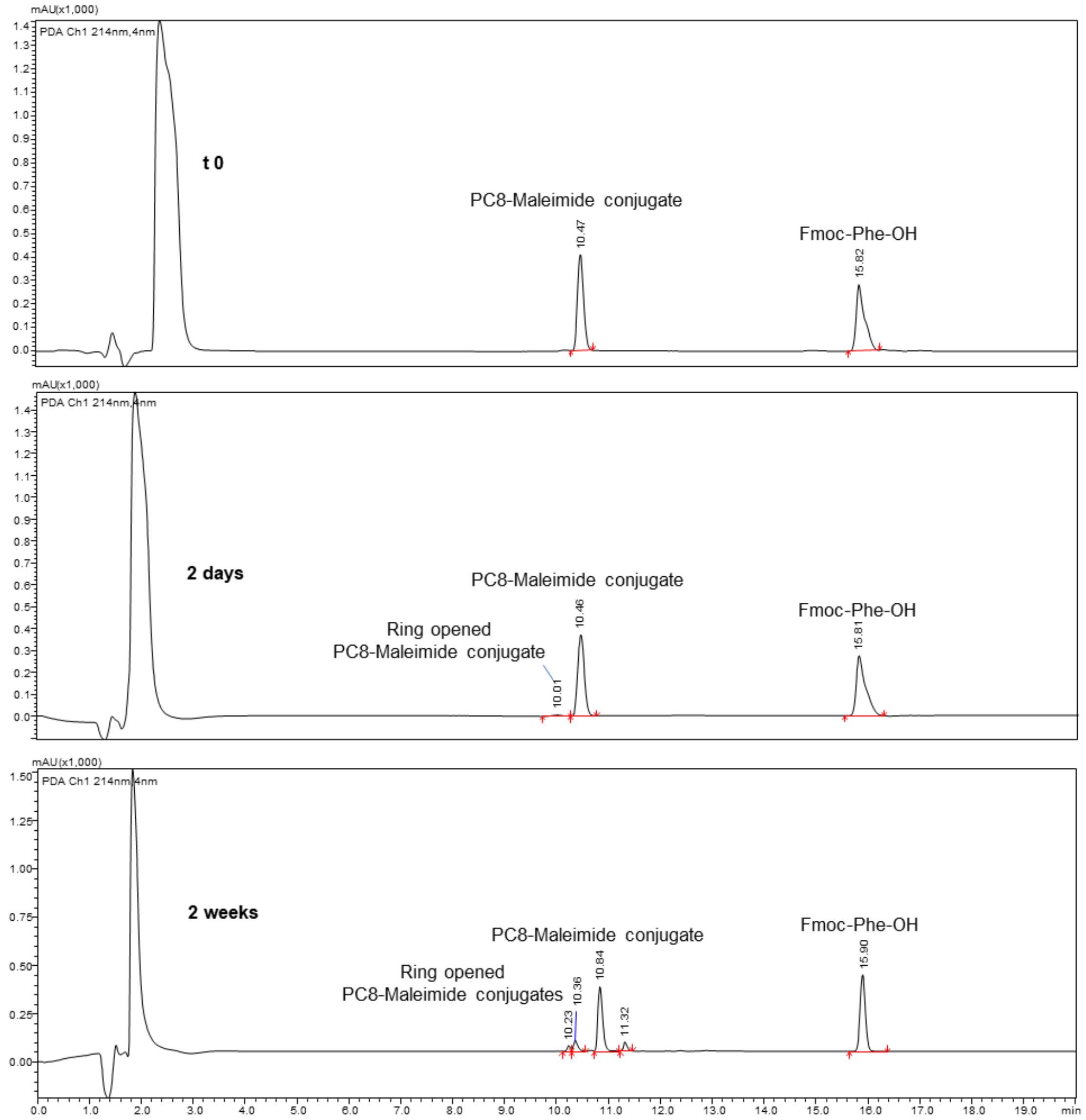

Supplementary Fig. 57: Representative chromatograms for PC8 maleimide conjugate stability in 50 mM PB, pH 6.0, at t_0 (top), after 2 days and 2 weeks (top to bottom) incubation at 25 °C.

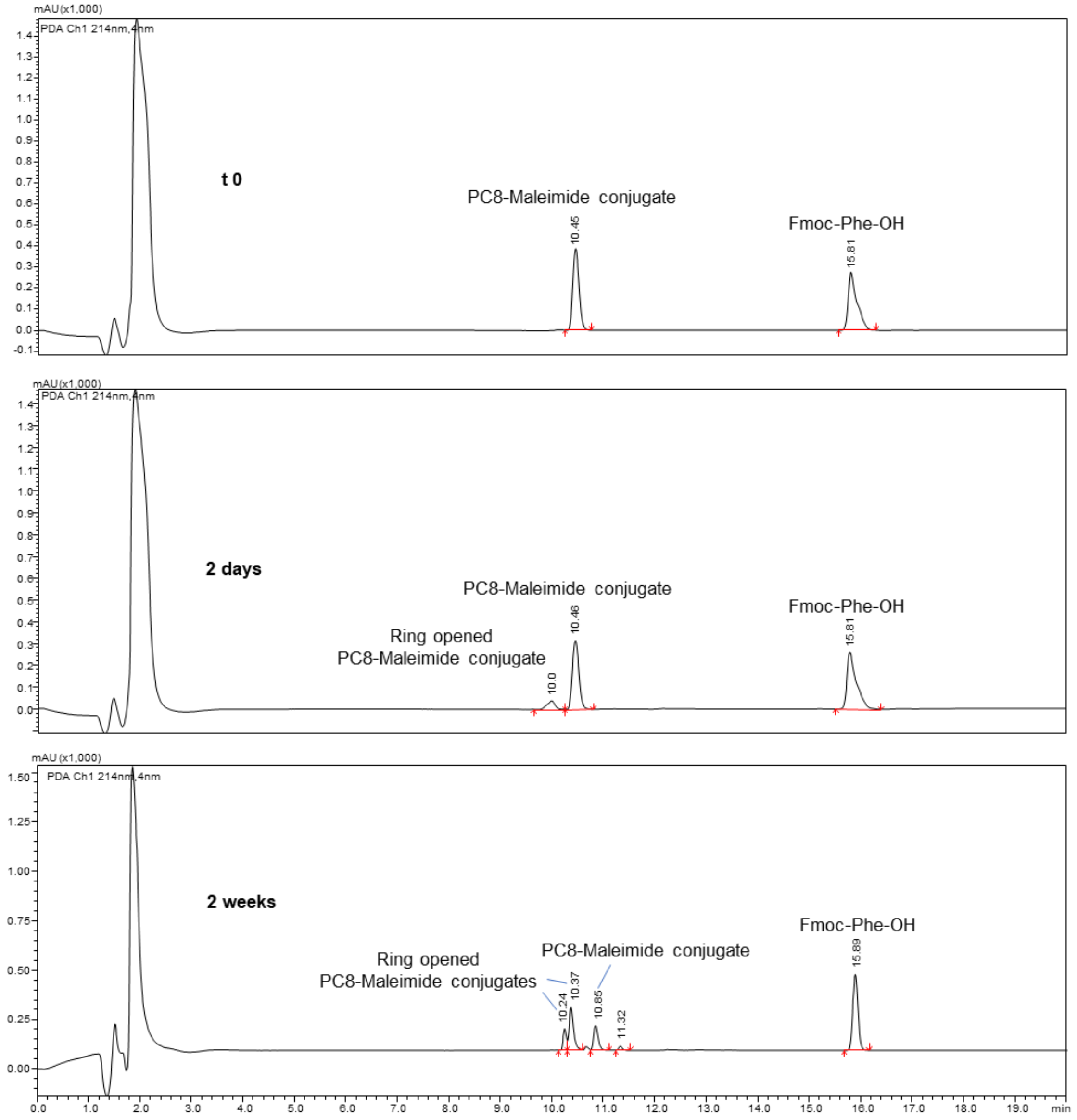

Supplementary Fig. 58: Representative chromatograms for PC8 maleimide conjugate stability in 50 mM PB, pH 7.0, at t_0 (top), after 2 days and 2 weeks (top to bottom) incubation at 25 °C.

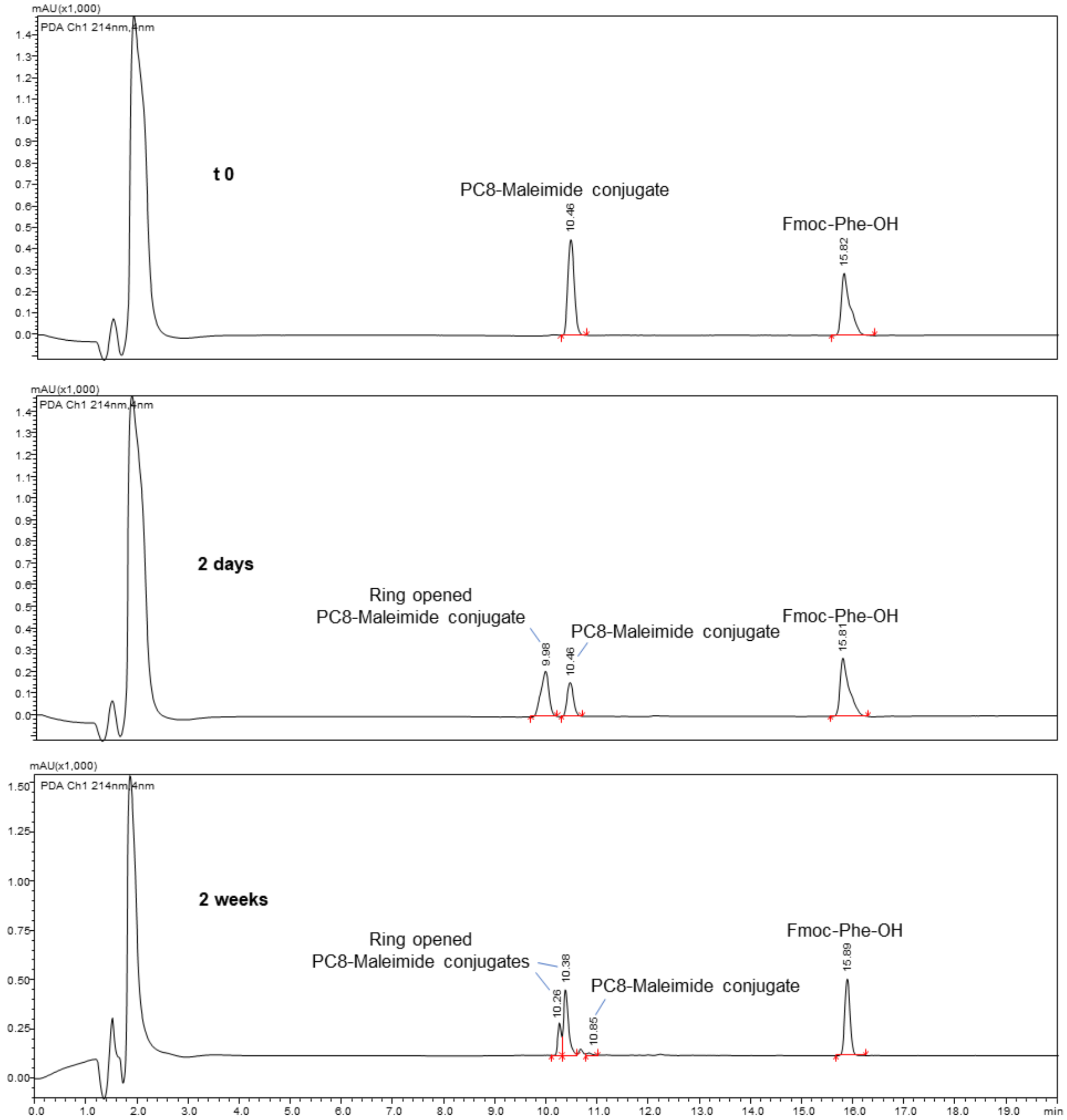

Supplementary Fig. 59: Representative chromatograms for PC8 maleimide conjugate stability in 50 mM PB, pH 8.0, at t_0 (top), after 2 days and 2 weeks (top to bottom) incubation at 25 °C.

8.2.2 Stability of PC8 conjugate (11) and PC8-maleimide conjugate in 1 mM glutathione (GSH) in physiological pH

PC8 conjugate **11** (0.5 mg/mL) and GSH (1 mM) were dissolved in 1x DPBS, pH 7.4. Fmoc-Phe-OH (0.025 mg/mL) was also added as an internal standard. The resultant solution was incubated for 2 weeks. 20 µL of each solution was injected into analytic HPLC (Atlantis T3 column (100 Å, 4.6 × 100 mm, 5 µm) with a flow rate of 1 mL/min with acetonitrile (solvent A, containing 0.1% *v/v* TFA) and Milli-Q water (solvent B, containing 0.1% *v/v* TFA). Mobile phase t = 0 min, 5% B; t = 15 min, 50%; t = 17 min, 100% B; t = 19 min, 100% B; t = 22 min, 5%, t = 26 min, 5%, stop to evaluate PC8 conjugate stability. The stability study was determined based on the peaks: PC8 conjugate **11** (RT 9.7 min), compound **1** (RT 10.7 min) and GSH conjugates (RT 9.0 and 9.4 min) were normalized to the IS (RT 15.8 min) peak area at 214 nm.

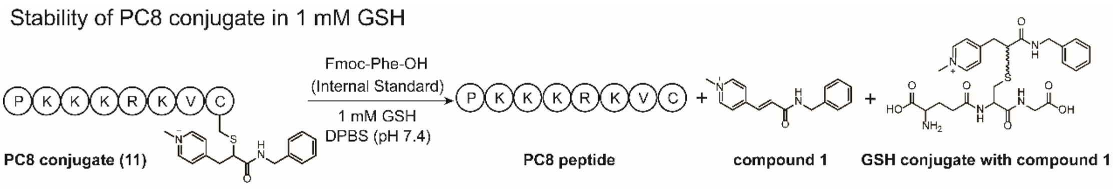

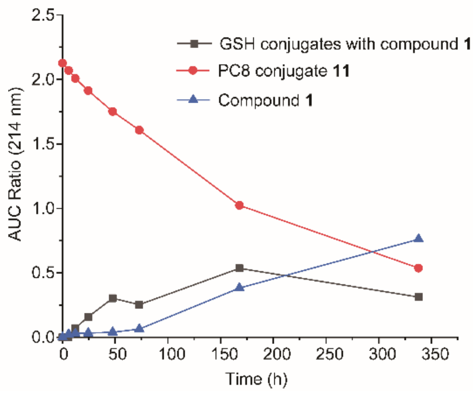

time	Percentage of PC8 conjugate remained
0 h	100%
6 h	97%
12 h	94%
1 day	90%
2 days	82%
3 days	76%
7 days	48%
14 days	25%

Supplementary Fig. 60: Stability studies of PC8 conjugate **11** in the presence of 1 mM GSH, in 1x DPBS pH 7.4. Source data are provided as a Source Data file.

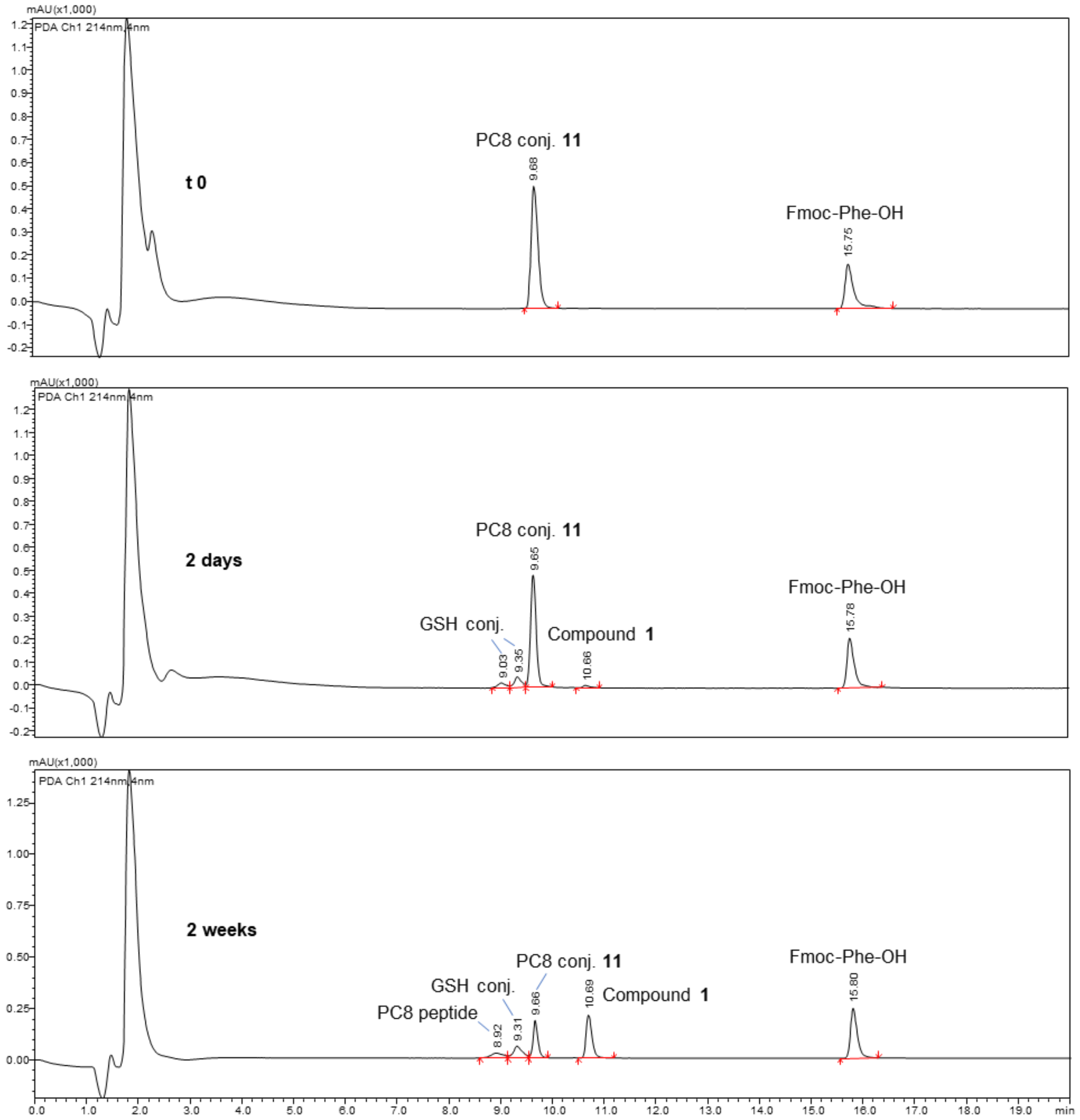

Supplementary Fig. 61: Representative chromatograms for PC8 conjugate **11** stability in presence of 1 mM GSH in 1x DPBS pH 7.4, at t_0 (top), after 2 days and 2 weeks (top to bottom) incubation at 25 °C.

PC8 maleimide conjugate (100 µg, 0.081 µmol) and GSH (30 µL, 0.200 µmol, 1 mM) were dissolved in 1x DPBS, pH 7.4. Fmoc-Phe-OH (0.025 mg/mL) was also added as an internal standard. The resultant solution was incubated for 2 weeks. 20 µL of each solution was injected into analytic HPLC (Atlantis T3 column (100 Å, 4.6 × 100 mm, 5 µm) with a flow rate of 1 mL/min with acetonitrile (solvent A, containing 0.1% *v/v* TFA) and Milli-Q water (solvent B, containing 0.1% *v/v* TFA). Mobile phase t = 0 min, 5% B; t = 15 min, 50%; t = 17 min, 100% B; t = 19 min, 100% B; t = 22 min, 5%, t = 26 min, 5%, stop to evaluate PC8 conjugate stability. The stability study was determined in triplicate based on the peaks: PC8 maleimide conjugate (RT 10.46 min), hydrolyzed PC8 maleimide conjugates (RT 10.3 and 10.4 min) and GSH maleimide conjugates (RT 11.01 min) were normalized to the IS (RT 15.8 min) peak area at 214 nm.

Stability of PC8-maleimide conjugate in 1 mM GSH

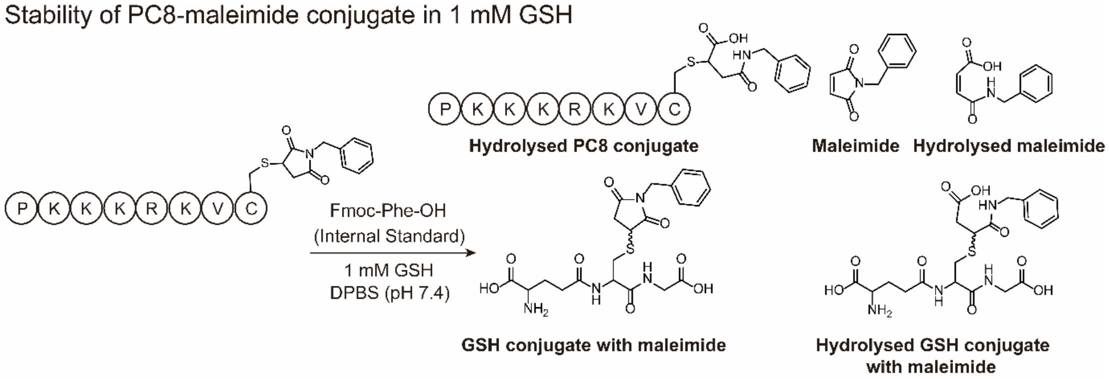

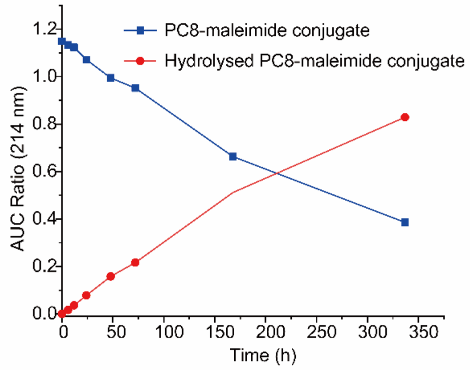

t (h)	Percentage of PC8 conjugate remained
0	100%
6	99%
12	98%
24	93%
48	87%
72	83%
7 days	58%
14 days	34%

Supplementary Fig. 62: Stability studies of PC8-maleimide conjugate in the presence of 1 mM GSH, in 1x DPBS pH 7.4. Source data are provided as a Source Data file.

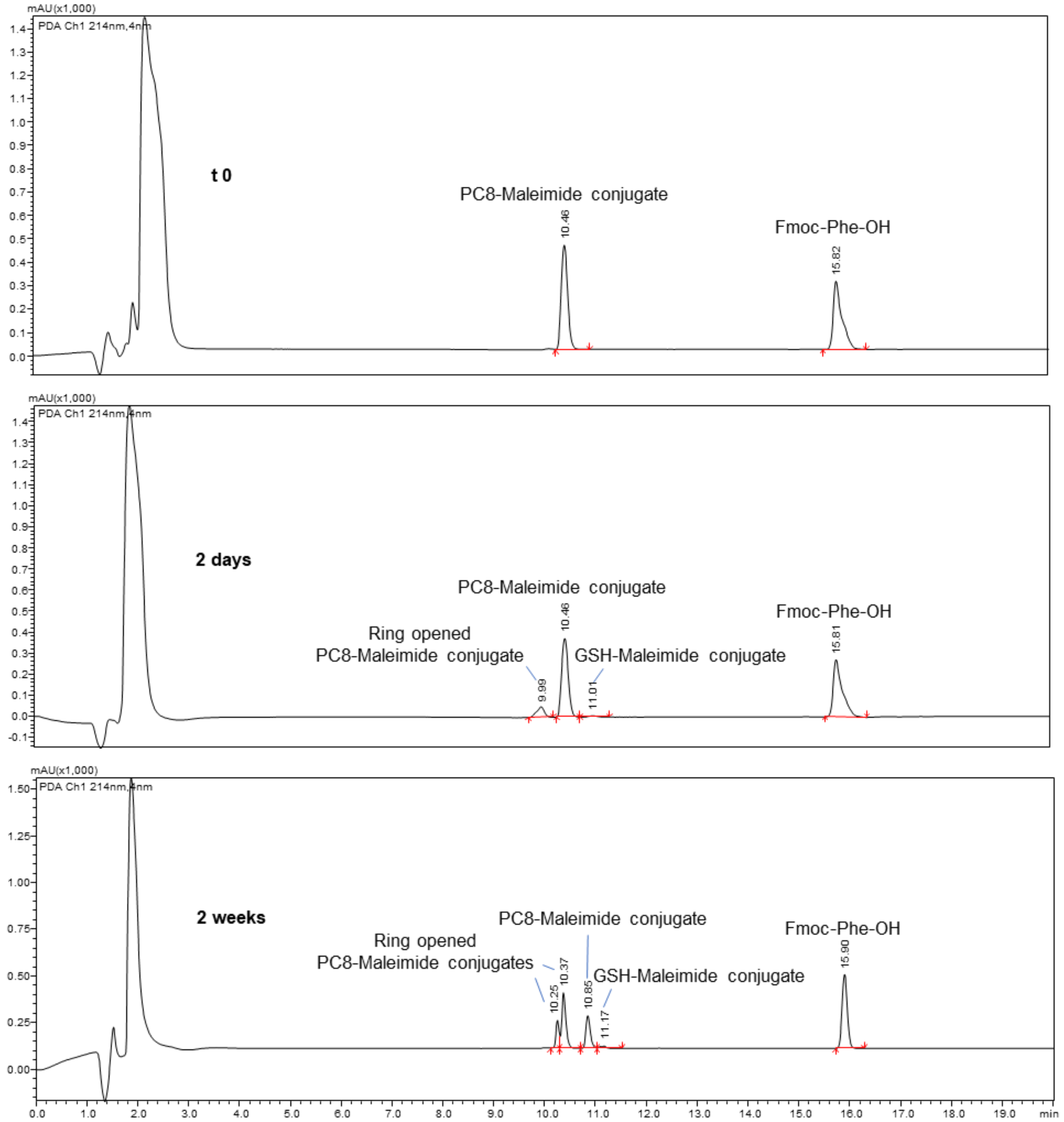

Supplementary Fig. 63: Representative chromatograms for PC8 maleimide conjugate stability in presence of 1 mM GSH in 1x DPBS pH 7.4, at t_0 (top), after 2 days and 2 weeks (top to bottom) incubation at 25 °C.

8.3 Site-selective modification of PC8 peptide and cysteine masking with 4-DPS

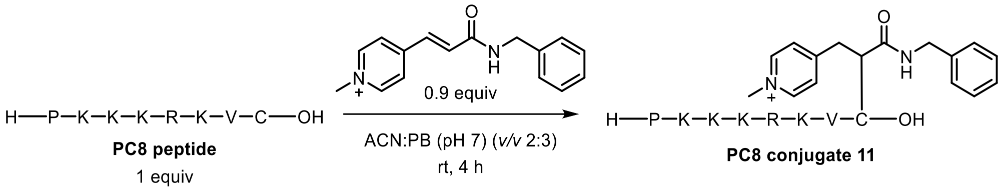

Supplementary Fig. 64: PC8 peptide modification with compound **1**.

PC8 peptide was selected as a model peptide substrate for modification. Since it contains four lysine residues in its sequence, it is a good option to check the chemoselectivity of *N*-alkylpyridinium derivatives on the peptide level. Specially, PC8 peptide (50 µg, 0.051 µmol) was dissolved in 2214 µL ACN:PB (50 mM, pH 7) = 2:3 mixture. Compound **1** (14.2 µg, 0.057 µmol) was added and the resultant mixture was incubated for 4 hours at room temperature. Next, 10 µL of the reaction mixture was injected to the HPLC using Zorbax Eclipse XDB-C18 HPLC column (80 Å, 9.4 × 250 mm, 5 µm) at a flow rate of 4 mL/min with acetonitrile (solvent A, containing 0.1% *v/v* TFA) and Milli-Q water (solvent B, containing 0.1% *v/v* TFA) mobile phase t = 0 min, 5% B; t = 15 min, 100%; t = 17 min, 100% B; t = 19 min, 5% B; t = 20 min, 5%, stop. HPLC trace of the crude reaction mixture indicated that only conjugate **11** was formed without any other multi-modification products showing excellent modification efficiency and chemoselectivity.

4,4'-Dithiodipyridine is a disulfide-containing compound that is often used for thiol quantification. It reacts with thiols quantitatively in a thiol-disulfide exchange reaction.

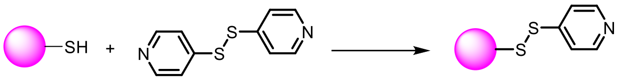

Supplementary Fig. 65: Reaction between thiol-containing substrates with 4-DPS.

In order to check if the reaction is chemoselective to thiol groups, PC8 peptide first reacted with 4-DPS to mask the cysteine residues in the peptide sequence. Specifically, PC8 peptide (50 µg, 0.051 µmol) was dissolved in ACN:PB (50 mM, pH 7) = 2:3 mixture followed by the addition of 4-DPS (22.3 µg. 0.10 µmol). After 4 h at rt, 4-DPS modified PC8 was purified to afford 4-DPS modified PC8. Then, 4-DPS modified PC8 (50ug, 0.045 µmol) was incubated with compound **1** (23 µg, 0.091 µmol) for another 4h. After that, 10 µL of the reaction mixture was injected into the HPLC and the data analysis indicated there is no further reaction observed between the masked PC8 peptide and compound **1**.

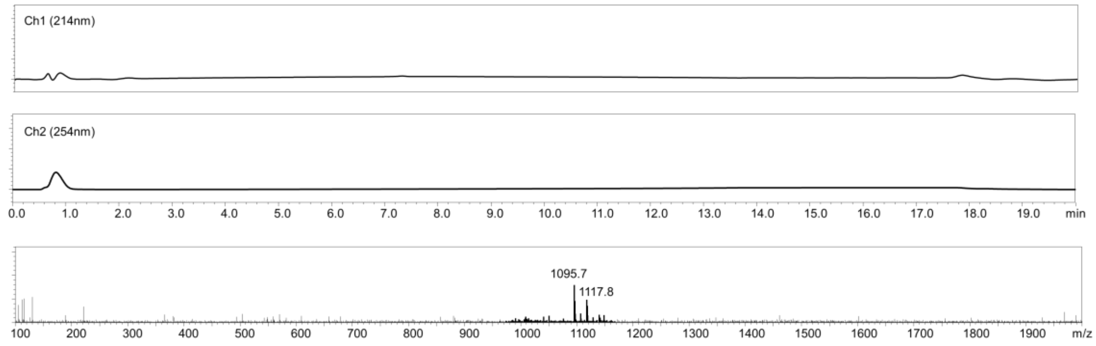

Supplementary Fig. 66: LC-MS of the 4-DPS masked PC8 peptide (calculated: 1095.6 $[M+H]^+$, 1117.6 $[M+Na]^+$; found: 1095.7 $[M+H]^+$, 1117.8 $[M+Na]^+$).

8.4 Comparison of the modification specificity of Tet peptide with using compound 1 and maleimide.

Maleimide reagents are one of the most commonly used reagents for protein modification. However, it is reported that maleimide reagents suffer from side reactions with amino groups when a large excess of maleimides is required to achieve efficient modification of proteins. Here, we compared the reaction specificity of *N*-alkylpyridinium reagents and maleimide reagents when reacting with cysteine residues. Tet peptide was chosen as the model substrate for evaluation since it contains a cysteine and lysine residue in its sequence. Tet peptide (50 µg, 0.023 µmol, 1 equiv) was dissolved in 50 µL ACN:PB (50 mM, pH 7.4) mixture in an Eppendorf. Next, compound **1** (11.7 µg, 0.046 µmol, 2 equiv) was added and the resultant mixture was incubated overnight at rt. The same protocol was also used for the Tet peptide modification with maleimides. Thereafter, the mixture was injected into HPLC to check the product. The data indicated that only a single modified product was observed with quantitative conversion when 2 equiv of the bioconjugation reagents were used. However, when increasing the compound **1** and maleimide to 10 equiv, only a single modified product was observed using compound **1** as modification reagent while both single and double modified products were observed when using maleimide as modification reagents.

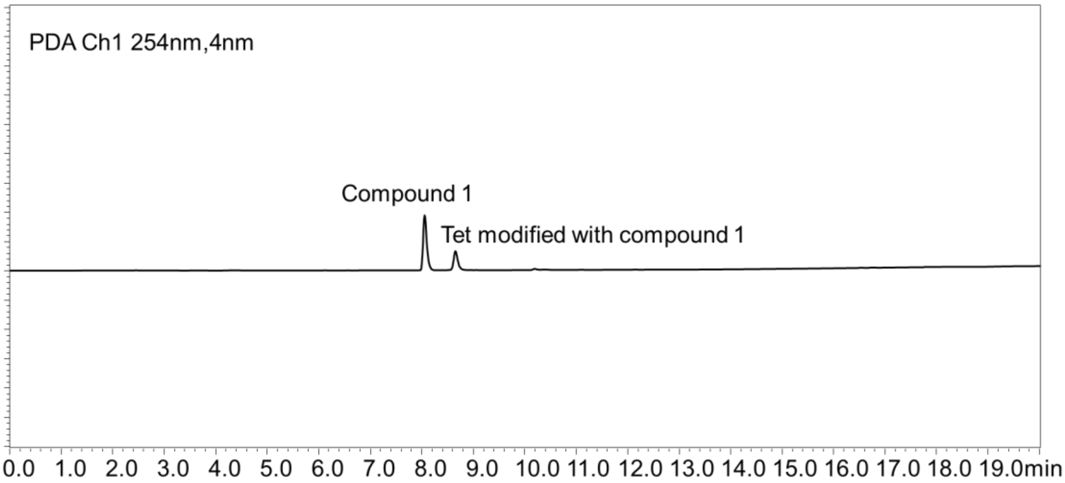

Supplementary Fig. 67: HPLC data of the reaction between Tet peptide and 2 equiv of compound **1**.

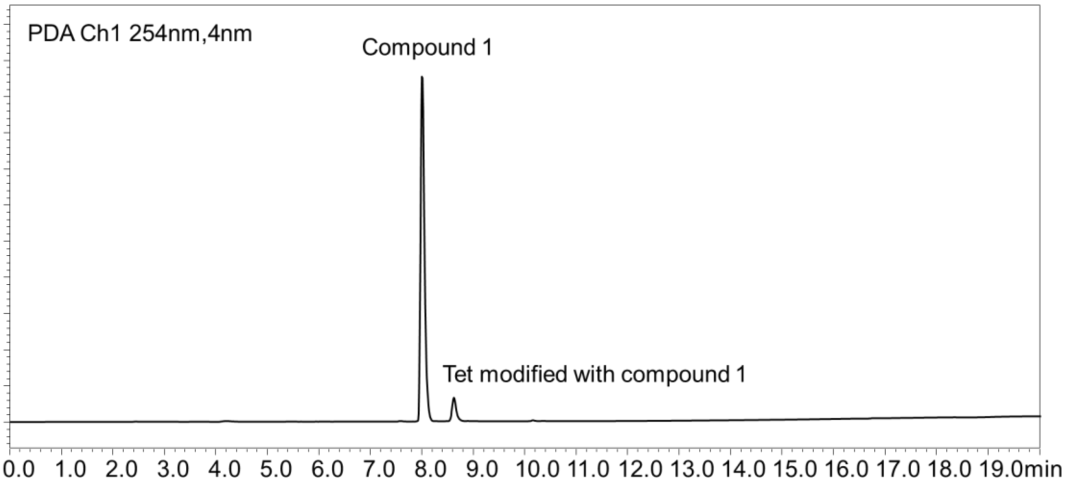

Supplementary Fig. 68: HPLC data of the reaction between Tet peptide and 10 equiv of compound **1**.

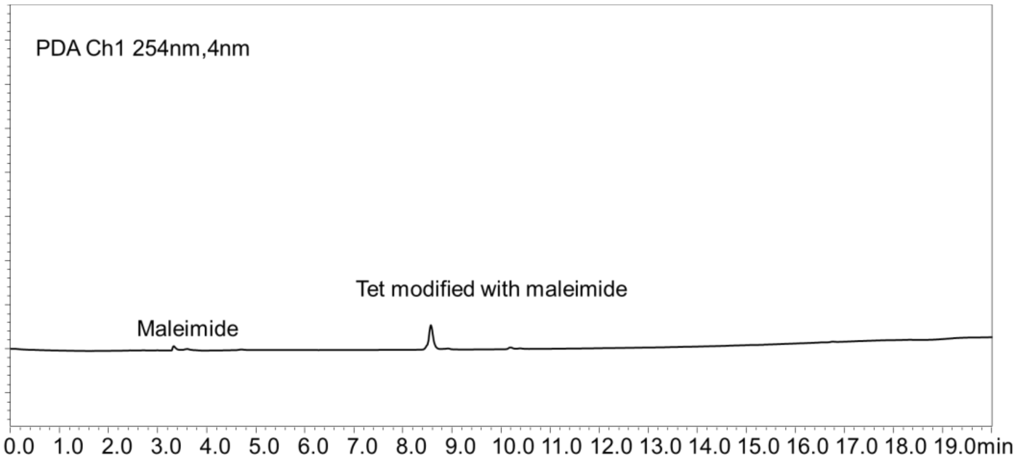

Supplementary Fig. 69: HPLC data of the reaction between Tet peptide and 2 equiv of maleimide.

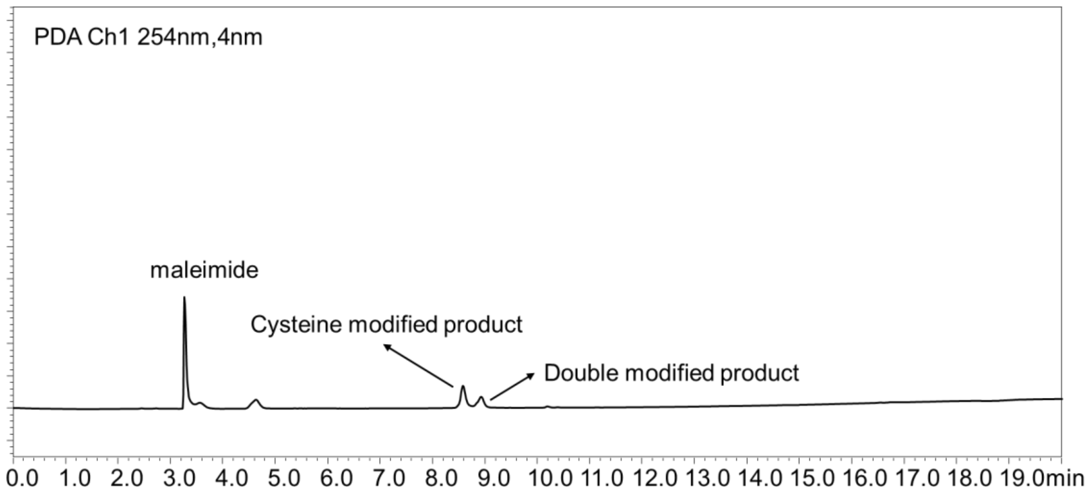

Supplementary Fig. 70: HPLC data of the reaction between Tet peptide and 10 equiv of maleimide.

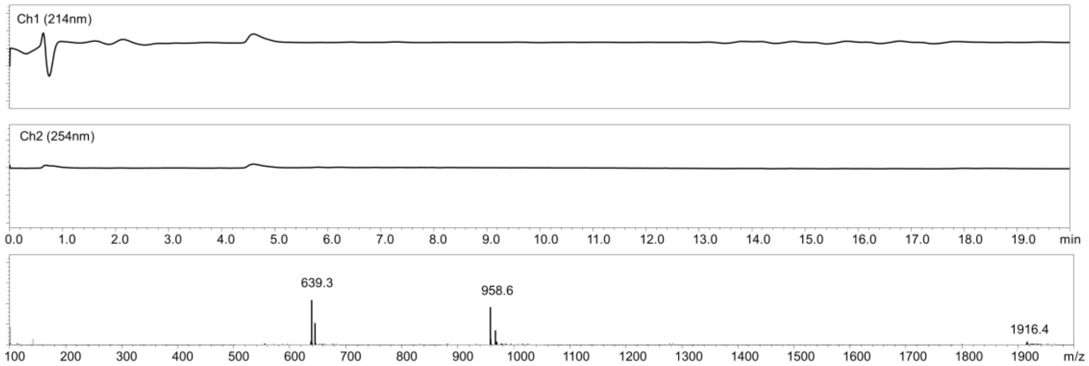

Supplementary Fig. 71: LC-MS data of the single modified Tet peptide (calculated: 1916.1 $[M]^{+}$, 958.5 $[M+H]^{2+}$, 639.3 $[M+2H]^{3+}$; found: 1916.4 $[M]^{+}$, 958.6 $[M+H]^{2+}$, 639.3 $[M+2H]^{3+}$).

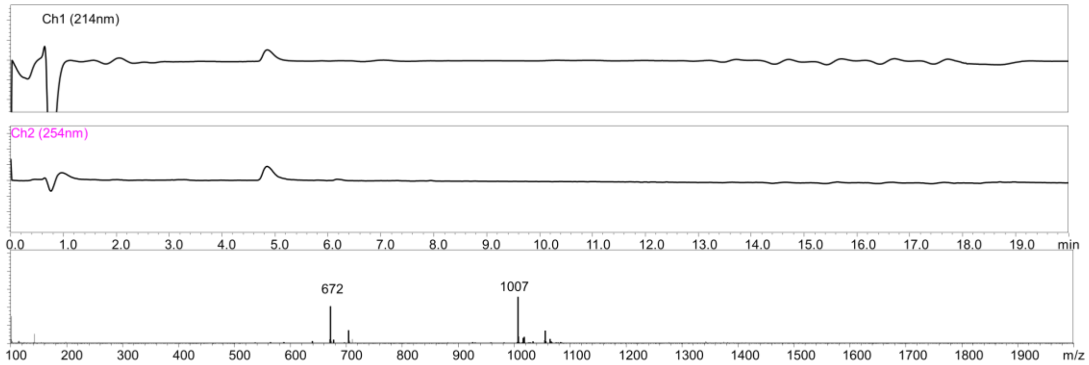

Supplementary Fig. 72: LCMS data of the double modified Tet peptide (calculated: 1007 $[M+H]^{2+}$, 672 $[M+2H]^{3+}$; found: 1007 $[M+H]^{2+}$, 672 $[M+2H]^{3+}$).

Supplementary Table 3: Comparison of the modification specificity of compound **1** and maleimide reagents when modifying Tet peptide, which contains a cysteine residue and a lysine residue in its backbone.

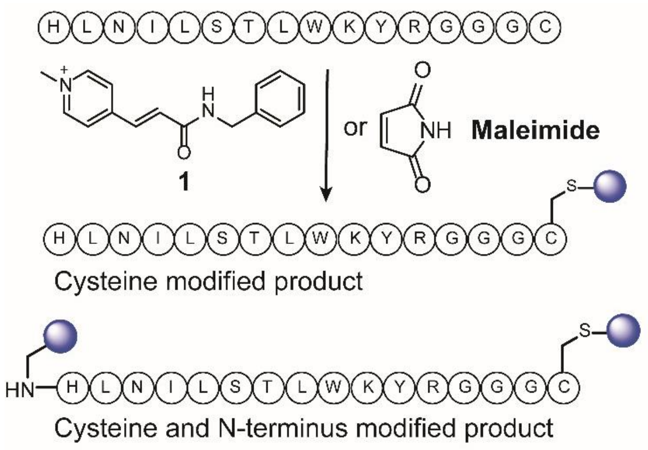

Substrate	Equiv.	Product distribution<br>Cysteine/Double-modified
1	2 eq	100% / No detected
MI	2 eq	100% / No detected
1	10 eq	100% / No detected
MI	10 eq	67% / 33%

9 Site-selective modification of proteins

Ubiquitin (Ub-K63C) was purchased from the company UbiQ as a lyophilized powder with a molecular weight of 8540 Da. The sequence of Ub-K63C: MQIFVKTLTGKTITLEVEPSDTIENVKAKIQDKEGIPPDQQRLIFAGKQLEDGRTLSDYNIQCES TLHLVLRLRGG.

Expression and Purification of Recombinant GST-Cys-C3bot1

C3 enzyme from *Clostridium botulinum* is a Rho protein inhibitor that affects and influences the intracellular Rho-mediated processes like epithelial cell migration and blood vessel formation. The cysteine mutation was inserted by site-directed mutagenesis by replacing the Alanine-1 to cysteine. The plasmid containing the GST-Cys-C3bot1 gene was transformed into *Escherichia* (*E.*) *coli* BL21 via heat shock. A single colony was then inoculated in 5 mL LB-medium (1% tryptone, 0.5% yeast extract, 1% NaCl, 100 µg/ml ampicillin) and cultured in a shaking incubator (Benchmark Scientific, Sayreville, NJ, USA) for 5 h at 37 °C and 180 rpm. This preculture was enlarged to 150 mL overnight-preculture in LB-medium incubated under the same conditions. From this second preculture, 140 mL were used to inoculate 4 L of the main culture in LB-medium which was grown to an OD_{600} of 0.6 to 0.8 at 37 °C and 180 rpm. After induction of protein expression with 0.5 mM isopropyl-d-1-thiogalactopyranoside (IPTG, Carl Roth, Karlsruhe, Germany) the main culture was incubated overnight (~18 h) at 29 °C and 180 rpm. The *E. coli* cells were harvested by centrifugation at 5500 rcf and 4 °C for 10 min and resuspended in 40 mL lysis buffer (10 mM NaCl, 20 mM Tris, 1% Triton X-100, 1% PMSF). Cell lysis was performed with EmulsiFlex-C3 homogenizer (Avestin Inc, Ottawa, Canada) at 1500 bar. Insoluble fragments were removed by centrifugation at 13,000 rcf at 4 °C for 30 min. The supernatant was filtered with 0.45 µm and 0.2 µm syringe filters and incubated overnight at 4 °C with 1.2 mL Protino Glutathione Agarose 4B-beads (Macherey-Nagel, Düren, Germany) equilibrated in PBS (137 mM NaCl, 2.7 mM KCl, 8 mM Na_2HPO_4, and 1.8 mM KH_2PO_4; pH 7.4). The beads were washed twice with washing buffer (150 mM NaCl, 20 mM Tris HCl; pH 7,4) and once with PBS by centrifugation at 4 °C and 3000 rcf for 5 min. To elute Cys-C3bot1 the GST-tag was cleaved by 30 NIH units thrombin (Amersham Biosciences, Little Chalfont, UK) per liter main culture for 1 h at room temperature. The beads were removed by centrifugation at 10,000 rcf for 30 sec at 4 °C and the supernatant was transferred onto 45 µL Benzamidine-Sepharose 6B-beads (GE Healthcare, Chicago, IL, USA) and incubated at room temperature for 10 min to deplete the thrombin. The benzamidine beads were removed by 30-sec centrifugation at 10,000 rcf and 4 °C. Protein concentration was determined against a BSA standard with densitometric analysis after SDS-PAGE with subsequent Coomassie staining. The Cys-C3bot1 enzyme was then further purified by ÄKTA pure™ chromatography system (Cytiva, Marlborough, MA, USA) using a Superdex™ 75 Increase 10/300 GL column (Cytiva, Marlborough, MA, USA).

The final sequence of Cys-C3bot1: GSPGISGGGGGSCYSNTYQEFTNIDQAKAWGNAQYKKY GLSKSEKEAIVSYTKSASEINGKLRQNKGVINGFPSNLIKQVELLDKSFNKMKTPENIMLFRGD

DPAYLGTEFQNTLLNSNGIINKTAFEKAKAKFLNKDRLEYGYISTSLMNVSQFAGRPIITKFKV
AKGSKAGYIDPISAFAGQLEMLLPRHSTYHIDDMRLSSDGKQIIITATMMGTAINPK

9.1 Site-selective modification of ubiquitin-K63C

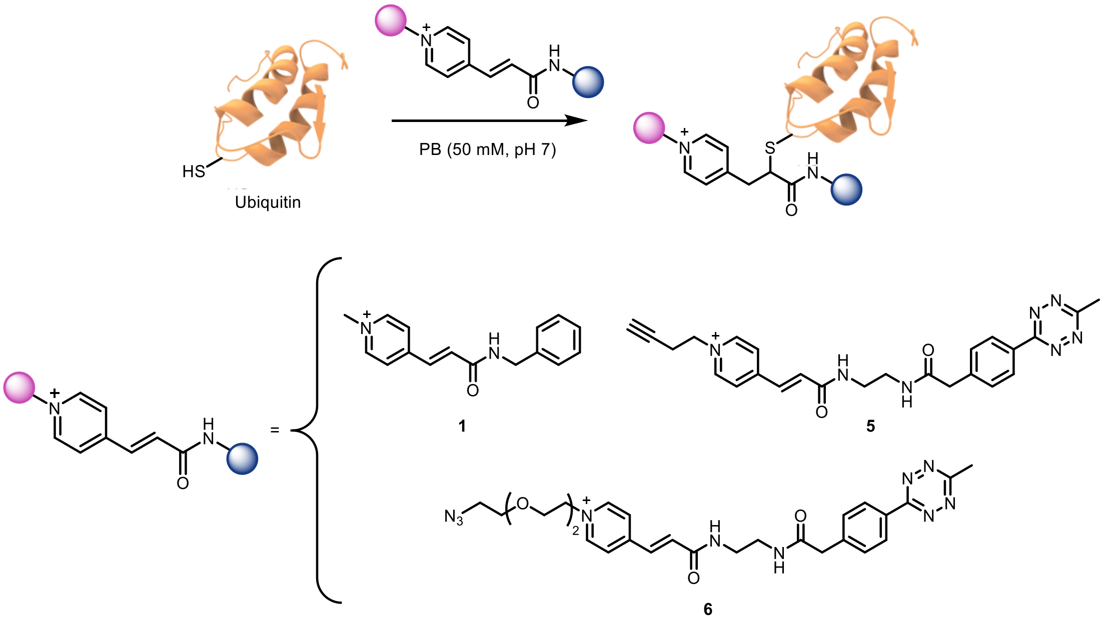

Supplementary Fig. 73: Dual modification of ubiquitin with different *N*-alkylpyridinium derivatives.

Ubiquitin (Ub-K63C), denoted as Ub, in which the lysine 63 has been mutated to a cysteine residue, was selected for modification. Ub was dissolved in DMSO to prepare a stock solution with 25 mg/mL according to the protocol from the commercial supplier. Next, Ub (80 µg, 9.37 nmol, 1 equiv) was added to an Eppendorf containing 80 µL 50 mM PB, pH 7. Then, compound **1** (21.8 µg, 74.8 nmol, 8 equiv) was also added and the resultant mixture was incubated for 4h to overnight at rt. After that, Amicon® Ultra Centrifugal Filter (3 kDa MWCO) was used to remove the excess of organic molecules and salts. ESI data indicated the successful modification of ubiquitin with compound **1**.

8 equiv of compound **5** and 35 equiv of **6** were used to ensure efficient modification of ubiquitin, considering the higher molecular weight of the reagent **6** that may have some steric hindrance when modifying complex protein substrates. Successful modifications were confirmed by ESI-HRMS shown in Fig. 6d and Supplementary Fig. 74–84.

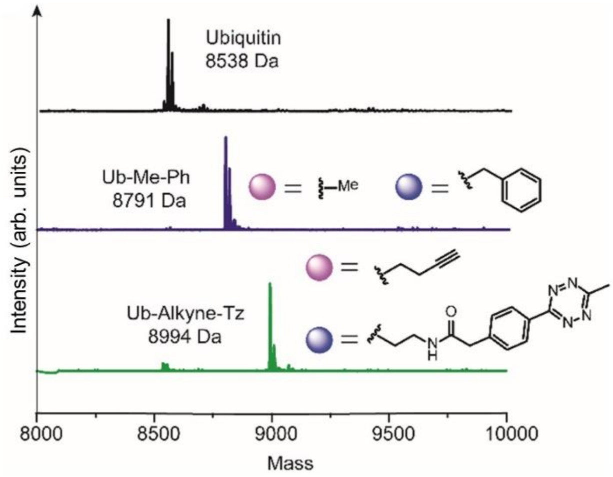

Supplementary Fig. 74: Deconvoluted ESI-MS data from the modifications of ubiquitin (K63C) with compounds **1** and **5** with MaxEnt1.

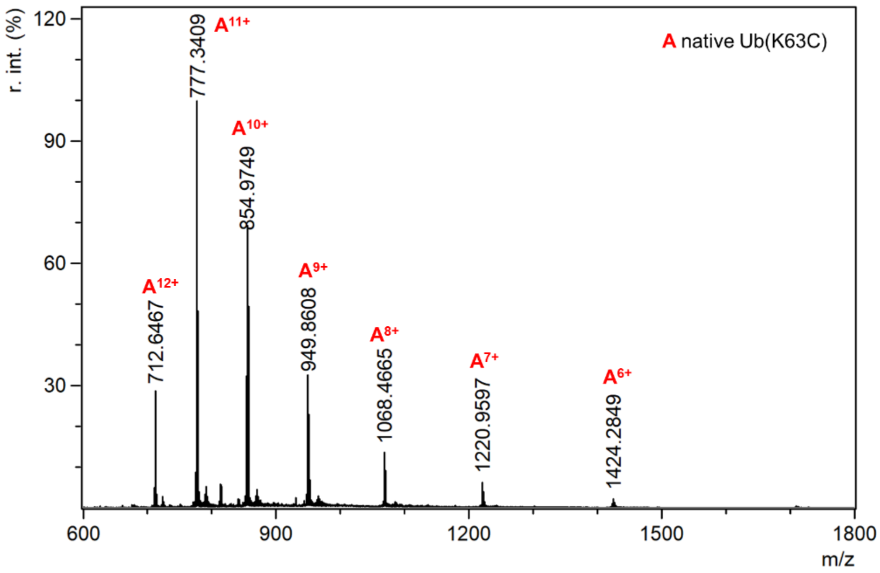

Supplementary Fig. 75: ESI-HRMS analysis from the direct infusion of desalted Ubiquitin(K63C) solution (combined ion series).

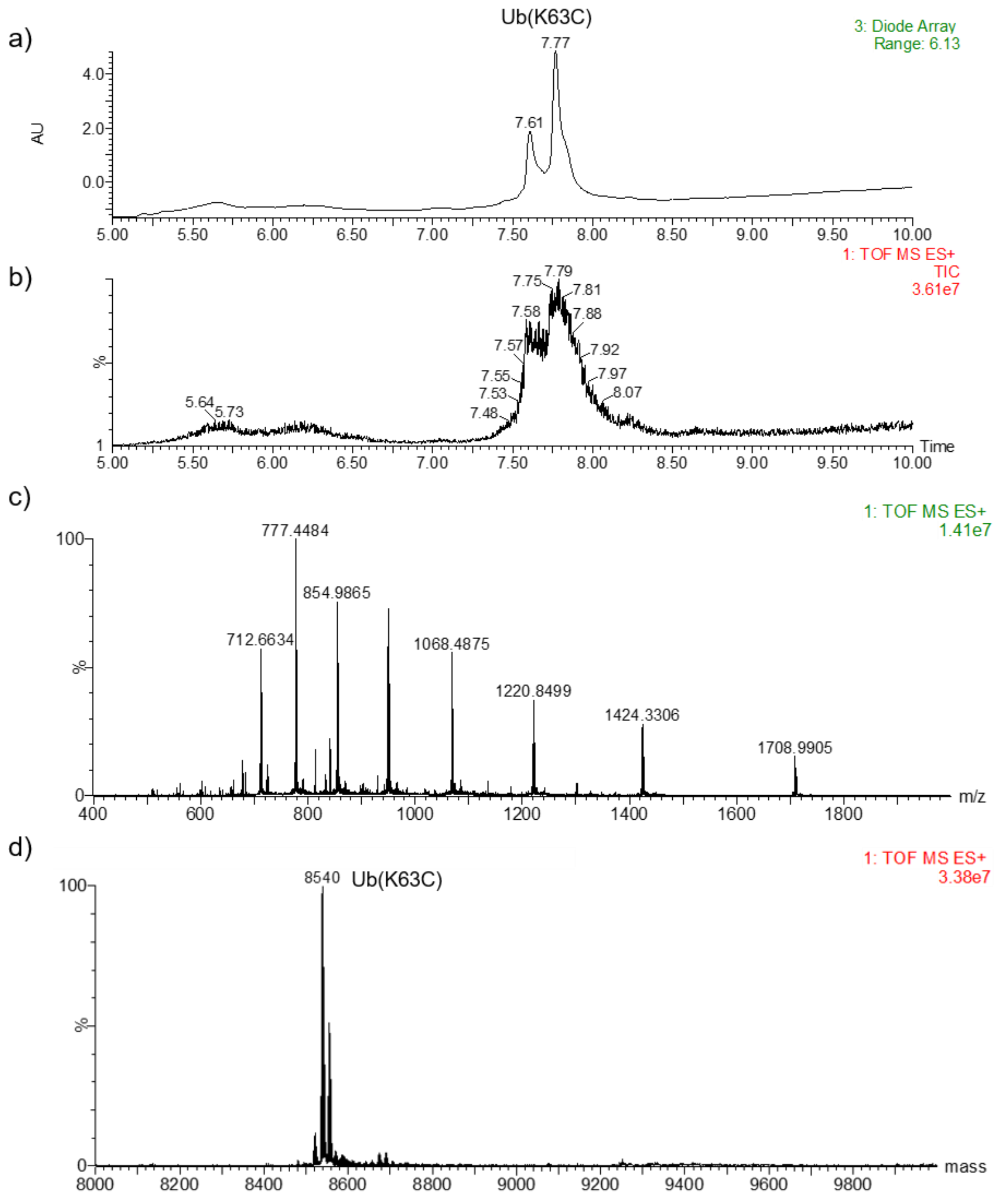

Supplementary Fig. 76: LC-ESI-HRMS analysis of commercially available Ubiquitin(K63C): a) UV/Vis chromatogram (Ubiquitin(K63C) with oxidized Met at RT 7.6 min and native Ubiquitin(K63C) at 7.8 min); b) TIC; c) combined ion series; d) deconvoluted spectrum.

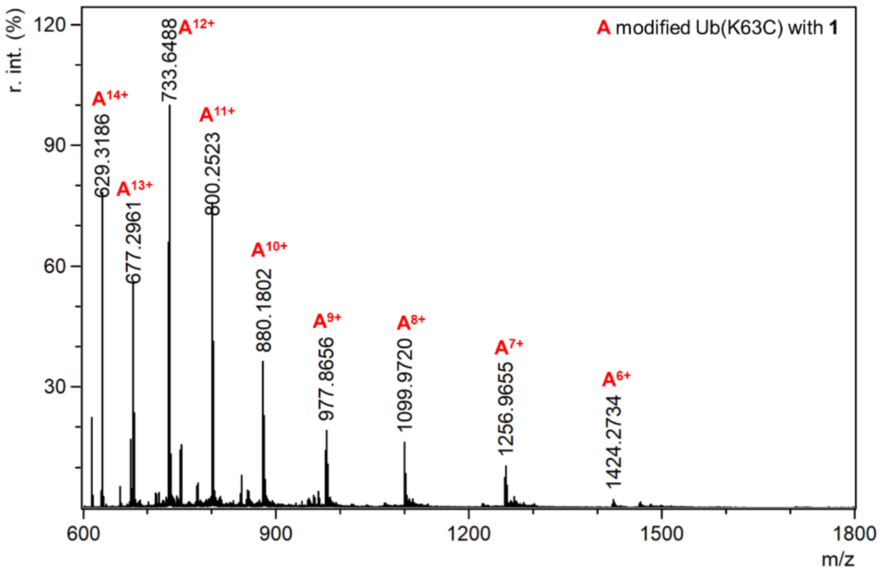

Supplementary Fig. 77: ESI-HRMS analysis from the direct infusion of desalted Ubiquitin(K63C) reaction mixture with compound **1** (combined ion series).

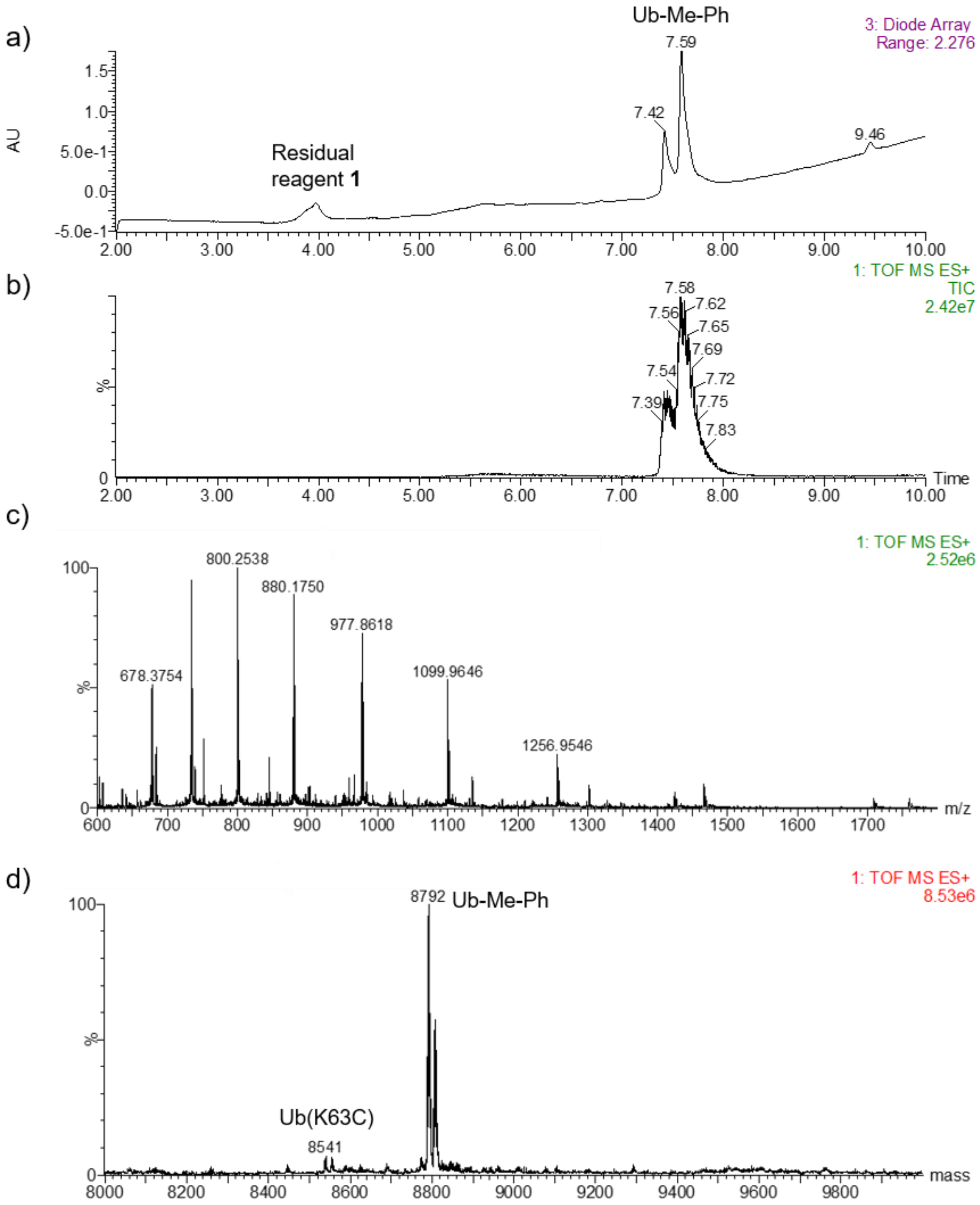

Supplementary Fig. 78: LC-ESI-HRMS analysis of Ubiquitin(K63C) modified with compound **1**: a) UV/Vis chromatogram (RT 7.4 min with oxidized Met and non-oxidized at 7.6 min); b) TIC; c) combined ion series; d) deconvoluted spectrum.

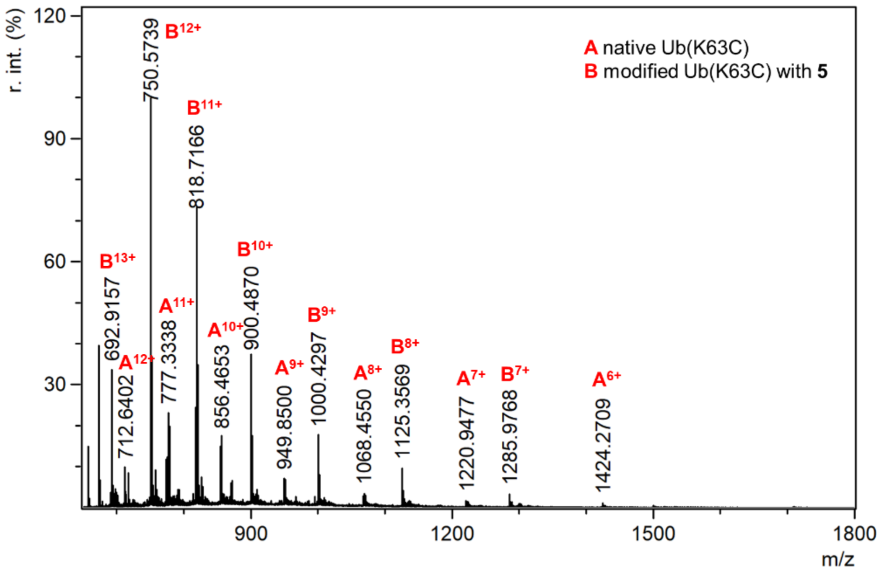

Supplementary Fig. 79: ESI-HRMS analysis from the direct infusion of desalted Ubiquitin(K63C) reaction mixture with compound **5** (combined ion series).

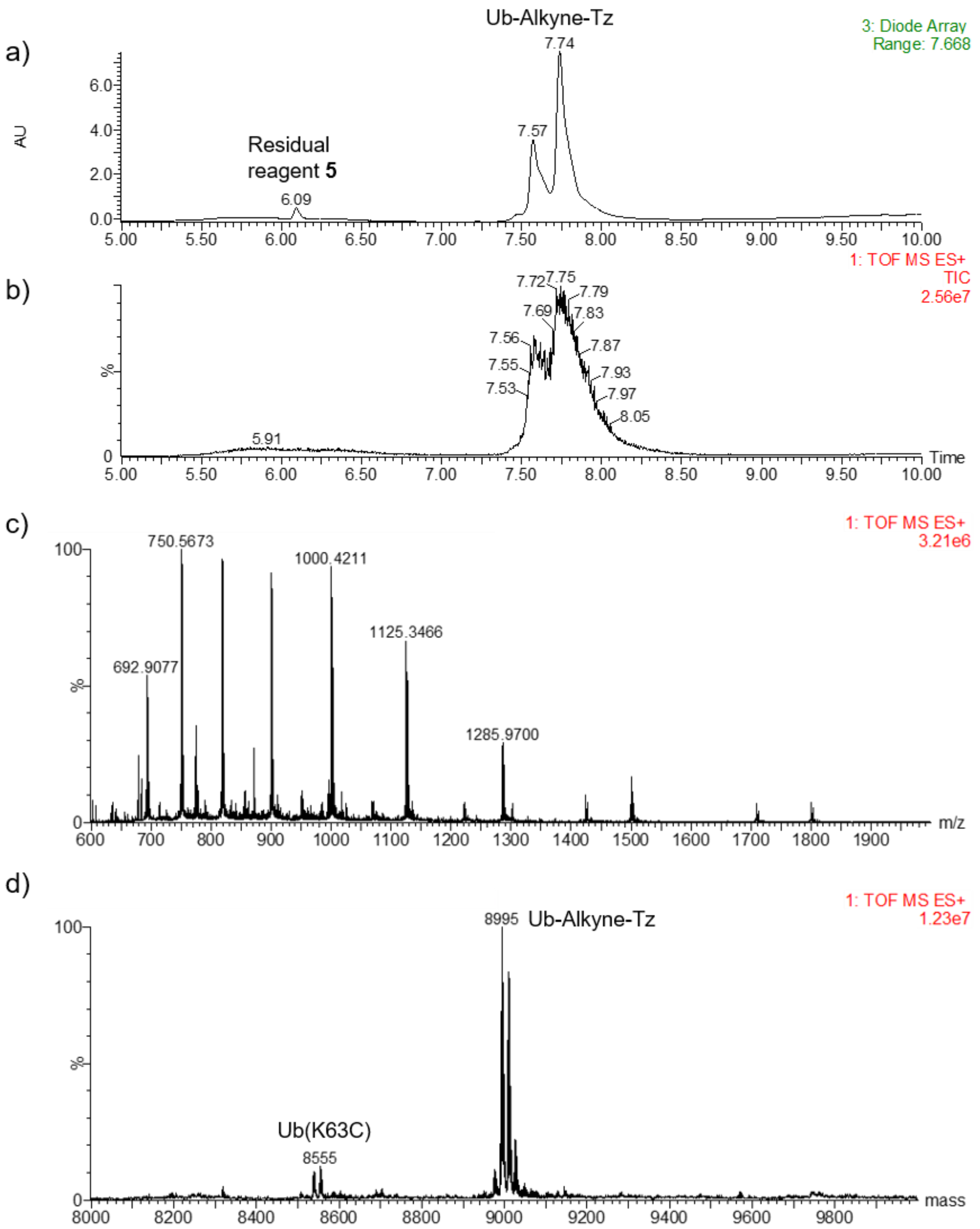

Supplementary Fig. 80: LC-ESI-HRMS analysis of Ubiquitin(K63C) modified with compound **5**: a) UV/Vis chromatogram (RT 7.6 min with oxidized Met and non-oxidized at 7.7 min); b) TIC; c) combined ion series; d) deconvoluted spectrum.

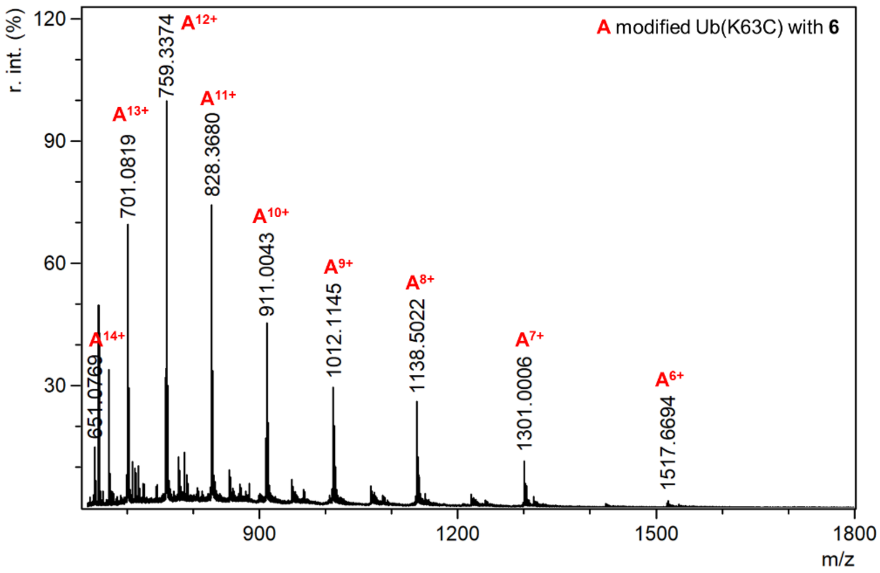

Supplementary Fig. 81: ESI-HRMS analysis from the direct infusion of desalted Ubiquitin(K63C) reaction mixture with compound **6** (combined ion series).

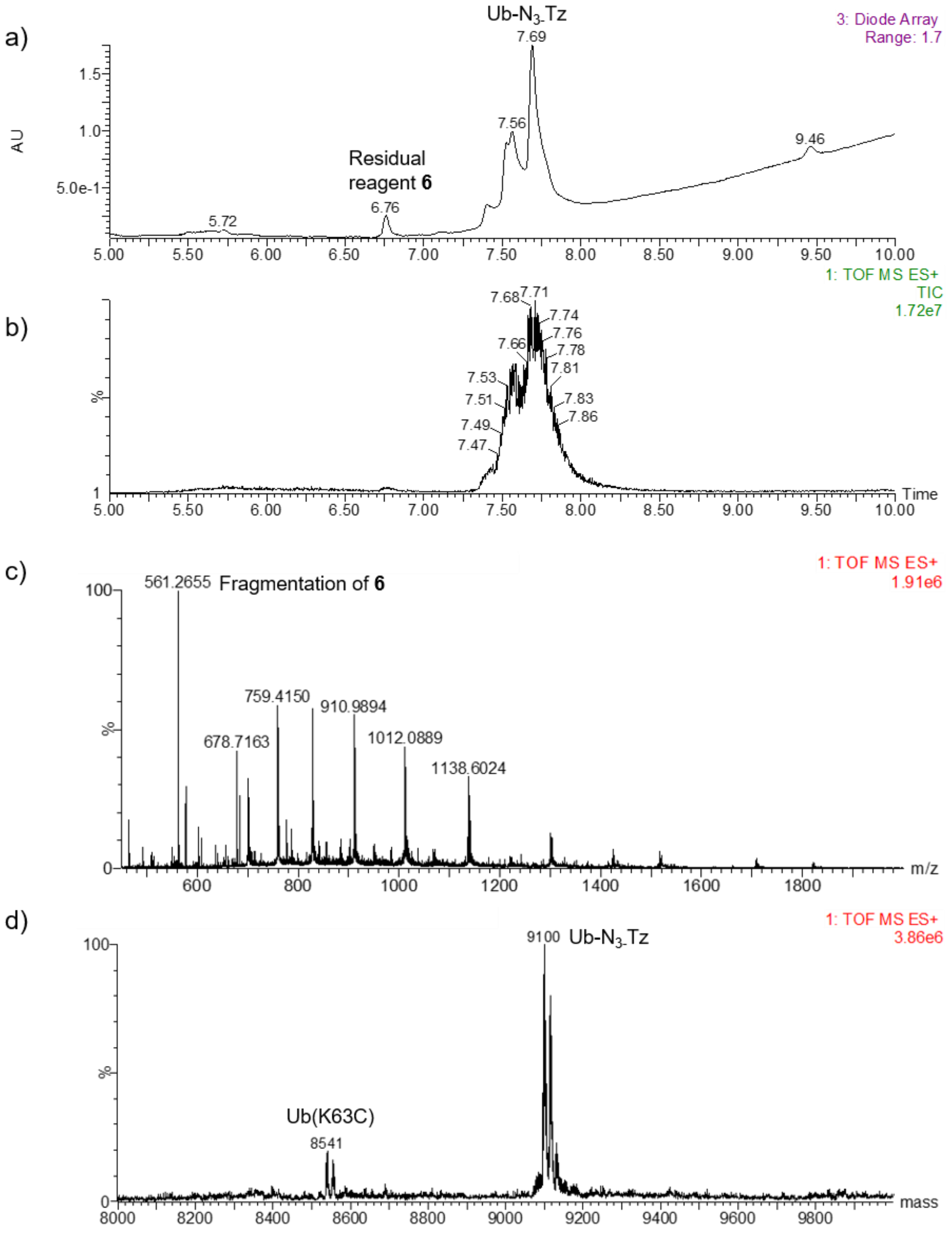

Supplementary Fig. 82: LC-ESI-HRMS analysis of Ubiquitin(K63C) modified with compound **6**: a) UV/Vis chromatogram (RT 7.6 min with oxidized Met and non-oxidized at 7.7 min); b) TIC; c) combined ion series (fragment **6**: calc. 561.6265, found: 561.2655); d) deconvoluted spectrum.

If cysteine residue ubiquitin was also masked by 4-DPS, no further reaction was observed when adding a large excess of compound **1**, using the same conditions as described above.

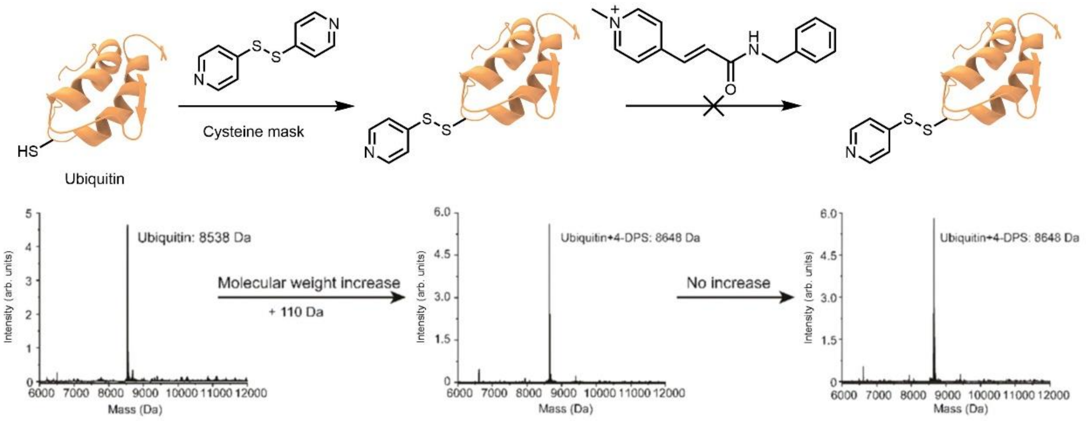

Supplementary Fig. 83: Modification of ubiquitin with 4-DPS first to mask the accessible cysteine residue followed by the addition of compound **1**.

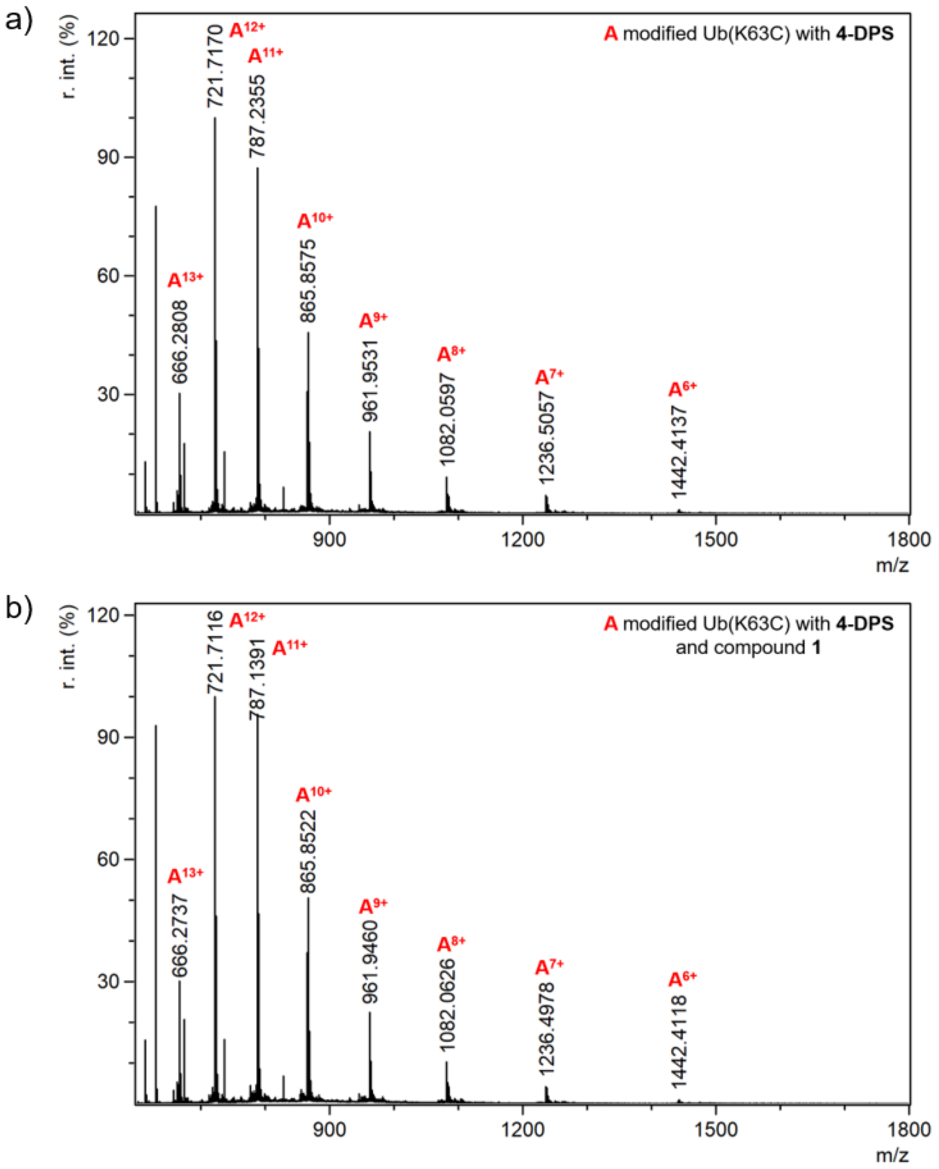

Supplementary Fig. 84: ESI-HRMS analysis from the direct infusion of desalted Ubiquitin(K63C) reaction mixture with a) 4-DPS and b) compound **1**, subsequently (combined ion series).

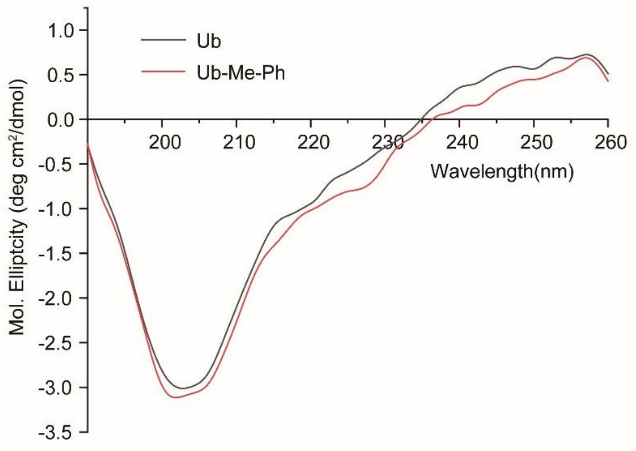

Supplementary Fig. 85: CD spectra of ubiquitin and modified ubiquitin with compound **1** from 190 nm to 260 nm.

9.2 Site-selective modification of the anti-MMR nanobody

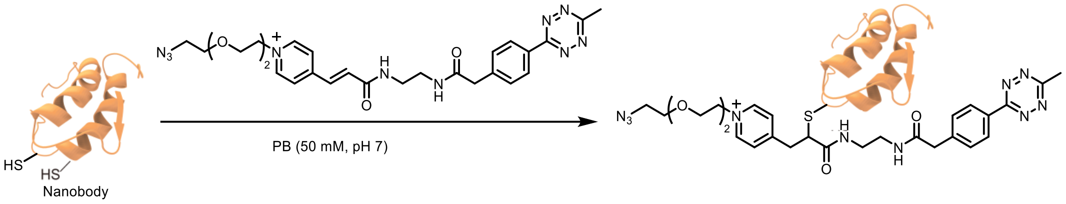

Supplementary Fig. 86: Site-selective dual modification of nanobody with compound **6**.

Nanobodies are the variable domain of heavy-chain-only antibodies that lack the Fc region. They bear the advantages of small size, high specificity, good solubility, and tissue penetration. The nanobody used in this manuscript targets the macrophage mannose receptors (MMR) that are often over-expressed on the surface of the tumor-associated macrophages. The expression and purification of the anti-MMR nanobody were shown previously and were provided by Prof. Lutz Nuhn.[11]

Anti-MMR nanobody (1 mg/mL in 50 mM PB buffer, pH 7) (15 µL, 0.10 nmol, 1 equiv) was reduced with TCEP (1 mg/mL in MQ water) (20 equiv, 5.7 µL, 2.00 nmol) at rt for 1h. Then, compound **6** (25.0 mg/mL in DMF) (40 equiv, 0.90 µL, 4.00 nmol) was added to the nanobody solution and the resultant mixture was incubated overnight at rt. After that, the reaction mixture was purified by using an Amicon® Ultra Centrifugal Filter (3 kDa MWCO) to remove the excess compound **6** and organic solvent with using water as an exchange solvent. ESI-MS data indicated the successful modification of the anti-MMR nanobody with compound **6**.

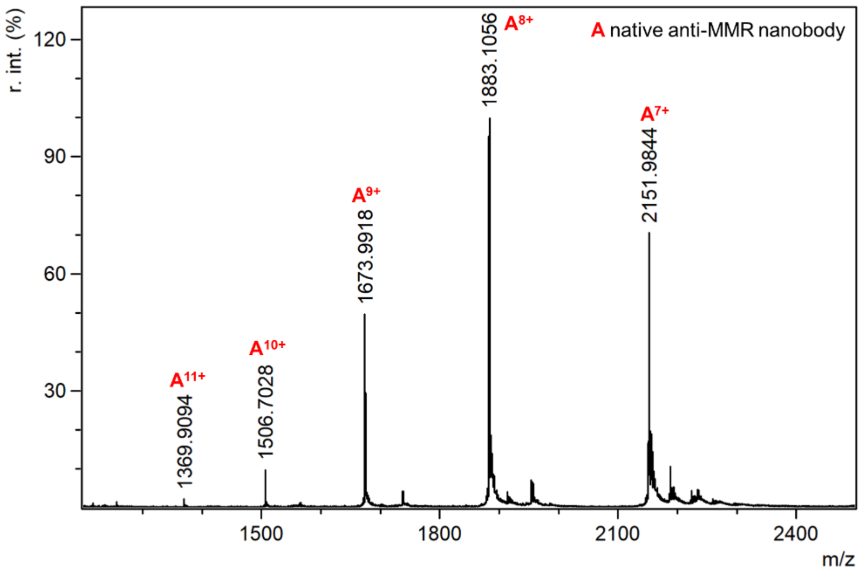

Supplementary Fig. 87: ESI-HRMS analysis from the direct infusion of reduced (with 20 equiv TCEP) and desalted anti-MMR nanobody (combined ion series).

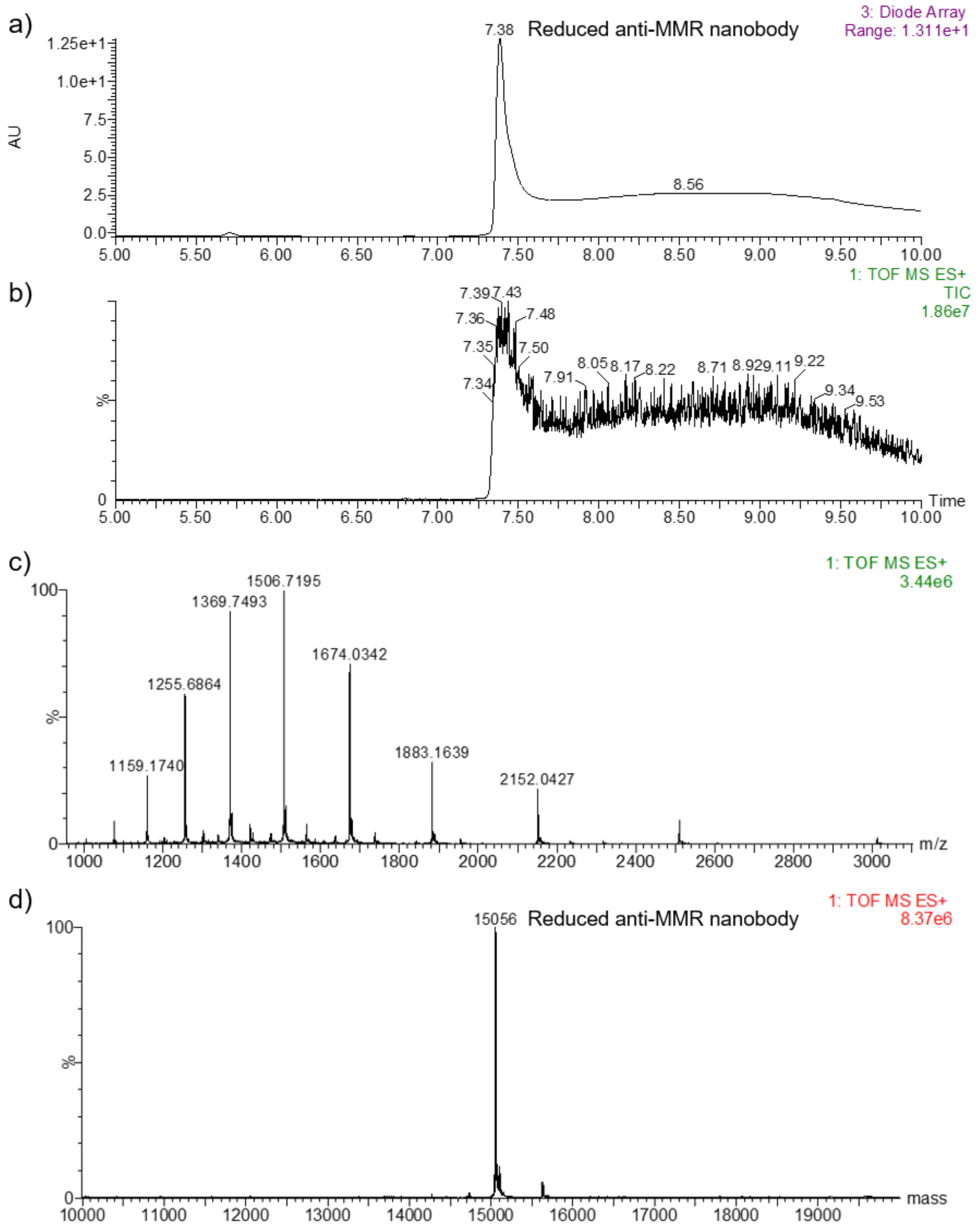

Supplementary Fig. 88: LC-ESI-HRMS analysis of reduced anti-MMR nanobody: a) UV/Vis chromatogram and TIC; b) combined ion series; c) deconvoluted spectrum.

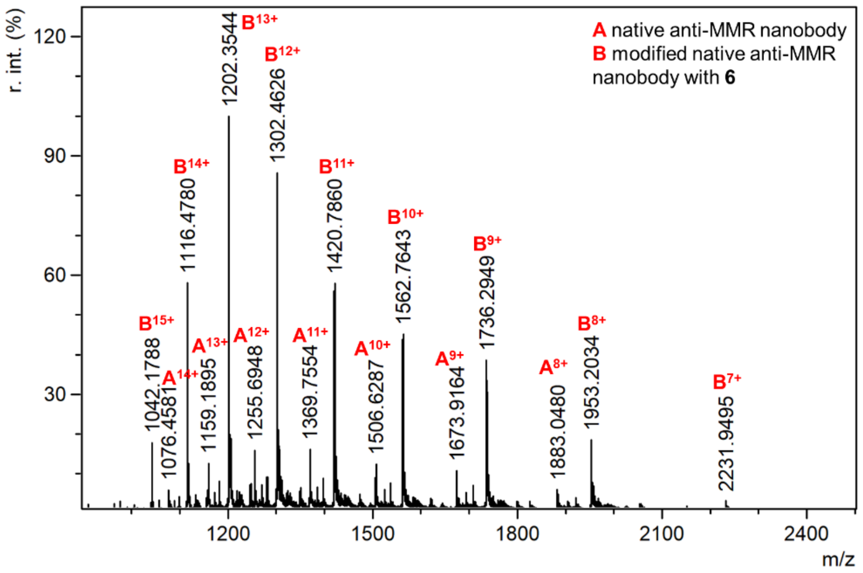

Supplementary Fig. 89: ESI-HRMS analysis from the direct infusion of desalted anti-MMR nanobody reaction mixture with compound **6** (combined ion series).

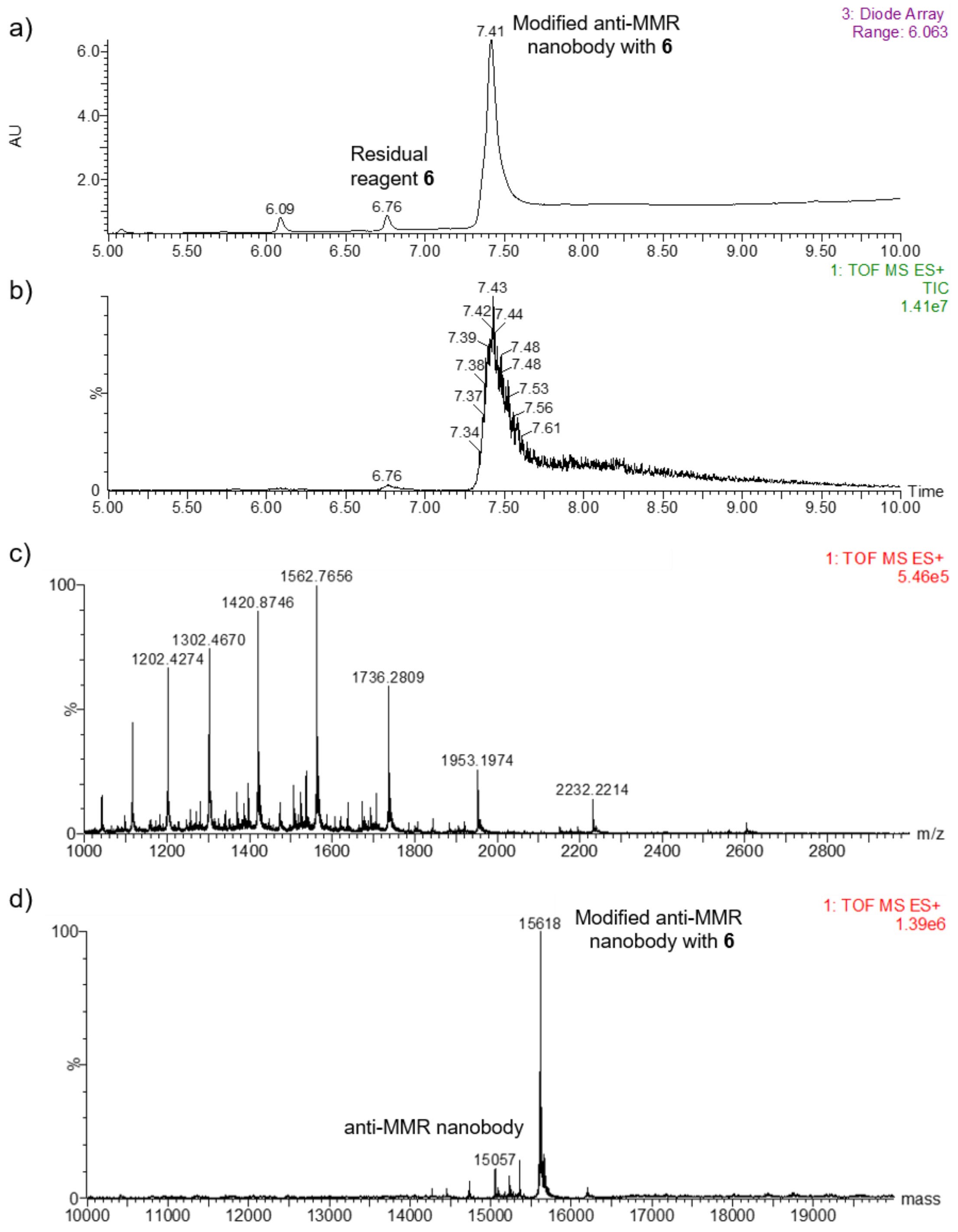

Supplementary Fig. 90: LC-ESI-HRMS analysis of anti-MMR nanobody modified with compound **6**: a) UV/Vis chromatogram and TIC; b) combined ion series; c) deconvoluted spectrum.

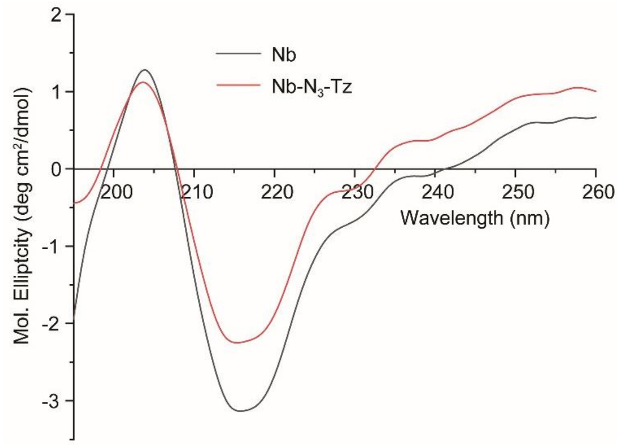

Supplementary Fig. 91: CD spectra of anti-MMR nanobody and modified nanobody with compound **6** from 190 nm to 260 nm.

9.3 Modification of trastuzumab

The used antibody here is a biosimilar of the HER-2 (Trastuzumab) Recombinant Human Monoclonal Antibody (clone 4D5-8) from Leinco Technologies (catalog number LT1500).

(Heavy chain)

EVQLVESGGGLVQPGGSLRLSCAASGFNIKDTYIHWVRQAPGKGLEWVARIYPTNGYTRYA
DSVKGRFTISADTSKNTAYLQMNSLRAEDTAVYYCSRWGGDGFYAMDYWGQGTLVTVSSA
STKGPSVFPLAPSSKSTSGGTAALGCLVKDYFPEPVTVSWNSGALTSGVHTFPAVLQSSGL
YSLSSVVTVPSSSLGTQTYICNVNHKPSNTKVDKKVEPKSCDKTHTCPPCPAPELLGGPSVF
LFPPKPKDTLMISRTPEVTCVVVDVSHEDPEVKFNWYVDGVEVHNAKTKPREEQYNSTYRV
VSVLTVLHQDWLNGKEYKCKVSNKALPAPIEKTISKAKGQPREPQVYTLPPSREEMTKNQVS
LTCLVKGFYPSDIAVEWESNGQPENNYKTTPPVLDSDGSFFLYSKLTVDKSRWQQGNVFSC
SVMHEALHNHYTQKSLSLSPG

(Light chain)

DIQMTQSPSSLSASVGDRVTITCRASQDVNTAVAWYQQKPGKAPKLLIYSASFLYSGVPSRF
SGSRSGTDFTLTISSLQPEDFATYYCQQHYTTPPTFGQGTKVEIKRTVAAPSVFIFPPSDEQL
KSGTASVVCLLNNFYPREAKVQWKVDNALQSGNSQESVTEQDSKDSTYSLSSTLTLSKADY
EKHKVYACEVTHQGLSSPVTKSFNRGEC

(Disulfide bridge: H22-H96, H147-H203, H264-H324, H370-H428, H229-H'229, H232-H'232, L23-L88, L134-L194, H223-L214)

9.3.1 Trastuzumab modification with reagent 6 and TCO-sulfoCy5

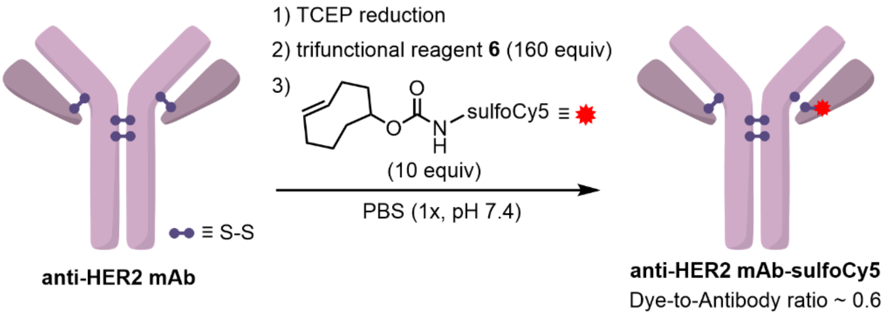

Wavelength	Abs (arb. units)	Average	Calculated conc. (μM)*
280 nm	0.601	0.619	2.07
	0.617		
	0.63		
	0.632		
	0.617		
650 nm	0.41	0.415	2.07
	0.399		
	0.426		
	0.435		
	0.404		

*concentration correction
Sample dilution factor (DF) = 1.25x
ε_{Cy5} = 250000 $M^{-1}cm^{-1}$
SulfoCy5 dye correction factor at 280 nm (CF) = 0.03
$\varepsilon_{IgG(280nm)}$ = 210000 $M^{-1}cm^{-1}$

$$\text{Degree of labelling of anti-HER2 mAb-sulfoCy5} = \frac{A_{Cy5} \times \varepsilon_{IgG(280nm)}}{(A_{280} - A_{Cy5} \times CF) \times \varepsilon_{Cy5}} = 0.57$$

Supplementary Fig. 92: Calculation of the DOL of **Anti-HER2 mAb-sulfoCy5**.

The purified Anti-HER2 mAb-sulfoCy5 conjugate afforded *via* 1,6-addition of thiols to reagent **6** and post-functionalization with TCO-sulfoCy5 was further analysed by LC-ESI-HRMS and nanodrop for degree of labelling determination. In addition, the afforded conjugate was deglycosylated with PNGaseF following the manufacturer's procedure before LC-ESI-HRMS analysis.

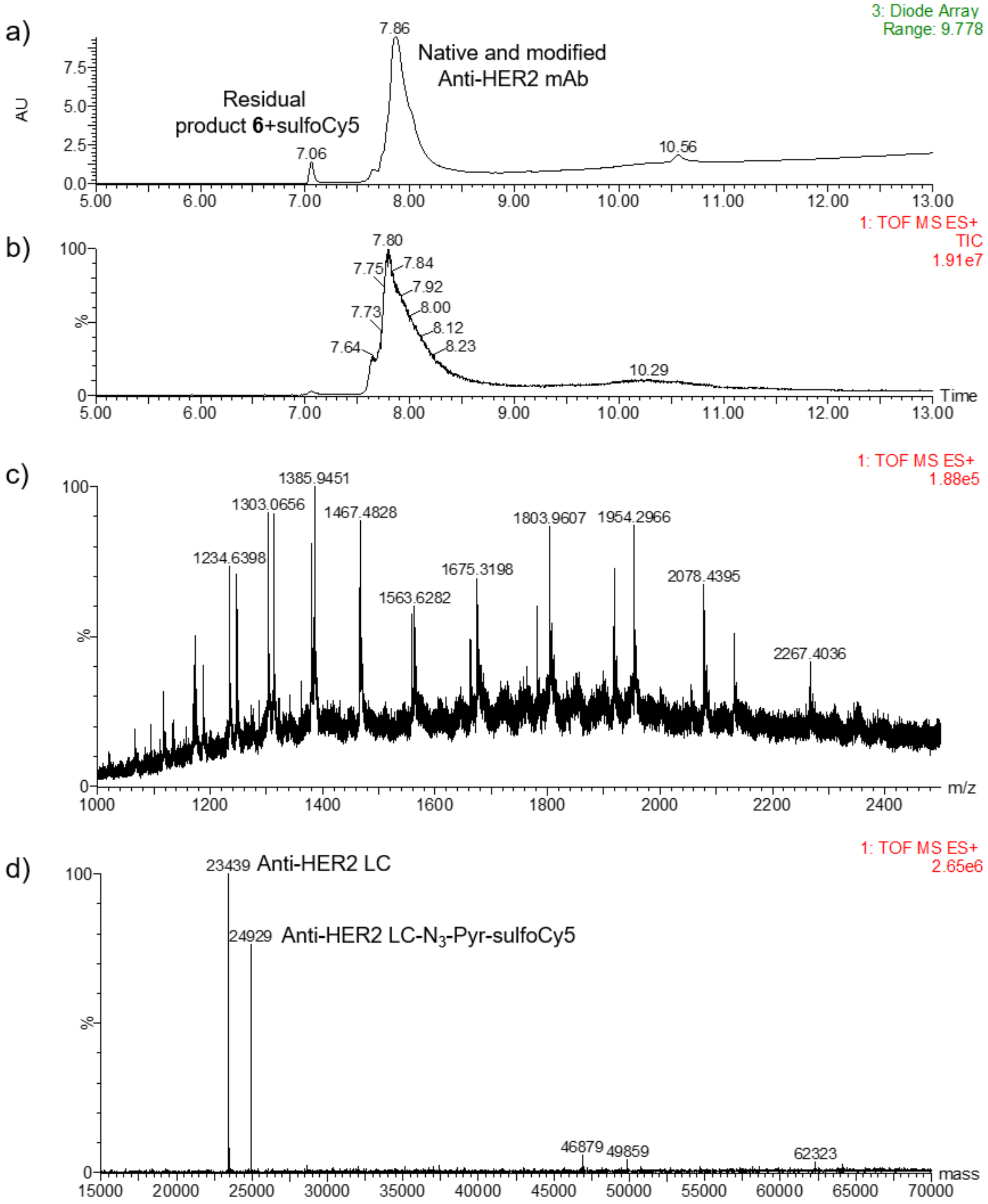

Supplementary Fig. 93: LC-ESI-HRMS analysis of anti-HER2 mAb modified with compound **6** and TCO-sulfoCy5: a) UV/Vis chromatogram (RT 7.1 min residual iEDDA product of compound **6** with TCO-sulfoCy5 and RT 7.9 min native and modified anti-HER2 mAb); b) TIC; c) combined ion series; d) deconvoluted spectrum: anti-HER2 light chain (LC) calc. 23442 Da, found 23439 Da and anti-HER2 LC-N_3-Pyr-sulfoCy5 calc. 24935 Da, found 24929 Da. The peaks at 46879 Da and 49859 Da correspond to the respective dimers.

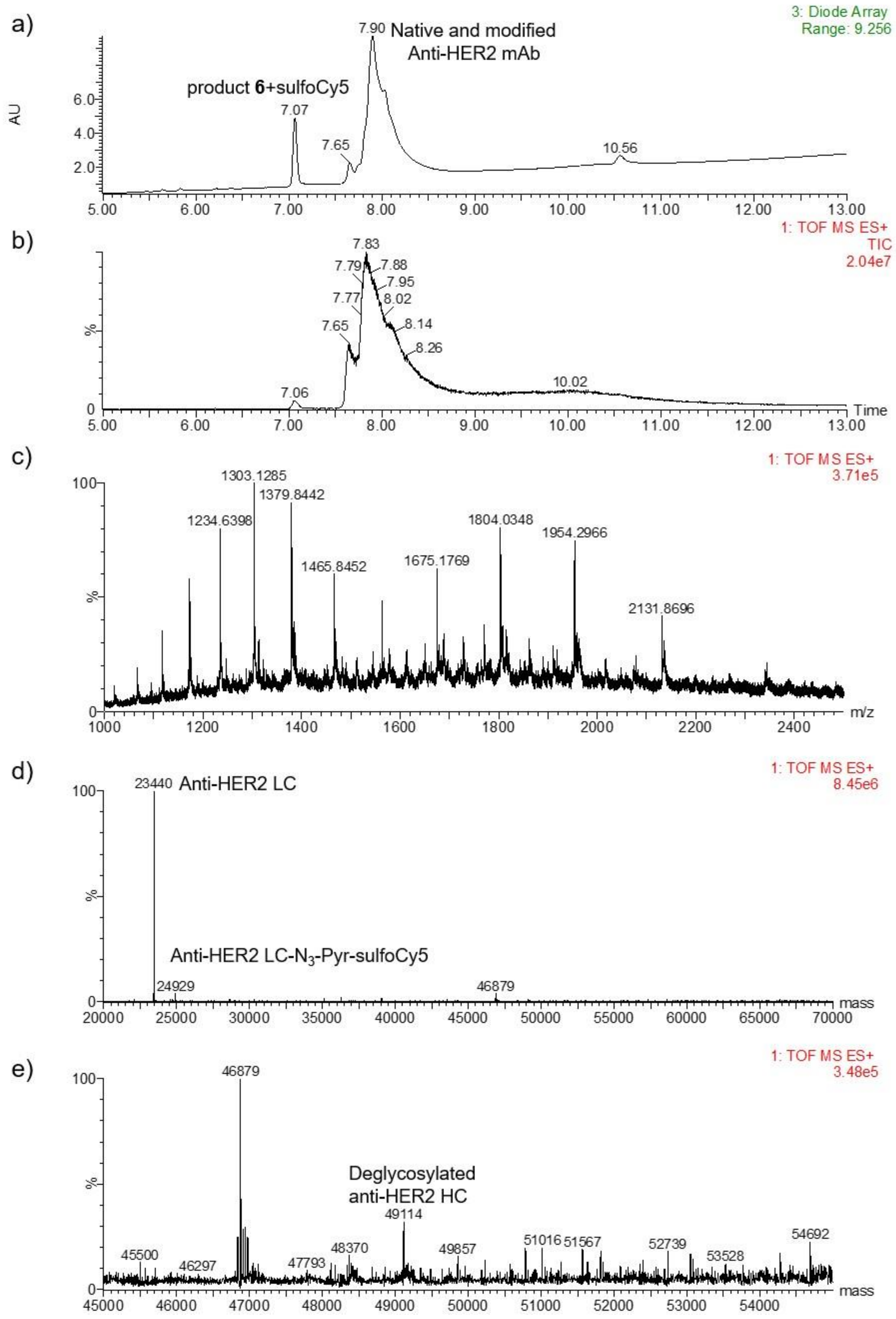

Supplementary Fig. 94: LC-ESI-HRMS analysis of deglycosylated anti-HER2 mAb conjugated with compound **6** and TCO-sulfoCy5: a) UV/Vis chromatogram (RT 7.1 min iEDDA product of compound **6** with TCO-sulfoCy5 and RT 7.9 min native and modified anti-HER2 mAb); b) TIC; c) combined ion series; d) deconvoluted spectrum: anti-HER2 light chain (LC) calc. 23442 Da, found 23440 Da and anti-HER2 LC-N_3-Pyr-sulfoCy5 calc. 24935 Da, found 24929 Da. e) zoom-in deconvoluted spectrum: deglycosylated anti-HER2 light chain (HC) calc. 49157 Da, found 49114 Da. The peak at 46879 Da correspond to the LC dimer.

9.3.2 Trastuzumab modification with Maleimide-sulfoCy5

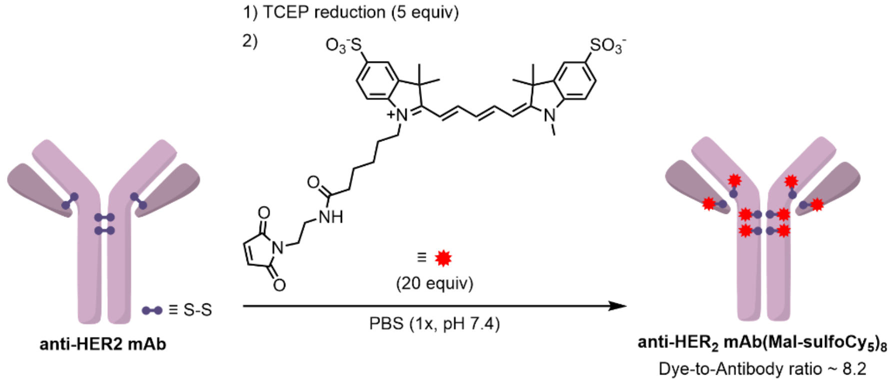

anti-HER2(4D5-8) (6.4 mg/mL in PBS 1x, pH 7.4) (6.0 µL, 0.27 nmol) was diluted to 2 mg/mL in 19.20 µL PBS 1x, pH 7.4 and TCEP (0.29 mg/mL, in 1 mM) (1.4 µL, 1.4 nmol) was added and shaken for 1h at 37 °C. Maleimide-sulfoCy5 (stock 5 mg/mL in MQ water) (0.87 µL, 5.4 nmol) was added and the resultant mixture was incubated for 4h at 37 °C. Then, the reaction mixture was transferred to ultrafiltration tube (0.5 mL, 10 kDa, PES filter) to remove the excess compound Maleimide-sulfoCy5, TCEP and organic solvent using PBS 1x, pH 7.4 as exchange solvent (6x, 12000G, 10 min) to be analysed by MALDI-ToF with sinapinic acid matrix and LC-ESI-HRMS confirm the complete cysteine functionalization and yield anti-HER2 mAb(Mal-sulfoCy5)$_8$ conjugate, as well some side reaction with amines in the light chain. In addition, the afforded conjugate was deglycosylated with PNGaseF following the manufacturer's procedure before LC-ESI-HRMS analysis.

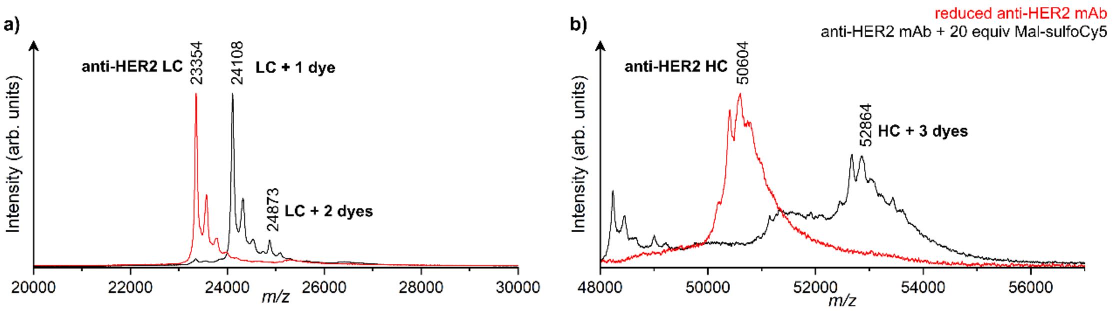

Supplementary Fig. 95: MALDI-ToF analysis of modified trastuzumab (anti-HER2 mAb) with 20 equiv of Maleimide-sulfoCy5. Light chain (anti-HER2 LC), heavy chain (anti-HER2 HC).

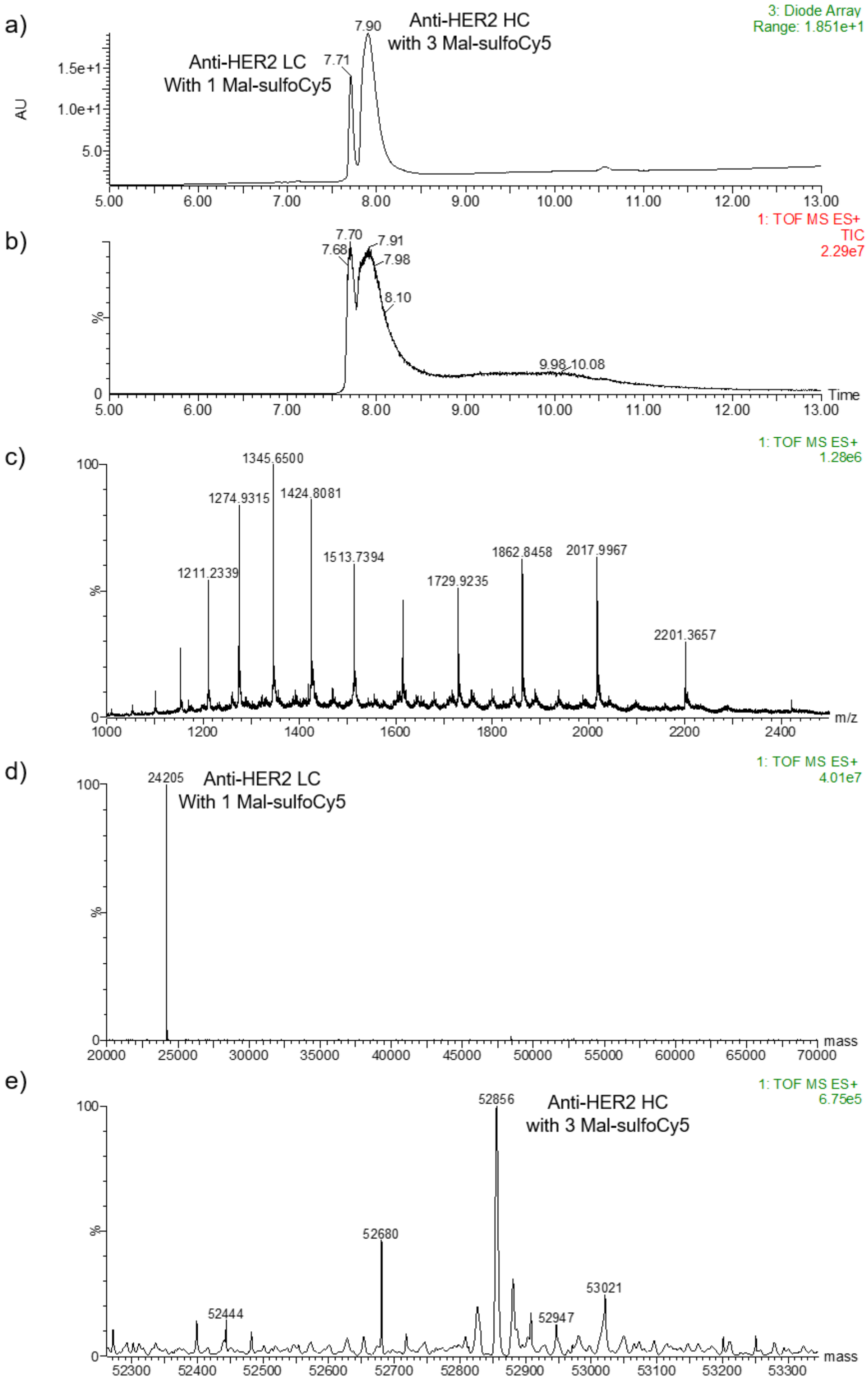

Supplementary Fig. 96: LC-ESI-HRMS analysis of anti-HER2 mAb modified with 20 equiv of maleimide-sulfoCy5: a) UV/Vis chromatogram (RT 7.7 min anti-HER2 mAb LC-Mal-sulfoCy5 and RT 7.9 min anti-HER2 mAb HC-(Mal-sulfoCy5)$_3$); b) TIC; c) combined ion series; d) deconvoluted spectrum: anti-HER2 mAb LC-Mal-sulfoCy5 calc. 24206 Da, found 24205 Da; e) zoom-in deconvoluted spectrum: anti-HER2 mAb HC-(Mal-sulfoCy5)$_3$ calc. 52911 Da, found 52856 Da. The peaks at 52680 Da and 53021 Da correspond to other glycoforms.

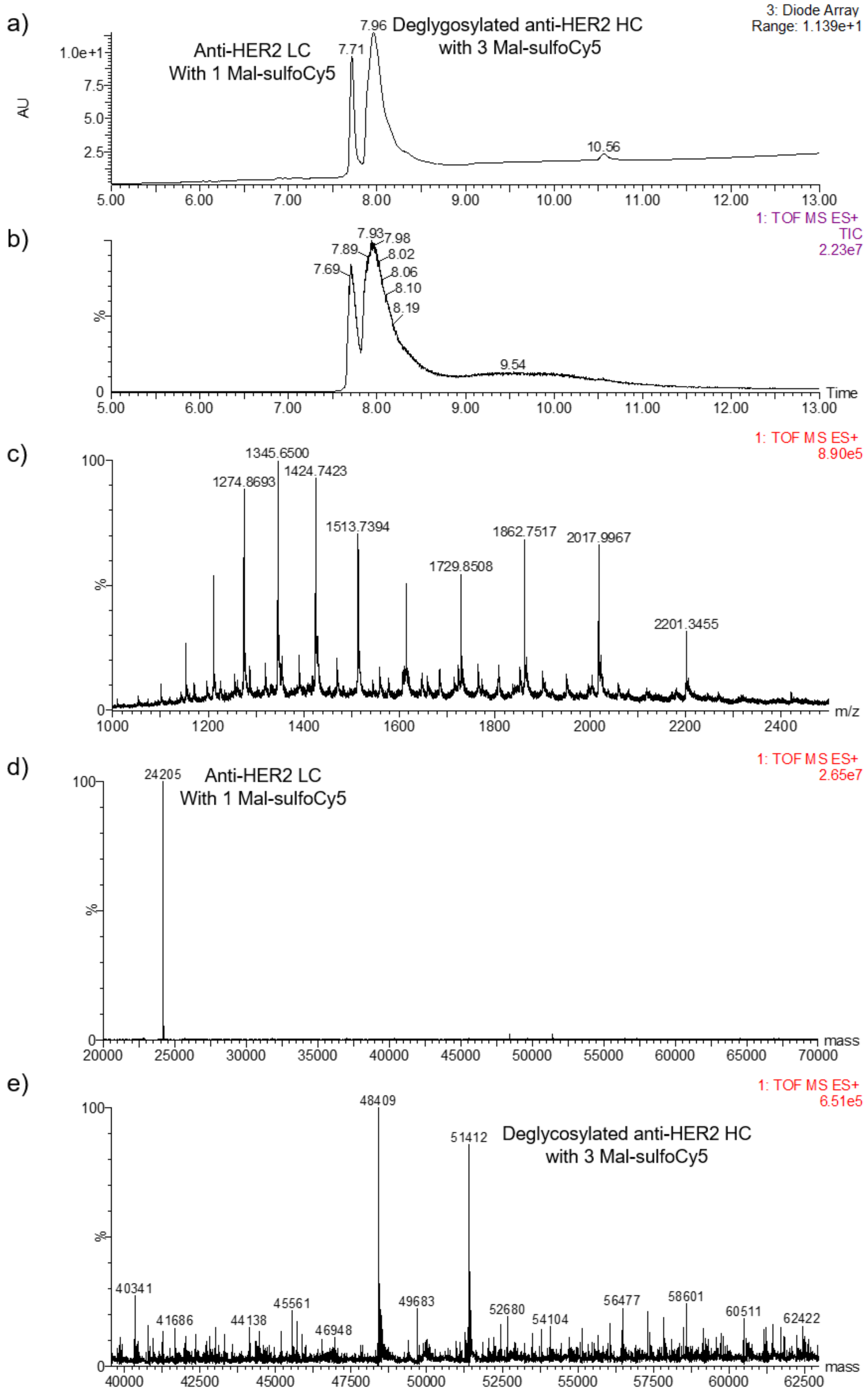

Supplementary Fig. 97: LC-ESI-HRMS analysis of deglycosylated anti-HER2 mAb(Mal-sulfoCy5)$_8$ conjugate: a) UV/Vis chromatogram (RT 7.7 min anti-HER2 mAb LC-Mal-sulfoCy5 and RT 7.9 min deglycosylated anti-HER2 mAb HC-(Mal-sulfoCy5)$_3$); b) TIC; c) combined ion series; d) deconvoluted spectrum: anti-HER2 mAb LC-Mal-sulfoCy5 calc. 24206 Da, found 24205 Da; e) zoom-in deconvoluted spectrum: anti-HER2 mAb HC-(Mal-sulfoCy5)$_3$ calc. 51449 Da, found 51412 Da. The peak at 48409 Da corresponds to the modified LC dimer.

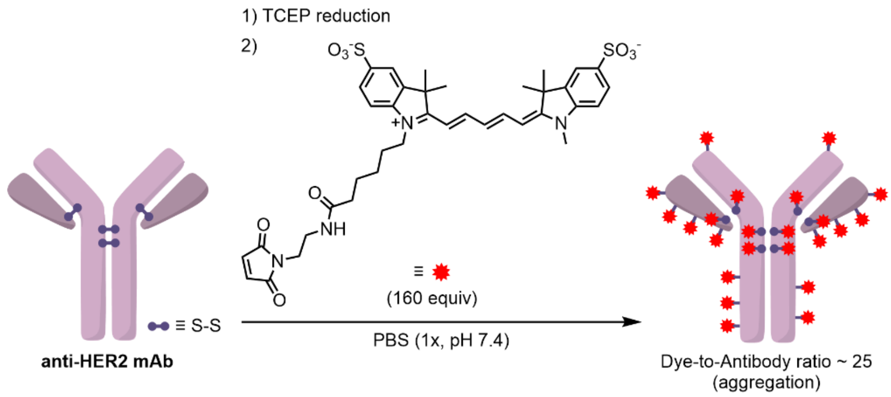

The same procedure was used to incubate anti-HER2(4D5-8) with large excess of Maleimide-sulfoCy5. A Maleimide-sulfoCy5 stock solution of 25 mg/mL in Milli-Q water was used instead (1.39 µL, 43.2 nmol) and the reaction mixture was shaken for 16h at 37 °C. The resulting reaction mixture afforded a blue precipitate, indicating the formation of insoluble aggregates. The reaction mixture was directly analysed by MALDI-ToF and show polydispersed mixture of protein conjugates with 5 dyes per light chain and a statistical dye-to-antibody ratio around 25, based on the monocharged peak of the obtained conjugates.

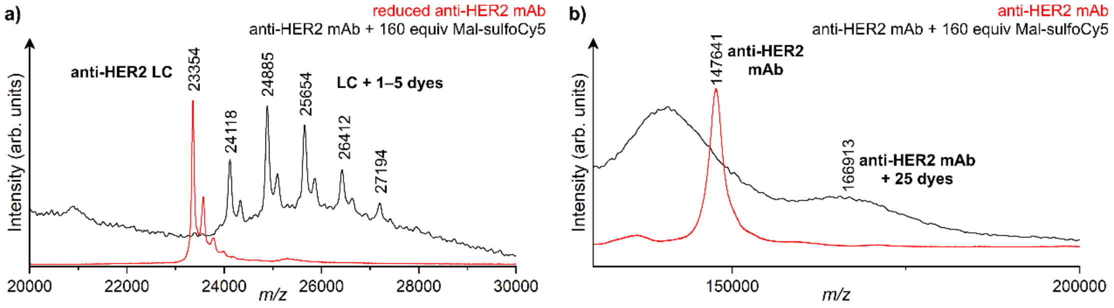

Supplementary Fig. 98: MALDI-ToF analysis of modified trastuzumab (anti-HER2 mAb) with 160 equiv of Maleimide-sulfoCy5. Light chain (anti-HER2 LC), heavy chain (anti-HER2 HC).

9.3.3 Trastuzumab reduction and deglycosylation with PNGaseF

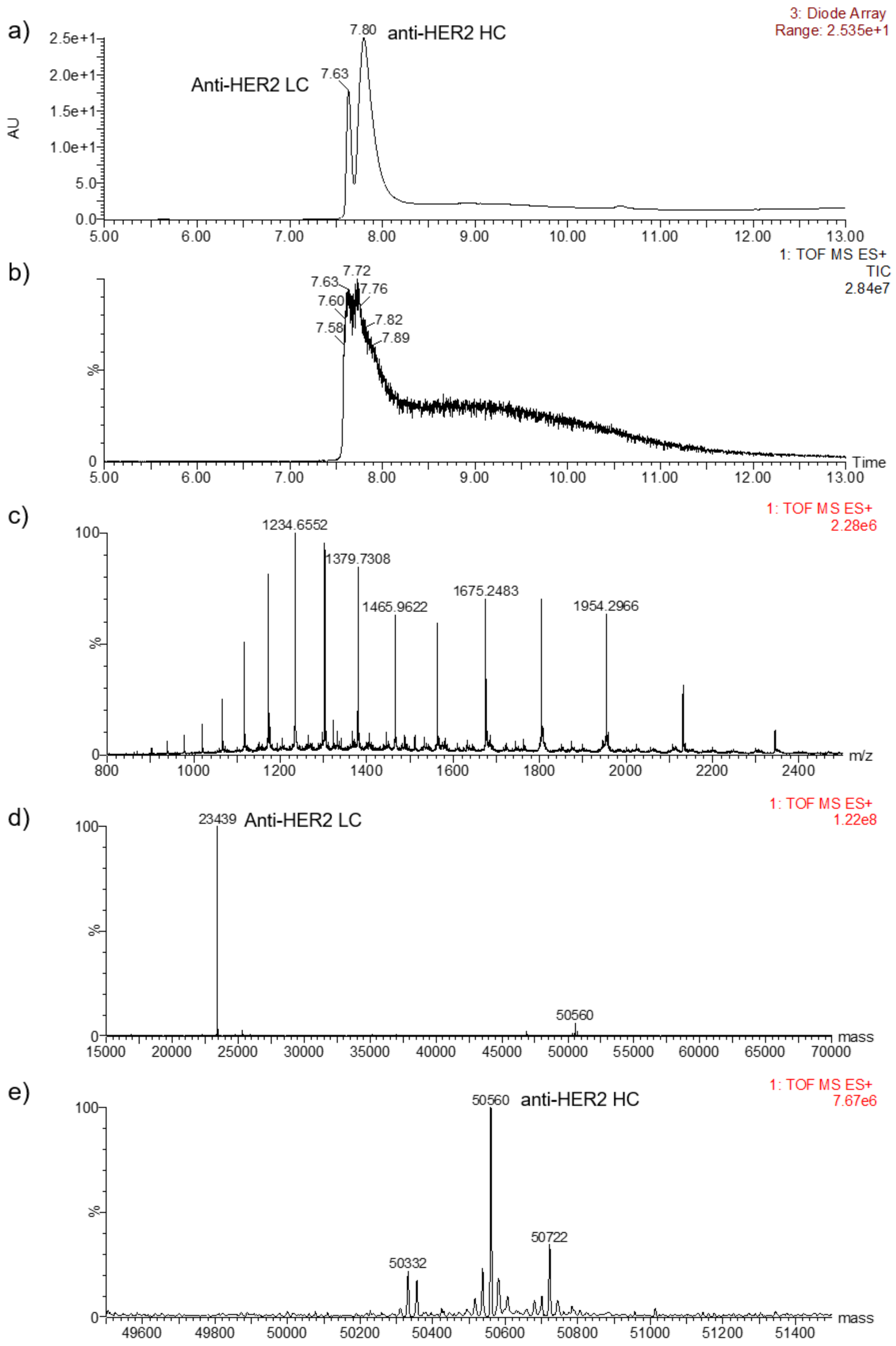

Supplementary Fig. 99: LC-ESI-HRMS analysis of reduced anti-HER2 mAb: a) UV/Vis chromatogram (RT 7.6 min anti-HER2 mAb LC and RT 7.8 min anti-HER2 mAb HC); b) TIC; c) combined ion series; d) deconvoluted spectrum: anti-HER2 mAb LC, calc. 23442 Da, found 23439 Da; e) zoom-in deconvoluted spectrum: anti-HER2 mAb HC calc. 50619 Da, found 50560 Da.

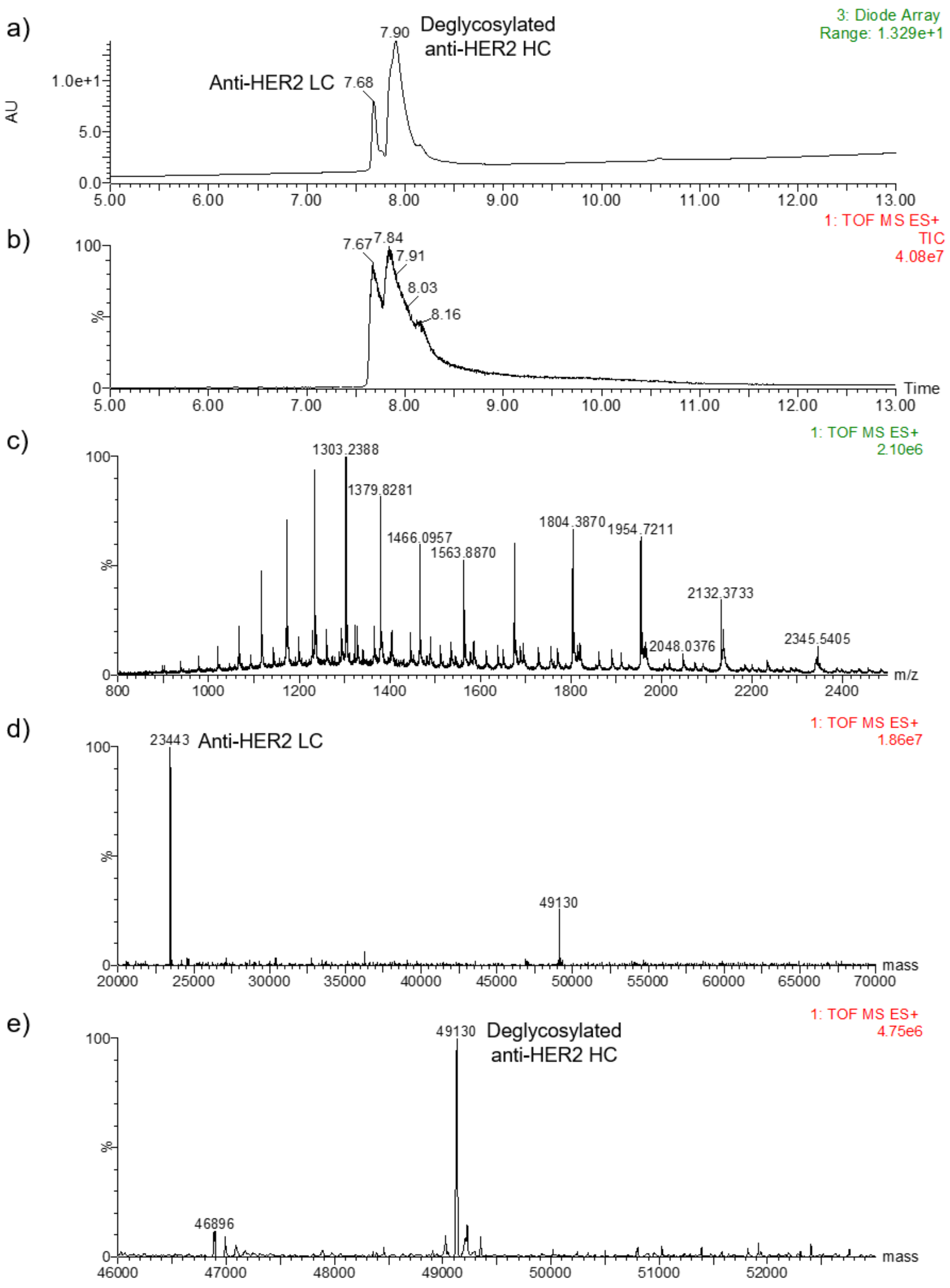

Supplementary Fig. 100: LC-ESI-HRMS analysis of reduced and deglycosylated anti-HER2 mAb: a) UV/Vis chromatogram (RT 7.7 min anti-HER2 mAb LC and RT 7.9 min deglycosylated anti-HER2 mAb HC); b) TIC; c) combined ion series; d) deconvoluted spectrum: anti-HER2 mAb LC, calc. 23442 Da, found 23443 Da; e) zoom-in deconvoluted spectrum: anti-HER2 mAb HC calc. 49157 Da, found 49130 Da.

9.4 Site-selective modification of the Cys-C3bot1

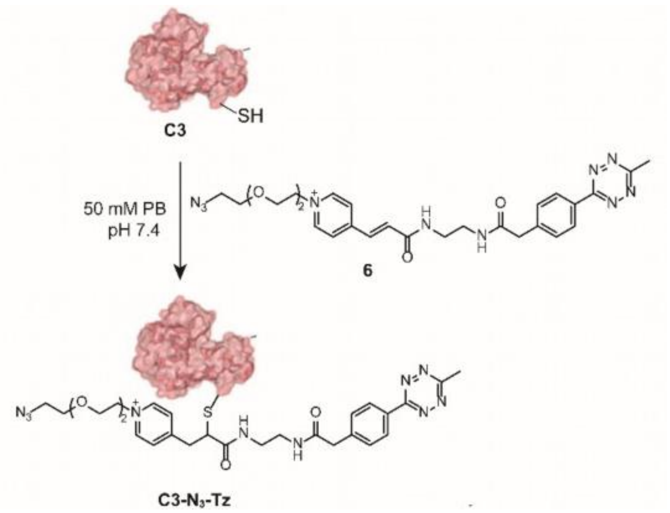

Supplementary Fig. 101: Site-selective modification of Cys-C3bot1 with compound **6**.

To a Cys-C3bot1 solution (0.372 mg/mL in 50 mM PB, pH 7.4) (300 µL, 0.0045 µmol), compound **6** (25.0 mg/mL in DMF) (20 equiv, 2.00 µL, 0.090 µmol) was added and the resultant mixture was incubated for 5 h at 20°C. The reaction mixture was transferred to an ultrafiltration tube (0.5 mL, 10 kDa, PES filter) to remove the excess compound **6** and organic solvent using 50 mM PB, pH 7.4 as exchange solvent (3x, 12000G, 10 min) to deliver **C3-N_3-*Tz*** conjugate.

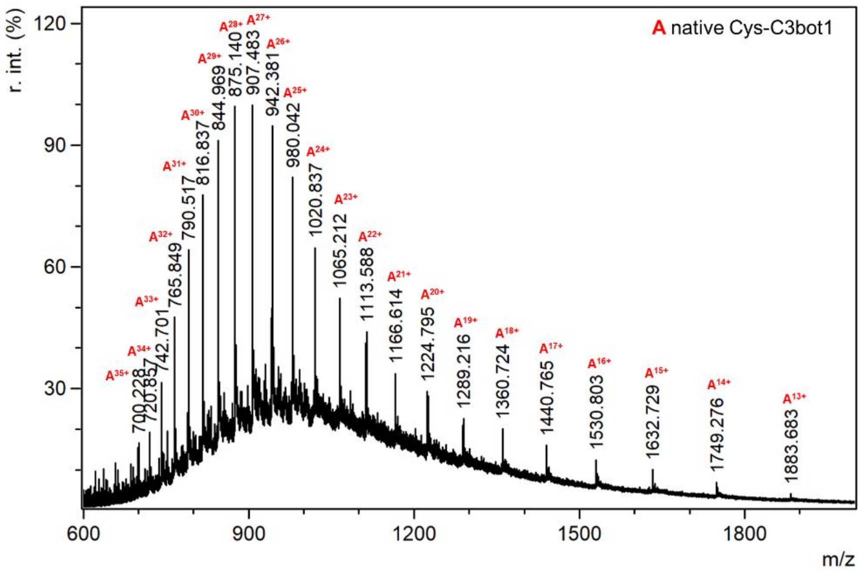

Supplementary Fig. 102: Combined ion series from the direct infusion of desalted Cys-C3bot1.

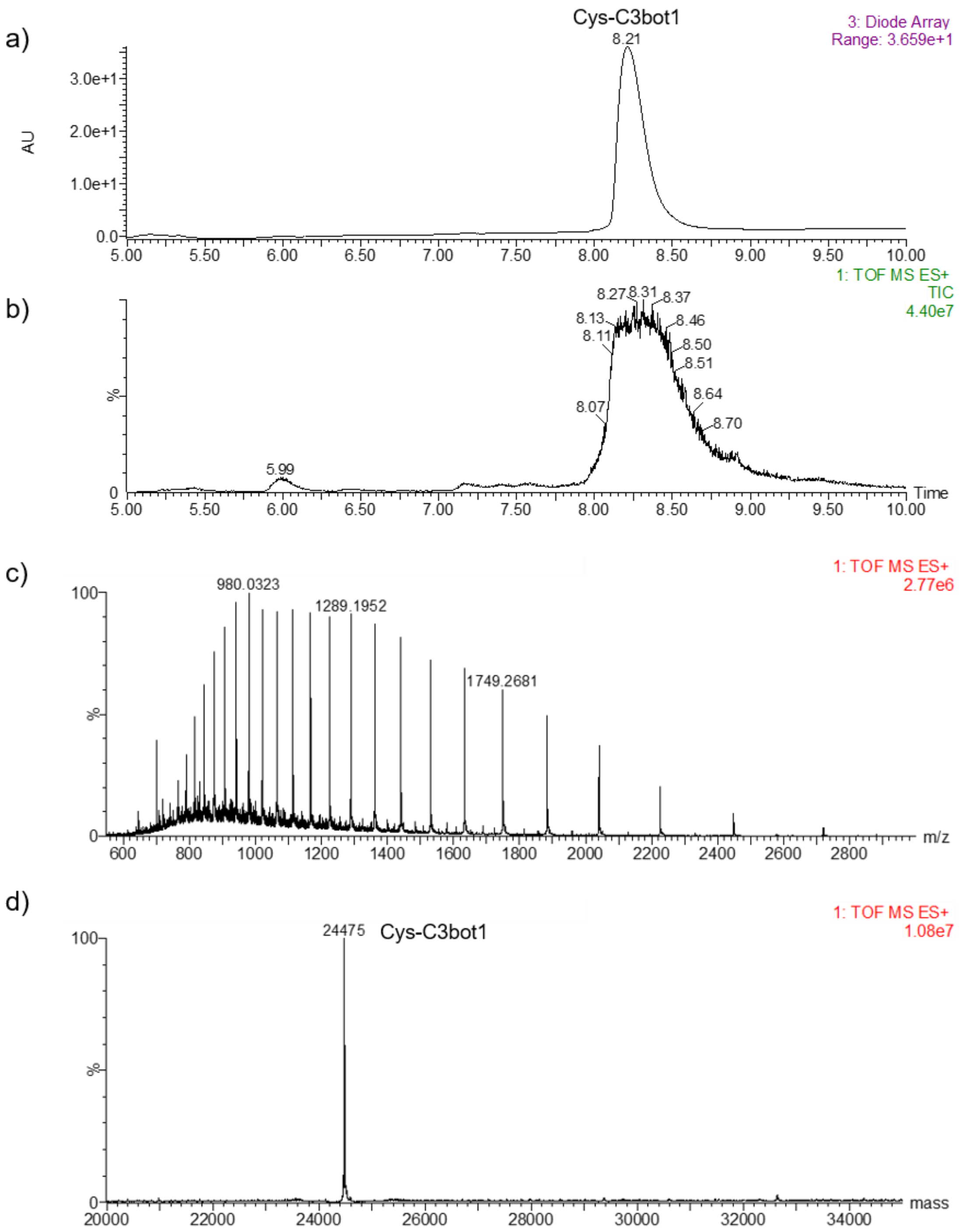

Supplementary Fig. 103: LC-ESI-HRMS analysis of native Cys-C3bot1: a) UV/Vis chromatogram and TIC; b) combined ion series; c) deconvoluted spectrum.

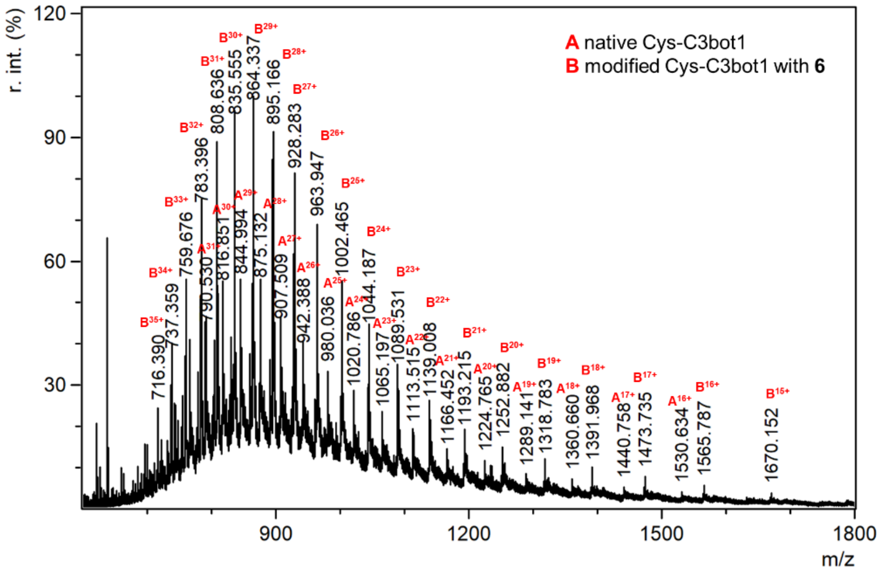

Supplementary Fig. 104: Combined ion series from the direct infusion of the desalted reaction mixture of Cys-C3bot1 modification with compound **6** to afford **C3-*N***

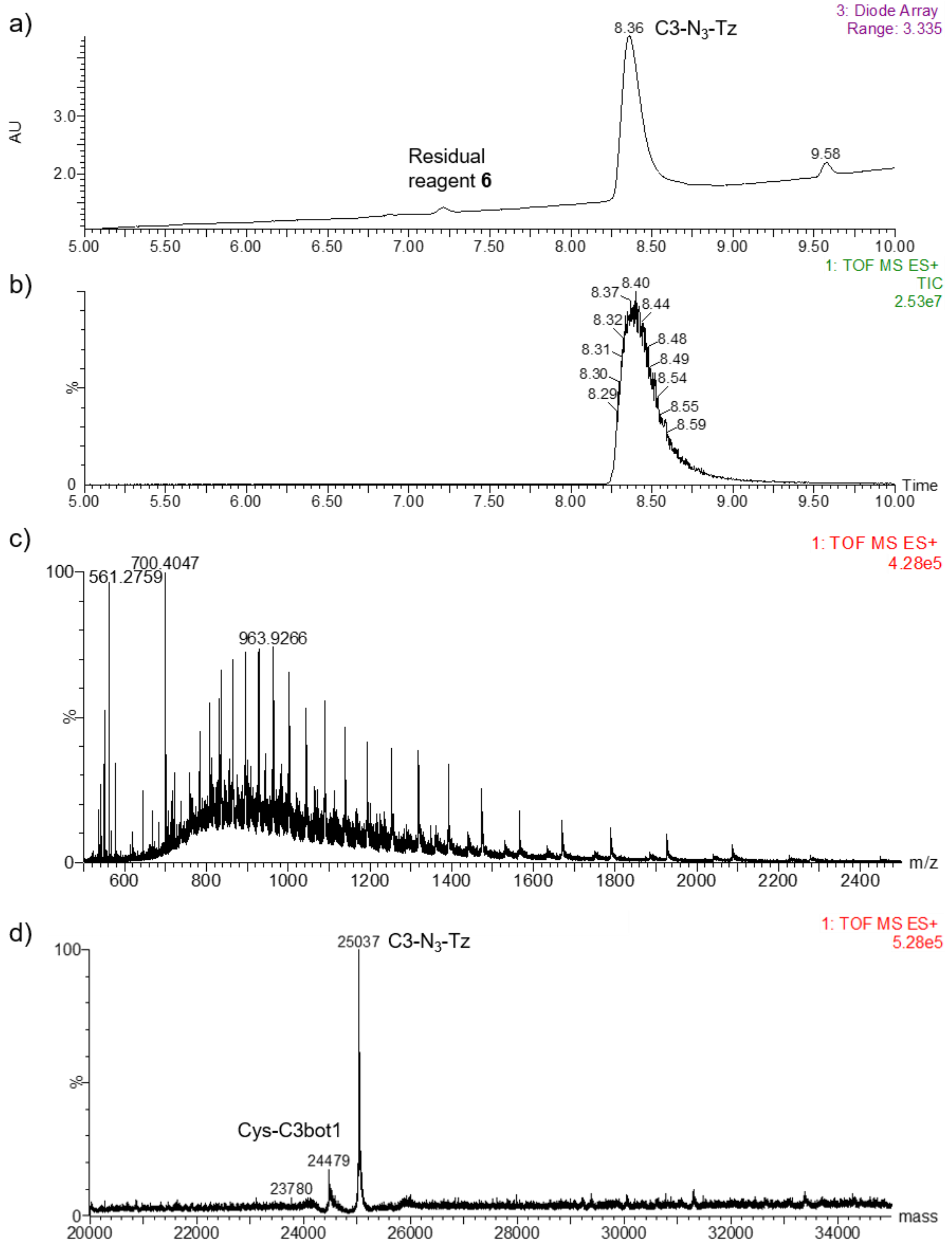

Supplementary Fig. 105: LC-ESI-HRMS analysis of Cys-C3bot1 modified with compound **6**: a) UV/Vis chromatogram and TIC; b) combined ion series; c) deconvoluted spectrum.

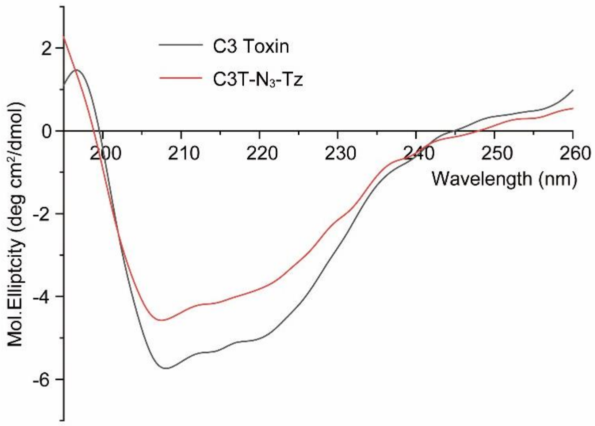

Supplementary Fig. 106: CD spectra of Cys-C3bot1 and modified toxin with compound **6** from 190 nm to 260 nm.

9.5 Dual-functionalization of Cys-C3bot1 with Cy3, Cy5 or RGDC

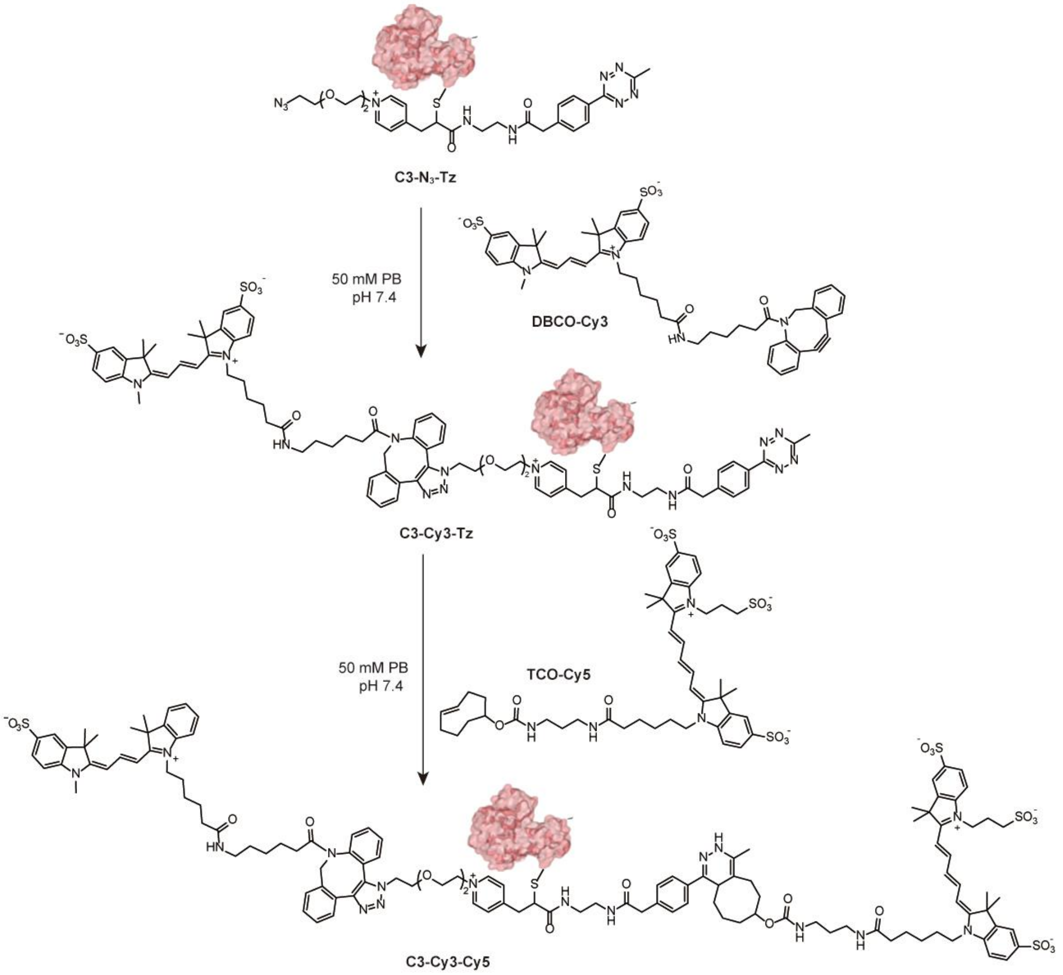

Supplementary Fig. 107: Dual functionalization of C3-N_3-Tz with a FRET pair (Cy3 and Cy5) *via* two bioorthogonal reactions.

A solution of Cy3-DBCO (5 mg/mL in H_2O/1% DMSO) (10 equiv, 2.87 µL, 0.015 µmol) was added to the previously purified **C3-*N3-Tz*** conjugate solution (100 µL) and mixed for 4h under same conditions. Then, Cy5-TCO.TEA (5 mg/mL in H_2O/1% DMSO) (10 equiv, 3.18 µL, 0.015 µmol) was added and mixed for 1 h. After confirming the successful C3 dual modification (*m/z* 26883) by ESI-HRMS*, the reaction mixture was purified by UF in a 6 mL tube (6x, 12000G, 5 min) using 50 mM PB, pH 7.4 as exchange solvent.

*An aliquot from each reaction step was desalted in a separate ultrafiltration tube (0.5 mL, 10 kDa, PES filter) using MQ water as exchange solvent (6x, 12000G, 10 min) to be analyzed by ESI-HRMS.

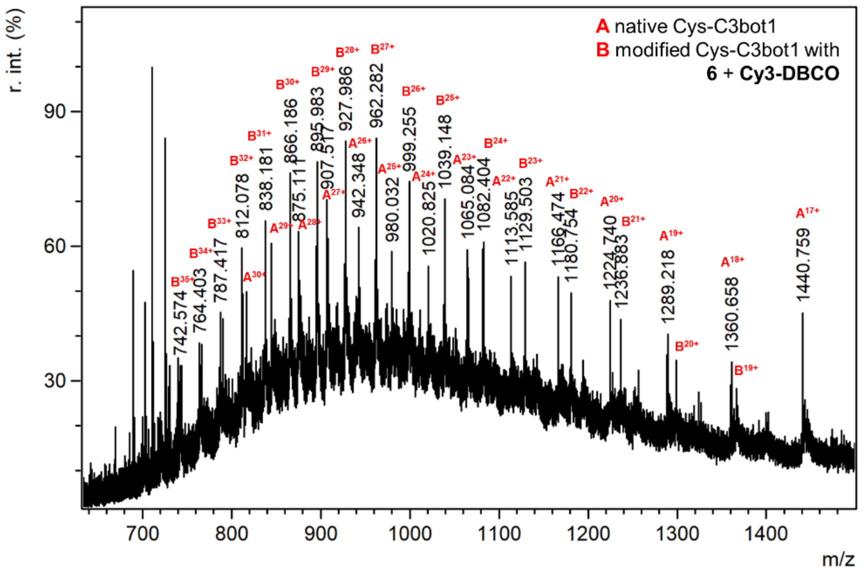

Supplementary Fig. 108: Combined ion series from the direct infusion of the desalted reaction mixture of **C3-*N₃-Tz*** conjugate modification with **Cy3-DBCO** to afford **C3-*Cy3-Tz*** conjugate.

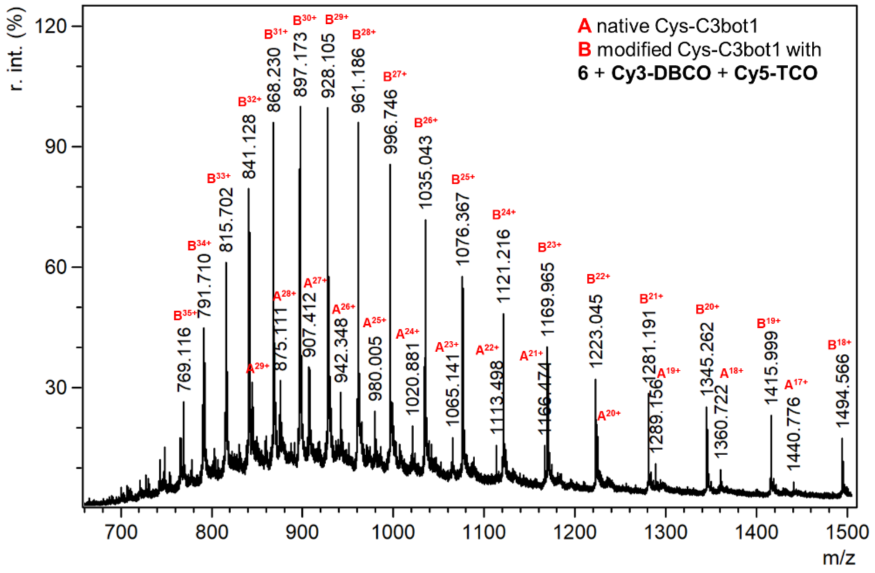

Supplementary Fig. 109: Combined ion series from the direct infusion of the desalted reaction mixture of **C3-*Cy3-Tz*** modification with compound **Cy5-TCO** to afford **C3-*Cy3-Cy5*** conjugate.

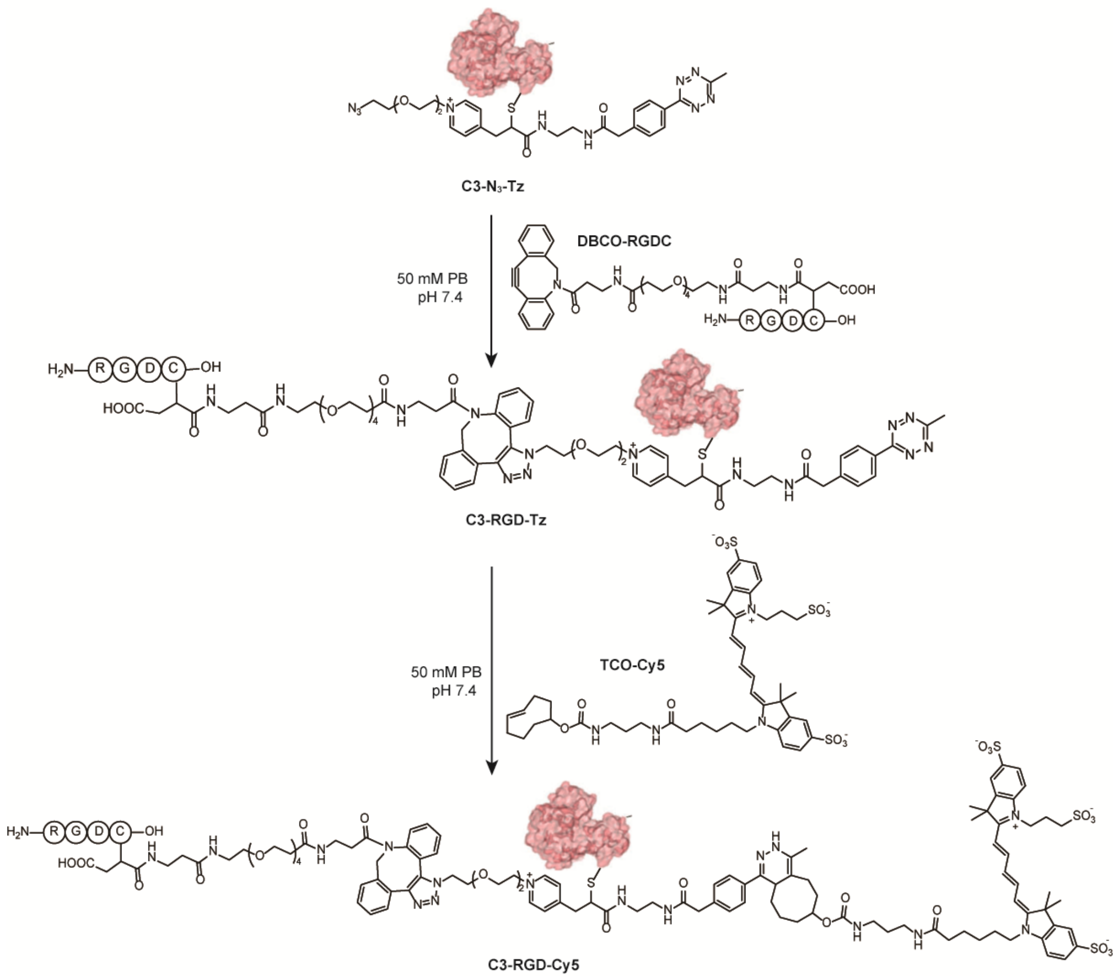

Supplementary Fig. 110: Dual functionalization of C3-N_3-Tz with an RGDC peptide and a Cy5 dye *via* two bioorthogonal reactions.

A solution of RGDC-DBCO (20 mM in 50 mM PB, pH7.4) (10 equiv, 0.75 µL, 0.015 µmol) was added, to the previously purified **C3-*N3-Tz*** conjugate solution (100 µL) and mixed for 4h under same conditions. Then, Cy5-TCO.TEA (5 mg/mL in H_2O/1% DMSO) (10 equiv, 3.18 µL, 0.015 µmol) was added and mixed for 1 h. After confirming the successful C3 dual modification (*m/z* 27110) by ESI-HRMS*, the reaction mixture was purified by UF in a 6 mL tube (6x, 12000G, 5 min) using 50 mM PB, pH 7.4 as exchange solvent.

*An aliquot from each reaction step was desalted in a separate ultrafiltration tube (0.5 mL, 10 kDa, PES filter) using MQ water as exchange solvent (6x, 12000G, 10 min) to be analyzed by ESI-HRMS.

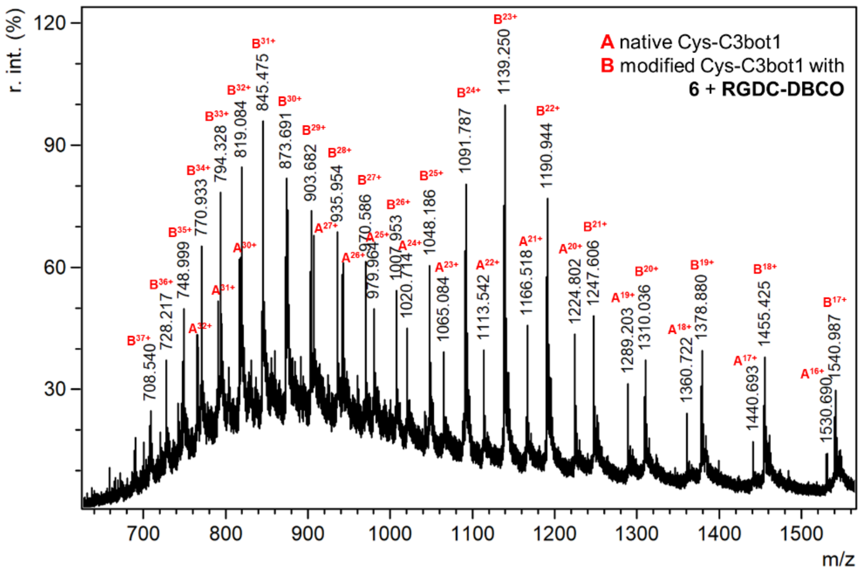

Supplementary Fig. 111: Combined ion series from the direct infusion of the desalted reaction mixture of **C3-*N₃*-*Tz*** conjugate modification with **RGDC-DBCO** to afford **C3-*RDG-Tz*** conjugate.

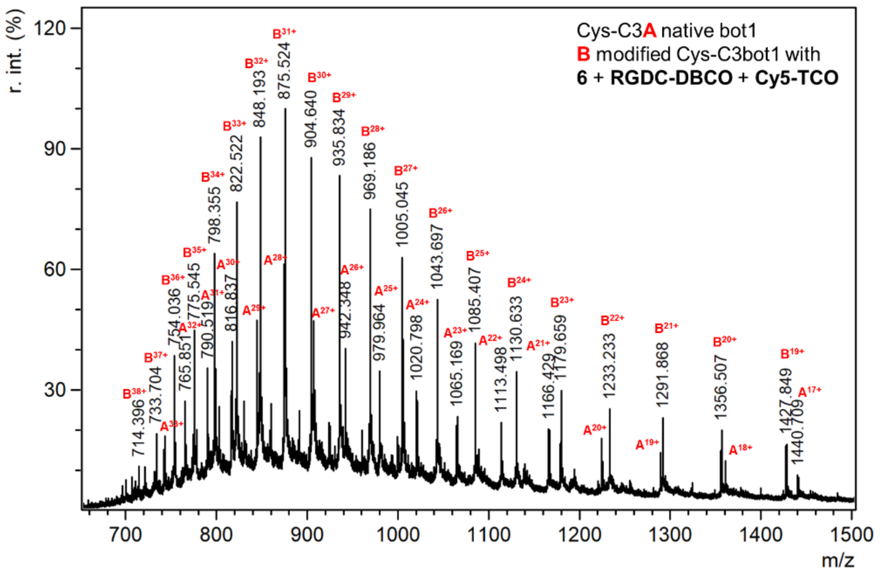

Supplementary Fig. 112: Combined ion series from direct infusion of desalted reaction mixture of **C3-*RDG-Tz*** conjugate modification with **Cy5-TCO** to afford **C3-*RGD-Cy5*** conjugate.

The degree of labeling (DOL) of the RGD- and Cy5-modified C3 Toxin (**C3-*RGD-Cy5***) using the absorbance of the protein, DBCO and Cy5 dye. Around 71% of labeling efficiency can be achieved with C3 over three sequential steps.

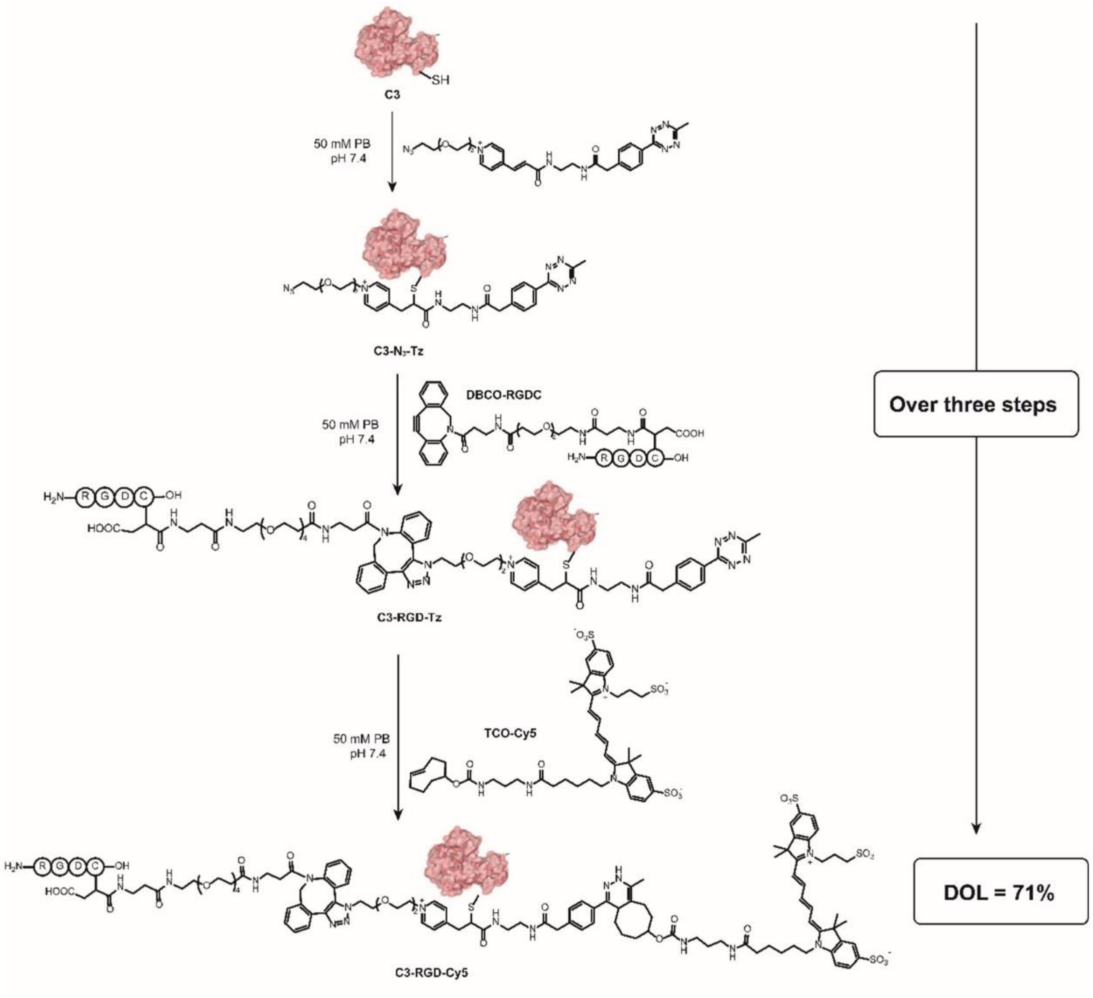

Wavelength	Abs (arb. units)	Average	Calculated conc. (µM)
A280nm	0.335	0.345	corrected [C3 toxin]* 7.88
	0.363		
	0.338		
A309nm	0.141	0.131	
	0.129		
	0.123		
A650nm	1.514	1.401	5.6
	1.372		
	1.316		

*concentration correction
Org. Biomol. Chem., **2020**, *18*, 1140-1147
SulfoCy5 dye CF(280nm) = 0.03
ε_{650} (the extinction coefficient of Cy5 dye) = 250000 M^{-1} cm^{-1}

Interchim - DQP580_DBCO CF
DBCO CF(280nm) = 1.089
ε_{309} (the extinction coefficient of DBCO) = 250000 M^{-1} cm^{-1}

$$\text{Degree of labelling (DOL) of C3-RGD-Cy5} = \frac{A_{max} \times \varepsilon_{280}\,(\text{protein})}{\left(A_{280} - A_{max} \times CF\right) \times \varepsilon_{max}} = \mathbf{71\%}$$

Supplementary Fig. 113: Calculation of the DOL of ***C3-RGD-Cy5***.

10 *In vitro* studies

10.1 Confocal microscopy

For confocal studies, commercially available A549 from human lung carcinoma tissue was cultured at 37 °C and 5 % CO_2 in Dulbecco's Modified Eagle's Medium (DMEM, 4.5g/L D-glucose, L-glutamine; Gibco by ThermoFisher Scientific, Germany), supplemented with 10 % FBS (Sigma–Aldrich, Germany) and 1 % Penicillin/Streptomycin (Invitrogen, Germany). Cells were cultured in a T75 culture flask and passaged two to three times per week using TrypLE™ Express (Gibco by ThermoFisher Scientific, Germany).

A549 cells were seeded at a density of $2*10^5$ cells per well in DMEM (10% FBS, 1 % penicillin/streptomycin) within an IBIDI8-well confocal slide. After adhering for 24h, cells were treated with the C3-*RGD-Cy5* and C3-*Cy3-Cy5* for 24h at 37°C, 5 %CO_2. The stock C3 protein samples were in 50 mM PB, pH 7.4 at a concentration of 8 µM of C3-*RGD-Cy5* and 11 µM of C3-*Cy3-Cy5* and diluted to a final concentration of 300 nM with DMEM. After incubation, cells were washed thrice with PBS, co-stained with NucBlue and fixed with 4% PFA. Cells were imaged with a Leica Stellaris 8 microscope HC PL APO CS2 40x/1.25GLYC objective, 405 nm at 1.0% laser power for NucBlue excitation, 554 nm at 0.6% for Cy3 and 649 nm at 2.6% for Cy5.

10.2 Intoxication studies

For the intoxication studies, commercially available A549 cells were cultivated in MEM medium (Gibco-Life Technologies, Carlsbad, CA, USA) supplemented with 10 % fetal calf serum (Gibco-Life Technologies, Carlsbad, CA, USA), 1 mM sodium pyruvate (Gibco-Life Technologies, Carlsbad, CA, USA), 0.1 mM non-essential amino acids (Thermo Fisher Scientific, Waltham, MA, USA) and 100 U/mL (1%) penicillin–streptomycin (Gibco-Life Technologies, Carlsbad, CA, USA). They were cultured at 37 °C, 5 % CO_2 and constant humidity. Subcultivation was performed every 3 to 4 days with split ratios of 1:3 to 1:10 after trypsinization (Roche Diagnostics, Basel, SUI). The A549 cells were seeded in a 24-well microtiter plate with a cell number of $5*10^5$ cells per ml 48 h prior to treatment and incubated at 37 °C, 5 % CO_2. They were then treated with 100 nM or 300 nM of Cys-C3bot1 (C3), C3-*Cy3-Cy5*, C3-*RGD-Cy5*, respectively or left untreated as a control. Phase contrast microscopy was used to monitor changes in cell morphology after intoxication.

10.3 Western Blot analysis for ADP-ribosylation

In this experiment, A549 cells were seeded and treated with the individual C3 conjugates or wildtype C3bot1 as described before in section 10.2. After 8 h incubation, the residual toxin was removed by washing twice with PBS (137 mM NaCl, 2.7 mM KCl, 8 mM Na_2HPO_4, and 1.8 mM KH_2PO_4; pH 7.4) before the cells were lysed at -20 °C for at least 30 min. The cells were scratched off in ADP ribosylation buffer (20 mM Tris-HCl, 1 mM EDTA, 1 mM DTT, 5 mM $MgCl_2$,

complete (1:50, freshly added); pH 7.5). The lysates were mixed with 10 pmol C3lim and biotin-labelled NAD^+ (6-biotin-17-NAD^+, 10 µM) as co-substrate and the sequential ADP-ribosylation reaction was performed at 37 °C for 30 min. The reaction was stopped by adding Laemmli buffer (0.3 M Tris-HCl, 10% SDS, 37.5% glycerol, 0.4 mM bromophenol blue) and heat denaturation at 95 °C for 10 min. SDS-PAGE was performed using a 12.5 % acrylamide gel before transferring the proteins onto a nitrocellulose membrane by semi-dry blotting. For documentation, the gel was stained with Coomassie Brillant Blue R250 (SERVA Electrophoresis GmbH, Heidelberg, GER) and the membrane was controlled by Ponceau S AppliChem GmbH, Darmstadt, GER) staining. Blocking with 5 % skim milk powder solution in PBS-T (137 mM NaCl, 2.7 mM KCl, 8 mM Na_2HPO_4, 1.8 mM KH_2PO_4, 0.1 % Tween20; pH 7.4) for 1 h at room temperature (RT) prevented unspecific antibody binding. The membrane was either incubated overnight at 4 °C with mouse Hsp90 α/β antibody (1:1000 in PBS-T; F-8, Article number: sc-13119, Santa Cruz Biotechnology, Dallas, TX, USA) as loading control or 1 h at RT with streptavidin-peroxidase conjugate (1:5000 in PBS-T; Article number: 11089153001, Sigma-Aldrich, St. Louis, MO, USA) to detect the biotin-labeled target protein. However, if the cellular Rho was already ADP-ribosylated by C3 during the first intoxication of the intact cells, less Rho is left unmodified for the subsequent sequential ADP-ribosylation reaction. Thus, a weak signal in the Western Blot corresponds to a high ADP-ribosyltransferase activity of the toxin in living cells. The experiment was performed twice (n1 and n2), each with duplicates that were run on two different membranes (n1.1, n1.2, n2.1, n2.2). For each of the four membranes, the loading control and the corresponding Rho(non-ADP-rib.) signal were detected on the same membrane that was cut horizontally to enable incubation with the different antibodies/binding proteins for Hsp90 and biotin. Hsp90 signals were detected with the iBright device, while the Rho-signals were detected in a classical dark chamber on X-ray film for a better control of exposure times. For the latter, the marker bands were transferred manually, since they cannot be detected in the dark chamber and the X-ray films were scanned for digitalisation. An internal assay control was performed where the cells were treated with C2IIa/C2IN-C3lim (100/100 nM). This control will ensure C3 uptake and show a maximal C3 intoxication effect in cells. The effect of the internal control is provided in the source data.

General statement for cell experiments

All experiments to analyze the effect of C3 toxin on cells were performed with commercially available cell lines (A549 cells). Therefore, as a standard sample size in this field of research, these experiments were performed four independent times with duplicates of each condition per experiment. This sample size was chosen according to previous studies in the same area of research.[12-14]

11 Supporting figures

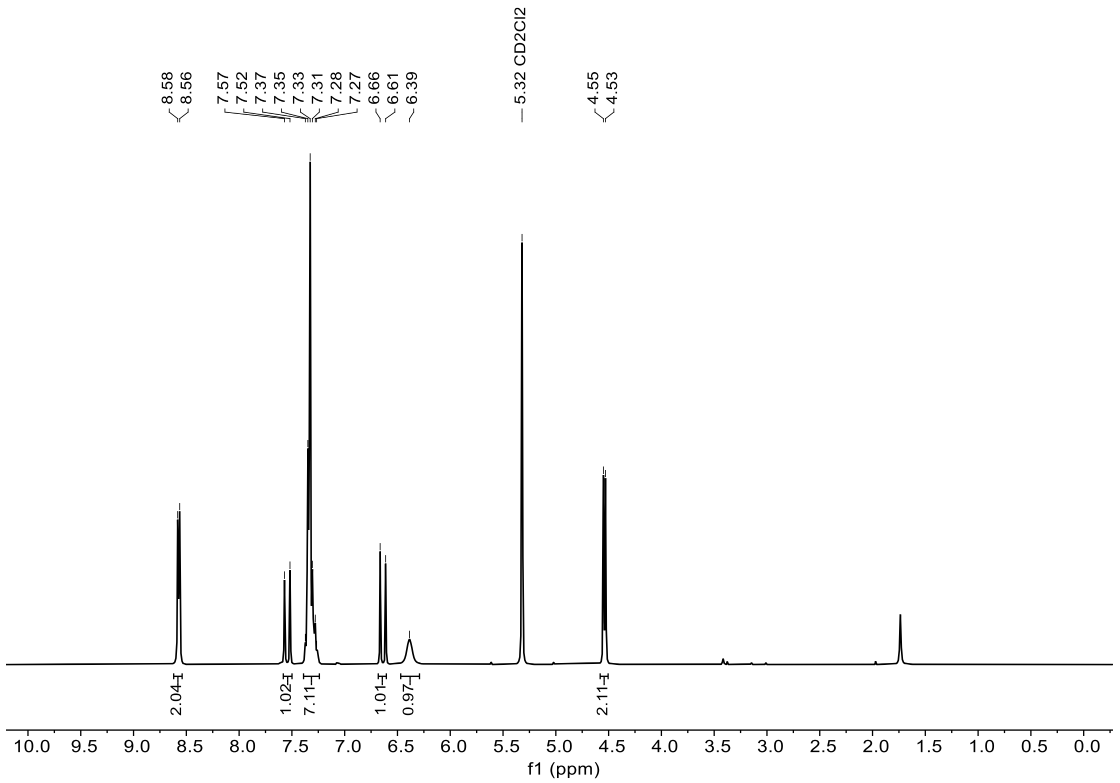

Supplementary Fig. 114: ^{1}H NMR of compound **12** (300 MHz, CD_2Cl_2, 298 K).

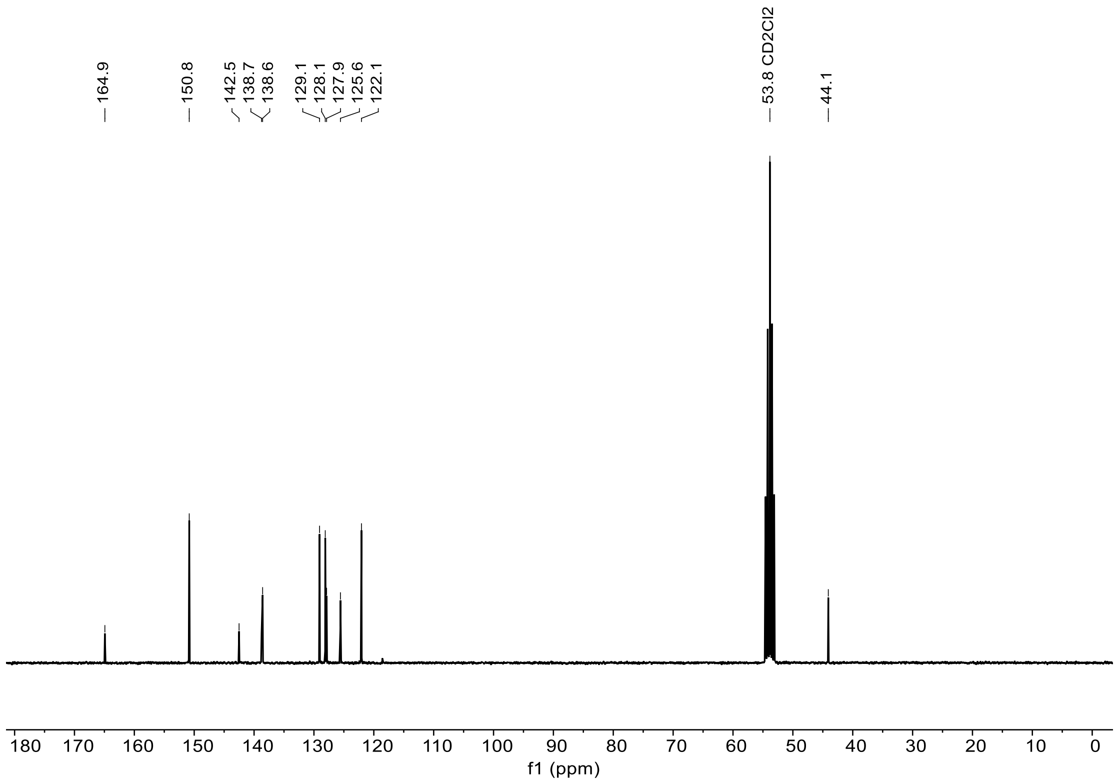

Supplementary Fig. 115: ^{13}C NMR of compound **12** (75 MHz, CD_2Cl_2, 298 K).

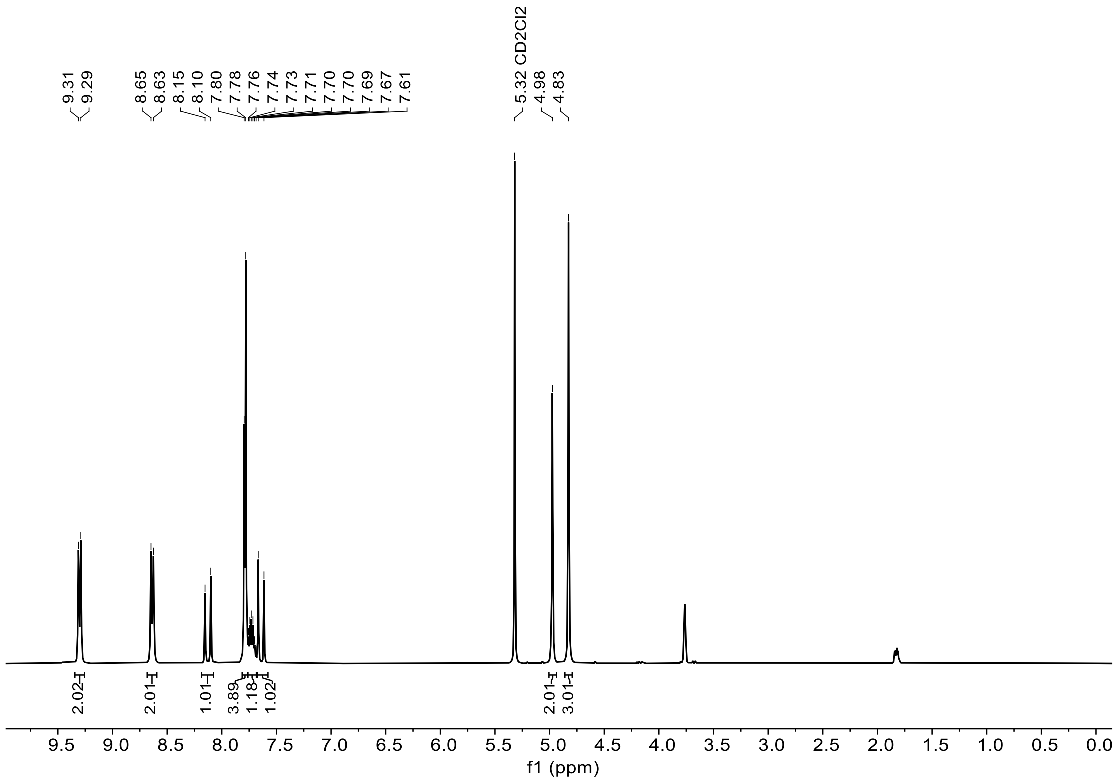

Supplementary Fig. 116: ^{1}H NMR of compound **1** (300 MHz, CD_2Cl_2, 298 K).

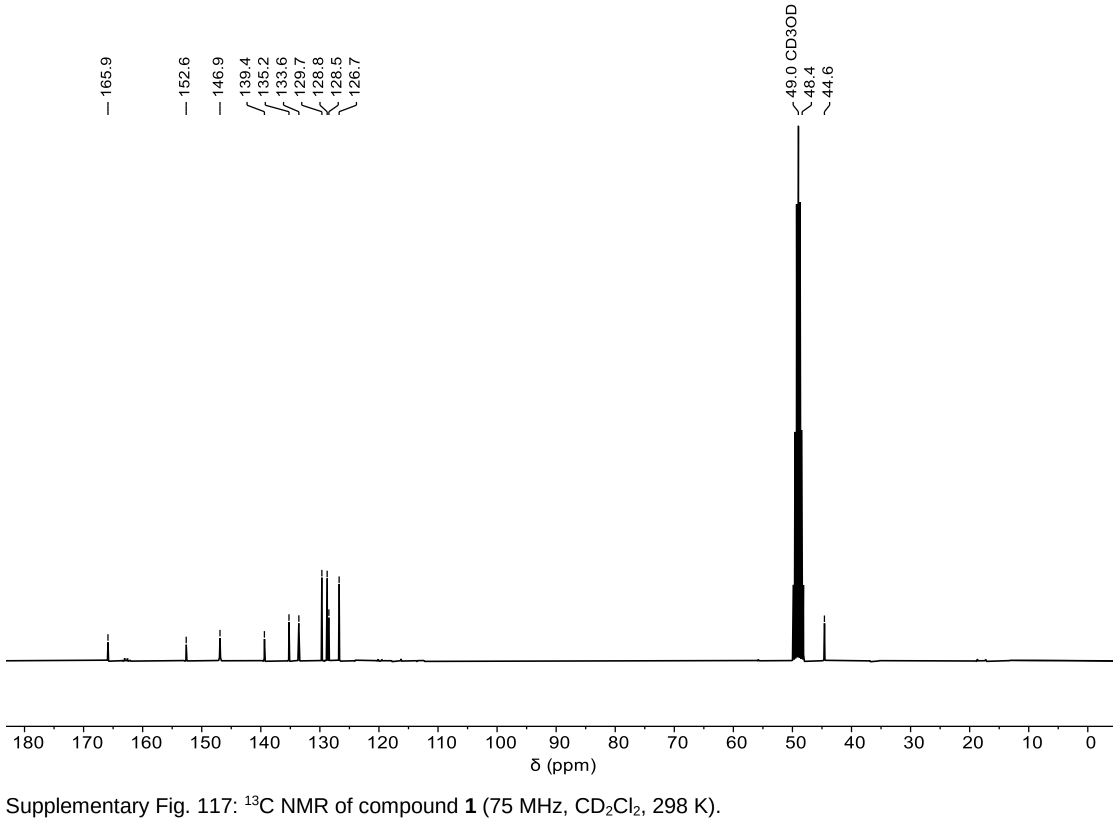

Supplementary Fig. 117: ^{13}C NMR of compound **1** (75 MHz, CD_2Cl_2, 298 K).

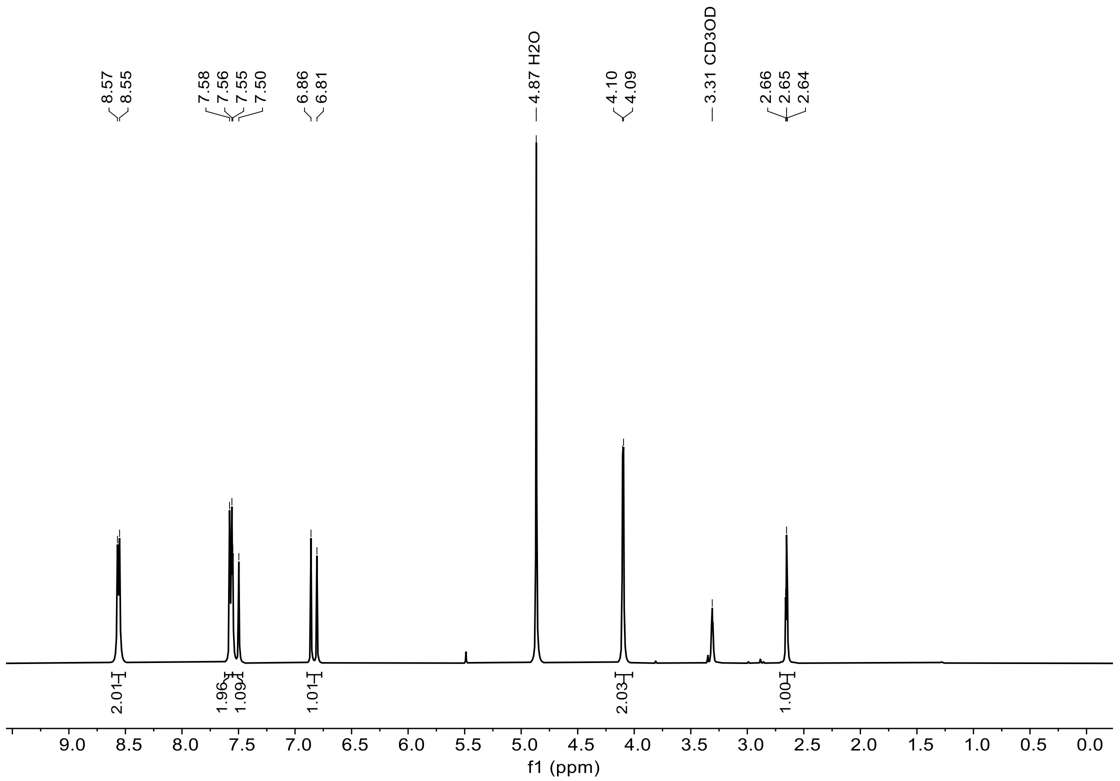

Supplementary Fig. 118: ^{1}H NMR of compound **13** (300 MHz, CD_3OD, 298 K).

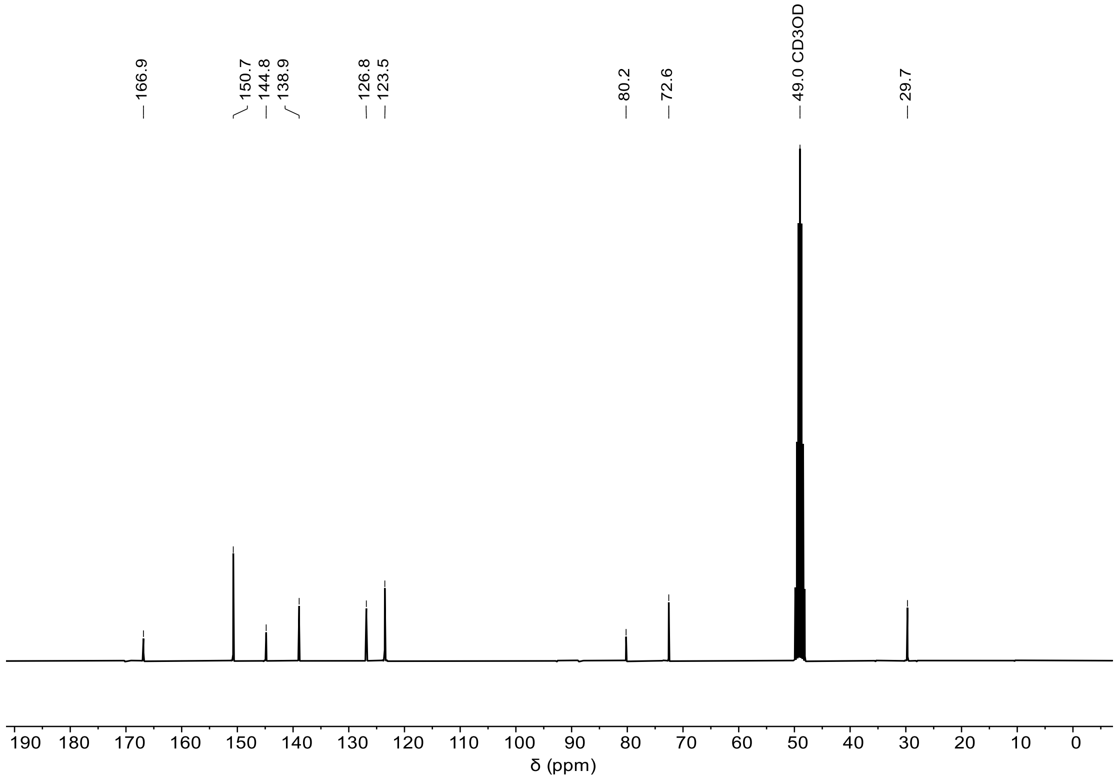

Supplementary Fig. 119: ^{13}C NMR of compound **13** (75 MHz, CD_3OD, 298 K).

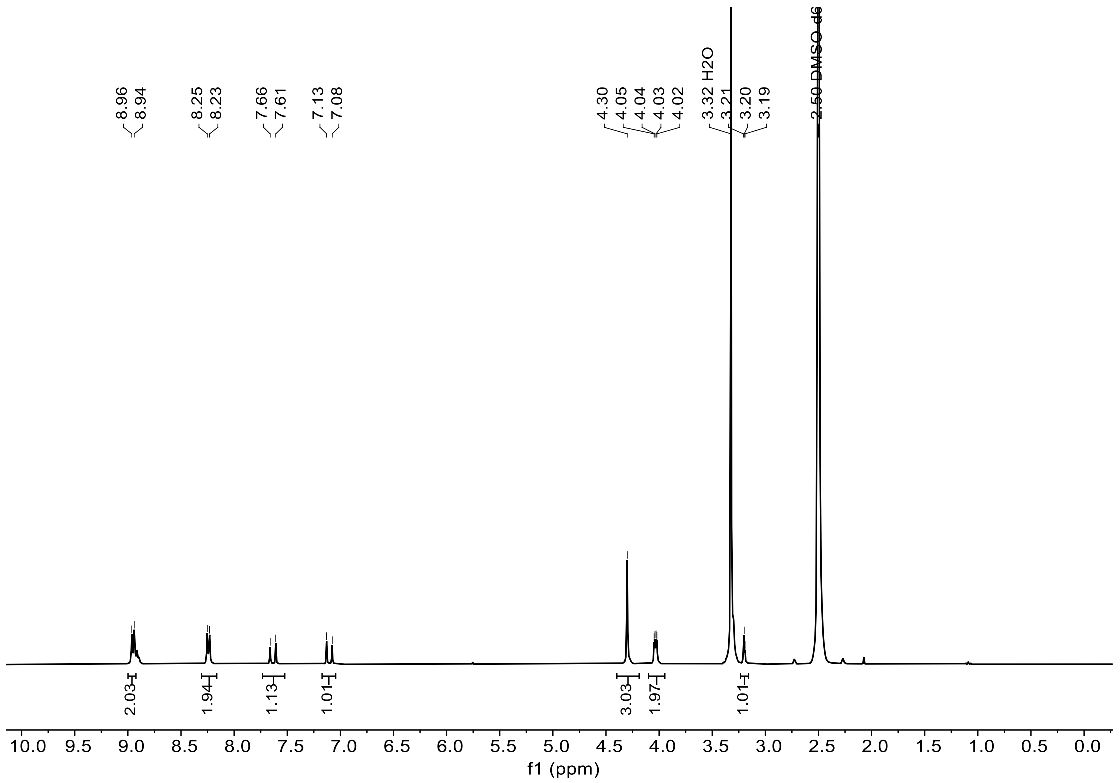

Supplementary Fig. 120: ^{1}H NMR of compound **2** (300 MHz, $(CD_3)_2SO$, 298 K).

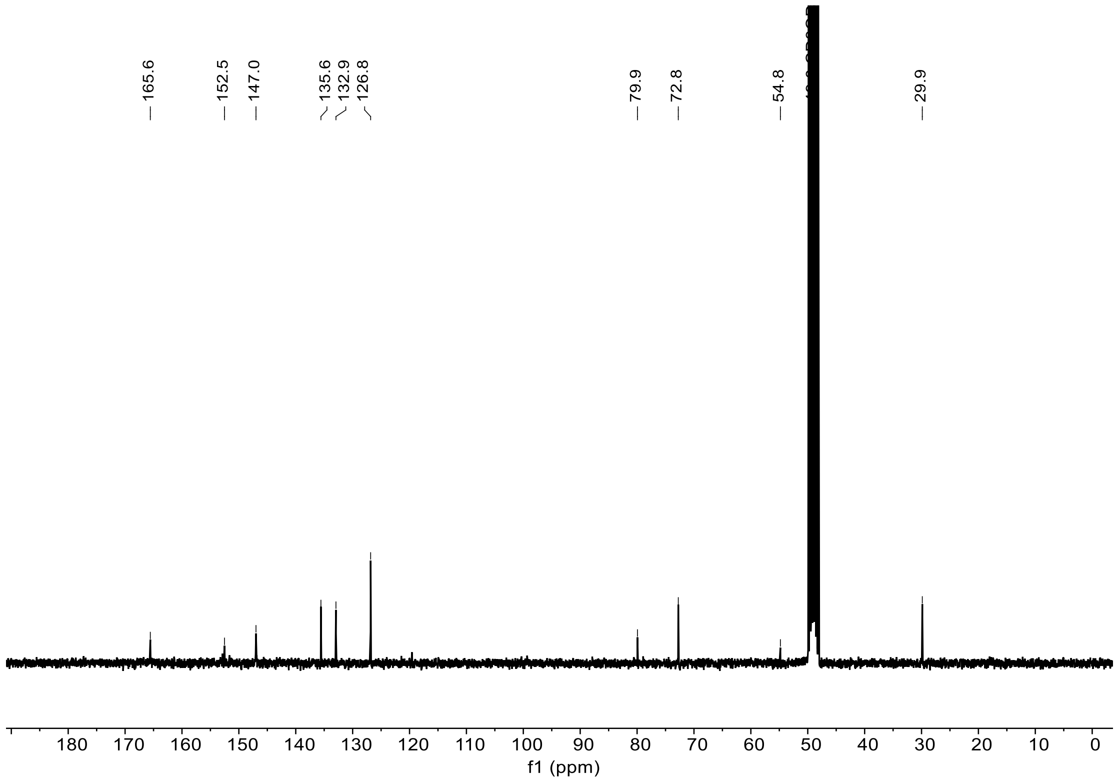

Supplementary Fig. 121: ^{13}C NMR of compound **2** (75 MHz, CD_3OD, 298 K).

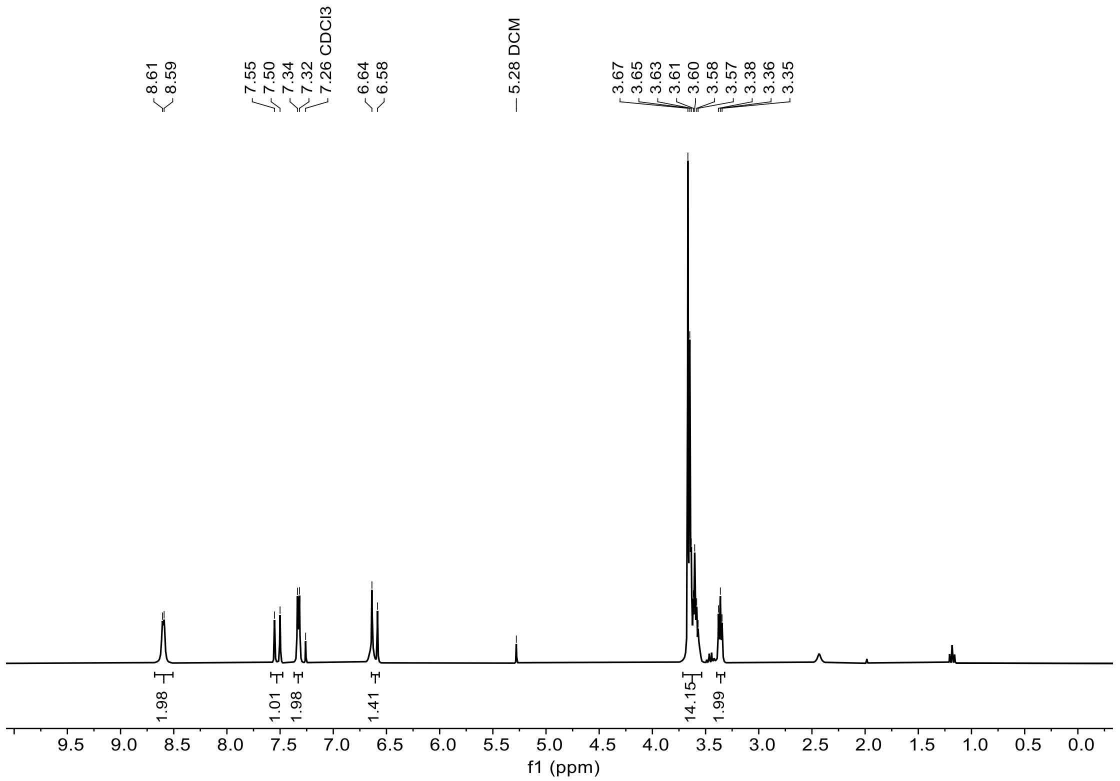

Supplementary Fig. 122: ^{1}H NMR of compound **14** (300 MHz, $CDCl_3$, 298 K).

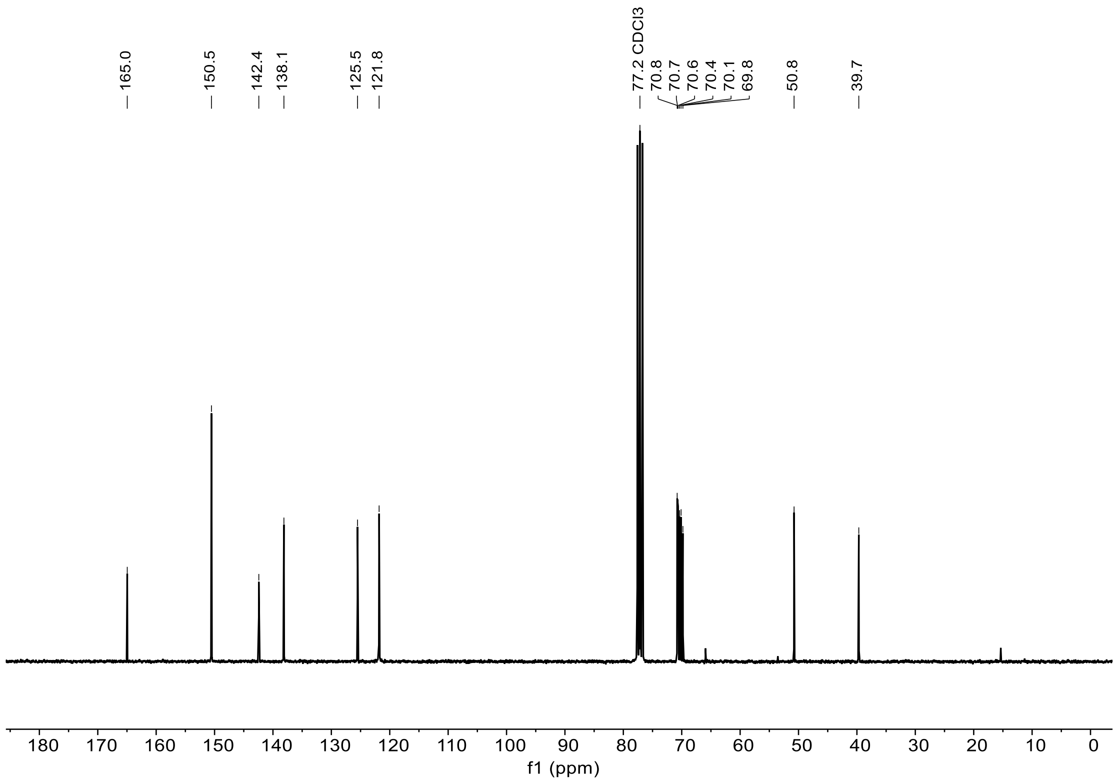

Supplementary Fig. 123: ^{13}C NMR of compound **14** (75 MHz, $CDCl_3$, 298 K).

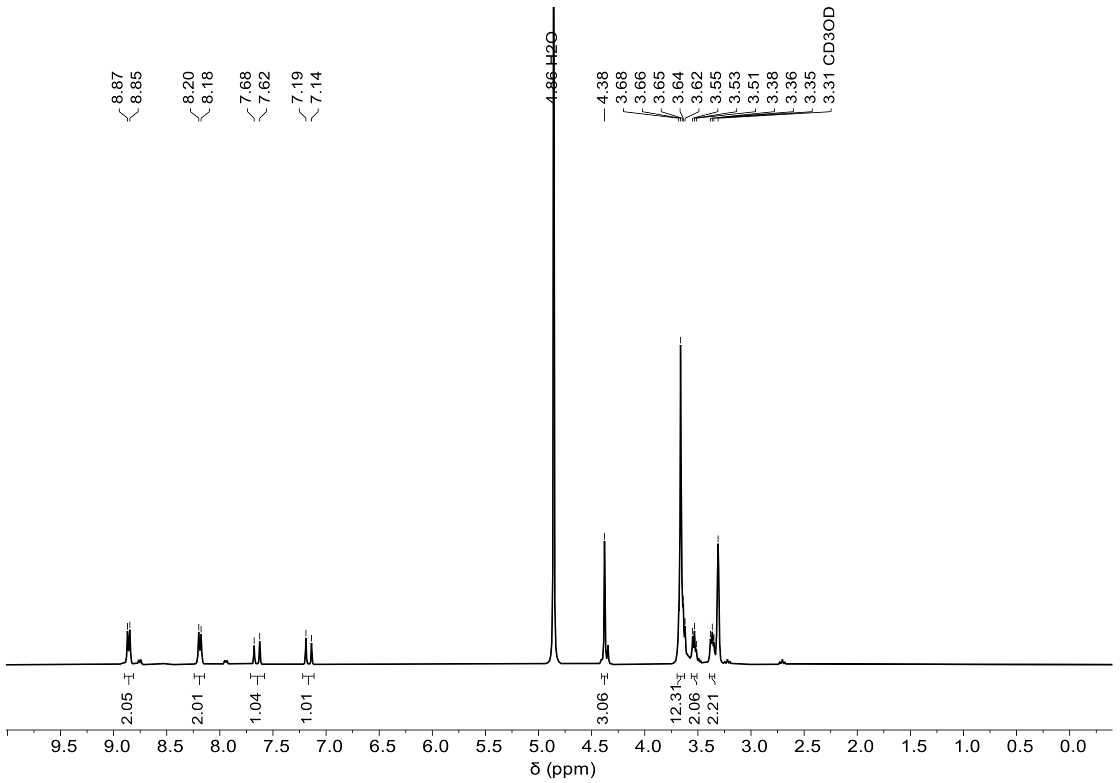

Supplementary Fig. 124: ^{1}H NMR of compound **3** (300 MHz, CD_3OD, 298 K).

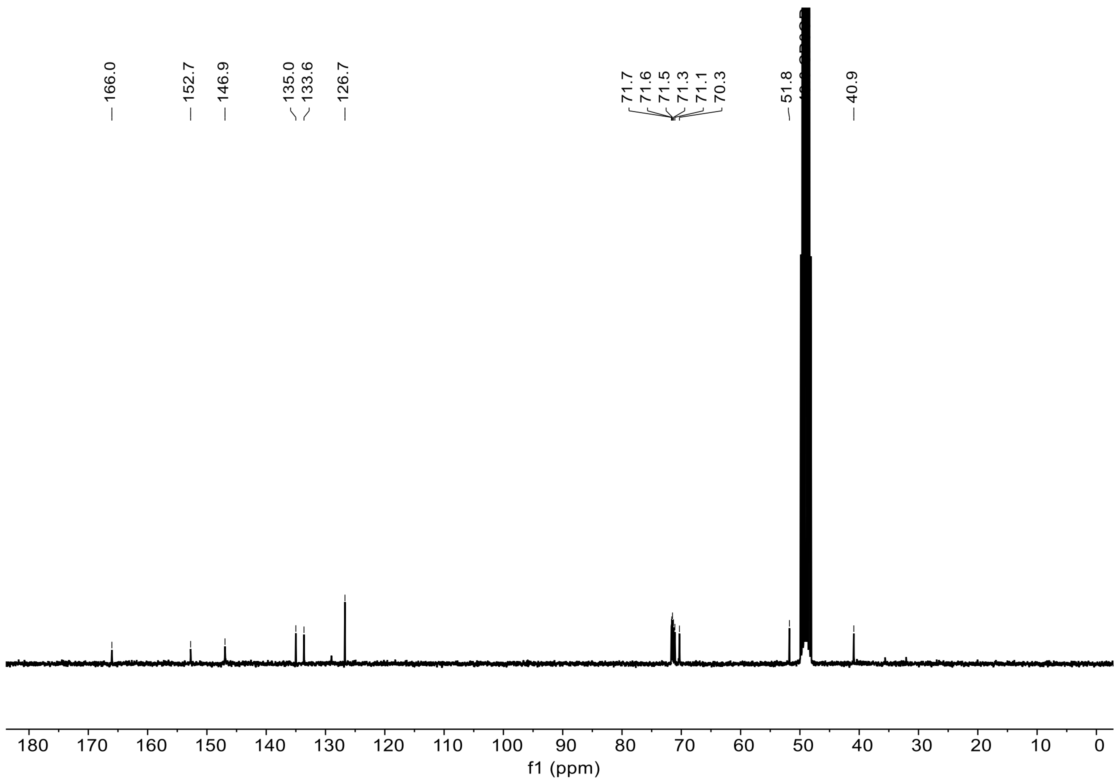

Supplementary Fig. 125: ^{13}C NMR of compound **3** (75 MHz, CD_3OD, 298 K).

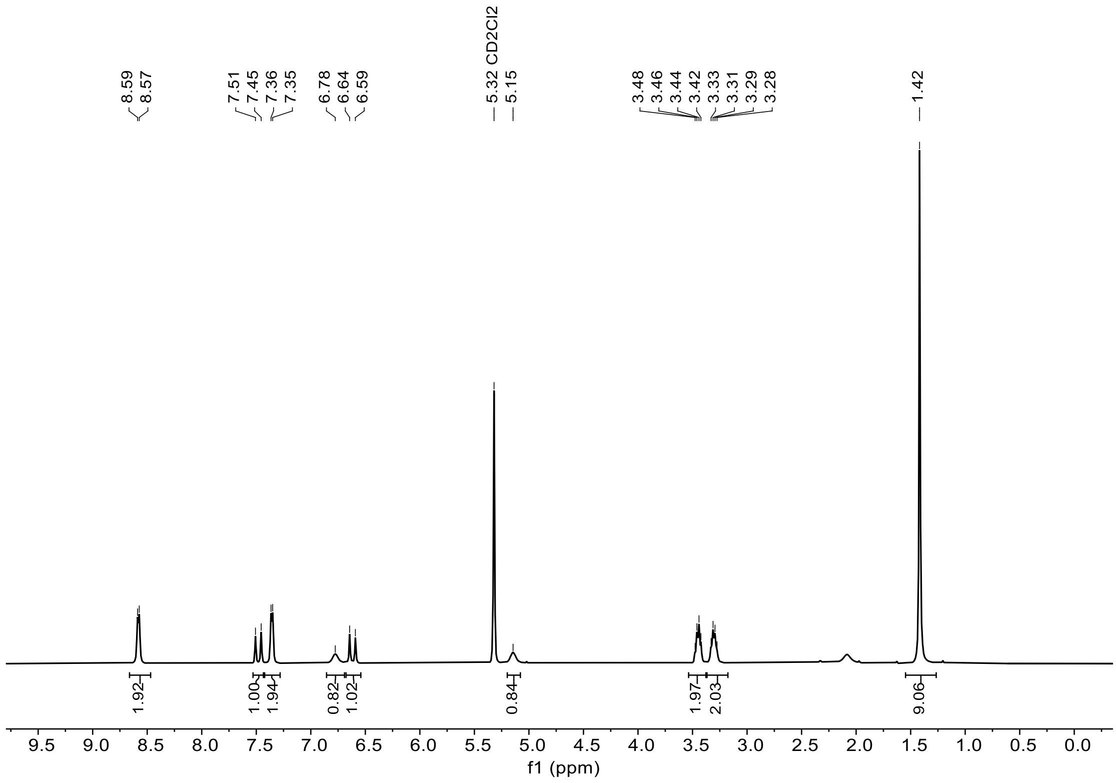

Supplementary Fig. 126: ^{1}H NMR of compound **15** (300 MHz, CD_2Cl_2, 298 K).

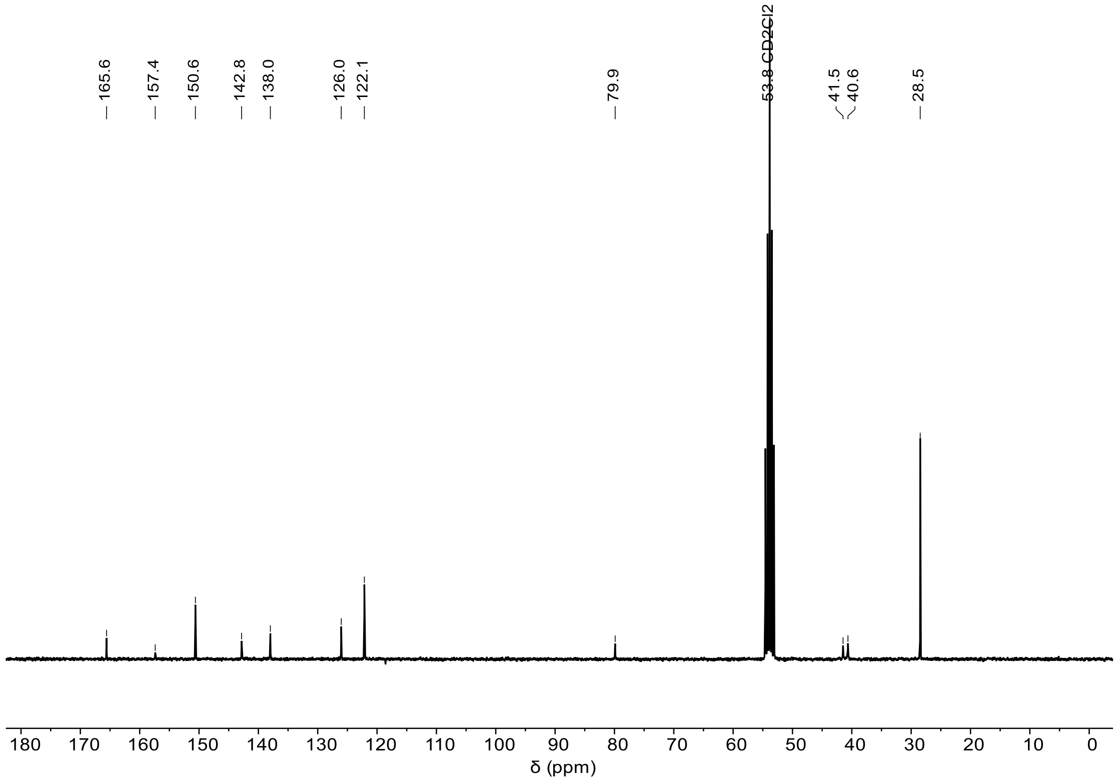

Supplementary Fig. 127: ^{13}C NMR of compound **15** (75 MHz, CD_2Cl_2, 298 K).

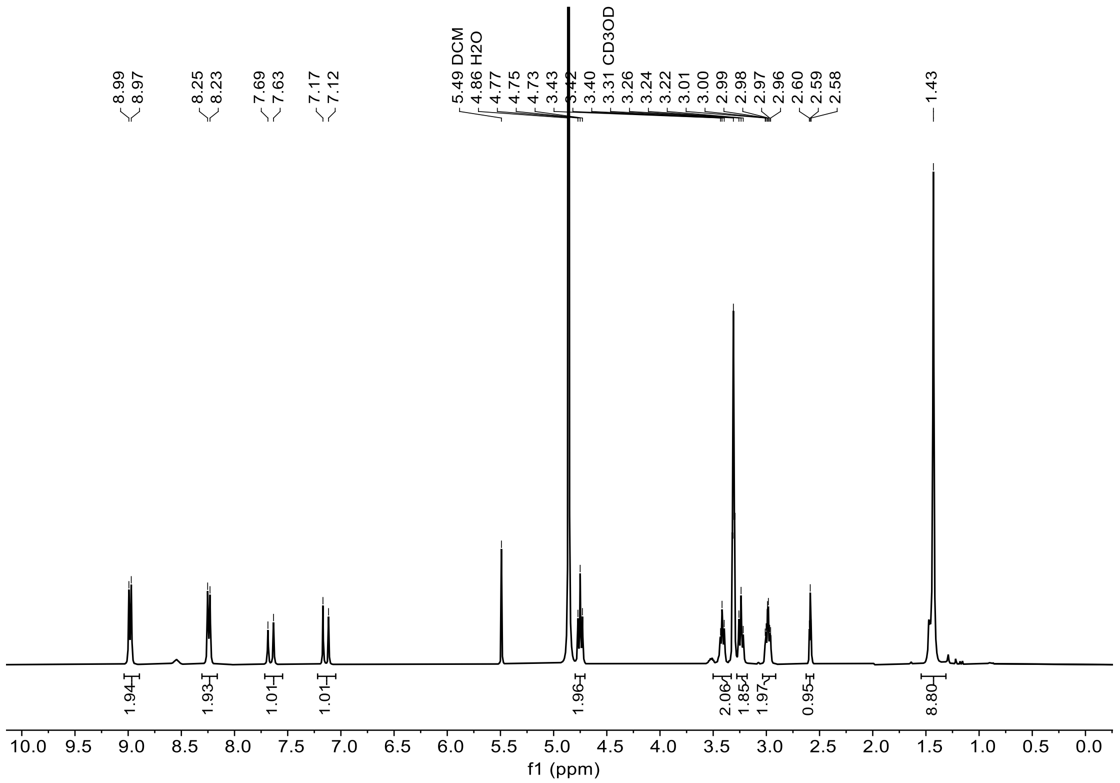

Supplementary Fig. 128: ^{1}H NMR of compound **4** (300 MHz, CD_3OD, 298 K).

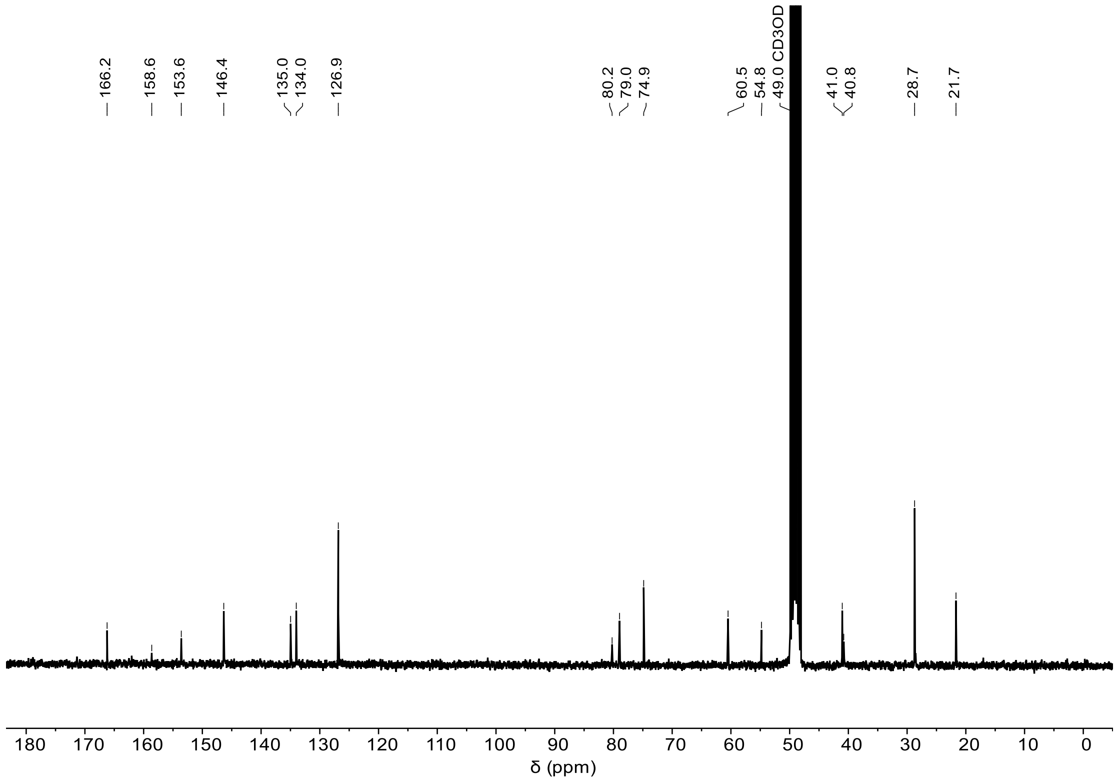

Supplementary Fig. 129: ^{13}C NMR of compound **4** (75 MHz, CD_3OD, 298 K).

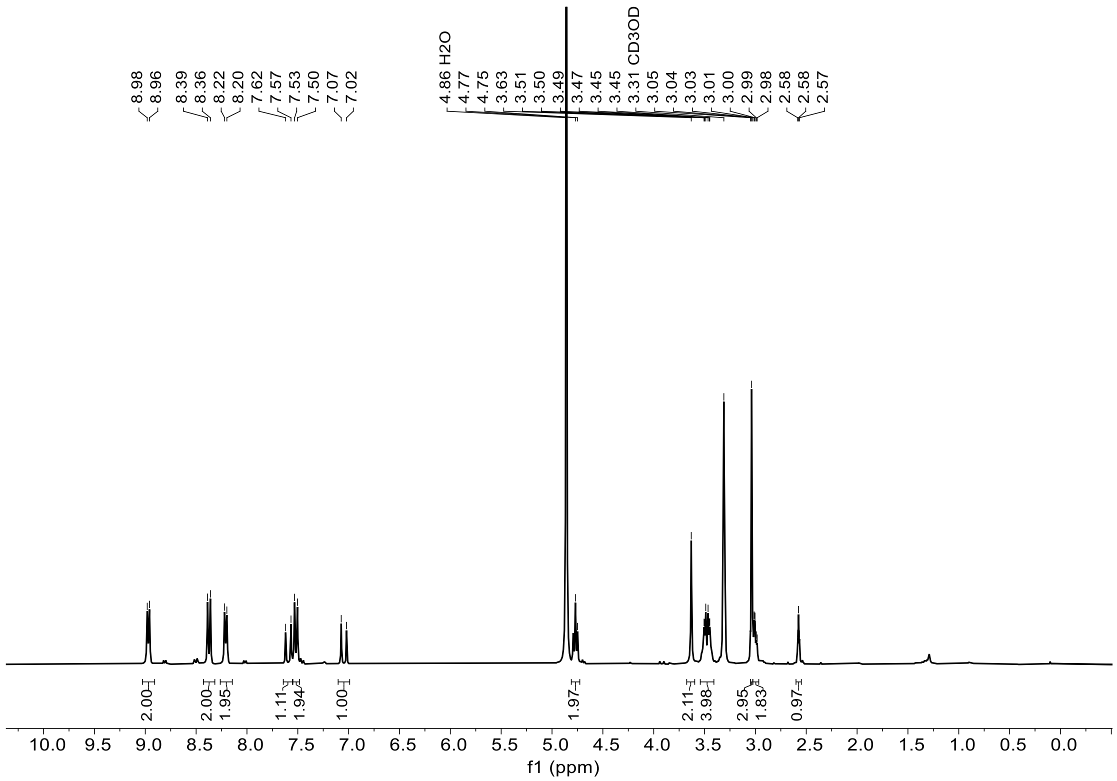

Supplementary Fig. 130: ^{1}H NMR of compound **5** (300 MHz, CD_3OD, 298 K).

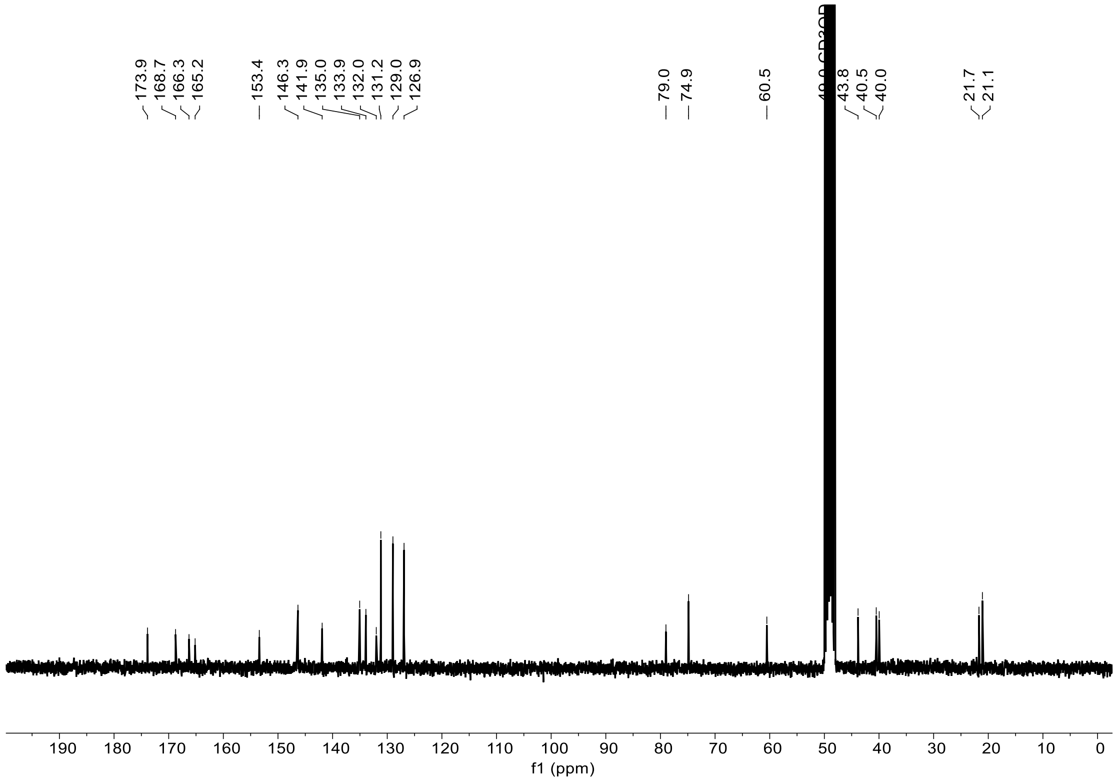

Supplementary Fig. 131: ^{13}C NMR of compound **5** (75 MHz, CD_3OD, 298 K).

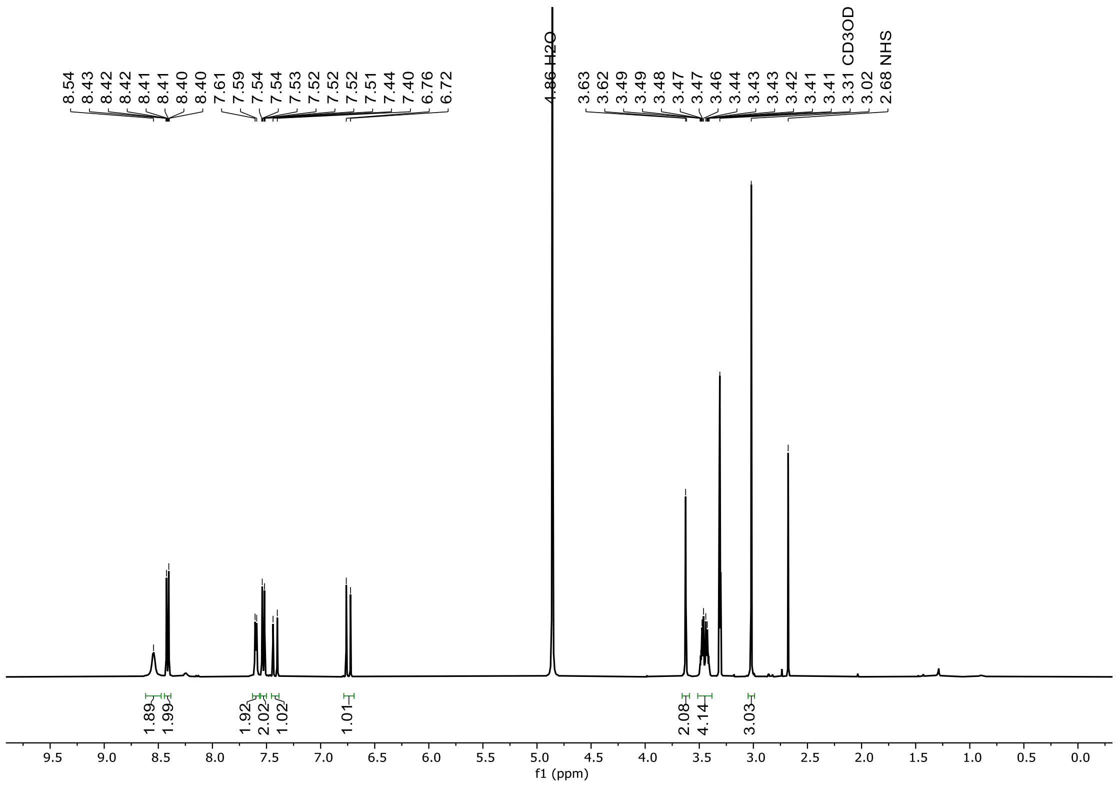

Supplementary Fig. 132: ^{1}H NMR of compound **18** (300 MHz, CD_3OD, 298 K).

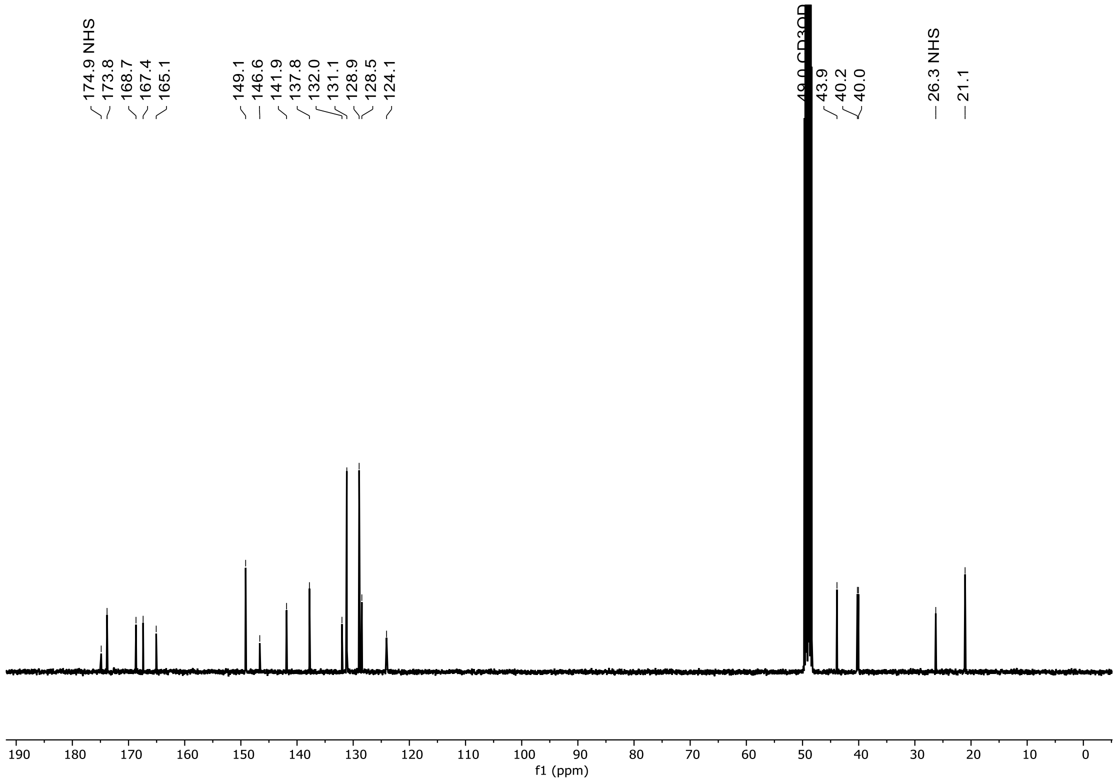

Supplementary Fig. 133: ^{13}C NMR of compound **18** (75 MHz, CD_3OD, 298 K).

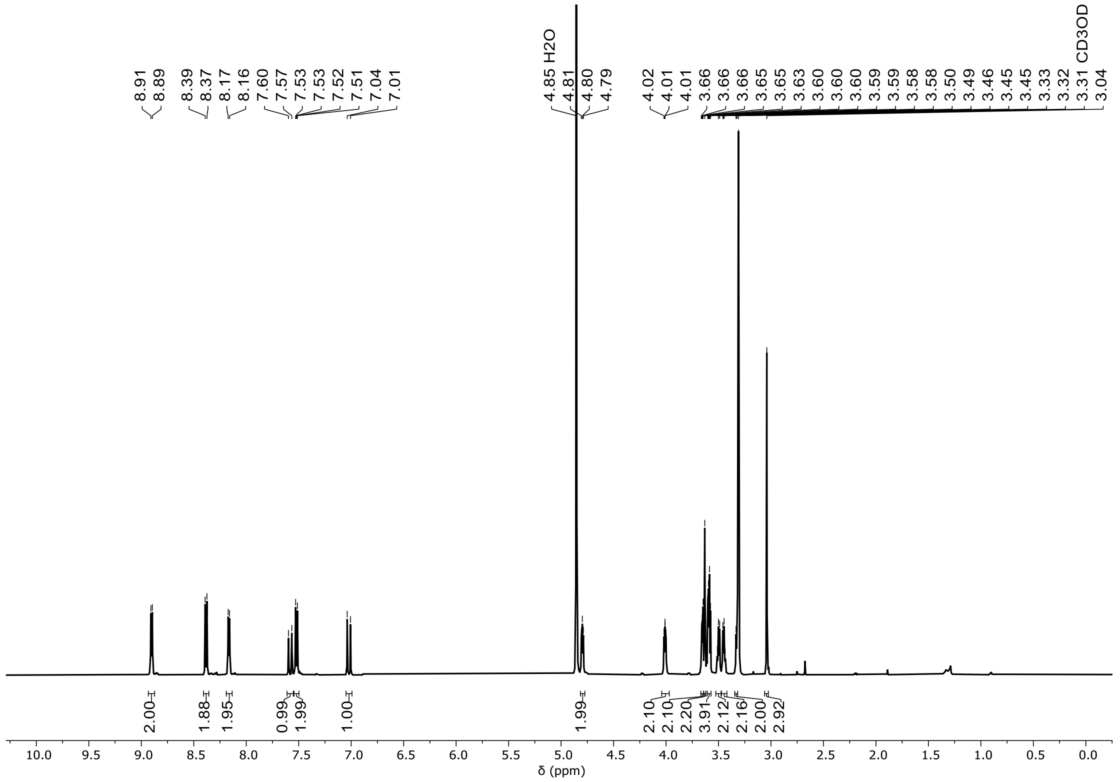

Supplementary Fig. 134: ^{1}H NMR of compound **6** (300 MHz, CD_3OD, 298 K).

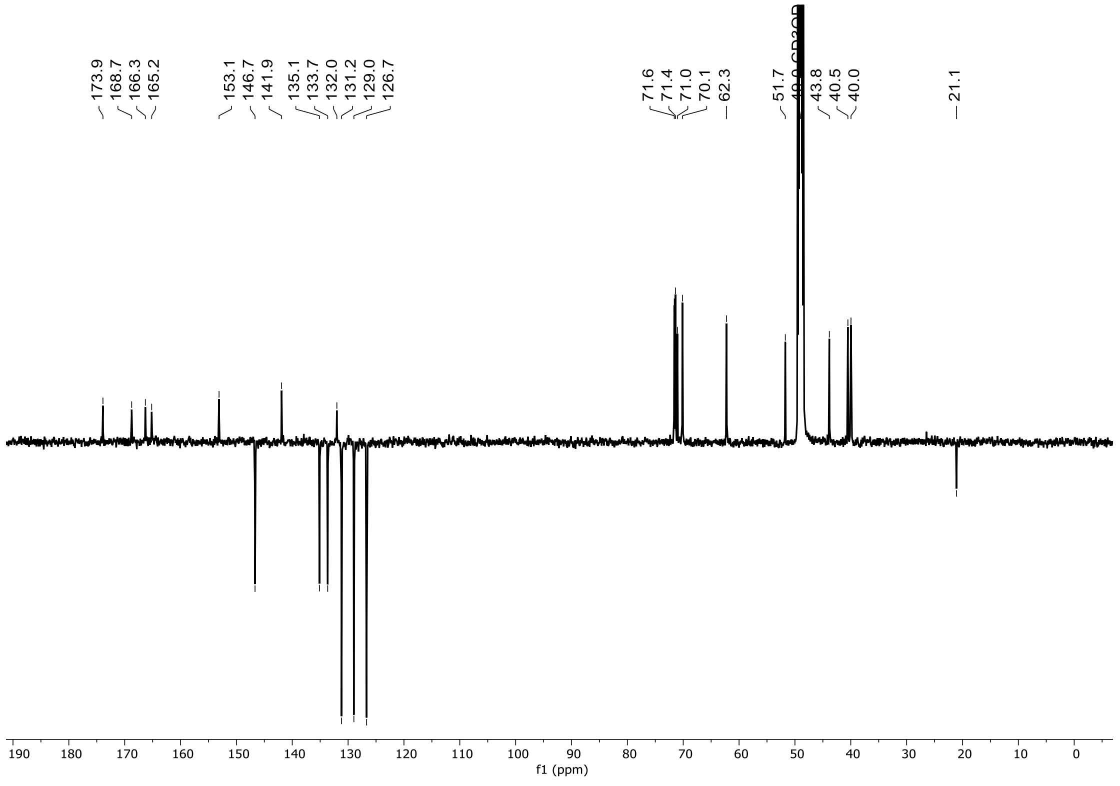

Supplementary Fig. 135: ^{13}C NMR of compound **6** (75 MHz, CD_3OD, 298 K).

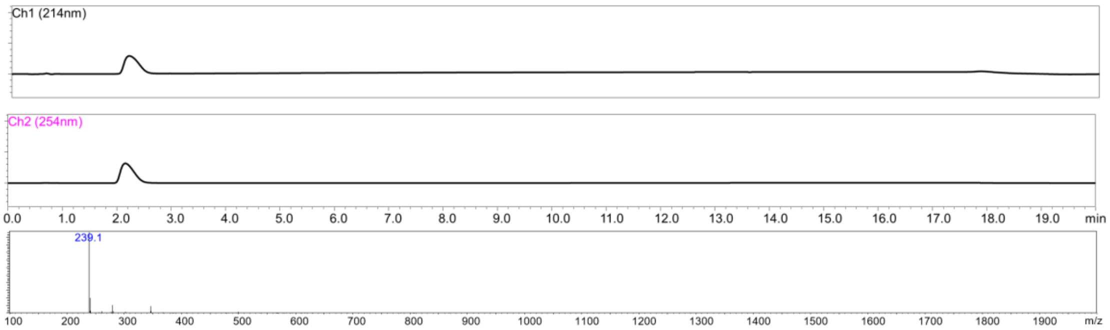

Supplementary Fig. 136: LC-MS analysis of compound **12**.

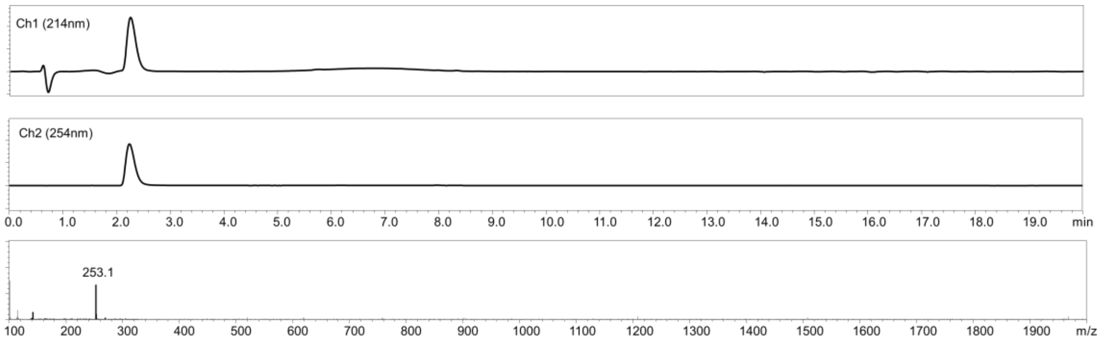

Supplementary Fig. 137: LC-MS analysis of compound **1**.

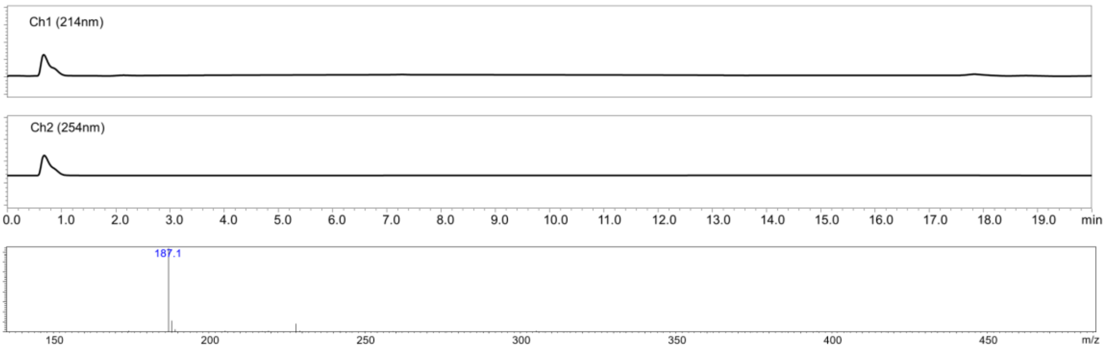

Supplementary Fig. 138: LC-MS analysis of compound **13**.

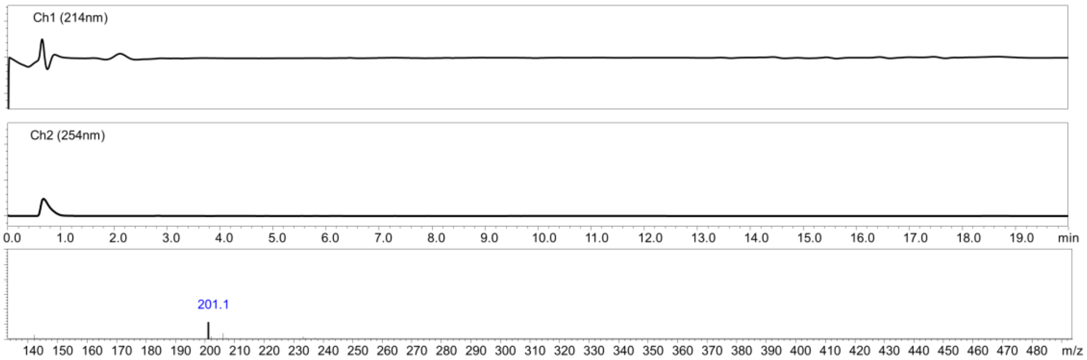

Supplementary Fig. 139: LC-MS analysis of compound **2**.

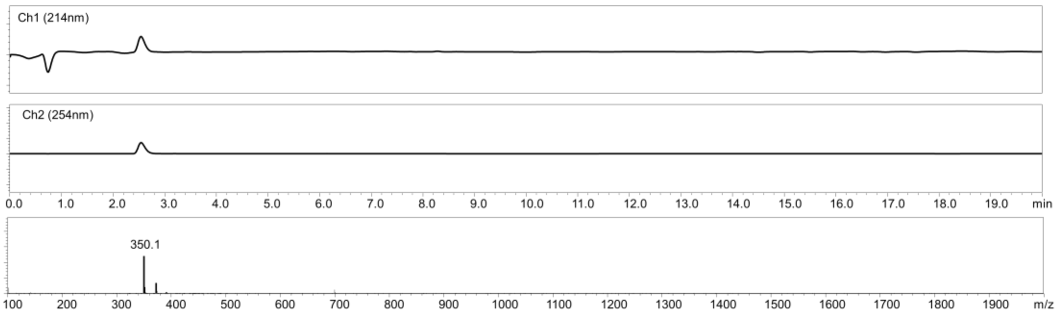

Supplementary Fig. 140: LC-MS analysis of compound **14**.

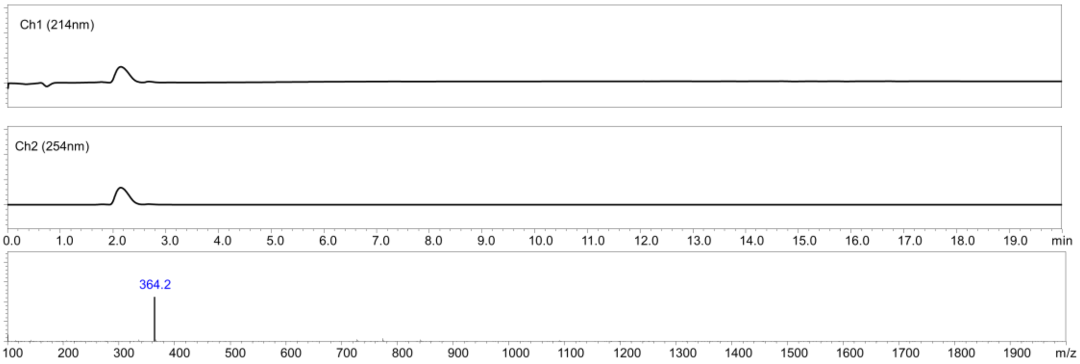

Supplementary Fig. 141: LC-MS analysis of compound **3**.

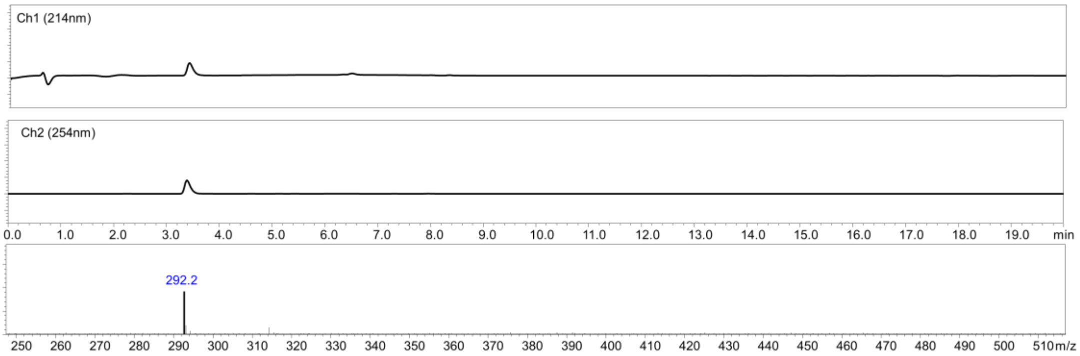

Supplementary Fig. 142: LC-MS analysis of compound **15**.

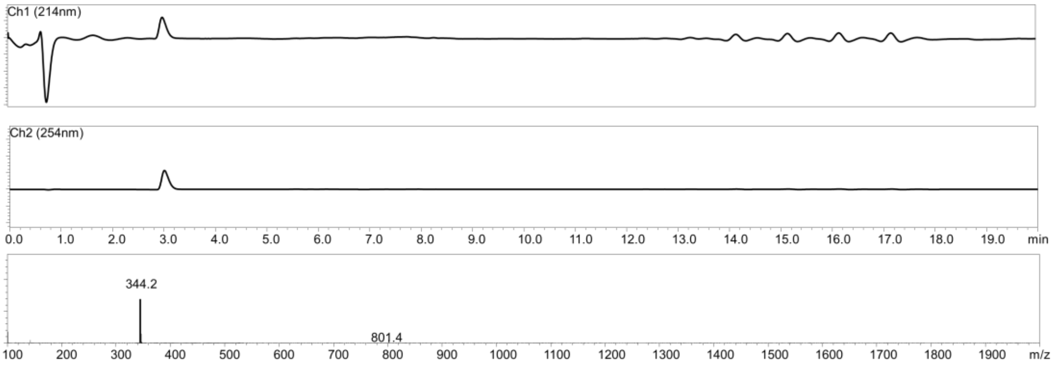

Supplementary Fig. 143: LC-MS analysis of compound **4**.

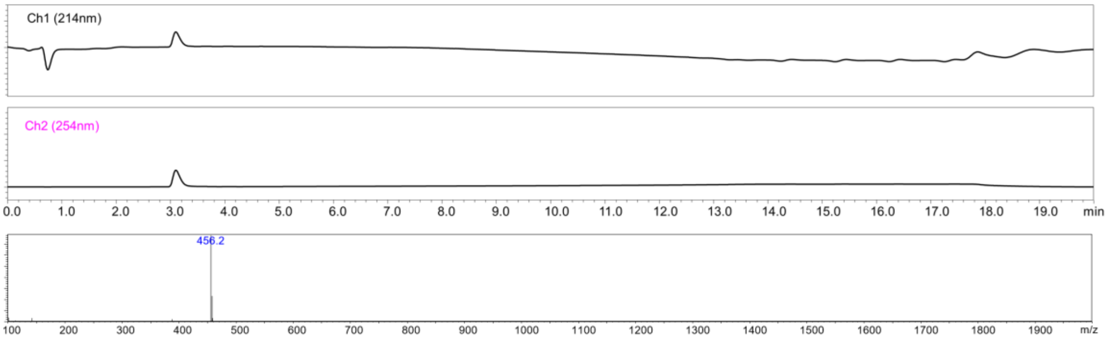

Supplementary Fig. 144: LC-MS analysis of compound **5**.

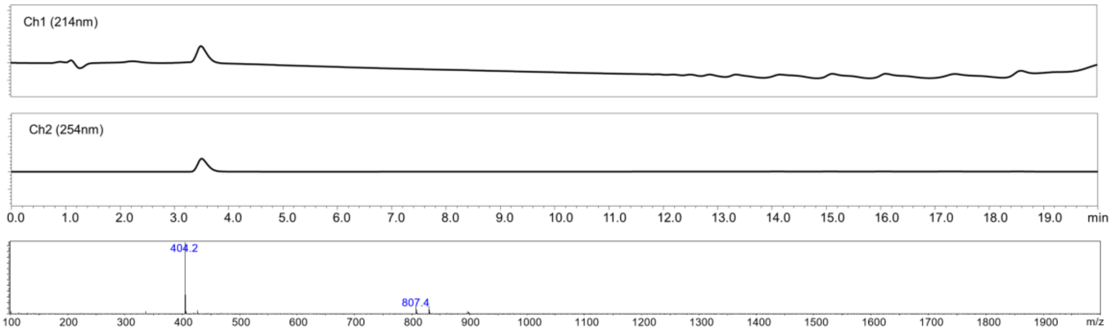

Supplementary Fig. 145: LC-MS analysis of compound **18**.

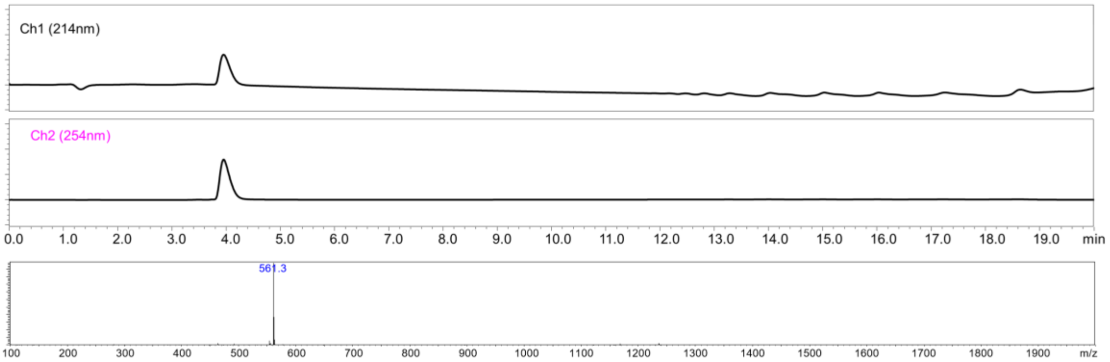

Supplementary Fig. 146: LC-MS analysis of compound **6**.

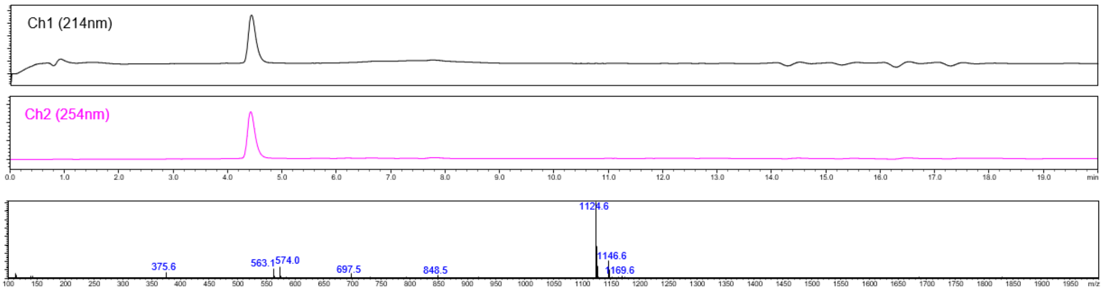

Supplementary Fig. 147: LC-MS analysis of RGDC-thiosuccinimide-DBCO.TFA^{-}.

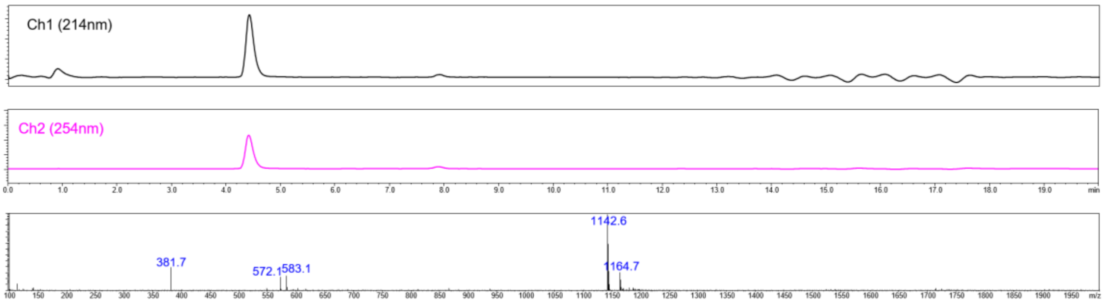

Supplementary Fig. 148: LC-MS analysis of RGDC-DBCO.TFA$^-$.

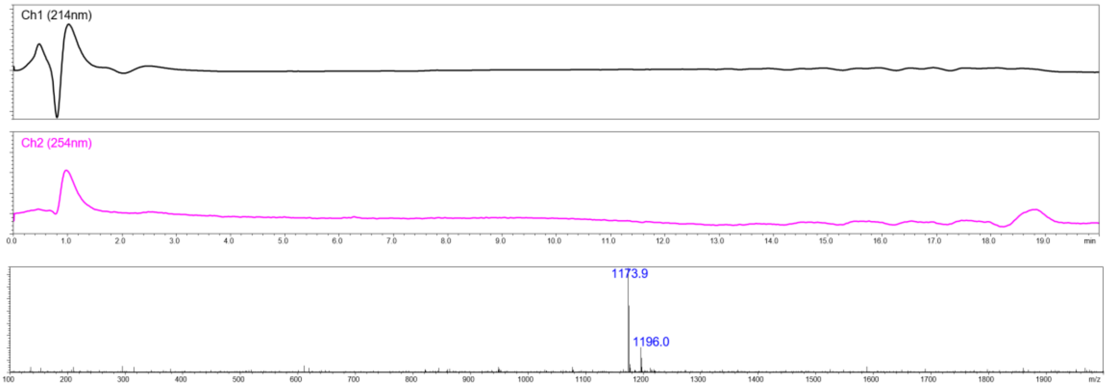

Supplementary Fig. 149: LC-MS analysis of PC8 maleimide conjugate.

12 Supplementary References

1. Wang, T. *et al.* Water-soluble allyl sulfones for dual site-specific labelling of proteins and cyclic peptides. *Chem. Sci.* **7**, 3234-3239 (2016).
2. Gil de Montes, E. *et al.* Azabicyclic vinyl sulfones for residue-specific dual protein labelling. *Chem. Sci.* **10**, 4515-4522 (2019).
3. Maruani, A. *et al.* A plug-and-play approach to antibody-based therapeutics via a chemoselective dual click strategy. *Nat. Commun.* **6**, 6645 (2015).
4. Gaussian 16, Revision C.01, M. J. Frisch, G. W. Trucks, H. B. Schlegel, G. E. Scuseria, M. A. Robb, J. R. Cheeseman, G. Scalmani, V. Barone, G. A. Petersson, H. Nakatsuji, X. Li, M. Caricato, A. V. Marenich, J. Bloino, B. G. Janesko, R. Gomperts, B. Mennucci, H. P. Hratchian, J. V. Ortiz, A. F. Izmaylov, J. L. Sonnenberg, D. Williams-Young, F. Ding, F. Lipparini, F. Egidi, J. Goings, B. Peng, A. Petrone, T. Henderson, D. Ranasinghe, V. G. Zakrzewski, J. Gao, N. Rega, G. Zheng, W. Liang, M. Hada, M. Ehara, K. Toyota, R. Fukuda, J. Hasegawa, M. Ishida, T. Nakajima, Y. Honda, O. Kitao, H. Nakai, T. Vreven, K. Throssell, J. A. Montgomery, Jr., J. E. Peralta, F. Ogliaro, M. J. Bearpark, J. J. Heyd, E. N. Brothers, K. N. Kudin, V. N. Staroverov, T. A. Keith, R. Kobayashi, J. Normand, K. Raghavachari, A. P. Rendell, J. C. Burant, S. S. Iyengar, J. Tomasi, M. Cossi, J. M. Millam, M. Klene, C. Adamo, R. Cammi, J. W. Ochterski, R. L. Martin, K. Morokuma, O. Farkas, J. B. Foresman, and D. J. Fox, Gaussian, Inc., Wallingford CT, 2019.
5. CYLview20; Legault, C. Y., Université de Sherbrooke, 2020 (http://www.cylview.org)
6. Zhao, Y. & Truhlar, D.G. The M06 suite of density functionals for main group thermochemistry, thermochemical kinetics, noncovalent interactions, excited states, and transition elements: two new functionals and systematic testing of four M06-class functionals and 12 other functionals. *Theor. Chem. Acc.* **120**, 215-241 (2008).
7. Cancès, E., Mennucci, B. & Tomasi, J. A new integral equation formalism for the polarizable continuum model: Theoretical background and applications to isotropic and anisotropic dielectrics. *J. Chem. Phys.* **107**, 3032-3041 (1997).
8. Cossi, M., Barone, V., Mennucci, B. & Tomasi, J. Ab initio study of ionic solutions by a polarizable continuum dielectric model. *Chem. Phys. Lett.* **286**, 253-260 (1998).
9. Mennucci, B. & Tomasi, J. Continuum solvation models: A new approach to the problem of solute's charge distribution and cavity boundaries. *J. Chem. Phys.* **106**, 5151-5158 (1997).
10. Tomasi, J., Mennucci, B. & Cammi, R. Quantum Mechanical Continuum Solvation Models. *Chem. Rev.* **105**, 2999-3094 (2005).
11. Nuhn, L. *et al.* Targeting Protumoral Tumor-Associated Macrophages with Nanobody-Functionalized Nanogels through Strain Promoted Azide Alkyne Cycloaddition Ligation. *Bioconjug. Chem.* **29**, 2394-2405 (2018).
12. Fahrer, J. *et al.* Selective and specific internalization of clostridial C3 ADP-ribosyltransferases into macrophages and monocytes. *Cell. Microbiol.* **12**, 233-247 (2010).
13. Gačanin, J. *et al.* Spatiotemporally Controlled Release of Rho-Inhibiting C3 Toxin from a Protein–DNA Hybrid Hydrogel for Targeted Inhibition of Osteoclast Formation and Activity. *Adv. Healthc. Mater.* **6**, 1700392 (2017).
14. Fellermann, M. *et al.* Clostridial C3 Toxins Enter and Intoxicate Human Dendritic Cells. *Toxins* **12**, 563 (2020).
