# Supplementary material for: Chemoselective dual functionalization of proteins via 1,6-addition of thiols to trifunctional N-alkylpyridinium
Source: Nat Commun. 2025 Jun 6;16:5278. doi: 10.1038/s41467-025-60237-y (PMC12144261; doi:10.1038/s41467-025-60237-y)
Supplement: Supplementary file 2 — Description of Additional Supplementary Files [file 41467_2025_60237_MOESM2_ESM.pdf]

Description of Additional Supplementary Files

Supplementary Data 1: Cartesian coordinates (xyz), energies and relevant properties of all DFT optimized structures
